# Author Correction: Catalytic enantioselective reductive alkynylation of amides enables one-pot syntheses of pyrrolidine, piperidine and indolizidine alkaloids

**DOI:** 10.1038/s41467-024-46252-5

**Published:** 2024-02-28

**Authors:** Fang-Fang Xu, Jin-Quan Chen, Dong-Yang Shao, Pei-Qiang Huang

**Affiliations:** Department of Chemistry and Fujian Provincial Key Laboratory of Chemical Biology, College of Chemistry and Chemical Engineering, Xiamen University, Xiamen, Fujian 361005 PR China

**Keywords:** Drug discovery and development, Natural product synthesis, Organocatalysis

Correction to: *Nature Communications* 10.1038/s41467-023-41846-x, published online 06 October 2023

The original version of this Article contained an error in the second paragraph on page 3, which incorrectly read ‘Recently, we established an Ir/Cu tandemcatalysis protocol^39^ and an Ir/Cu/organocatalyst multicatalysis protocol^40^ for the enantioselective reductive alkynylation of amides (Fig. 1b, c).’ The correct version states ‘Recently, Huang and Wang established’ in place of ‘Recently, we established’. This has been corrected in both the PDF and HTML versions of the Article.

