# Supplementary material for: Catalytic enantioselective reductive alkynylation of amides enables one-pot syntheses of pyrrolidine, piperidine and indolizidine alkaloids
Source: Nat Commun. 2023 Oct 6;14:6251. doi: 10.1038/s41467-023-41846-x (PMC10558451; doi:10.1038/s41467-023-41846-x)
Supplement: Supplementary file 1 — Supplementary Information [file 41467_2023_41846_MOESM1_ESM.pdf]

Supplementary Information

Article

Catalytic Enantioselective Reductive Alkynylation of Amides Enables One-Pot Syntheses of Pyrrolidine, Piperidine and Indolizidine Alkaloids

In the format provided by the authors and unedited

Catalytic Enantioselective Reductive Alkynylation of Amides Enables One-Pot Syntheses of Pyrrolidine, Piperidine and Indolizidine Alkaloids

Fang-Fang Xu, Jin-Quan Chen, Dong-Yang Shao, Pei-Qiang Huang*

Department of Chemistry and Fujian Provincial Key Laboratory of Chemical Biology, College of Chemistry and Chemical Engineering, Xiamen University, Xiamen, Fujian 361005, P. R. China.
*email: pqhuang@xmu.edu.cn

Table of Contents

1. General information 3
Supplementary Fig 1. The Structures of All Amides Used 4
Supplementary Fig 2. The Structures of All Alkynes Used 4
2. Synthesis of Materials. 5
2.1 Synthesis of amides 5
2.2 Synthesis of alkyne **2l** 10
3. Supplementary Methods. 11
3.1 One-pot Catalytic Asymmetric Reductive Alkynylation of Tertiary Amides 11
3.2 One-pot Catalytic Asymmetric Synthesis of (–)-*cis*-2-Methyl-6-nonyl-piperidine Alkaloid [(–)-(2*S*,6*R*)-**A-3**]) 44
3.3 One-pot Catalytic Asymmetric Synthesis of (–)-*cis*-2-Methyl-6-undecyl-piperidine Alkaloid [(–)-(2*S*,6*R*)-**A-4**] 45
3.4 One-pot Catalytic Asymmetric Synthesis of Alkaloid (−)-*cis*-197F [(–)-(2*S*,6*R*)-**A-5**] 46
3.5 One-pot Catalytic Asymmetric Synthesis of Alkaloid (−)-*cis*-225C [(–)-(2*S*,5*R*)-**A-2**] 47
3.6 One-pot Catalytic Asymmetric Synthesis of Alkaloid (−)-*cis*-1,2-Dimethyl-6-nonyl-piperidine [(−)-(2*S*,6*R*)-**A-6**] 48
3.7 One-pot Catalytic Asymmetric Synthesis of Alkaloid (–)-Bgugaine [(–)-(*R*)-**A-1**)] 48
3.8 One-pot Catalytic Asymmetric Synthesis of Alkaloid (+)-Bgugaine (*ent*-**A-1**) 49
3.9 One-pot Catalytic Asymmetric Synthesis of Alkaloid (–)-209D [(–)-**A-7**] 50
3.10 One-pot Catalytic Asymmetric Synthesis of Alkaloid (+)-Monomorine I [(+)-**A-8**)] 51
3.11 One-pot Catalytic Asymmetric Synthesis of Intermediate **4a** 52
4. Supplementary Notes 54
4.1 NMR Spectra of Compounds 54
5. Supplementary References 108

1. General information

All reactions were performed anhydrously under nitrogen atmosphere. All reagents were purchased from commercial suppliers without further purification. Solvent purification was conducted according to Purification of Laboratory Chemicals (Peerrin, D. D.; Armarego, W. L. and Perrins, D. R., Pergamon Press: Oxford, 1980). Yields were calculated based on the weights of chromatographically isolated products. Reactions were monitored by thin-layer chromatography (TLC) on plates (GF254) supplied by Yantai Chemicals (China). The TLC spots were visualized under ultraviolet light or by staining with an ethanolic solution of phosphomolybdic acid and cerium sulfate or iodine vapor. Flash column chromatography was performed using silica gel (200-300 mesh) from Qingdao Haiyang Chemicals. Melting points were determined on a Büchi M560 Automatic Melting Point apparatus and are uncorrected. NMR spectra were recorded on Bruker AV III 400 and Bruker AV III 500 instruments, and calibrated with tetramethylsilane (TMS) (δ H = 0.00 ppm.) and $CDCl_3$ (δ C = 77.00 ppm.) as internal references. Multiplicities were designated as follows: s = singlet, d = doublet, t = triplet, q = quartet, br = broad, dd = double doublet, td = triple doublet, dt = double triplet, dq = double quartet, m = multiplet. Infrared (IR) spectra were measured on a Nicolet FT-380 spectrometer using film KBr pellet techniques. High-resolution mass spectra analyses were performed on a Fourier transform ion cyclotron resonance (FT-ICR) mass spectrometer (Bruker Daltonics) with a 7-T magnet (Magnex) and an electrospray ionization (ESI) source (Apollo II, Bruker Daltonics) under positive-ion mode. Optical rotations were measured on an Anton Paar MCP-500 polarimeter. THF and toluene were distilled over sodium benzophenone ketyl under N2. Dichloromethane was distilled over calcium hydride under N2. Chiral HPLC analysis was performed on an Agilent LC1260 instrument. The absolute configuration of the known products was assigned by comparison of optical rotation with the literature, while the absolute configuration of the new products with similar substitutions was assigned by analogy, based on the sign of their specific rotations and the assumption that the sense of asymmetric induction was the same as for all other derivatives, both corroborate each other. Amides **1a**[1], **1b**[1], **1c**[2b], **1e**[2a], **1f**[3], **1g**[4], **1j**[5], **1k**[6], **1n**[7a], **1p**[7b], alkyne **2i**[8], were prepared according to the procedures described in the literature. All other commercially available compounds were used as received.

Supplementary Fig 1. The Structures of All Amides Used

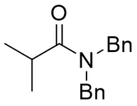
1a
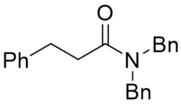
1b
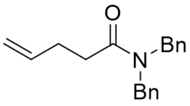
1c
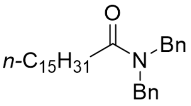
1d
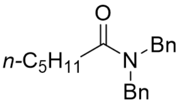
1e
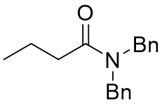
1f

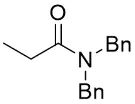
1g
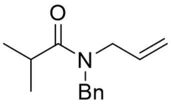
1h
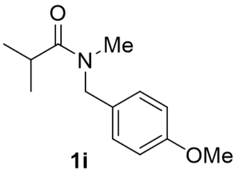
1i
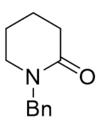
1j
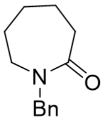
1k
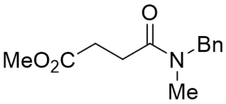
1l

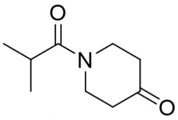
1m
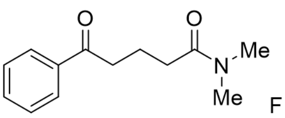
1n
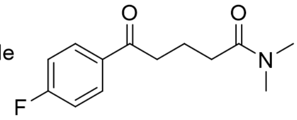
1o
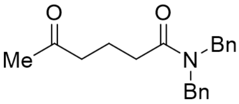
1p
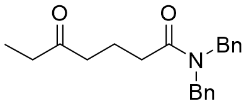
1q

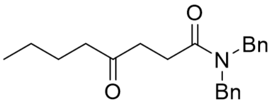
1r
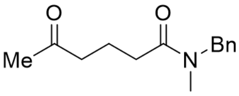
1s
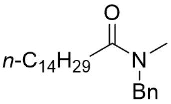
1t
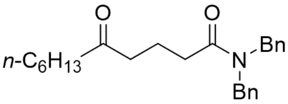
1u

Supplementary Fig 2. The Structures of All Alkynes Used

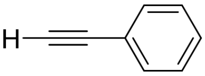
2a
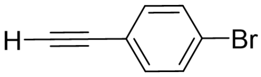
2b
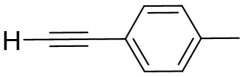
2c
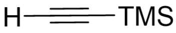
2d

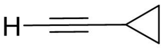
2e
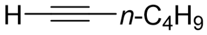
2f
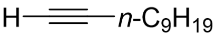
2g
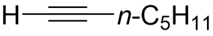
2h

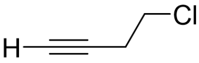
2i
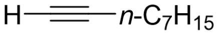
2j
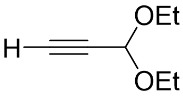
2k
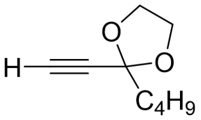
2l

2. Synthesis of Materials.

2.1 Synthesis of amides

General Procedure A (Preparation of Amides)

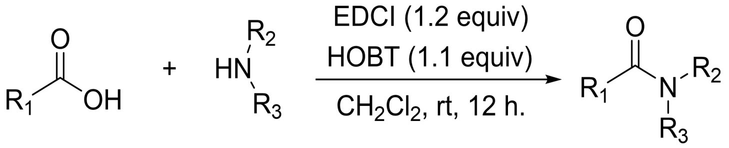

To a stirring solution of a carboxylic acid (20 mmol, 1.0 equiv), HOBT (2.97 g, 22 mmol, 1.1 equiv) and *N*-ethyl-*N'*-(3-dimethylaminopropyl)carbodiimide hydrochloride (EDCI, 4.6 g, 24 mmol, 1.2 equiv) in CH_2Cl_2 (60 mL) was added an amine (24 mmol, 1.2 equiv). The reaction mixture was stirred at room temperature for 12 h and then CH_2Cl_2 (50 mL) and water (50 mL) were added. The organic layer was separated and washed successively with 1 N HCl (3 × 50 mL), and sat. aqueous $NaHCO_3$ (50 mL). The aqueous phase was extracted with CH_2Cl_2 (3 × 20 mL). The combined organic phases were dried over anhydrous Na_2SO_4, filtered, and concentrated under reduced pressure. The residue was purified by flash column chromatography on silica gel eluting with an ethyl acetate/hexane mixture to give the desired amide **1**.

N,N-Dibenzylpalmitamide (1d)

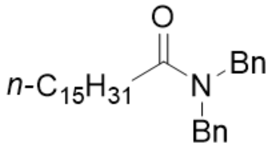

Following general procedure A, amide **1d** was obtained as a white solid in 81% yield; M. p. 48 – 49 ˚C; **IR** (film) $\tilde{v}$: 2923, 2852, 1651, 1495, 1465, 1453, 1420, 1361, 1209, 1078 cm^{-1}; **^{1}H NMR** (400 MHz, Chloroform-*d*) δ 7.38 – 7.24 (m, 6H), 7.21 (d, *J* = 7.4 Hz, 2H), 7.14 (d, *J* = 7.4 Hz, 2H), 4.60 (s, 2H), 4.44 (s, 2H), 2.41 (t, *J* = 7.6 Hz, 2H), 1.82–1.69 (m, 2H), 1.37 – 1.19 (m, 24H), 0.88 (t, *J* = 6.7 Hz, 3H) ppm; **^{13}C NMR** (100 MHz, Chloroform-*d*) δ 173.7, 137.5, 136.6, 128.9, 128.5, 128.2, 127.5, 127.3, 126.3, 49.9, 47.9, 33.2, 31.9, 29.6 (4C), 29.5 (2C), 29.4 (2C), 29.3 (2C), 25.4, 22.6, 14.1 ppm; **HRMS** (ESI) *m/z* for $C_{30}H_{46}NO$ ($[M+H]^+$): 436.3574; Found: 436.3578.

N-Allyl-*N*-benzylisobutyramide (1h)

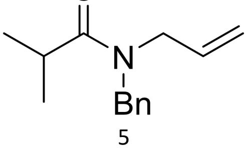

Following general procedure A, amide **1h** was obtained as a colorless oil in 75% yield; **IR** (film) $\tilde{\nu}$: 2967, 2931, 2871, 1651, 1495, 1454, 1435, 1360, 1218, 1168, 1087, 1029, 924, 721 cm^{-1}; ^{1}**H NMR** (400 MHz, Chloroform-*d*, data of two rotamers in a ratio of 3: 2, *M* = major rotamer; *m* = minor rotamer) δ 7.42 – 7.07 (m, 5H), 5.90 – 5.60 (m, 1H), 5.26 – 5.04 (m, 2H), 4.60 (s, 2H, *M*), 4.55 (s, 2H, *m*), 4.01 (d, *J* = 5.9 Hz, 2H, *m*), 3.90 – 3.80 (d, *J* = 4.8 Hz, 2H, *M*), 2.87 – 2.71 (m, 1H), 1.19 (d, *J* = 6.6 Hz, 3H, *M* + *m*), 1.15 (d, *J* = 6.6 Hz, 3H, *M* + *m*) ppm; ^{13}**C NMR** (100 MHz, Chloroform-*d*) δ 177.5 (*M*), 177.3 (*m*), 137.7 (*M*), 137.0 (*m*), 133.0, 128.7, 128.4, 127.8, 127.3, 127.0, 126.0, 116.9 (*m*), 116.3 (*M*), 49.6 (*m*), 48.6 (*M*), 47.9 (*M*), 47.7 (*m*), 30.3 (*m*), 30.0 (*M*), 19.6 (2C) ppm; **HRMS** (ESI) *m/z* for $C_{14}H_{20}NO$ ([M+H]$^{+}$): 218.1539; Found: 218.1543.

***N*-(4-Methoxybenzyl)-*N*-methylisobutyramide (1i)**

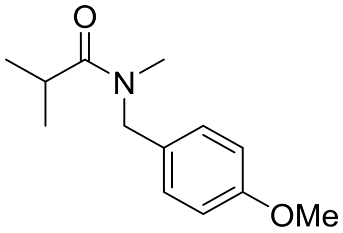

Following general procedure A, amide **1i** was obtained as a pale yellow oil in 83% yield; **IR** (film) $\tilde{\nu}$: 2968, 2933, 2837, 1639, 1512, 1462, 1409, 1247, 1175, 1109, 1087, 1033 cm^{-1}; ^{1}**H NMR** (400 MHz, Chloroform-*d*, data of two rotamers in a ratio of 3: 2, *M* = major rotamer; *m* = minor rotamer) δ 7.16 (d, *J* = 8.2 Hz, 2H, *M*), 7.08 (d, *J* = 8.1 Hz, 2H, *m*), 6.88 (d, *J* = 8.4 Hz, 2H, *m*), 6.84 (d, *J* = 8.3 Hz, 2H, *M*), 4.52 (s, 2H, *M*), 4.51 (s, 2H, *m*), 3.78 (s, 3H, *m*), 3.77 (s, 3H, *M*), 2.92 (s, 3H, *M*), 2.91 (s, 3H, *m*), 2.89 – 2.76 (m, 1H), 1.16 (d, *J* = 6.4 Hz, 3H, *M* + *m*), 1.14 (d, *J* = 6.4 Hz, 3H, *M* + *m*) ppm; ^{13}**C NMR** (100 MHz, Chloroform-*d*) δ 177.3 (*m*), 176.6 (*M*), 158.8 (*m*), 158.6 (*M*), 129.5 (*m*), 128.9 (*M*), 128.6 (*m*), 127.2 (*M*), 114.0 (*m*), 113.6 (*M*), 55.0 (*m*), 54.9 (*M*), 52.2 (*m*), 49.8 (*M*), 34.0 (*M*), 33.5 (*m*), 30.1 (*M*), 30.0 (*m*), 19.5, 18.9 ppm; **HRMS** (ESI) *m/z* for $C_{13}H_{20}NO_2$ ([M+H]$^{+}$): 222.1489; Found: 222.1481.

Methyl 4-(benzyl(methyl)amino)-4-oxobutanoate (1l)

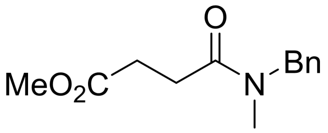

Following general procedure A, amide **1l** was obtained as a colorless oil in 70% yield; **IR** (film) $\tilde{\nu}$: 2951, 1735, 1647, 1495, 1452, 1411, 1368, 1219, 1170, 1120, 1028 cm^{-1}; ^{1}**H NMR** (400 MHz, Chloroform-*d*, data of two rotamers in a ratio of 3: 2, *M* = major rotamer; *m* = minor rotamer) δ 7.40 – 7.13 (m, 5H), 4.57 (s, 2H, *M*), 4.54 (s, 2H, *m*), 3.67 (s, 3H, *M*), 3.66 (s, 3H, *m*), 2.92 (s, 3H, *m*), 2.92 (s, 3H, *M*), 2.74 – 2.60 (m, 4H) ppm; ^{13}**C NMR** (100 MHz, Chloroform-*d*) δ 173.1 (*M*), 173.0 (*m*), 171.1 (*m*), 170.9 (*M*),

136.9 (*M*), 136.0 (*m*), 128.5 (*m*), 128.1 (*M*), 127.5, 127.1 (*m*), 126.8 (*M*), 125.9, 52.6 (*m*), 51.2 (*M*), 50.4, 34.1 (*M*), 33.4 (*m*), 28.7, 27.8 (*M*), 27.4 (*m*) ppm; **HRMS** (ESI) *m/z* for $C_{13}H_{18}NO_3$ ($[M+H]^+$): 236.1281; Found: 236.1287.

1-Isobutyrylpiperidin-4-one (1m)

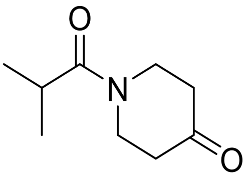

Following general procedure A, amide **1m** was obtained as a pale yellow oil in 70% yield; **IR** (film) $\tilde{\nu}$: 2930, 2854, 1715, 1644, 1446, 1362, 1316, 1261, 1202, 1175, 1087, 1052 cm^{-1}; **^{1}H NMR** (400 MHz, Chloroform-*d*) δ 3.98 – 3.74 (m, 4H), 3.00 – 2.80 (m, 1H), 2.67 – 2.40 (m, 4H), 1.19 (d, $J = 6.7$ Hz, 3H), 1.18 (d, $J = 6.6$ Hz, 3H) ppm; **^{13}C NMR** (100 MHz, Chloroform-*d*) δ 206.9, 175.7, 43.8 (2C), 41.4, 40.9, 30.1, 19.4 (2C) ppm; **HRMS** (ESI) *m/z* for $C_9H_{16}NO_2$ ($[M+H]^+$): 170.1176; Found: 170.1171.

5-(4-Fluorophenyl)-*N*,*N*-dimethyl-5-oxopentanamide (1o)

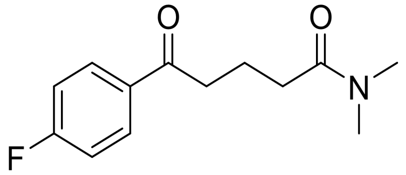

Following general procedure A, amide **1o** was obtained as a pale yellow oil in 87% yield; **IR** (film) $\tilde{\nu}$: 2930, 2854, 1715, 1644, 1446, 1362, 1316, 1261, 1202, 1175, 1087, 1052 cm^{-1}; **^{1}H NMR** (400 MHz, Chloroform-*d*) δ 8.05-8.00 (m, 2H), 7.17 – 7.06 (m, 2H), 3.07 (t, $J = 6.9$ Hz, 2H), 3.01 (s, 3H), 2.95 (s, 3H), 2.44 (t, $J = 7.0$ Hz, 2H), 2.15 – 2.01 (m, 2H) ppm; **^{13}C NMR** (100 MHz, Chloroform-*d*) δ 198.5, 172.3, 165.6 (d, $J = 254.5$ Hz), 133.2 (d, $J = 3.1$ Hz), 130.7 (d, $J = 9.3$ Hz), 115.5 (d, $J = 21.8$ Hz), 37.7, 37.1, 35.2, 32.1, 19.5 ppm; **^{19}F NMR** (471 MHz, Chloroform-*d*) δ – 105.49; **HRMS** (ESI) *m/z* for $C_{13}H_{17}PNO_2$ ($[M+H]^+$):238.1238; Found: 238.1230.

***N*-Benzyl-*N*-methyl-5-oxohexanamide (1s)**

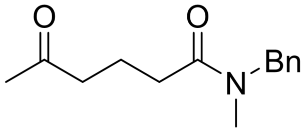

Following general procedure A, amide **1s** was obtained as a pale yellow oil in 75% yield; **IR** (film) $\tilde{\nu}$: 2937, 1709, 1632, 1495, 1453, 1407, 1358, 1261, 1159, 1121, 1063 cm^{-1}; **^{1}H NMR** (400 MHz, Chloroform-*d*, data of two rotamers in a ratio of 3: 2, *M* = major rotamer; *m* = minor rotamer) 7.38 –

7.27 (m, 3H), 7.22 (d, *J* = 8.0 Hz, 1H), 7.15 (d, *J* = 8.0 Hz, 1H), 4.58 (s, 2H, *M*), 4.54 (s, 2H, *m*), 2.94 (s, 3H, *m*), 2.91 (s, 3H, *M*), 2.58 (t, *J* = 6.9 Hz, 2H, *M*), 2.53 (t, *J* = 6.9 Hz, 2H, *m*), 2.42 (t, *J* = 7.0 Hz, 2H, *M*), 2.39 (t, *J* = 7.0 Hz, 2H, *m*), 2.15 (s, 3H, *M*), 2.11 (s, 3H, *m*), 1.99 – 1.89 (m, 2H) ppm; **^{13}C NMR** (100 MHz, Chloroform-*d*) δ 208.6 (*M*), 208.5 (*m*), 172.7 (*m*), 172.3 (*M*), 137.3 (*M*), 136.6 (*m*), 128.8 (*m*), 128.5 (*M*), 127.9, 127.5 (*m*), 127.2 (*M*), 126.2, 53.2 (*m*), 50.7 (*M*), 42.7, 34.7 (*M*), 33.8 (*m*), 32.3 (*M*), 31.9 (*m*), 29.8 (*M*), 29.7 (*m*), 19.2 (*m*), 19.0 (*M*) ppm; **HRMS** (ESI) *m/z* for $C_{14}H_{20}NO_2$ ($[M+H]^+$): 234.1489; Found: 234.1481.

N-Benzyl-*N*-methylpentadecanamide (1t)

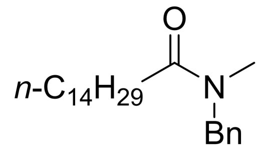

Following general procedure A, amide **1t** was obtained as a pale yellow oil in 67% yield; **IR** (film) $\tilde{\nu}$: 2923, 2852, 1729, 1651, 1466, 1454, 1402, 1262, 1105, 1029 cm^{-1}; **^{1}H NMR** (400 MHz, Chloroform-*d*, data of two rotamers in a ratio of 11: 8, *M* = major rotamer; *m* = minor rotamer) δ 7.37 – 7.22 (m, 4H), 7.16 (d, *J* = 7.5 Hz, 1H), 4.59 (s, 1H, *M*), 4.53 (s, 1H, *m*), 2.94 (s, 3H, *m*), 2.91 (s, 3H, *M*), 2.37(d, *J* = 8.0 Hz, 2H), 1.82 – 1.52 (m, 2H), 1.40 – 1.20 (m, 22H), 0.88 (t, *J* = 6.6 Hz, 3H) ppm; **^{13}C NMR** (100 MHz, Chloroform-*d*) δ 173.6 (*M*), 173.3 (*m*), 137.5 (*m*), 136.7 (*M*), 128.8 (m), 128.5 (M), 127.9 (M), 127.5 (m), 127.2 (*m*), 126.2 (*M*), 53.3, 50.7, 34.7, 33.8, 33.5, 33.1, 31.8, 29.6, 29.5, 29.4, 29.3, 29.3, 25.4, 25.1, 22.6, 14.3 ppm; **HRMS** (ESI) *m/z* for $C_{23}H_{40}NO$ ($[M+H]^+$): 346.3104; Found: 346.3109.

General Procedure B (Preparation of Keto Amides) [9a]

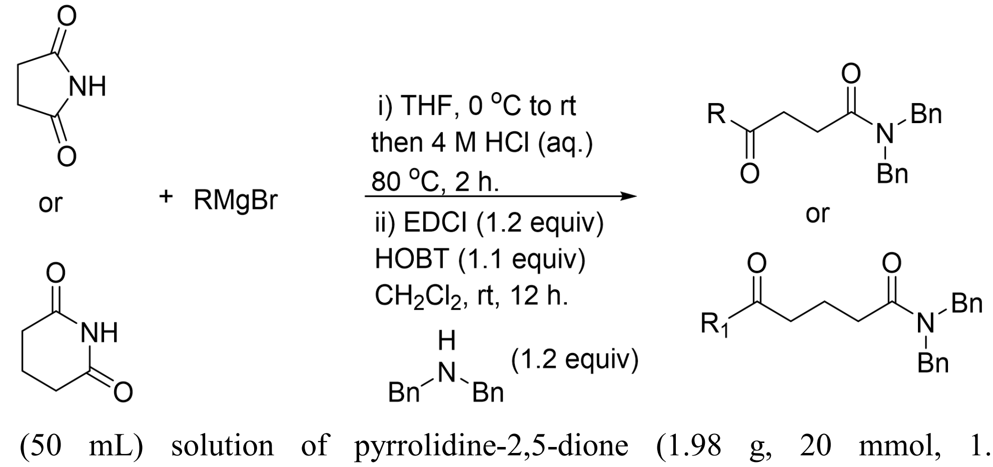

To a THF (50 mL) solution of pyrrolidine-2,5-dione (1.98 g, 20 mmol, 1.0 equiv) or piperidine-2,6-dione (2.26 g, 20.0 mmol, 1.0 equiv) was added slowly a Grignard reagent (3.0 equiv) at 0 °C. The mixture was stirred at 0 °C for 1 hour, and then at room temperature overnight. HCl (4 M, 300 mL) was added, and the mixture was stirred at 80 °C for 2 hours. Solid KOH was added to adjust

the pH to >13, and the mixture was extracted twice with Et_2O. The aqueous phase was separated and acidified to pH < 2 with conc. HCl, and extracted with Et_2O for three times. The combined organic phases were dried over Na_2SO_4, filtered, and the filtrate was concentrated under reduced pressure to give the corresponding keto carboxylic acid. To a stirring solution of a carboxylic acid (20 mmol), HOBT (2.97 g, 22 mmol) and *N*-(3-Dimethylaminopropyl)-*N*'-Ethylcarbodiimide Hydrochloride (EDCI, 4.6 g, 24 mmol) in CH_2Cl_2 (60 mL) was added dibenzylamine (24 mmol). The reaction was stirred at room temperature for 12 h and then CH_2Cl_2 (50 mL) and water (50 mL) were added. The organic layer was separated and washed successively with 1 M HCl (3 × 50 mL) and sat. aqueous $NaHCO_3$ (50 mL). The aqueous phase was extracted with CH_2Cl_2 (3 × 20 mL). The combined organic phases were dried over anhydrous Na_2SO_4, filtered, and concentrated under reduced pressure. The residue was purified by flash column chromatography on silica gel eluting with ethyl acetate/hexane to give the desired amide**.**

N,*N*-dibenzyl-5-oxoheptanamide (**1q**)

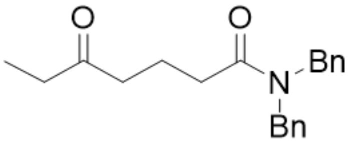

Following general procedure B, keto amide **1q** was obtained as a pale yellow oil with 56% yield(unoptimized); **IR** (film) $\tilde{\nu}$: 2974, 2936, 1712, 1651, 1495, 1452, 1362, 1206, 1114, 1077, 1028 cm^{-1}; **^{1}H NMR (500 MHz, Chloroform-*d*)** δ 7.37-7.32 (m, 2H), 7.31-7.29 (m, 4H), 7.22 – 7.19 (m, 2H), 7.15 – 7.12 (m, 2H), 4.60 (s, 2H), 4.45 (s, 2H), 2.51 (t, *J* = 7.0 Hz, 2H), 2.45 (t, *J* = 7.2 Hz, 2H), 2.40 (q, *J* = 7.4 Hz, 2H), 2.01-1.95 (m, 2H), 1.03 (t, *J* = 7.3 Hz, 3H); **^{13}C NMR** (126 MHz, Chloroform-*d*) δ 211.1, 172.9, 136. 5, 128.8, 128.5, 128.1, 127.5, 127.3, 126.2, 49.7, 48.0, 41.2, 35.7, 32.1, 19.3, 7.7 ppm; **HRMS** (ESI) *m/z* for $C_{21}H_{26}NO_2$ ($[M+H]^+$):324.1958, Found: 324.1950;

***N*,*N*-Dibenzyl-4-oxooctanamide (1r)**

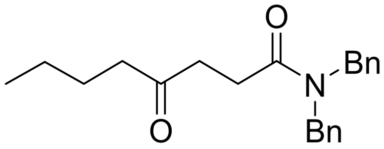

Following general procedure B, keto amide **1r** was obtained as a pale yellow oil in 55% yield (unoptimized); **IR** (film) $\tilde{\nu}$: 2957, 2930, 2871, 1712, 1647, 1495, 1452, 1362, 1213, 1166, 1127, 1078, 1043 cm^{-1}; **^{1}H NMR** (400 MHz, Chloroform-*d*) δ 7.38 – 7.31 (m, 2H), 7.31 – 7.23 (m, 4H), 7.22 – 7.14 (m, 4H), 4.58 (s, 2H), 4.49 (s, 2H), 2.81 (t, *J* = 6.2 Hz, 2H), 2.69 (t, *J* = 6.2 Hz, 2H), 2.50 (t, *J* = 7.5 Hz, 2H), 1.68–1.52 (m, 2H), 1.38 – 1.27 (m, 2H), 0.91 (t, *J* = 7.4 Hz, 3H) ppm; **^{13}C NMR** (100 MHz, Chloroform-*d*) δ 209.7, 172.0, 137.0, 136.2, 128.7, 128.3, 127.9, 127.3, 127.0, 126.3, 49.6, 48.0, 42.4,

37.0, 26.9, 25.7, 22.1, 13.6 ppm; **HRMS** (ESI) *m/z* for $C_{22}H_{28}NO_2$ ($[M+H]^+$): 338.2115; Found: 338.2110.

***N,N*-Dibenzyl-5-oxoundecanamide (1u)**

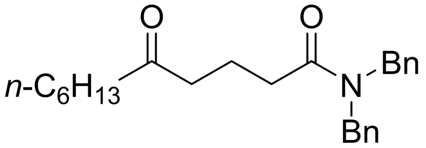

Following general procedure B, keto amide **1u** was obtained as a pale yellow oil in 66% yield; **IR** (film) $\tilde{v}$: 2954, 2929, 2858, 1709, 1647, 1466, 1419, 1364, 1295, 1218, 1094, 1079, 1028 cm^{-1}; **^{1}H NMR** (400 MHz, Chloroform-*d*) δ 7.39 – 7.25 (m, 6H), 7.20 (d, *J* = 6.7 Hz, 2H), 7.15 (d, *J* = 7.1 Hz, 2H), 4.60 (s, 2H), 4.46 (s, 2H), 2.52 (t, *J* = 7.0 Hz, 2H), 2.46 (t, *J* = 7.2 Hz, 2H), 2.38 (t, *J* = 7.5 Hz, 2H), 2.03 – 1.92 (m, 2H), 1.58 – 1.47 (m, 2H), 1.35–1.25 (m, 6H), 0.87 (t, *J* = 6.7 Hz, 3H) ppm; **^{13}C NMR** (100 MHz, Chloroform-*d*) δ 211.0, 173.1, 137.3, 136.5, 128.9, 128.6, 128.2, 127.5, 127.3, 126.3, 49.8, 48.1, 42.8, 41.7, 32.2, 31.5, 28.8, 23.7, 22.4, 19.4, 13.9 ppm; **HRMS** (ESI) *m/z* for $C_{25}H_{34}NO_2$ ($[M+H]^+$): 380.2584; Found: 380.2586.

2.2 Synthesis of alkyne 2l

2-Butyl-2-ethynyl-1,3-dioxolane (2l)

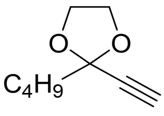

2l

Alkyne **2l** was prepared in one-pot from commercially available materials as a pale-yellow oil in 74% yield according to the procedures described in the literature with modification[9b]. **^{1}H NMR** (400 MHz, Chloroform-*d*) δ 4.18 – 4.07 (m, 2H), 4.06 – 3.96 (m, 2H), 2.51 (d, *J* = 2.9 Hz, 1H), 1.89 (t, *J* = 8.2 Hz, 2H), 1.59 – 1.51 (m, 2H), 1.44 – 1.34 (m, 2H), 0.92 (t, *J* = 7.5 Hz, 3H) ppm; **^{13}C NMR** (100 MHz, Chloroform-*d*) δ 103.0, 81.6, 77.2, 71.7, 64.6, 38.7, 25.9, 22.5, 13.9 ppm; **HRMS** (ESI) *m/z* for $C_9H_{15}O_2$ ($[M+H]^+$): 155.1067; Found: 155.1060.

3. Supplementary Methods.

3.1 One-pot Catalytic Asymmetric Reductive Alkynylation of Tertiary Amides

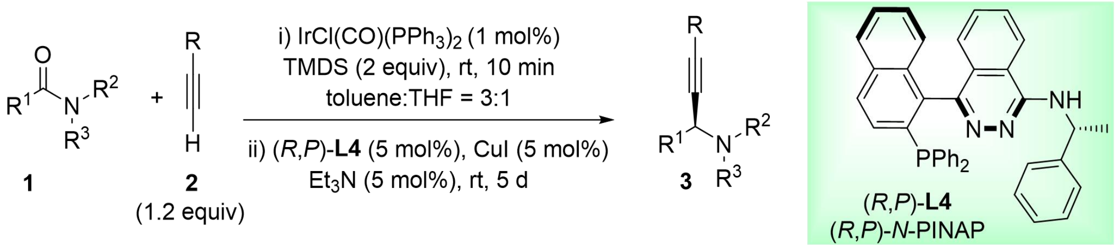

General procedure C:

To a flame-dried Schlenk tube were added CuI (3.8 mg, 0.02 mmol), (*R*,*P*)-*N*-PINAP [(*R*,*P*)-**L4**] (11.2 mg, 0.02 mmol) and toluene: THF = 3:1 (1 mL) under a N_2 atmosphere. After being stirred at room temperature for 5 min, triethylamine (6 µL, 0.02 mmol) and alkyne **2** (0.48 mmol) were added, and the resulting mixture was stirred at room temperature for 30 min.

To another flame-dried Schlenk tube were added sequentially $IrCl(CO)(PPh_3)_2$ (3.12 mg, 1 mol%), an amide **1** (0.4 mmol, 1 equiv), TMDS (144 µL, 0.8 mmol, 2 equiv) and toluene: THF = 3:1 (1 mL) under N_2 atmosphere at room temperature. After being stirred for 10 minutes, the resulting mixture was added to the abovementioned Schlenk tube containing CuI, (*R*,*P*)-**L4**, triethylamine and alkyne at 0 °C. The mixture was stirred at room temperature for 5 d. The reaction mixture was concentrated under reduced pressure, and the residue was purified by flash chromatography on silica gel eluting with petroleum ether/ethyl acetate to afford the corresponding chiral propargylic amine **3**.

(*S*)-(–)-*N*,*N*-Dibenzyl-4-methyl-1-phenylpent-1-yn-3-amine (3a)

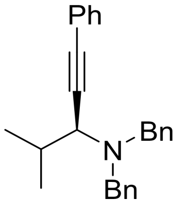

Following general procedure C, the reaction of amide **1a** (106.9 mg, 0.4 mmol) with alkyne **2a** (52 μL, 0.48 mmol) gave, after FC (eluent: EtOAc/*n*-hexane = 1: 200), propargylic amine **3a** (130 mg, yield: 92%, 98% *ee*) as a colorless oil; $[\alpha]_D^{25}$ = –333.8 (*c* 1, $CHCl_3$) {lit.: $[\alpha]_D^{28}$ = –312 (*c* 0.95, $CHCl_3$), 95% *ee*}[10a]; **IR** (film) $\tilde{v}$: 2957, 2925, 2807, 1946, 1598, 1489, 1453, 1363, 1266, 1069, 1028 cm^{-1}; **^{1}H NMR** (500 MHz, Chloroform-*d*) δ 7.54 – 7.49 (m, 2H), 7.43 (d, *J* =7.5 Hz, 4H), 7.36 - 7.27 (m, 7H), 7.22 (t, *J* = 7.5 Hz, 2H), 3.88 (d, *J* = 13.7 Hz, 2H), 3.47 (d, *J* = 13.7 Hz, 2H), 3.12 (d, *J* = 10.3 Hz, 1H), 2.07 –1.93 (m, 1H), 1.05 (d, *J* = 6.5 Hz, 3H), 1.04 (d, *J* = 6.5 Hz, 3H) ppm; **^{13}C NMR** (125 MHz, Chloroform-*d*) δ 139.8, 131.8, 128.9, 128.3, 128.2, 127.8, 126.9, 123.7, 87.38, 86.0, 59.7 (2C), 55.1, 30.8, 20.9, 19.9 ppm; **HRMS** (ESI) *m/z* for $C_{26}H_{27}NNa$ ($[M+Na]^+$): 376.2036; Found: 376.2044; **Chiral HPLC** (Chiralpak OD-H, hexane/isopropanol = 99:1, 1 mL/min., λ = 254 nm), t_R (major) = 3.6 min., t_R (minor) = 3.8 min., 98% *ee*.

Supplementary Fig. 3. HPLC traces of 3a

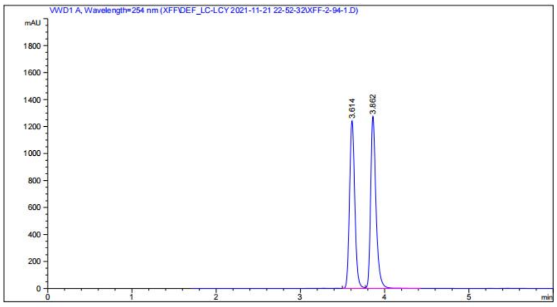

面积百分比报告

排序 : 信号
乘积因子: : 1.0000
稀释因子: : 1.0000
内标使用乘积因子和稀释因子

信号 1: VWD1 A, Wavelength=254 nm

峰 #	保留时间 [min]	类型	峰宽 [min]	峰面积 [mAU*s]	峰高 [mAU]	峰面积 %
1	3.614	VV	0.0664	5360.48291	1241.65137	48.8583
2	3.862	VV	0.0664	5611.00049	1274.53748	51.1417
总量 :				1.09715e4	2516.18884	

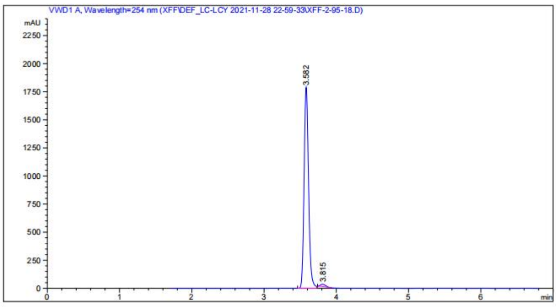

面积百分比报告

排序 : 信号
乘积因子: : 1.0000
稀释因子: : 1.0000
内标使用乘积因子和稀释因子

信号 1: VWD1 A, Wavelength=254 nm

峰 #	保留时间 [min]	类型	峰宽 [min]	峰面积 [mAU*s]	峰高 [mAU]	峰面积 %
1	3.582	VV	0.0670	7654.78662	1786.98938	98.7823
2	3.815	MM R	0.0781	94.36334	20.14789	1.2177
总量 :				7749.14996	1807.13727	

(*S*)-(–)-*N*,*N*-Dibenzyl-1,5-diphenylpent-1-yn-3-amine (3b)

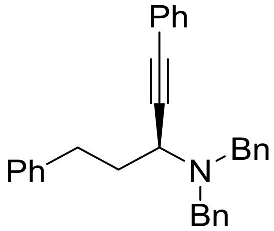

Following general procedure C, the reaction of amide **1b** (131.8 mg, 0.4 mmol) with alkyne **2a** (52 µL, 0.48 mmol) gave, after FC (eluent: EtOAc/*n*-hexane = 1: 20), propargylic amine **3b** (149.6 mg, 90% yield, 95% *ee*) as a colorless oil; $[\alpha]_D^{25}$ = –180.1 (*c* 1, $CHCl_3$); **IR** (film) $\tilde{v}$: 2959, 2838, 1602, 1455, 1259, 1205, 1152, 1067, 1027 cm^{-1}; **^{1}H NMR** (500 MHz, Chloroform-*d*) δ 7.51 – 7.49 (m, 2H), 7.42 (d, *J* = 5.0 Hz, 4H), 7.36 – 7.30 (m, 7H), 7.27 – 7.21 (m, 2H), 7.20 (t, *J* = 7.4 Hz, 2H), 7.13 (t, *J* = 7.3 Hz, 1H), 7.06 (d, *J* = 6.8 Hz, 2H), 3.92 (d, *J* = 13.7 Hz, 2H), 3.67 (t, *J* = 7.5 Hz, 1H), 3.52 (d, *J* = 13.7 Hz, 2H), 2.85 – 2.64 (m, 2H), 2.16 – 1.99 (m, 2H) ppm; **^{13}C NMR** (126 MHz, Chloroform-*d*) δ 141.9, 139.7, 131.8, 128.9, 128.4, 128.3 (3C), 127.9, 126.9, 125.7, 123.5, 87.6, 85.5, 55.1 (2C), 51.9, 35.7, 32.7 ppm; **HRMS** (ESI) *m/z* for $C_{31}H_{30}N$ ($[M+H]^+$): 403.2295, Found: 403.2295; **Chiral HPLC** (Chiralpak OD-H, hexane, 1 mL/min., λ = 254 nm), t_R (major) = 28.2 min., t_R (minor) = 36.2 min., 95% *ee*.

Supplementary Fig. 4. HPLC traces of 3b

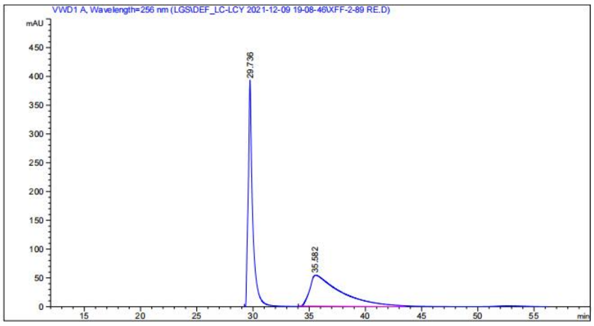

面积百分比报告

排序 : 信号
乘积因子: : 1.0000
稀释因子: : 1.0000
内标使用乘积因子和稀释因子

信号 1: VWD1 A, Wavelength=256 nm

峰 #	保留时间 [min]	类型	峰宽 [min]	峰面积 [mAU*s]	峰高 [mAU]	峰面积 %
1	29.736	MM R	0.4597	1.08446e4	393.18536	50.8744
2	35.582	MM R	3.2522	1.04719e4	53.66566	49.1256

总量 : 2.13165e4 446.85102

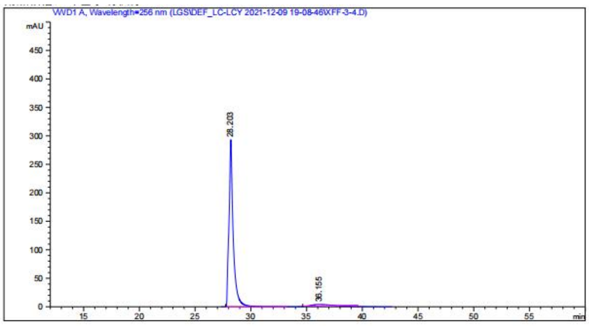

面积百分比报告

排序 : 信号
乘积因子: : 1.0000
稀释因子: : 1.0000
内标使用乘积因子和稀释因子

信号 1: VWD1 A, Wavelength=256 nm

峰 #	保留时间 [min]	类型	峰宽 [min]	峰面积 [mAU*s]	峰高 [mAU]	峰面积 %
1	28.203	BB	0.3540	7743.36719	293.34265	97.3974
2	36.155	MM R	1.4628	206.91769	2.35760	2.6026

总量 : 7950.28488 295.70025

(*S*)-(–)-*N*,*N*-Dibenzyl-1-phenylhept-6-en-1-yn-3-amine (3c)

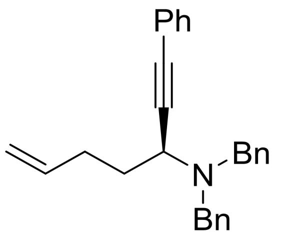

Following general procedure C, the reaction of amide **1c** (111.8 mg, 0.4 mmol) with alkyne **2a** (52 μL, 0.48 mmol) gave, after FC (eluent: EtOAc/*n*-hexane = 1: 200), propargylic amine **3c** (133 mg, 91% yield, 90% *ee*) as a colorless oil; $[\alpha]_D^{25}$ = –218.1 (*c* 1, $CHCl_3$); **IR** (film) $\tilde{v}$: 3076, 3042, 2932, 1897, 1639, 1598, 1489, 1453, 1320, 1070, 1027 cm^{-1}; **^{1}H NMR** (500 MHz, Chloroform-*d*) δ 7.53 – 7.47 (m, 2H), 7.41 (d, *J* = 7.5 Hz, 4H), 7.35 – 7.30 (m, 7H), 7.23 (t, *J* = 7.3 Hz, 2H), 5.76-5.67 (m, 1H), 4.96 – 4.84 (m, 2H), 3.89 (d, *J* = 13.8 Hz, 2H), 3.63 (t, *J* = 7.6 Hz, 1H), 3.49 (d, *J* = 13.8 Hz, 2H), 2.28-2.11 (m, 2H), 1.94 – 1.75 (m, 2H) ppm; **^{13}C NMR** (126 MHz, Chloroform-*d*) δ 139.8, 137.9, 131.8, 128.9, 128.3, 128.2, 127.9, 126.9, 123.5, 114.8, 87.7, 85.4, 55.1 (2C), 51.9, 33.1, 30.6 ppm; **HRMS** (ESI) *m/z* for $C_{27}H_{28}NO$ ($[M+H]^+$): 366.2216, Found: 366.2224; **Chiral HPLC** (Chiralpak OD-H, hexane/*i*-PrOH = 99/1, 1 mL/min., λ = 254 nm), t_R (major) =3.6 min., t_R (minor) = 3.9 min., 90% *ee*.

Supplementary Fig. 5. HPLC traces of 3c

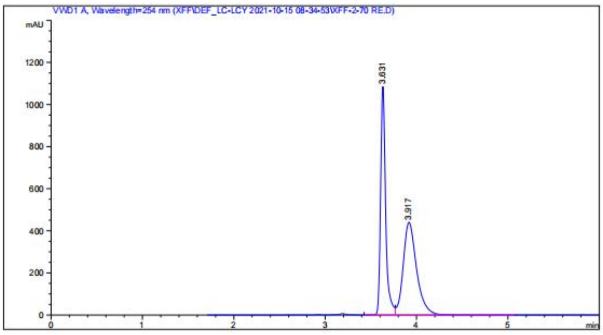

面积百分比报告

排序 : 信号
乘积因子: : 1.0000
稀释因子: : 1.0000
内标使用乘积因子和稀释因子

信号 1: VWD1 A, Wavelength=254 nm

峰 #	保留时间 [min]	类型	峰宽 [min]	峰面积 [mAU*s]	峰高 [mAU]	峰面积 %
1	3.631	BV	0.0575	4146.95850	1086.92554	48.0765
2	3.917	VB	0.1538	4478.79443	439.82983	51.9235
总量 :				8625.75293	1526.75537	

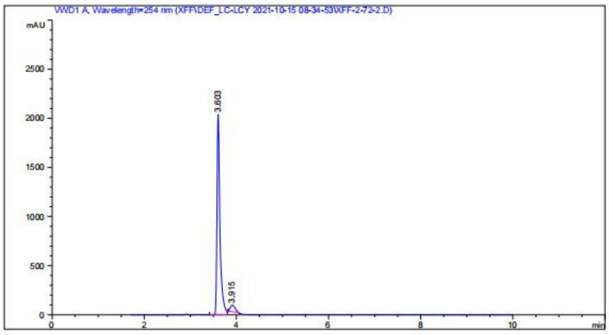

面积百分比报告

排序 : 信号
乘积因子: : 1.0000
稀释因子: : 1.0000
内标使用乘积因子和稀释因子

信号 1: VWD1 A, Wavelength=254 nm

峰 #	保留时间 [min]	类型	峰宽 [min]	峰面积 [mAU*s]	峰高 [mAU]	峰面积 %
1	3.603	BV	0.0652	9130.04883	2040.89819	95.0950
2	3.915	MM R	0.1196	470.93295	65.65057	4.9050
总量 :				9600.98178	2106.54877	

(*S*)-(–)-*N*,*N*-Dibenzyl-1-phenyloctadec-1-yn-3-amine (3d)

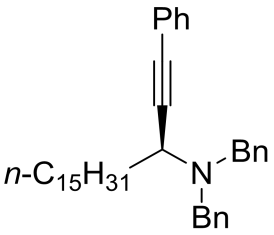

Following general procedure C, the reaction of amide **1d** (174.3 mg, 0.4 mmol) with alkyne **2a** (52 μL, 0.48 mmol) gave, after FC (eluent: EtOAc/*n*-hexane = 1: 10), propargylic amine **3d** (194 mg, yield: 94%) as a colorless oil; $[\alpha]_D^{25}$ = –136.8 (*c* 1, $CHCl_3$); **IR** (film) $\tilde{v}$: 2924, 2852, 1489, 1454, 1363, 1253, 1070, 1028 cm^{-1}; **^{1}H NMR** (500 MHz, Chloroform-*d*) δ 7.53 – 7.45 (m, 2H), 7.41 (d, *J* = 4.8 Hz, 4H), 7.35 – 7.28 (m, 7H), 7.22 (m, 2H), 3.89 (d, *J* = 13.8 Hz, 2H), 3.59 (t, *J* = 8.0 Hz, 1H), 3.48 (d, *J* = 13.8 Hz, 2H), 1.83 – 1.66 (m, 2H), 1.52 – 1.36 (m, 2H), 1.30 – 1.17 (m, 24H), 0.88 (t, *J* = 6.8 Hz, 3H) ppm; **^{13}C NMR** (126 MHz, Chloroform-*d*) δ 139.9, 131.8, 128.8, 128.3, 128.2, 127.8, 126.8, 123.6, 88.1, 85.1, 54.9 (2C), 52.1, 33.8, 31.9, 29.7 (4C) 29.6 (2C), 29.5 (2C), 29.4, 29.2, 26.4, 22.7, 14.1 ppm; **HRMS** (ESI) *m/z* for $C_{38}H_{52}N$ ($[M+H]^+$): 522.4094, Found: 522.4098; **Chiral HPLC** (Chiralpak OD-H, hexane, 1 mL/min., λ = 254 nm), t_R (major) = 10.0 min., t_R (minor) = 9.4 min., 90% *ee*.

Supplementary Fig. 6. HPLC traces of 3d

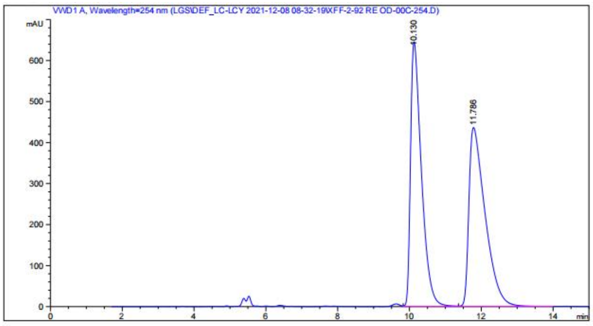

面积百分比报告

排序 : 信号
乘积因子: : 1.0000
稀释因子: : 1.0000
内标使用乘积因子和稀释因子

信号 1: VWD1 A, Wavelength=254 nm

峰 #	保留时间 [min]	类型	峰宽 [min]	峰面积 [mAU*s]	峰高 [mAU]	峰面积 %
1	10.130	VV	0.3150	1.35516e4	645.42310	49.7736
2	11.786	VB	0.4655	1.36749e4	436.51016	50.2264

总量 : 2.72265e4 1081.93326

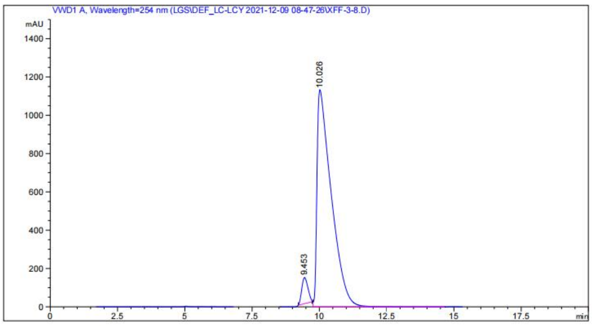

面积百分比报告

排序 : 信号
乘积因子: : 1.0000
稀释因子: : 1.0000
内标使用乘积因子和稀释因子

信号 1: VWD1 A, Wavelength=254 nm

峰 #	保留时间 [min]	类型	峰宽 [min]	峰面积 [mAU*s]	峰高 [mAU]	峰面积 %
1	9.453	MM R	0.2644	2154.02563	135.79593	4.9135
2	10.026	VV	0.5256	4.16845e4	1131.97791	95.0865

总量 : 4.38386e4 1267.77383

(*S*)-(–)-*N*,*N*-Dibenzyl-1-phenyloct-1-yn-3-amine (3e)

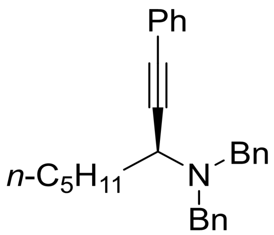

Following general procedure C, the reaction of amide **1e** (118.2 mg, 0.4 mmol) with alkyne **2a** (52 μL, 0.48 mmol) gave, after FC (eluent: EtOAc/*n*-hexane = 1: 30), propargylic amine **3e** (131.3 mg, yield: 86%, 90% *ee*) as a colorless oil; $[\alpha]_D^{25} = -135.5$ (*c* 1, $CHCl_3$); **IR** (film) $\tilde{v}$: 2931, 2857, 1946, 1874, 1599, 1489, 1453, 1363, 1258, 1070, 1027 cm^{-1}; **^{1}H NMR** (400 MHz, Chloroform-*d*) δ 7.53 – 7.47 (m, 2H), 7.41 (d, *J* = 7.5 Hz, 4H), 7.30 (d, *J* = 14.7 Hz, 7H), 7.24 – 7.18 (m, 2H), 3.88 (d, *J* = 13.7 Hz, 2H), 3.60 (t, *J* = 7.5 Hz, 1H), 3.48 (d, *J* = 13.7 Hz, 2H), 1.8–1.65 (m, 2H), 1.54–1.36 (m, 2H), 1.32 – 1.22 (m, 2H), 1.20 – 1.12 (m, 2H), 0.85 (t, *J* = 7.2 Hz, 3H) ppm; **^{13}C NMR** (100 MHz, Chloroform-*d*) δ 139.9, 131.8, 128.8, 128.2, 127.8, 126.8, 123.6, 88.1, 85.1, 54.9 (2C), 52.1, 33.7, 31.3, 26.0, 22.6, 14.0 ppm; **HRMS** (ESI) *m/z* for $C_{28}H_{32}N$ ($[M+H]^+$): 382.2529, Found: 382.2535; **Chiral HPLC** (Chiralpak OD-H, hexane, 1 mL/min., λ = 254 nm), t_R (major) = 10.9 min., t_R (minor) = 9.1 min., 90% *ee*.

Supplementary Fig. 7. HPLC traces of 3e

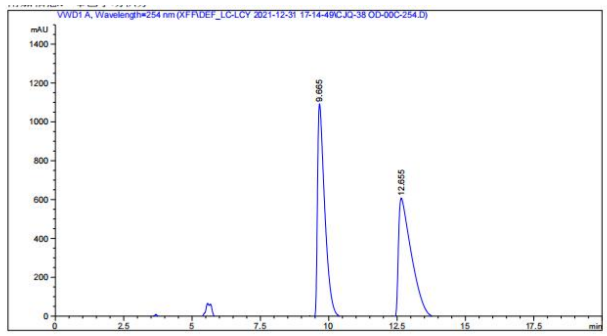

面积百分比报告

排序 : 信号
乘积因子: : 1.0000
稀释因子: : 1.0000
内标使用乘积因子和稀释因子

信号 1: VWD1 A, Wavelength=254 nm

峰 #	保留时间 [min]	类型	峰宽 [min]	峰面积 [mAU*s]	峰高 [mAU]	峰面积 %
1	9.665	BB	0.2759	2.05942e4	1102.65845	49.6886
2	12.655	BB	0.4843	2.08523e4	618.49371	50.3114
总量 :				4.14465e4	1721.15216	

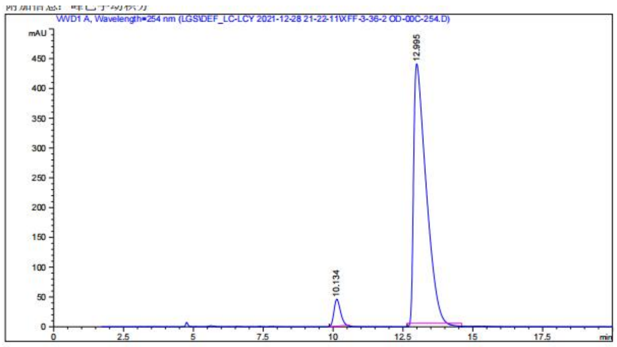

面积百分比报告

排序 : 信号
乘积因子: : 1.0000
稀释因子: : 1.0000
内标使用乘积因子和稀释因子

信号 1: VWD1 A, Wavelength=254 nm

峰 #	保留时间 [min]	类型	峰宽 [min]	峰面积 [mAU*s]	峰高 [mAU]	峰面积 %
1	10.134	MM R	0.2680	722.92999	44.95443	5.1053
2	12.995	MM R	0.5144	1.34373e4	435.39490	94.8947
总量 :				1.41603e4	480.34932	

(*S*)-(–)-*N*,*N*-Dibenzyl-1-phenylhex-1-yn-3-amine (3f)

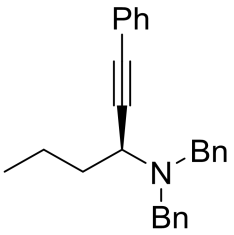

Following general procedure C, the reaction of amide **1f** (106.9 mg, 0.4 mmol) with alkyne **2a** (52 μL, 0.48 mmol) gave, after FC (eluent: EtOAc/*n*-hexane = 1: 200), propargylic amine **3f** (115 mg, yield: 81%, 90% *ee*) as a colorless oil; $[\alpha]_D^{25}$ = –243.7 (*c* 1, $CHCl_3$); **IR** (film) $\tilde{\nu}$: 2957, 2928, 2871, 1598, 1489, 1453, 1362, 1070, 1027 cm^{-1}; **^{1}H NMR** (400 MHz, Chloroform-*d*) δ 7.52 – 7.49 (m, 2H), 7.44 – 7.41 (m, 4H), 7.34 – 7.29 (m, 7H), 7.25 – 7.20 (m, 2H), 3.89 (d, *J* = 13.8 Hz, 2H), 3.62 (t, *J* = 7.2 Hz, 1H), 3.49 (d, *J* = 13.8 Hz, 2H), 1.87 – 1.60 (m, 2H), 1.55 – 1.47 (m, 2H), 0.83 (t, *J* = 7.3 Hz, 3H) ppm; **^{13}C NMR** (100 MHz, Chloroform-*d*) δ 139.9, 131.8, 128.8, 128.3, 128.2, 127.8, 126.8, 123.6, 88.1, 85.1, 54.9 (2C), 51.9, 36.0, 19.6, 13.7 ppm; **HRMS** (ESI) *m/z* for $C_{26}H_{28}N$ ($[M+H]^+$): 354.2216, Found: 354.2219; **Chiral HPLC** (Chiralpak OD-H, hexane, 1 mL/min., λ = 254 nm), t_R (major) = 14.3 min., t_R (minor) = 10.3 min., 90% *ee*.

Supplementary Fig. 8. HPLC traces of 3f

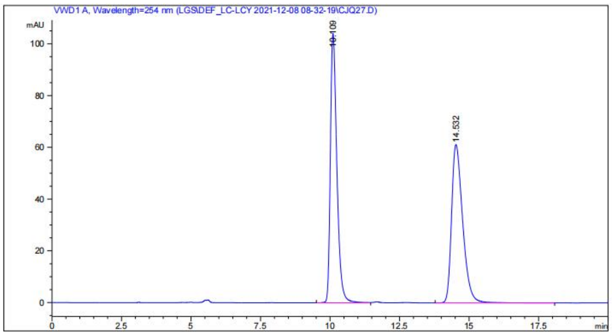

面积百分比报告

排序 : 信号
乘积因子: : 1.0000
稀释因子: : 1.0000
内标使用乘积因子和稀释因子

信号 1: VWD1 A, Wavelength=254 nm

峰 #	保留时间 [min]	类型	峰宽 [min]	峰面积 [mAU*s]	峰高 [mAU]	峰面积 %
1	10.109	BV	0.2475	1690.62732	104.09542	49.9447
2	14.532	BB	0.4249	1694.36853	61.21101	50.0553
总量 :				3384.99585	165.30643	

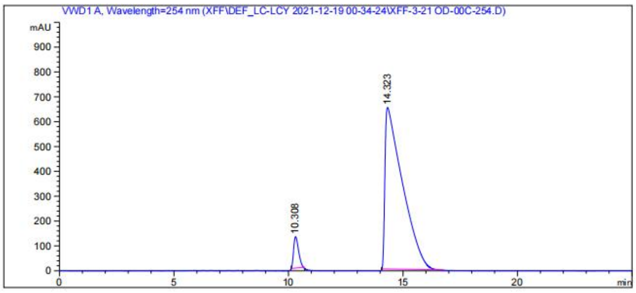

面积百分比报告

排序 : 信号
乘积因子: : 1.0000
稀释因子: : 1.0000
内标使用乘积因子和稀释因子

信号 1: VWD1 A, Wavelength=254 nm

峰 #	保留时间 [min]	类型	峰宽 [min]	峰面积 [mAU*s]	峰高 [mAU]	峰面积 %
1	10.308	MM R	0.2462	1871.10657	126.64461	5.1333
2	14.323	MM R	0.8859	3.45796e4	650.06226	94.8667
总量 :				3.64507e4	776.70686	

(*S*)-(–)-*N*,*N*-Dibenzyl-1-phenylpent-1-yn-3-amine (3g)

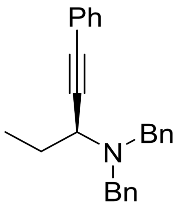

Following general procedure C, the reaction of amide **1g** (101.3 mg, 0.4 mmol) with alkyne **2a** (52 µL, 0.48 mmol) gave, after FC (eluent: EtOAc/*n*-hexane = 1: 200), propargylic amine **3g** (107 mg, yield: 79%, 91% *ee*) as a colorless oil; $[\alpha]_D^{25}$ = –264.8 (*c* 1, $CHCl_3$); **IR** (film) $\tilde{v}$: 2963, 2930, 2831, 1876, 1598, 1489, 1453, 1260, 1070, 1027 cm^{-1}; ^{1}**H NMR** (400 MHz, Chloroform-*d*) δ 7.51 – 7.48 (m, 2H), 7.42 (d, *J* = 7.5 Hz, 4H), 7.32–7.29 (m, 7H), 7.22 (t, *J* = 8.0 Hz, 2H), 3.88 (d, *J* = 13.8 Hz, 2H), 3.59 – 3.40 (m, 3H), 1.93 – 1.67 (m, 2H), 0.99 (t, *J* = 7.3 Hz, 3H) ppm; ^{13}**C NMR** (125 MHz, Chloroform-*d*) *δ* 139.9, 131.8, 128.8, 128.3, 128.2, 127.8, 126.8, 87.9, 85.3, 54.9 (2C), 54.1, 27.0, 11.3 ppm; **HRMS** (ESI) *m/z* for $C_{25}H_{26}N$ ($[M+H]^+$): 340.2060, Found: 340.2067; **Chiral HPLC** (Chiralpak OD-H, hexane/*i*-PrOH = 99/1, 1 mL/min., λ = 254 nm), t_R (major) =3.7 min., t_R (minor) = 3.9 min., 91% *ee*.

Supplementary Fig. 9. HPLC traces of 3g

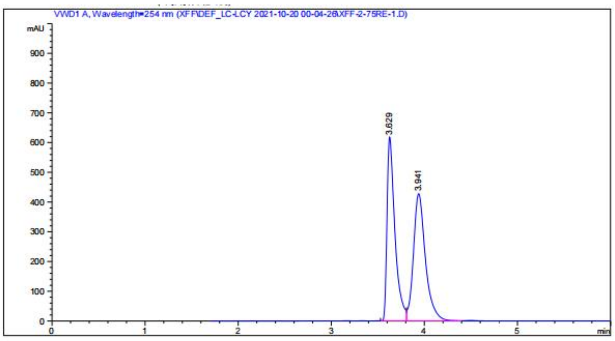

面积百分比报告

排序 : 信号
乘积因子: : 1.0000
稀释因子: : 1.0000
内标使用乘积因子和稀释因子

信号 1: VWD1 A, Wavelength=254 nm

峰 #	保留时间 [min]	类型	峰宽 [min]	峰面积 [mAU*s]	峰高 [mAU]	峰面积 %
1	3.629	BV	0.0876	3632.69312	617.80939	48.6438
2	3.941	VV	0.1346	3835.25293	427.66766	51.3562
总量 :				7467.94604	1045.47705	

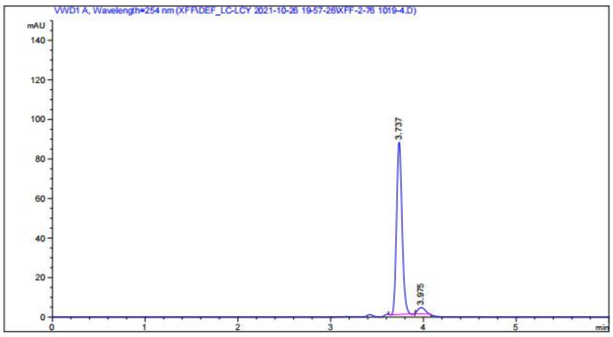

面积百分比报告

排序 : 信号
乘积因子: : 1.0000
稀释因子: : 1.0000
内标使用乘积因子和稀释因子

信号 1: VWD1 A, Wavelength=254 nm

峰 #	保留时间 [min]	类型	峰宽 [min]	峰面积 [mAU*s]	峰高 [mAU]	峰面积 %
1	3.737	MM R	0.0687	359.79578	87.23345	95.5404
2	3.975	MM R	0.0890	16.79443	3.14667	4.4596
总量 :				376.59021	90.38012	

(*S*)-(–)-*N*-Allyl-*N*-benzyl-4-methyl-1-phenylpent-1-yn-3-amine (3h)

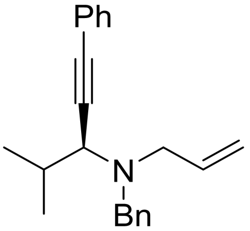

Following general procedure C, the reaction of amide **1h** (86.8 mg, 0.4 mmol) with alkyne **2a** (52 μL, 0.48 mmol) gave, after FC (eluent: EtOAc/*n*-hexane = 1: 200), propargylic amine **3h** (98.3 mg, 81% yield, 93% *ee*) as a colorless oil; $[\alpha]_D^{25}$ = –179.52 (*c* 1, $CHCl_3$); **IR** (film) $\tilde{v}$: 3080, 3029, 2957, 2924, 2811, 1874, 1598, 1488, 1466, 1365, 1266, 1070, 1028, 918, 755, 690 cm^{-1}; **^{1}H NMR** (500 MHz, Chloroform-*d*) δ 7.47 (d, *J* = 5.3 Hz, 2H), 7.39 (d, *J* = 7.5 Hz, 2H), 7.31 – 7.28 (m, 5H), 7.22 (t, *J* = 6.8 Hz, 1H), 5.91-5.83 (m, 1H), 5.26 (d, *J* = 17.2 Hz, 1H), 5.12 (d, *J* = 10.2 Hz, 1H), 3.92 (d, *J* = 14.0 Hz, 1H), 3.43 (d, *J* = 14.0 Hz, 1H), 3.29 (d, *J* = 10.2 Hz, 1H), 3.18 (d, *J* = 10.3 Hz, 1H), 3.02 – 2.97 (m, 1H), 1.97 – 1.90 (m, 1H), 1.05 (d, *J* = 6.5 Hz, 3H), 1.02 (d, *J* = 6.6 Hz, 3H) ppm; **^{13}C NMR** (126 MHz, Chloroform-*d*) δ 140.0, 136.9, 131.8, 128.8, 128.2, 128.2, 127.8, 126.7, 123.7, 116.9, 87.7, 85.7, 59.9, 55.1, 53.9, 31.1, 20.9, 19.9 ppm; **HRMS** (ESI) *m/z* for $C_{22}H_{26}N$ ($[M+H]^+$): 304.2060, Found: 304.2067; **Chiral HPLC** (Chiralpak OD-H, hexane, 1 mL/min., λ = 254 nm), t_R (major) =6.2 min., t_R (minor) = 6.8 min., 93% *ee*.

Supplementary Fig. 10. HPLC traces of 3h

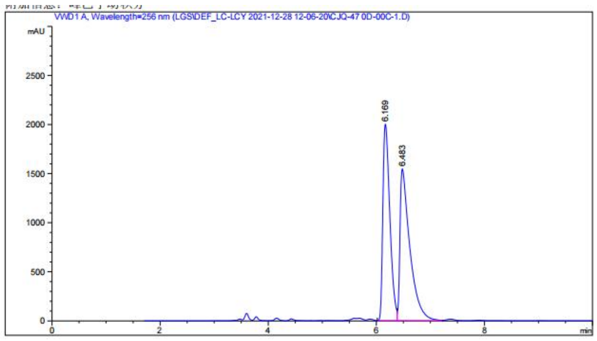

面积百分比报告

排序 : 信号
乘积因子: : 1.0000
稀释因子: : 1.0000
内标使用乘积因子和稀释因子

信号 1: VWD1 A, Wavelength=256 nm

峰 #	保留时间 [min]	类型	峰宽 [min]	峰面积 [mAU*s]	峰高 [mAU]	峰面积 %
1	6.169	BV	0.1384	1.79011e4	2000.78540	47.6213
2	6.483	VV	0.1817	1.96895e4	1545.29504	52.3787

总量 : 3.75906e4 3546.08044

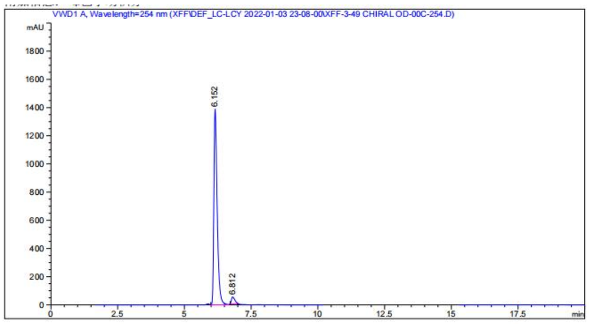

面积百分比报告

排序 : 信号
乘积因子: : 1.0000
稀释因子: : 1.0000
内标使用乘积因子和稀释因子

信号 1: VWD1 A, Wavelength=254 nm

峰 #	保留时间 [min]	类型	峰宽 [min]	峰面积 [mAU*s]	峰高 [mAU]	峰面积 %
1	6.152	VV	0.1313	1.20514e4	1387.53271	96.5421
2	6.812	MM R	0.1471	431.65152	48.89597	3.4579

总量 : 1.24830e4 1436.42868

(*S*)-(–)-*N*-(4-Methoxybenzyl)-*N*,4-dimethyl-1-phenylpent-1-yn-3-amine (3i)

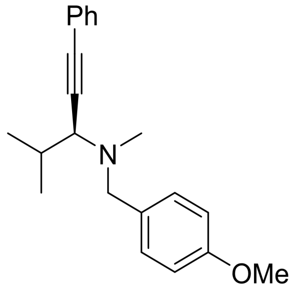

Following general procedure C, the reaction of amide **1i** (88.5 mg, 0.4 mmol) with alkyne **2a** (52 μL, 0.48 mmol) gave, after FC (eluent: EtOAc/*n*-hexane = 1: 20), propargylic amine **3i** (109.4 mg, 89% yield, 90% *ee*) as a white solid; $[\alpha]_D^{25}$ = –164.5 (*c* 1, $CHCl_3$); **IR** (film) $\tilde{\nu}$: 2956, 1612, 1511, 1243, 1069, 1036, 756, 691 cm^{-1}; **^{1}H NMR** (400 MHz, Chloroform-*d*) δ 7.51 – 7.43 (m, 2H), 7.35 – 7.26 (m, 5H), 6.85 (d, J = 8.4 Hz, 2H), 3.79 (s, 3H), 3.66 (d, J = 13.1 Hz, 1H), 3.48 (d, J = 13.1 Hz, 1H), 3.08 (d, J = 10.2 Hz, 1H), 2.23 (s, 3H), 1.96 – 1.87 (m, 1H), 1.09 (d, J = 6.6 Hz, 3H) 1.04 (d, J = 6.6 Hz, 3H) ppm; **^{13}C NMR** (100 MHz, Chloroform-*d*) δ 158.6, 131.7, 129.9, 128.2, 127.7, 123.7, 113.5, 87.1, 86.3, 63.0, 58.89, 55.2, 37.6, 30.9, 20.9, 19.7 ppm; **HRMS** (ESI) *m/z* for $C_{21}H_{26}NO$ ($[M+H]^+$): 308.2009, Found: 308.2009. **Chiral HPLC** (Chiralpak OJ-H, hexane/*i*-PrOH = 99/1, 1 mL/min., λ = 254 nm), t_R (major) =7.6 min., t_R (minor) = 6.9 min., 90% *ee*.

Supplementary Fig. 11. HPLC traces of 3i

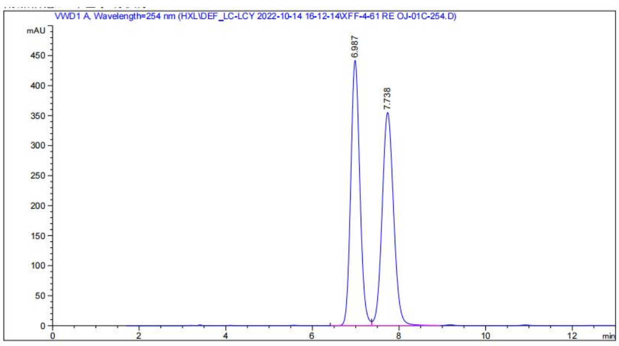

面积百分比报告

排序 : 信号
乘积因子: : 1.0000
稀释因子: : 1.0000
内标使用乘积因子和稀释因子

信号 1: VWD1 A, Wavelength=254 nm

峰 #	保留时间 [min]	类型	峰宽 [min]	峰面积 [mAU*s]	峰高 [mAU]	峰面积 %
1	6.987	BV	0.2254	6415.72949	441.77902	49.5690
2	7.738	VV	0.2865	6527.29053	355.05237	50.4310
总量 :				1.29430e4	796.83139	

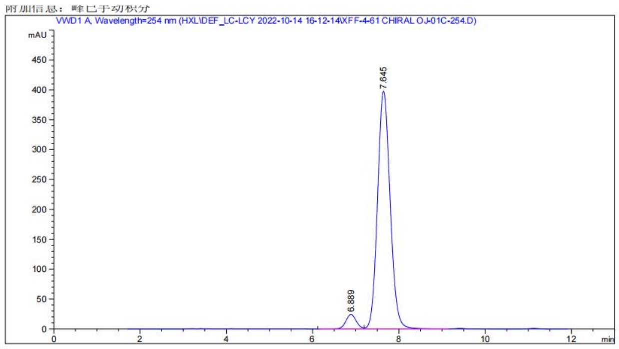

面积百分比报告

排序 : 信号
乘积因子: : 1.0000
稀释因子: : 1.0000
内标使用乘积因子和稀释因子

信号 1: VWD1 A, Wavelength=254 nm

峰 #	保留时间 [min]	类型	峰宽 [min]	峰面积 [mAU*s]	峰高 [mAU]	峰面积 %
1	6.889	BV	0.2628	410.00665	24.30481	4.8422
2	7.645	VV	0.3144	8057.27637	397.62158	95.1578
总量 :				8467.28302	421.92639	

(*S*)-(–)-1-Benzyl-2-((4-bromophenyl)ethynyl)piperidine (3j)

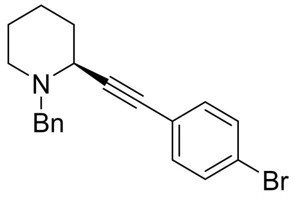

Following general procedure C, the reaction of amide **1j** (75.6 mg, 0.4 mmol) with alkyne **2b** (58 μL, 0.48 mmol) gave, after FC (eluent: EtOAc/*n*-hexane = 1: 100), propargylic amine **3j** (94.6 mg, 67% yield, 52% *ee*) as a colorless oil; $[\alpha]_D^{25}$ = –43.6 (*c* 1, $CHCl_3$); **IR** (film) $\tilde{v}$: 2922, 2854, 1451, 1073 cm^{-1}; **^{1}H NMR** (400 MHz, Chloroform-*d*) δ 7.45 (d, *J* = 8.3 Hz, 2H), 7.38 (d, *J* = 6.9 Hz, 2H), 7.35 – 7.28 (m, 4H), 7.25 (t, *J* = 6.0 Hz, 1H), 3.75 – 3.58 (m, 3H), 2.65 – 2.46 (m, 2H), 1.84 – 1.80 (m, 2H), 1.75 – 1.64 (m, 1H), 1.63 – 1.51 (m, 3H) ppm; **^{13}C NMR** (100 MHz, Chloroform-*d*) δ 138.5, 133.2, 131.5, 129.2, 128.2, 126.9, 122.5, 121.9, 88.8, 85.6, 60.6, 51.7, 49.3, 31.3, 25.7, 20.8 ppm; **HRMS** (ESI) *m/z* for $C_{20}H_{21}BrN$ ($[M+H]^+$): 354.0852, 356.0831, Found: 354.0857, 356.0840; **Chiral HPLC** (Chiralpak OJ-H, hexane/*i*-PrOH = 99/1, 1 mL/min., λ = 254 nm), t_R (major) =6.8 min., t_R (minor) = 8.2 min., 52% *ee*.

Supplementary Fig. 12. HPLC traces of 3j

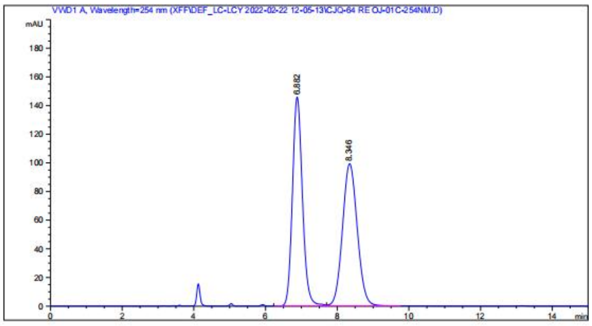

面积百分比报告

排序 : 信号
乘积因子: : 1.0000
稀释因子: : 1.0000
内标使用乘积因子和稀释因子

信号 1: VWD1 A, Wavelength=254 nm

峰 #	保留时间 [min]	类型	峰宽 [min]	峰面积 [mAU*s]	峰高 [mAU]	峰面积 %
1	6.882	BV	0.2980	2823.15210	145.76205	49.8410
2	8.346	VB	0.4450	2841.16675	99.24428	50.1590
总量 :				5664.31885	245.00633	

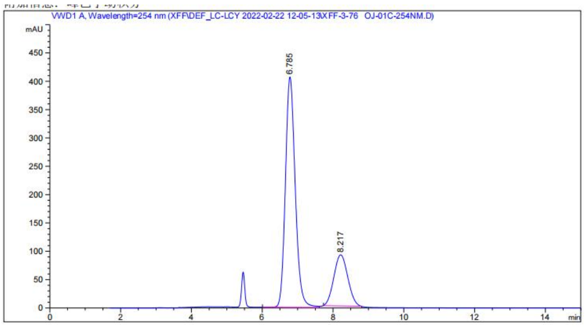

面积百分比报告

排序 : 信号
乘积因子: : 1.0000
稀释因子: : 1.0000
内标使用乘积因子和稀释因子

信号 1: VWD1 A, Wavelength=254 nm

峰 #	保留时间 [min]	类型	峰宽 [min]	峰面积 [mAU*s]	峰高 [mAU]	峰面积 %
1	6.785	BV	0.2964	7819.38037	406.47629	76.0397
2	8.217	MM R	0.4552	2463.91235	90.21661	23.9603
总量 :				1.02833e4	496.69290	

(*S*)-(–)-1-Benzyl-2-((4-bromophenyl)ethynyl)azepane (3k)

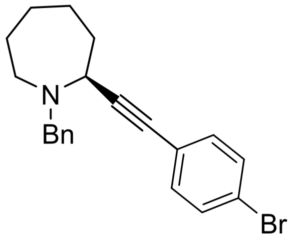

Following general procedure C, the reaction of amide **1k** (81.3 mg, 0.4 mmol) with alkyne **2b** (58 μL, 0.48 mmol) gave, after FC (eluent: EtOAc/*n*-hexane = 1: 100), propargylic amine **3k** (99.8 mg, 68% yield, 97% *ee*) as a colorless oil; $[\alpha]_D^{25}$ = –67.8 (*c* 1, $CHCl_3$); **IR** (film) $\tilde{v}$: 2922, 2851, 1484, 1453, 1180, 1070 cm^{-1}; **^{1}H NMR** (500 MHz, Chloroform-*d*) δ 7.51 – 7.36 (m, 4H), 7.33 – 7.30 (m, 4H), 7.26 – 7.22 (m, 1H), 3.83 – 3.73 (m, 3H), 2.85 – 2.80 (m, 1H), 2.63 – 2.58 (m, 1H), 2.06 – 2.00 (m, 1H), 1.85 – 1.69 (m, 4H), 1.65 – 1.61 (m, 2H), 1.59 – 1.49 (m, 1H) ppm; **^{13}C NMR** (100 MHz, Chloroform-*d*) δ 139.9, 133.2, 131.4, 128.8, 128.2, 126.9, 122.6, 121.8, 90.5, 84.2, 60.8, 54.1, 50.5, 34.8, 28.8, 27.6, 23.8 ppm; **HRMS** (ESI) *m/z* for $C_{21}H_{23}BrN$ ($[M+H]^+$): 368.1008, 370.0988; Found: 368.1013, 370.0995; **Chiral HPLC** (Chiralpak OD-H, hexane/*i*-PrOH = 99/1, 1 mL/min., λ = 254 nm), t_R (major) =3.9 min., t_R (minor) = 3.6 min., 97% *ee*.

Supplementary Fig. 13. HPLC traces of 3k

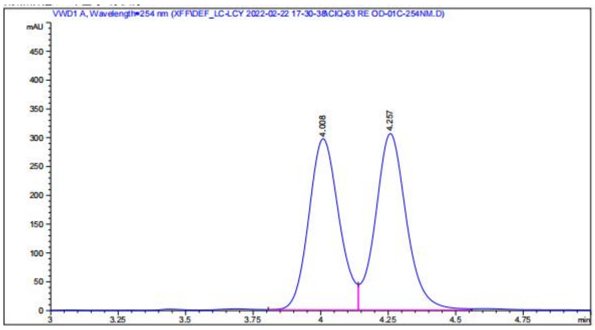

面积百分比报告

排序 : 信号
乘积因子: : 1.0000
稀释因子: : 1.0000
内标使用乘积因子和稀释因子

信号 1: VWD1 A, Wavelength=254 nm

峰 #	保留时间 [min]	类型	峰宽 [min]	峰面积 [mAU*s]	峰高 [mAU]	峰面积 %
1	4.008	BV	0.1194	2299.87720	296.77475	48.6678
2	4.257	VV	0.1205	2425.79053	305.91589	51.3322

总量 : 4725.66772 602.69064

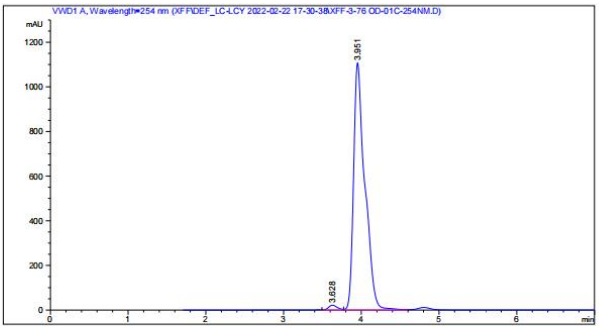

面积百分比报告

排序 : 信号
乘积因子: : 1.0000
稀释因子: : 1.0000
内标使用乘积因子和稀释因子

信号 1: VWD1 A, Wavelength=254 nm

峰 #	保留时间 [min]	类型	峰宽 [min]	峰面积 [mAU*s]	峰高 [mAU]	峰面积 %
1	3.628	VV	0.1186	166.89735	21.50172	1.4716
2	3.951	VV	0.1437	1.11741e4	1107.32312	98.5284

总量 : 1.13410e4 1128.82484

(*S*)-(–)-*N*,*N*-Dibenzyl-4-methyl-1-(*p*-tolyl)pent-1-yn-3-amine (3l)

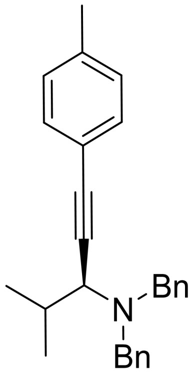

Following general procedure C, the reaction of amide **1a** (106.9 mg, 0.4 mmol) with alkyne **2c** (62 μL, 0.48 mmol) gave, after FC (eluent: EtOAc/*n*-hexane = 1: 200), propargylic amine **3l** (124.9 mg, 85% yield, 94% *ee*) as a colorless oil; $[\alpha]_D^{25}$ = –300.4 (*c* 1, $CHCl_3$); **IR** (film) $\tilde{v}$: 2957, 2922, 2868, 1494, 1453, 1267, 1069 cm^{-1}; **^{1}H NMR** (400 MHz, Chloroform-*d*) δ 7.42 (t, *J* = 8.1 Hz, 6H), 7.31 (t, *J* = 7.4 Hz, 4H), 7.23 (d, *J* = 7.4 Hz, 2H), 7.14 (d, *J* = 7.8 Hz, 2H), 3.87 (d, *J* = 13.8 Hz, 2H), 3.46 (d, *J* = 13.7 Hz, 2H), 3.10 (d, *J* = 10.3 Hz, 1H), 2.36 (s, 3H), 2.06 – 1.93 (m, 1H), 1.04 (d, *J* = 6.5 Hz, 3H), 1.03 (d, *J* = 6.6 Hz, 3H) ppm; **^{13}C NMR** (100 MHz, Chloroform-*d*) δ 139.8, 137.8, 131.7, 129.0, 128.9, 128.2, 126.8, 120.6, 86.5, 86.0, 59.7, 55.1 (2C), 30.8, 21.4, 20.9, 19.9 ppm; **HRMS** (ESI) *m/z* for $C_{27}H_{30}N$ ($[M+H]^+$): 368.2373; Found: 368.2380; **Chiral HPLC** (Chiralpak OD-H, hexane, 1 mL/min., λ = 254 nm), t_R (major) = 8.9 min., t_R (minor) = 8.6 min., 94% *ee*.

Supplementary Fig. 14. HPLC traces of 3l

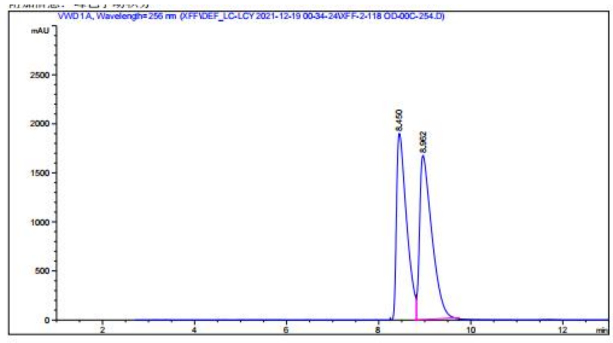

面积百分比报告

排序 : 信号
乘积因子: : 1.0000
稀释因子: : 1.0000
内标使用乘积因子和稀释因子

信号 1: VWD1 A, Wavelength=256 nm

峰 #	保留时间 [min]	类型	峰宽 [min]	峰面积 [mAU*s]	峰高 [mAU]	峰面积 %
1	8.450	MF R	0.2554	2.92886e4	1911.21741	48.7005
2	8.962	FM R	0.3074	3.08516e4	1672.93091	51.2995

总量 : 6.01402e4 3584.14832

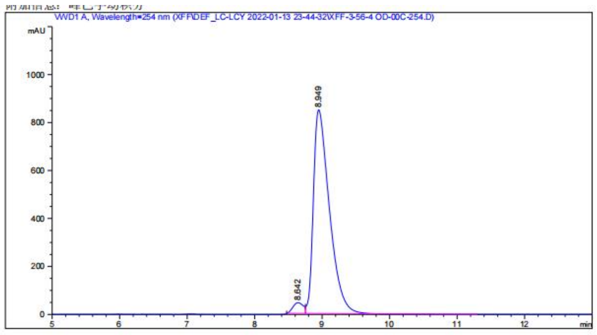

面积百分比报告

排序 : 信号
乘积因子: : 1.0000
稀释因子: : 1.0000
内标使用乘积因子和稀释因子

信号 1: VWD1 A, Wavelength=254 nm

峰 #	保留时间 [min]	类型	峰宽 [min]	峰面积 [mAU*s]	峰高 [mAU]	峰面积 %
1	8.642	MF R	0.1770	470.34027	44.28419	3.2563
2	8.949	FM R	0.2743	1.39738e4	849.10718	96.7437

总量 : 1.44441e4 893.39137

(*S*)-(–)-*N*,*N*-Dibenzyl-4-methyl-1-(trimethylsilyl)pent-1-yn-3-amine (3m)

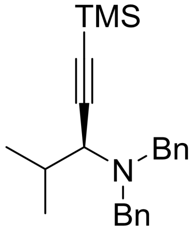

Following general procedure C, the reaction of amide **1a** (106.9 mg, 0.4 mmol) with alkyne **2d** (68 μL, 0.48 mmol) gave, after FC (eluent: EtOAc/*n*-hexane = 1: 200), propargylic amine **3m** (104.9 mg, 75% yield, 98% *ee*) as a white solid; M. p. 47 – 48 °C; $[\alpha]_D^{25}$ = –251.6 (*c* 1, $CHCl_3$) {lit.: $[\alpha]_D^{20}$ = –222.9 (*c* 1, $CHCl_3$), 98% *ee*}[10b]; **IR** (film) $\tilde{v}$: 2958, 2832, 2808, 2157, 1603, 1494, 1453, 1249, 1018, 926, 841, 746, 698, 638 cm^{-1}; **^{1}H NMR** (400 MHz, Chloroform-*d*) δ 7.39 (d, *J* = 7.5 Hz, 4H), 7.30 (t, *J* = 7.4 Hz, 4H), 7.2 (t, *J* = 6.0 Hz, 2H), 3.79 (d, *J* = 13.7 Hz, 2H), 3.34 (d, *J* = 13.7 Hz, 2H), 2.89 (d, *J* = 10.3 Hz, 1H), 1.92 – 1.83 (m, 1H), 0.98 (d, *J* = 6.5 Hz, 3H), 0.96 (d, *J* = 6.6 Hz, 3H), 0.24 (s, 9H) ppm; **^{13}C NMR** (100 MHz, Chloroform-*d*) δ 139.8, 128.9, 128.2, 126.8, 103.8, 89.9, 59.9, 54.9 (2C), 30.5, 20.8, 19.8, 0.41 (3C) ppm; **HRMS** (ESI) *m/z* for $C_{23}H_{32}NSi$ ($[M+H]^+$): 350.2299; Found: 350.2305; **Chiral HPLC** (Chiralpak OD-H, hexane, 1 mL/min., λ = 254 nm), t_R (major) = 3.9 min., t_R (minor) = 4.1 min., 98% *ee*.

Supplementary Fig. 15. HPLC traces of 3m

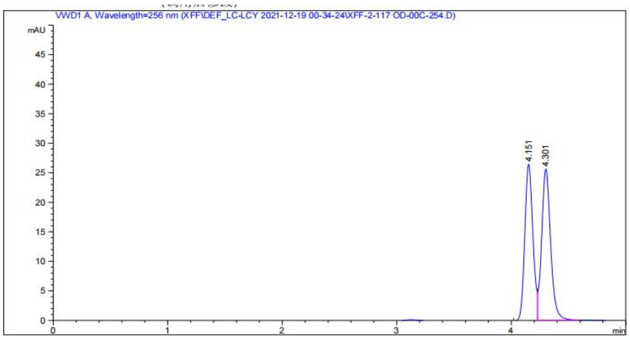

面积百分比报告

排序 : 信号
乘积因子: : 1.0000
稀释因子: : 1.0000
内标使用乘积因子和稀释因子

信号 1: VWD1 A, Wavelength=256 nm

峰 #	保留时间 [min]	类型	峰宽 [min]	峰面积 [mAU*s]	峰高 [mAU]	峰面积 %
1	4.151	BV	0.0761	129.59013	26.45706	48.0925
2	4.301	VB	0.0837	139.87030	25.63550	51.9075
总量 :				269.46043	52.09257	

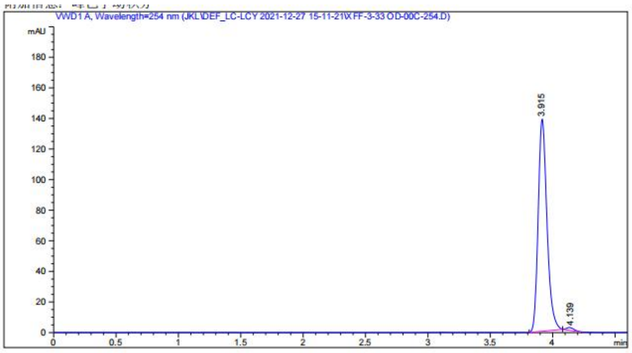

面积百分比报告

排序 : 信号
乘积因子: : 1.0000
稀释因子: : 1.0000
内标使用乘积因子和稀释因子

信号 1: VWD1 A, Wavelength=254 nm

峰 #	保留时间 [min]	类型	峰宽 [min]	峰面积 [mAU*s]	峰高 [mAU]	峰面积 %
1	3.915	MM R	0.0817	680.00598	138.76653	98.8800
2	4.139	MM R	0.0705	7.70262	1.81996	1.1200
总量 :				687.70860	140.58649	

(*S*)-(–)-*N*,*N*-Dibenzyl-1-cyclopropyl-4-methylpent-1-yn-3-amine (3n)

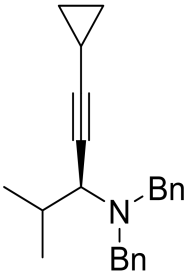

Following general procedure C, the reaction of amide **1a** (106.9 mg, 0.4 mmol) with alkyne **2e** (42 μL, 0.48 mmol) gave, after FC (eluent: EtOAc/*n*-hexane = 1: 200), propargylic amine **3n** (92.7 mg, 73% yield, 94% *ee*) as a colorless oil; $[\alpha]_D^{25}$ = –243.1 (*c* 1, $CHCl_3$); **IR** (film) $\tilde{v}$: 2956, 2868, 2807, 2222, 1602, 1494, 1453, 1267, 1069, 1028 cm^{-1}; **^{1}H NMR** (400 MHz, Chloroform-*d*) δ 7.46 (d, *J* = 7.5 Hz, 4H), 7.37 (d, *J* = 7.4 Hz, 4H), 7.28 (t, *J* = 7.5 Hz, 2H), 3.84 (d, *J* = 13.7 Hz, 2H), 3.40 (d, *J* = 13.7 Hz, 2H), 2.89 (d, *J* = 10.4 Hz, 1H), 1.95-1.86 (m, 1H), 1.43-1.37 (m, 1H), 1.04 (d, *J* = 6.5 Hz, 3H), 1.00 (d, *J* = 6.6 Hz, 3H), 0.90 – 0.86 (m, 2H), 0.79 – 0.75 (m, 2H) ppm; **^{13}C NMR** (100 MHz, Chloroform-*d*) δ 140.0, 128.9, 128.1, 126.7, 89.1, 72.3, 59.2, 54.9 (2C), 30.9, 20.9, 19.9, 8.6 (2C), 0.43 ppm; **HRMS** (ESI) *m/z* for $C_{23}H_{28}N$ ($[M+H]^+$): 318.2227; Found: 318.2226; **Chiral HPLC** (Chiralpak OD-H, hexane, 1 mL/min., λ = 254 nm), t_R (major) = 4.8 min., t_R (minor) = 4.2 min., 94% *ee*.

Supplementary Fig. 16. HPLC traces of 3n

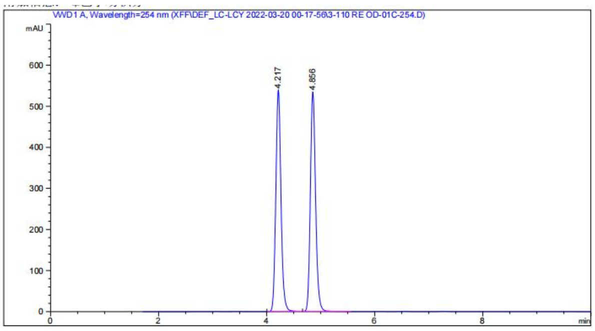

面积百分比报告

排序 : 信号
乘积因子: : 1.0000
稀释因子: : 1.0000
内标使用乘积因子和稀释因子

信号 1: VWD1 A, Wavelength=254 nm

峰 #	保留时间 [min]	类型	峰宽 [min]	峰面积 [mAU*s]	峰高 [mAU]	峰面积 %
1	4.217	BB	0.1035	3576.38452	538.16998	49.9069
2	4.856	BB	0.1045	3589.73340	533.43115	50.0931
总量 :				7166.11792	1071.60114	

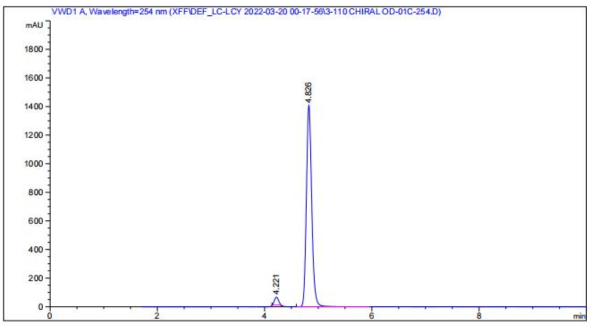

面积百分比报告

排序 : 信号
乘积因子: : 1.0000
稀释因子: : 1.0000
内标使用乘积因子和稀释因子

信号 1: VWD1 A, Wavelength=254 nm

峰 #	保留时间 [min]	类型	峰宽 [min]	峰面积 [mAU*s]	峰高 [mAU]	峰面积 %
1	4.221	MM R	0.0878	291.92307	55.39262	2.9113
2	4.826	BV	0.1068	9735.15918	1406.11536	97.0887
总量 :				1.00271e4	1461.50798	

(*S*)-(–)-*N*,*N*-Dibenzyl-2-methylnon-4-yn-3-amine (3o)

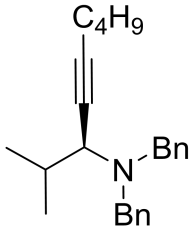

Following general procedure C, the reaction of amide **1a** (106.9 mg, 0.4 mmol) with alkyne **2f** (54 µL, 0.48 mmol) gave, after FC (eluent: EtOAc/*n*-hexane = 1: 100), propargylic amine **3o** (110.7 mg, 83% yield, 93% *ee*) as a colorless oil; $[\alpha]_D^{25}$ = –391.3 (*c* 1, $CHCl_3$); **IR** (film) $\tilde{v}$: 2956, 2931, 1603, 1494, 1453, 1363, 1286, 1069 cm^{-1}; **^{1}H NMR** (500 MHz, Chloroform-*d*) δ 7.40 (d, *J* = 7.5 Hz, 4H), 7.29 (t, *J* = 7.5 Hz, 4H), 7.21 (t, *J* = 7.4 Hz, 2H), 3.78 (d, *J* = 13.8 Hz, 2H), 3.35 (d, *J* = 13.7 Hz, 2H), 2.85 (d, *J* = 8.0 Hz, 1H), 2.30 (t, *J* = 6.1 Hz, 2H), 1.89 – 1.82 (m, 1H), 1.61 – 1.46 (m, 4H), 0.99 – 0.94 (m, 9H) ppm; **^{13}C NMR** (126 MHz, Chloroform-*d*) δ 140.1, 128.9, 128.1, 126.7, 85.7, 77.1, 59.2, 54.9 (2C), 31.4, 30.9, 21.9, 20.9, 19.9, 18.4, 13.6 ppm; **HRMS** (ESI) *m/z* for $C_{24}H_{32}N$ ($[M+H]^+$): 334.2529; Found: 334.2533; **Chiral HPLC** (Chiralpak OD-H, hexane/*i*-PrOH = 99/1, 1 mL/min., λ = 254 nm), t_R (major) = 6.5 min., t_R (minor) = 6.8 min., 93% *ee*.

Supplementary Fig. 17. HPLC traces of 3o

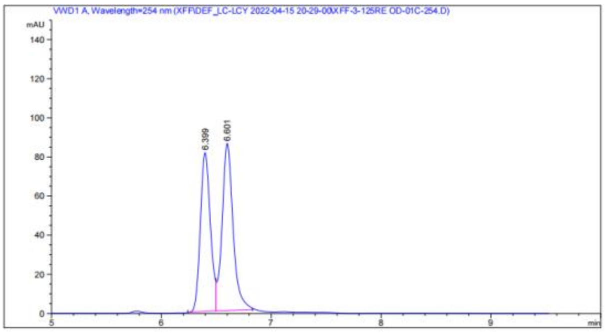

面积百分比报告

排序 : 信号
乘积因子: : 1.0000
稀释因子: : 1.0000
内标使用乘积因子和稀释因子

信号 1: VWD1 A, Wavelength=254 nm

峰 #	保留时间 [min]	类型	峰宽 [min]	峰面积 [mAU*s]	峰高 [mAU]	峰面积 %
1	6.399	MF R	0.1052	511.96115	81.14389	46.1817
2	6.601	FM R	0.1163	596.61871	85.47660	53.8183

总量 : 1108.57986 166.62049

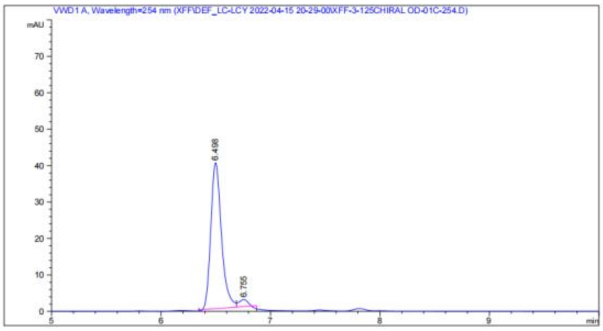

面积百分比报告

排序 : 信号
乘积因子: : 1.0000
稀释因子: : 1.0000
内标使用乘积因子和稀释因子

信号 1: VWD1 A, Wavelength=254 nm

峰 #	保留时间 [min]	类型	峰宽 [min]	峰面积 [mAU*s]	峰高 [mAU]	峰面积 %
1	6.498	MF R	0.1123	270.09839	40.08462	96.2756
2	6.755	FM R	0.0921	10.44869	1.89160	3.7244

总量 : 280.54708 41.97622

(–)-Methyl (*S*)-4-(benzyl(methyl)amino)-6-phenylhex-5-ynoate (3p)

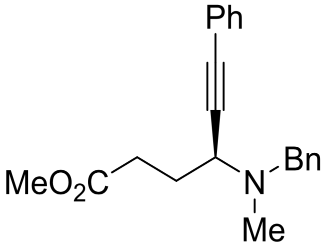

Following general procedure C, the reaction of amide **1l** (94.1 mg, 0.4 mmol) with alkyne **2a** (52 μL, 0.48 mmol) gave, after FC (eluent: EtOAc/*n*-hexane = 1: 100), propargylic amine **3p** (92.6 mg, 72% yield, 92% *ee*) as a colorless oil; $[\alpha]_D^{25}$ = –129.92 (*c* 1, $CHCl_3$); **IR** (film) $\tilde{v}$: 3358, 3028, 2929, 2850, 1676, 1597, 1488, 1452, 1142, 1070 cm^{-1}; **^{1}H NMR** (500 MHz, Chloroform-*d*) δ 7.36 – 7.32 (m, 2H), 7.21 (d, *J* = 5.0 Hz, 2H), 7.20 – 7.15 (m, 5H), 7.11 (t, *J* = 7.2 Hz, 1H), 3.59 (d, *J* = 13.2 Hz, 1H), 3.50 – 3.74 (m, 4H), 3.43 (d, *J* = 13.2 Hz, 1H), 2.38 (t, *J* = 7.4 Hz, 2H), 2.15 (s, 3H), 1.99 – 1.89 (m, 2H) ppm; **^{13}C NMR** (126 MHz, Chloroform-*d*) δ 173.5, 139.0, 131.7, 128.8, 128.2, 128.1, 127.9, 126.9, 123.1, 86.3, 86.2, 59.4, 54.8, 51.4, 37.2, 30.8, 28.6 ppm; **HRMS** (ESI) *m/z* for $C_{21}H_{24}NO_2$ ($[M+H]^+$): 322.1802; Found: 322.1809; **Chiral HPLC** (Chiralpak OJ-H, hexane/*i*-PrOH = 99/1, 1 mL/min., λ = 254 nm), t_R (major) = 16.9 min., t_R (minor) = 14.5 min., 92% *ee*.

Supplementary Fig. 18. HPLC traces of 3p

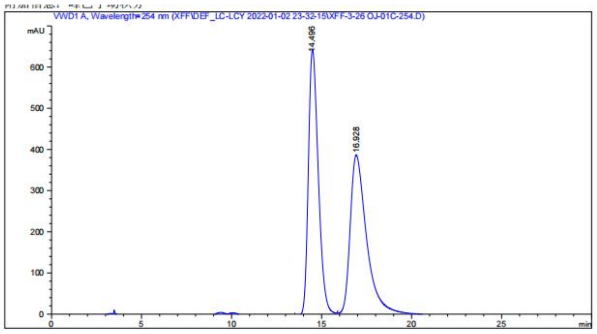

面积百分比报告

排序 : 信号
乘积因子: : 1.0000
稀释因子: : 1.0000
内标使用乘积因子和稀释因子

信号 1: VWD1 A, Wavelength=254 nm

峰 #	保留时间 [min]	类型	峰宽 [min]	峰面积 [mAU*s]	峰高 [mAU]	峰面积 %
1	14.496	BV	0.5919	2.47964e4	645.91028	49.6676
2	16.928	VB	0.9672	2.51282e4	389.21127	50.3324

总量 : 4.99246e4 1035.12155

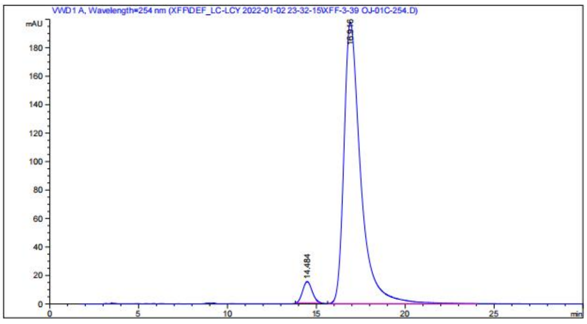

面积百分比报告

排序 : 信号
乘积因子: : 1.0000
稀释因子: : 1.0000
内标使用乘积因子和稀释因子

信号 1: VWD1 A, Wavelength=254 nm

峰 #	保留时间 [min]	类型	峰宽 [min]	峰面积 [mAU*s]	峰高 [mAU]	峰面积 %
1	14.484	MM R	0.6125	550.14624	14.97049	4.0985
2	16.916	BB	0.9773	1.28728e4	197.52335	95.9015

总量 : 1.34230e4 212.49384

(*S*)-(–)-1-(4-Methyl-1-phenylpent-1-yn-3-yl)piperidin-4-one (3q)

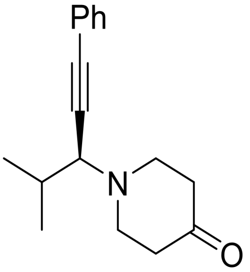

Following general procedure C, the reaction of amide **1m** (67.9 mg, 0.4 mmol) with alkyne **2a** (52 μL, 0.48 mmol) gave, after FC (eluent: EtOAc/*n*-hexane = 1: 100), propargylic amine **3q** (93.9 mg, 92% yield, 90% *ee*) as a white solid; M. p. 95 – 96 °C; $[\alpha]_D^{25}$ = –23.8 (*c* 1, $CHCl_3$) {lit.: $[\alpha]_D^{31}$ = –22.6 (*c* 1.00, $CHCl_3$), 90% ee}[11]; **IR** (film) $\tilde{\nu}$: 2960, 2909, 2813, 1716, 1334, 1212, 1074 cm^{-1}; **^{1}H NMR** (400 MHz, Chloroform-*d*) δ 7.41 – 7.39 (m, 2H), 7.31 – 7.25 (m, 3H), 3.18 (d, *J* = 10.0 Hz, 1H), 3.01 – 2.95 (m, 2H), 2.80 – 2.74 (m, 2H), 2.55 – 2.42 (m, 4H), 1.97 – 1.88 (m, 1H), 1.15 (d, *J* = 6.6 Hz, 3H), 1.08 (d, *J* = 6.6 Hz, 3H) ppm; **^{13}C NMR** (100 MHz, Chloroform-*d*) δ 209.3, 131.6, 128.2, 127.9, 123.1, 86.4, 86.1, 64.4, 49.4, 41.5 (2C), 31.0 (2C), 20.6, 19.8 ppm; **HRMS** (ESI) *m/z* for $C_{17}H_{22}NO$ ($[M+H]^+$): 256.1696; Found: 256.1701; **Chiral HPLC** (Chiralpak OD-H, hexane/*i*-PrOH = 99/1, 1 mL/min., λ = 254 nm), t_R (major) = 6.7 min., t_R (minor) = 7.7 min., 90% *ee*.

Supplementary Fig. 19. HPLC traces of 3q

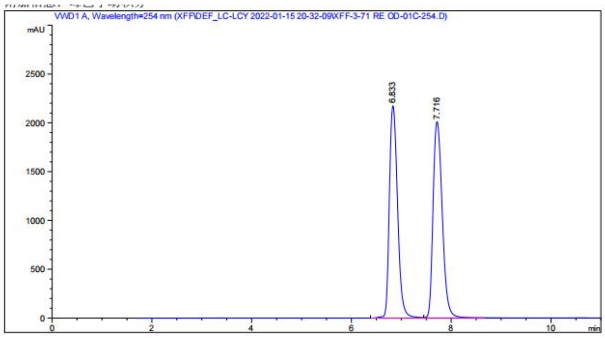

面积百分比报告

排序 : 信号
乘积因子: : 1.0000
稀释因子: : 1.0000
内标使用乘积因子和稀释因子

信号 1: VWD1 A, Wavelength=254 nm

峰 #	保留时间 [min]	类型	峰宽 [min]	峰面积 [mAU*s]	峰高 [mAU]	峰面积 %
1	6.833	BV	0.1809	2.47349e4	2171.87842	49.1065
2	7.716	VB	0.2028	2.56350e4	2009.42981	50.8935

总量 : 5.03700e4 4181.30823

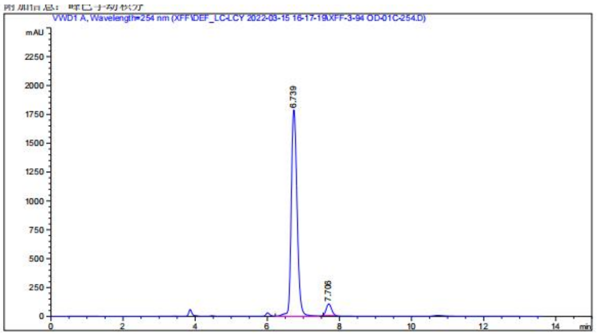

面积百分比报告

排序 : 信号
乘积因子: : 1.0000
稀释因子: : 1.0000
内标使用乘积因子和稀释因子

信号 1: VWD1 A, Wavelength=254 nm

峰 #	保留时间 [min]	类型	峰宽 [min]	峰面积 [mAU*s]	峰高 [mAU]	峰面积 %
1	6.739	VV	0.1760	2.00699e4	1787.91431	95.2506
2	7.706	MM R	0.1670	1000.73322	99.89236	4.7494

总量 : 2.10707e4 1887.80667

(*S*)-(+)-5-(Dimethylamino)-1,7-piphenylhept-6-yn-1-one (3r)

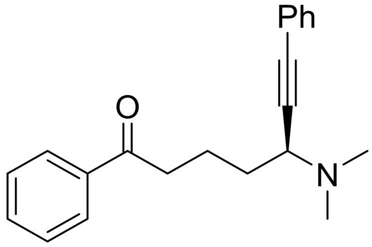

Following general procedure C, the reaction of keto amide **1n** (87.7 mg, 0.4 mmol) with alkyne **2a** (52 μL, 0.48 mmol) gave, after FC (eluent: EtOAc/*n*-hexane = 1: 100), propargylic amine **3r** (106.3 mg, 87% yield, 90% *ee*) as a colorless oil; $[\alpha]_D^{25}$ = +10.8 (*c* 1, $CHCl_3$); **IR** (film) $\tilde{v}$: 2903, 2847, 2781, 1598, 1451, 1311, 1024 cm^{-1}; **^{1}H NMR** (500 MHz, Chloroform-*d*) δ 7.97 – 7.95 (m, 2H), 7.52 (t, *J* = 7.3 Hz, 1H), 7.45 – 7.40 (m, 4H), 7.29 – 7.25 (m, 3H), 3.57 (t, *J* = 7.5 Hz, 1H), 3.04 (t, *J* = 7.9 Hz, 2H), 2.32 (s, 6H), 2.01 – 1.90 (m, 2H), 1.84 – 1.76 (m, 2H) ppm; **^{13}C NMR** (126 MHz, Chloroform-*d*) δ 199.8, 136.9, 132.8, 131.7, 128.5, 128.1, 127.9, 127.8, 123.2, 86.5, 86.2, 57.9, 41.4 (2C), 38.1, 33.3, 21.4 ppm; **HRMS** (ESI) *m/z* for $C_{21}H_{24}NO$ ($[M+H]^+$): 306.1852; Found: 306.1859; **Chiral HPLC** (Chiralpak OD-H, hexane/*i*-PrOH = 99/1, 1 mL/min., λ = 254 nm), t_R (major) = 34.9 min., t_R (minor) = 17.7 min., 90% *ee*.

Supplementary Fig. 20. HPLC traces of 3r

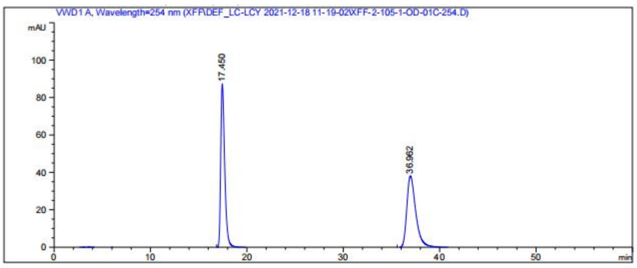

面积百分比报告

排序 : 信号
乘积因子: : 1.0000
稀释因子: : 1.0000
内标使用乘积因子和稀释因子

信号 1: VWD1 A, Wavelength=254 nm

峰 #	保留时间 [min]	类型	峰宽 [min]	峰面积 [mAU*s]	峰高 [mAU]	峰面积 %
1	17.450	BB	0.4353	2509.37231	87.30737	50.0446
2	36.962	BB	0.9860	2504.89502	38.25008	49.9554

总量 : 5014.26733 125.55745

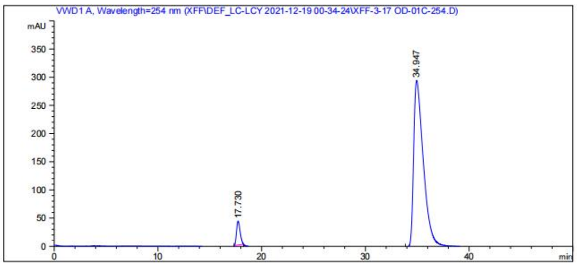

面积百分比报告

排序 : 信号
乘积因子: : 1.0000
稀释因子: : 1.0000
内标使用乘积因子和稀释因子

信号 1: VWD1 A, Wavelength=254 nm

峰 #	保留时间 [min]	类型	峰宽 [min]	峰面积 [mAU*s]	峰高 [mAU]	峰面积 %
1	17.730	MM R	0.4037	1022.64850	42.22220	5.0680
2	34.947	BB	0.9635	1.91559e4	295.01599	94.9320

总量 : 2.01785e4 337.23819

(*S*)-(–)-5-(Dimethylamino)-1-(4-fluorophenyl)-7-phenylhept-6-yn-1-one (3s)

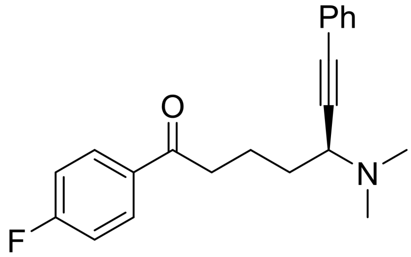

Following general procedure C, the reaction of keto amide **1o** (94.9 mg, 0.4 mmol) with alkyne **2a** (52 µL, 0.48 mmol) gave, after FC (eluent: EtOAc/*n*-hexane = 1: 100), propargylic amine **3s** (121.6 mg, 94% yield, 90% *ee*) a pale yellow oil; $[\alpha]_D^{25}$ = +16.0 (*c* 1, $CHCl_3$); **IR** (film) $\tilde{v}$: 2940, 1686, 1597, 1505, 1494, 1230, 1156, 914, 834, 756, 691 cm^{-1}; **^{1}H NMR** (400 MHz, Chloroform-*d*) δ 7.88 – 7.81 (m, 2H), 7.31 – 7.28 (m, 2H), 7.18 – 7.12 (m, 3H), 7.00 – 6.93 (m, 2H), 3.46 (t, *J* = 7.5 Hz, 1H), 2.88 (t, *J* = 7.2 Hz, 2H), 1.88 – 1.78 (m, 2H), 1.70 – 1.64 (m, 2H) ppm; **^{13}C NMR** (100 MHz, Chloroform-*d*) δ 197.9, 165.3 (d, $J_{C\text{-}F}$ = 250.2 Hz), 133.1(d, $J_{C\text{-}F}$ = 3.0 Hz), 130.4(d, $J_{C\text{-}F}$ = 8.9 Hz), 128.0, 127.1, 123.0, 115.3(d, $J_{C\text{-}F}$ = 21.8 Hz), 86.3, 86.1, 57.7, 41.2 (2C), 37.8, 33.1, 21.2 ppm; **^{19}F NMR** (471 MHz, Chloroform-*d*) δ -105.58; **HRMS** (ESI) *m/z* for $C_{21}H_{23}FNO$ ($[M+H]^+$): 324.1758; Found: 324.1763; **Chiral HPLC** (Chiralpak OD-H, hexane/*i*-PrOH = 99/1, 1 mL/min., λ = 254 nm), t_R (major) = 16.5 min., t_R (minor) = 13.5 min., 90% *ee*.

Supplementary Fig. 21. HPLC traces of 3s

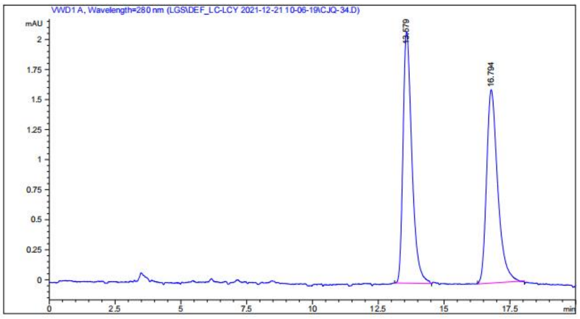

面积百分比报告

排序 : 信号
乘积因子: : 1.0000
稀释因子: : 1.0000
内标使用乘积因子和稀释因子

信号 1: VWD1 A, Wavelength=280 nm

峰 #	保留时间 [min]	类型	峰宽 [min]	峰面积 [mAU*s]	峰高 [mAU]	峰面积 %
1	13.579	BB	0.3515	48.89673	2.09374	50.7263
2	16.794	MM R	0.4917	47.49654	1.61002	49.2737
总量 :				96.39327	3.70376	

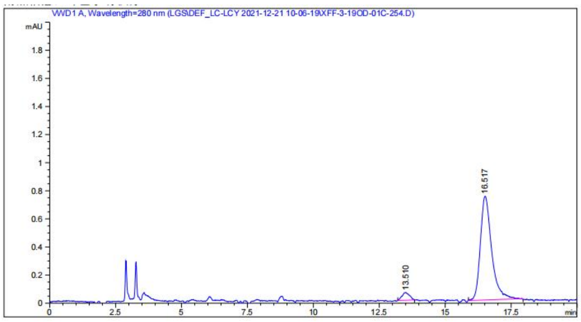

面积百分比报告

排序 : 信号
乘积因子: : 1.0000
稀释因子: : 1.0000
内标使用乘积因子和稀释因子

信号 1: VWD1 A, Wavelength=280 nm

峰 #	保留时间 [min]	类型	峰宽 [min]	峰面积 [mAU*s]	峰高 [mAU]	峰面积 %
1	13.510	MM R	0.3329	1.10670	5.54126e-2	4.7973
2	16.517	MM R	0.4951	21.96257	7.39361e-1	95.2027
总量 :				23.06927	7.94773e-1	

(*S*)-(–)-6-(Dibenzylamino)-8-phenyloct-7-yn-2-one (3t)

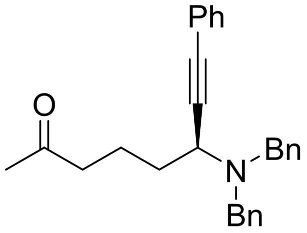

Following general procedure C, the reaction of keto amide **1p** (123.8 mg, 0.4 mmol) with alkyne **2a** (52 μL, 0.48 mmol) gave, after FC (eluent: EtOAc/*n*-hexane = 1: 100), propargylic amine **3t** (145.6 mg, 91% yield, 91% *ee*) as a colorless oil; $[\alpha]_D^{25}$ = –103.9 (*c* 1, $CHCl_3$); **IR** (film) $\tilde{\nu}$: 2930, 2832, 1950, 1716, 1598, 1489, 1453, 1363, 1161, 1119, 1027 cm^{-1}; **^{1}H NMR** (500 MHz, Chloroform-*d*) δ 7.50 – 7.49 (m, 2H), 7.40 (d, *J* = 7.1 Hz, 4H), 7.33 – 7.28 (m, 7H), 7.22 (t, *J* = 7.3 Hz, 2H), 3.87 (d, *J* = 13.7 Hz, 2H), 3.60 (t, *J* = 6.5 Hz, 1H), 3.47 (d, *J* = 13.7 Hz, 2H), 2.22 (t, *J* = 7.2 Hz, 1H), 2.01 (s, 3H), 1.87 – 1.63 (m, 4H) ppm; **^{13}C NMR** (126 MHz, Chloroform-*d*) δ 208.4, 139.6, 131.8, 128.9, 128.2, 128.1, 127.9, 126.9, 123.3, 87.4, 85.4, 54.9, 51.5, 42.7, 32.9, 29.7, 20.3 ppm; **HRMS** (ESI) *m/z* for $C_{28}H_{30}NO$ ($[M+H]^+$): 396.2322; Found: 396.2330; **Chiral HPLC** (Chiralpak OD-H, hexane/*i*-PrOH = 99/1, 1 mL/min., λ = 254 nm), t_R (major) = 8.1 min., t_R (minor) = 13.4 min., 91% *ee*.

Supplementary Fig. 22. HPLC traces of 3t

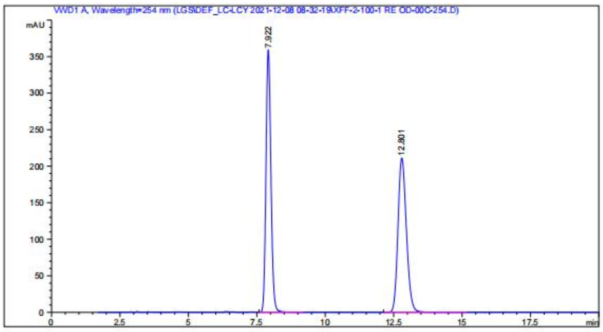

面积百分比报告

排序 : 信号
乘积因子: : 1.0000
稀释因子: : 1.0000
内标使用乘积因子和稀释因子

信号 1: VWD1 A, Wavelength=254 nm

峰 #	保留时间 [min]	类型	峰宽 [min]	峰面积 [mAU*s]	峰高 [mAU]	峰面积 %
1	7.922	VB	0.1962	4498.59131	358.86368	49.9179
2	12.801	BB	0.3313	4513.38428	211.17439	50.0821

总量 : 9011.97559 570.03807

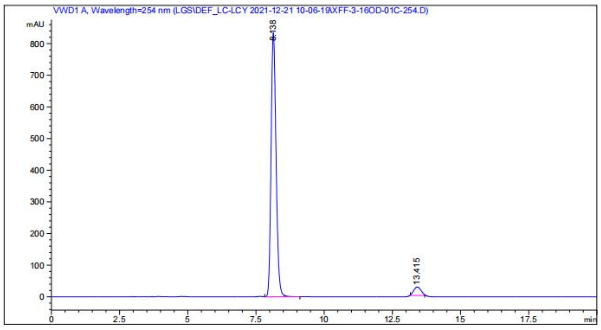

面积百分比报告

排序 : 信号
乘积因子: : 1.0000
稀释因子: : 1.0000
内标使用乘积因子和稀释因子

信号 1: VWD1 A, Wavelength=254 nm

峰 #	保留时间 [min]	类型	峰宽 [min]	峰面积 [mAU*s]	峰高 [mAU]	峰面积 %
1	8.138	VB	0.1963	1.05967e4	833.81494	95.6115
2	13.415	MM R	0.3054	486.38403	26.54585	4.3885

总量 : 1.10831e4 860.36079

(*S*)-(–)-6-(Dibenzylamino)dodec-7-yn-2-one (3u)

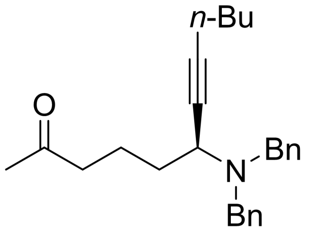

Following general procedure C, the reaction of keto amide **1p** (123.8 mg, 0.4 mmol) with alkyne **2f** (54 µL, 0.48 mmol) gave, after FC (eluent: EtOAc/*n*-hexane = 1: 100), propargylic amine **3u** (118.6 mg, 79% yield, 90% *ee*) as a colorless oil; $[\alpha]_D^{25}$ = –164.7 (*c* 1, $CHCl_3$); **IR** (film) $\tilde{\nu}$: 2932, 2859, 1716, 1494, 1453, 1361, 1160, 1118, 1072 cm^{-1}; **^{1}H NMR** (400 MHz, Chloroform-*d*) δ 7.37 (d, *J* = 7.1 Hz, 4H), 7.33 – 7.26 (m, 4H), 7.25 – 7.18 (m, 2H), 3.77 (d, *J* = 13.7 Hz, 2H), 3.37 (d, *J* = 13.7 Hz, 2H), 3.32 (t, *J* = 7.6 Hz, 1H), 2.33 (t, *J* = 7.2 Hz, 2H), 2.28 (t, *J* = 6.9 Hz, 2H), 2.07 (s, 3H), 1.76 – 1.62 (m, 1H), 1.59 – 1.48 (m, 4H), 1.46 – 1.34 (m, 3H), 0.97 (t, *J* = 7.1 Hz, 3H) ppm; **^{13}C NMR** (100 MHz, Chloroform-*d*) δ 209.0, 140.0, 128.8, 128.3, 126.8, 85.1, 77.7, 54.8, 51.5, 43.6, 33.8, 31.3, 29.8, 25.9, 23.4, 21.9, 18.4, 13.6 ppm; **HRMS** (ESI) *m/z* for $C_{24}H_{32}N$ ($[M+H]^+$): 376.2635; Found: 376.2639; **Chiral HPLC** (Chiralpak OD-H, hexane/*i*-PrOH = 99/1, 1 mL/min., λ = 254 nm), t_R (major) = 5.2 min., t_R (minor) = 7.8 min., 90% *ee*.

Supplementary Fig. 23. HPLC traces of 3u

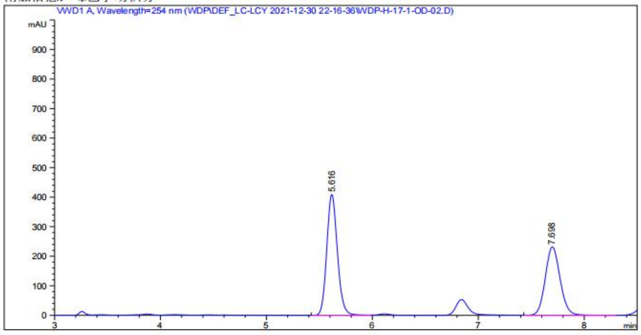

面积百分比报告

排序 : 信号
乘积因子: : 1.0000
稀释因子: : 1.0000
内标使用乘积因子和稀释因子

信号 1: VWD1 A, Wavelength=254 nm

峰 #	保留时间 [min]	类型	峰宽 [min]	峰面积 [mAU*s]	峰高 [mAU]	峰面积 %
1	5.616	BV	0.1094	2848.24707	408.14145	55.9429
2	7.698	VV	0.1512	2243.09570	231.19760	44.0571

总量 : 5091.34277 639.33905

*** 报告结束 ***

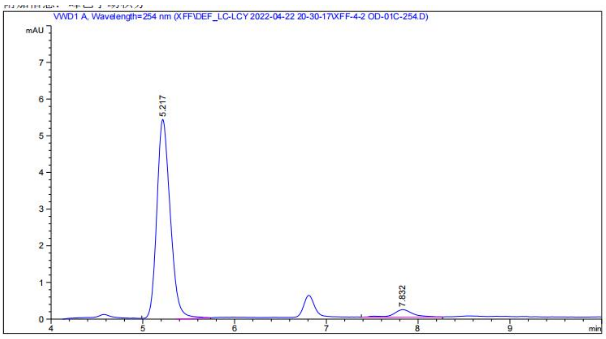

面积百分比报告

排序 : 信号
乘积因子: : 1.0000
稀释因子: : 1.0000
内标使用乘积因子和稀释因子

信号 1: VWD1 A, Wavelength=254 nm

峰 #	保留时间 [min]	类型	峰宽 [min]	峰面积 [mAU*s]	峰高 [mAU]	峰面积 %
1	5.217	VB	0.1552	55.78874	5.46177	94.9342
2	7.832	BB	0.2198	2.97697	2.02159e-1	5.0658

总量 : 58.76571 5.66393

*** 报告结束 ***

(*S*)-(–)-6-(Dibenzylamino)heptadec-7-yn-2-one (3v)

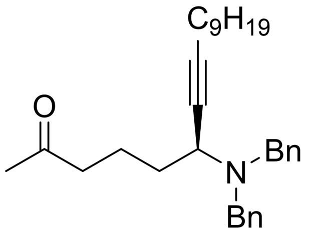

Following general procedure C, the reaction of keto amide **1p** (123.6 mg, 0.4 mmol) with alkyne **2g** (54 µL, 0.48 mmol) gave, after FC (eluent: EtOAc/*n*-hexane = 1: 10), propargylic amine **3v** (151.5 mg, 85% yield, 90% *ee*) as a colorless oil; $[\alpha]_D^{25}$ = –71.5 (*c* 1, $CHCl_3$); **IR** (film) $\tilde{\nu}$: 2926, 2854, 1717, 1494, 1453, 1362, 1161, 1118, 1075, 1027 cm^{-1}; **^{1}H NMR** (500 MHz, Chloroform-*d*) δ 7.37 (d, *J* = 7.0 Hz, 4H), 7.29 (t, *J* = 7.5 Hz, 4H), 7.21 (t, *J* = 7.3 Hz, 2H), 3.77 (d, *J* = 13.6 Hz, 2H), 3.36 (d, *J* = 13.7 Hz, 2H), 3.34 (d, *J* = 13.7 Hz, 2H), 2.27 (t, *J* = 6.9 Hz, 2H), 2.20 (t, *J* = 6.2 Hz, 2H), 2.02 (s, 3H), 1.75 – 1.60 (m, 3H), 1.60 – 1.51 (m, 3H), 1.50 – 1.44 (m, 2H), 1.37-1.26 (m, 10H), 0.88 (t, *J* = 6.5 Hz, 3H) ppm; **^{13}C NMR** (126 MHz, Chloroform-*d*) δ 208.7, 139.95, 128.9, 128.2, 126.8, 85.3, 77.5, 54.8 (2C), 51.1, 42.8, 33.3, 31.9, 29.7, 29.6, 29.3, 29.2 (2C), 28.9, 22.6, 20.5, 18.7, 14.1 ppm; **HRMS** (ESI) *m/z* for $C_{13}H_{44}NO$ ($[M+H]^+$): 446.3417; Found: 446.3414; **Chiral HPLC** (Chiralpak OD-H, hexane/*i*-PrOH = 99/1, 1 mL/min., λ = 254 nm), t_R (major) = 5.6 min., t_R (minor) = 7.6 min., 90% *ee*.

Supplementary Fig. 24. HPLC traces of 3v

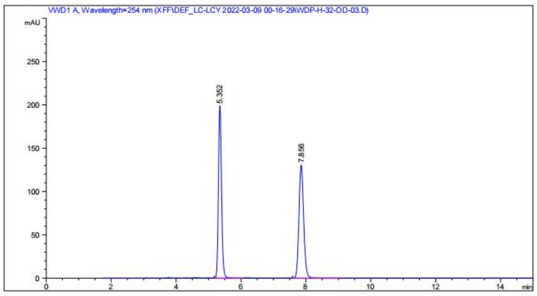

面积百分比报告

排序 : 信号
乘积因子: : 1.0000
稀释因子: : 1.0000
内标使用乘积因子和稀释因子

信号 1: VWD1 A, Wavelength=254 nm

峰 #	保留时间 [min]	类型	峰宽 [min]	峰面积 [mAU*s]	峰高 [mAU]	峰面积 %
1	5.352	VV	0.1032	1315.95569	198.94849	49.9381
2	7.856	VB	0.1579	1319.21863	130.58946	50.0619
总量 :				2635.17432	329.53795	

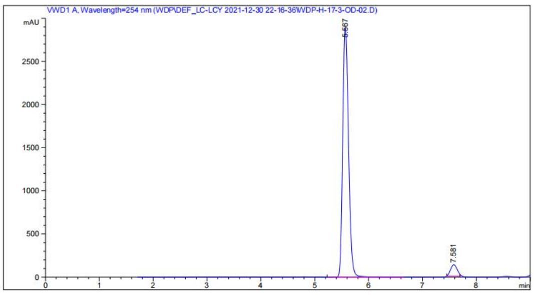

面积百分比报告

排序 : 信号
乘积因子: : 1.0000
稀释因子: : 1.0000
内标使用乘积因子和稀释因子

信号 1: VWD1 A, Wavelength=254 nm

峰 #	保留时间 [min]	类型	峰宽 [min]	峰面积 [mAU*s]	峰高 [mAU]	峰面积 %
1	5.567	BV	0.1199	2.17610e4	2889.74609	95.1580
2	7.581	MM R	0.1397	1107.27917	132.09895	4.8420
总量 :				2.28683e4	3021.84505	

(*S*)-(–)-7-(Dibenzylamino)tridec-8-yn-3-one (3w)

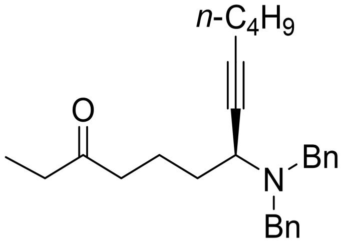

Following general procedure C, the reaction of keto amide **1q** (129.4 mg, 0.4 mmol) with alkyne **2f** (54 µL, 0.48 mmol) gave, after FC (eluent: EtOAc/*n*-hexane = 1: 15), propargylic amine **3w** (126.2 mg, 81% yield, 91% *ee*) as a colorless oil; $[\alpha]_D^{25}$ = –57.4 (*c* 1, $CHCl_3$); **IR** (film) $\tilde{\nu}$: 2955, 2931, 1715, 1494, 1453, 1373, 1118, 1047, 1027 cm^{-1}; **^{1}H NMR** (500 MHz, Chloroform-*d*) δ 7.37 (d, *J* = 7.0 Hz, 4H), 7.29 (t, *J* = 7.5 Hz, 4H), 7.21 (t, *J* = 7.4 Hz, 2H), 3.77 (d, *J* = 13.6 Hz, 2H), 3.36 (d, *J* = 13.7 Hz, 2H), 3.34 – 3.31 (m, 1H), 2.33 – 2.25 (m, 4H), 2.22 – 2.16 (m, 2H), 1.75 – 1.61 (m, 3H), 1.59 – 1.49 (m, 5H), 1.00 (t, *J* = 7.3 Hz, 3H), 0.97 (t, *J* = 7.3 Hz, 3H) ppm; **^{13}C NMR** (126 MHz, Chloroform-*d*) δ 211.3, 139.9, 128.8, 128.1, 126.8, 85.2, 77.5, 54.8 (2C), 51.1, 41.4, 35.7, 33.3, 31.3, 21.9, 20.5, 18.3, 13.6, 7.7 ppm; **HRMS** (ESI) *m/z* for $C_{27}H_{36}NO$ ($[M+H]^+$): 390.2791; Found: 390.2786; **Chiral HPLC** (Chiralpak OD-H, hexane/*i*-PrOH = 99/1, 1 mL/min., λ = 254 nm), t_R (major) = 4.2 min., t_R (minor) = 5.9 min., 91% *ee*.

Supplementary Fig. 25. HPLC traces of 3w

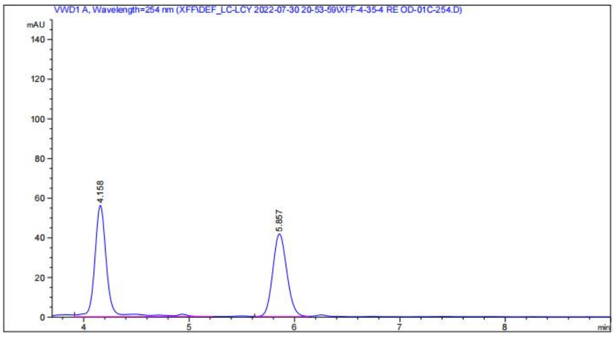

面积百分比报告

排序 : 信号
乘积因子: : 1.0000
稀释因子: : 1.0000
内标使用乘积因子和稀释因子

信号 1: VWD1 A, Wavelength=254 nm

峰 #	保留时间 [min]	类型	峰宽 [min]	峰面积 [mAU*s]	峰高 [mAU]	峰面积 %
1	4.158	VB S	0.1648	449.31293	56.13216	53.5955
2	5.857	VV	0.1448	389.02847	41.70771	46.4045
总量 :				838.34140	97.83987	

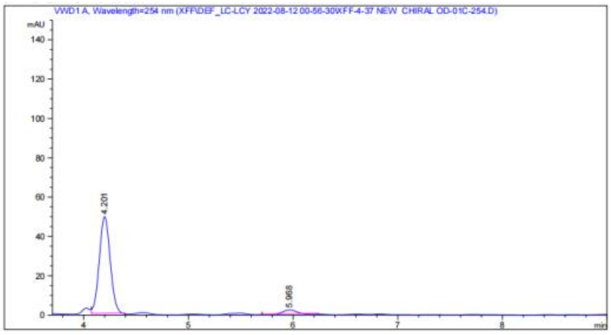

面积百分比报告

排序 : 信号
乘积因子: : 1.0000
稀释因子: : 1.0000
内标使用乘积因子和稀释因子

信号 1: VWD1 A, Wavelength=254 nm

峰 #	保留时间 [min]	类型	峰宽 [min]	峰面积 [mAU*s]	峰高 [mAU]	峰面积 %
1	4.201	FM R	0.1204	355.19739	49.18933	95.6122
2	5.968	MM R	0.1380	16.30060	1.96880	4.3878
总量 :				371.49799	51.15814	

(*S*)-(–)-8-(Dibenzylamino) pentadec-9-yn-5-one (3x)

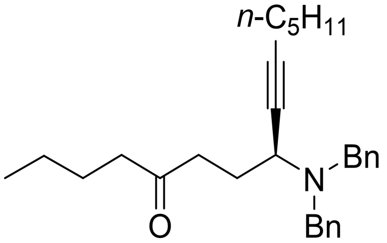

Following general procedure C, the reaction of keto amide **1r** (135mg, 0.4 mmol) with alkyne **2h** (62 μL, 0.48 mmol) gave, after FC (eluent: EtOAc/*n*-hexane = 1: 10), propargylic amine **3x** (138.5 mg, 83% yield, 91% *ee*) as a colorless oil; $[\alpha]_D^{25} = -51.2$ (*c* 1, $CHCl_3$); **IR** (film) $\tilde{v}$: 2956, 2930, 2858, 1715, 1494, 1453, 1364, 1118, 1071, 1028 cm^{-1}; **^{1}H NMR** (500 MHz, Chloroform-*d*) δ 7.34 (d, *J* = 7.6 Hz, 4H), 7.28 (t, *J* = 7.5 Hz, 4H), 7.20 (t, *J* = 7.2 Hz, 2H), 3.78 (d, *J* = 13.7 Hz, 2H), 3.37 (d, *J* = 13.7 Hz, 2H), 3.32 (t, *J* = 6.7 Hz, 1H), 2.48 – 2.39 (m, 2H), 2.34 – 2.21 (m, 4H), 1.92 – 1.87 (m, 2H), 1.62 – 1.53 (m, 2H), 1.51 – 1.42 (m, 4H), 1.41 – 1.34 (m, 2H), 1.29 – 1.22 (m, 2H), 0.94 (t, *J* = 7.4 Hz, 3H), 0.88 (t, *J* = 7.3 Hz, 3H) ppm; **^{13}C NMR** (126 MHz, Chloroform-*d*) δ 210.5, 139.8, 128.8, 128.1, 126.8, 85.7, 77.2, 54.9 (2C), 51.4, 42.5, 39.5, 31.1, 28.8, 27.8, 25.8, 22.3, 22.2, 18.6, 14.0, 13.8 ppm; **HRMS** (ESI) *m/z* for $C_{29}H_{40}NO$ ($[M+H]^+$): 418.3104; Found: 418.3101; **Chiral HPLC** (Chiralpak OD-H, hexane/*i*-PrOH = 99/1, 1 mL/min., λ = 254 nm), t_R (major) = 4.2 min., t_R (minor) = 5.5 min., 91% *ee*.

Supplementary Fig. 26. HPLC traces of 3x

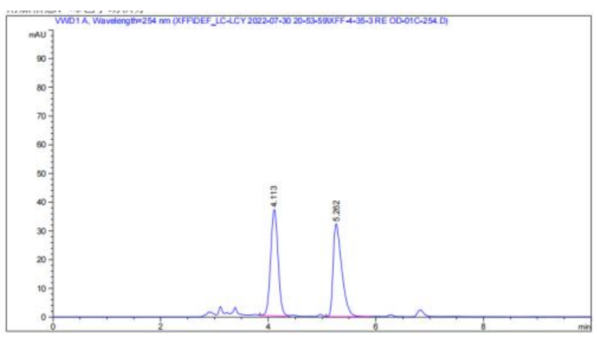

面积百分比报告

排序 : 信号
乘积因子: : 1.0000
稀释因子: : 1.0000
内标使用乘积因子和稀释因子

信号 1: VWD1 A, Wavelength=254 nm

峰 #	保留时间 [min]	类型	峰宽 [min]	峰面积 [mAU*s]	峰高 [mAU]	峰面积 %
1	4.113	VV	0.1507	354.38132	37.00202	49.7876
2	5.262	BV	0.1672	357.40555	32.26331	50.2124
总量 :				711.78687	69.26532	

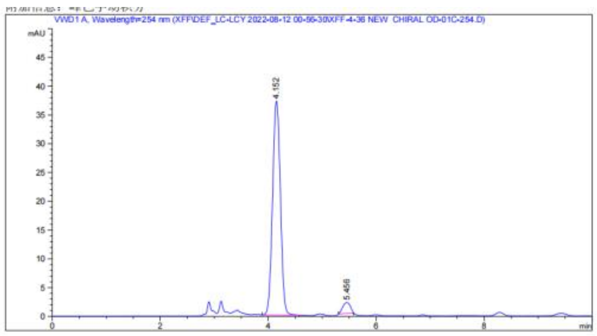

面积百分比报告

排序 : 信号
乘积因子: : 1.0000
稀释因子: : 1.0000
内标使用乘积因子和稀释因子

信号 1: VWD1 A, Wavelength=254 nm

峰 #	保留时间 [min]	类型	峰宽 [min]	峰面积 [mAU*s]	峰高 [mAU]	峰面积 %
1	4.152	BB	0.1603	373.63632	37.16576	95.2849
2	5.456	MM R	0.1632	18.48916	1.88818	4.7151
总量 :				392.12548	39.05394	

(*S*)-(–)-6-(Benzyl(methyl)amino)-10-chlorodec-7-yn-2-one (3y)

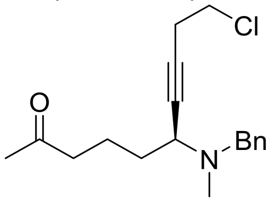

Following general procedure C, the reaction of keto amide **1s** (93.3 mg, 0.4 mmol) with alkyne **2i** (43 µL, 42.5 mg, 0.48 mmol) gave, after FC (eluent: EtOAc/*n*-hexane = 1: 10), propargylic amine **3y** (96.4 mg, 79% yield, 91% *ee*) a colorless oil; $[\alpha]_D^{25} = –101.8$ (*c* 1, $CHCl_3$); **IR** (film) $\tilde{v}$: 2952, 2794, 1715, 1453, 1364, 1161, 1124, 1076, 1020, 828, 739 cm^{-1}; **^{1}H NMR** (400 MHz, Chloroform-*d*) δ 7.38 – 7.18 (m, 5H), 3.65 – 3.60 (m, 3H), 3.44 (d, *J* = 12.0 Hz, 1H), 3.37 – 3.27 (m, 1H), 2.79 – 2.65 (m, 2H), 2.41 – 2.34 (m, 2H), 2.19 (s, 3H), 2.11 (s, 3H), 2.10 (d, *J* = 3.0 Hz, 3H), 1.76 – 1.51 (m, 4H) ppm; **^{13}C NMR** (100 MHz, Chloroform-*d*) δ 208.7, 139.2, 128.9, 128.1, 126.9, 81.6, 79.4, 59.1, 54.8, 43.0, 42.7, 37.4, 33.0, 29.8, 23.1, 20.6 ppm; **HRMS** (ESI) *m/z* for $C_{18}H_{28}NO$ ($[M+H]^+$): 306.1619; Found: 306.1622; **Chiral HPLC** (Chiralpak OJ-H, hexane/*i*-PrOH = 99/1, 1 mL/min., λ = 254 nm), t_R (major) = 13.7 min., t_R (minor) = 15.7 min., 91% *ee*.

Supplementary Fig. 27. HPLC traces of 3y

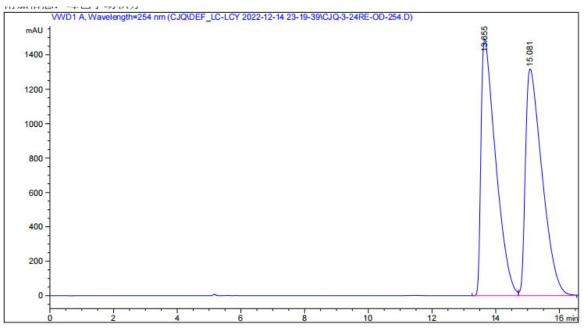

面积百分比报告

排序 : 信号
乘积因子: : 1.0000
稀释因子: : 1.0000
内标使用乘积因子和稀释因子

信号 1: VWD1 A, Wavelength=254 nm

峰 #	保留时间 [min]	类型	峰宽 [min]	峰面积 [mAU*s]	峰高 [mAU]	峰面积 %
1	13.655	BV	0.4724	4.77385e4	1491.37024	49.8306
2	15.081	VV	0.5527	4.80632e4	1317.23547	50.1694

总量 : 9.58017e4 2808.60571

*** 报告结束 ***

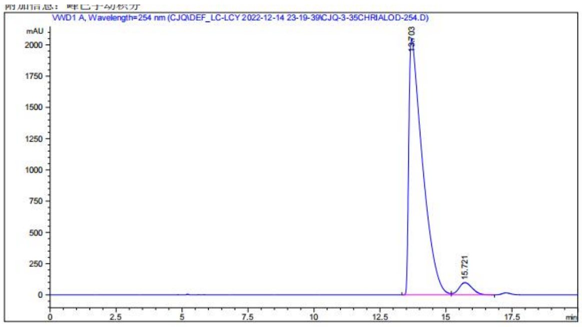

面积百分比报告

排序 : 信号
乘积因子: : 1.0000
稀释因子: : 1.0000
内标使用乘积因子和稀释因子

信号 1: VWD1 A, Wavelength=254 nm

峰 #	保留时间 [min]	类型	峰宽 [min]	峰面积 [mAU*s]	峰高 [mAU]	峰面积 %
1	13.703	BV	0.5192	7.38797e4	2046.24670	95.4849
2	15.721	VV	0.5526	3493.44824	98.30133	4.5151

总量 : 7.73732e4 2144.54803

*** 报告结束 ***

(*S*)-(–)-6-(Dibenzylamino) pentadec-7-yn-2-one (3z)

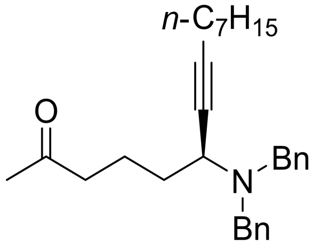

Following general procedure C, the reaction of keto amide **1p** (123.8 mg, 0.4 mmol) with alkyne **2j** (75 μL, 0.48 mmol) gave, after FC (eluent: EtOAc/*n*-hexane = 1: 10), propargylic amine **3ao** (150.2 mg, 90% yield, 98% *ee*) as a colorless oil; $[\alpha]_D^{25} = -135.2$ (*c* 1, $CHCl_3$); **IR** (film) $\tilde{v}$: 2925, 2853, 1716, 1493, 1360, 1160, 1072, 1027 cm^{-1}; **^{1}H NMR** (400 MHz, Chloroform-*d*) δ 7.37 (d, *J* = 7.5 Hz, 4H), 7.29 (t, *J* = 7.4 Hz, 4H), 7.21 (m, 2H), 3.77 (d, *J* = 13.8 Hz, 2H), 3.37 (d, *J* = 13.8 Hz, 2H), 3.31 – 3.30 (m, 1H), 2.33 (t, *J* = 7.1 Hz, 2H), 2.27 (t, *J* = 6.9 Hz, 2H), 2.07 (s, 3H), 1.72 – 1.62 (m, 1H), 1.62 – 1.53 (m, 3H), 1.50 – 1.40 (m, 4H), 1.37 – 1.30 (m, 6H), 0.95 (t, *J* = 7.0 Hz, 3H) ppm; **^{13}C NMR** (100 MHz, Chloroform-*d*) δ 209.1, 140.0, 128.8, 128.1, 126.8, 85.2, 77.7, 54.8 (2C), 51.5, 43.6, 33.8, 31.8, 29.8, 29.2, 28.8, 25.9, 23.4, 22.6, 18.7, 14.1 ppm; **HRMS** (ESI) *m/z* for $C_{24}H_{32}N$ ($[M+H]^+$): 418.3104; Found: 418.3109; **Chiral HPLC** (Chiralpak OD-H, hexane/*i*-PrOH = 99/1, 1 mL/min., λ = 254 nm), t_R (major) = 4.7 min., t_R (minor) = 8.9 min., 98% *ee*

Supplementary Fig. 28. HPLC traces of 3z

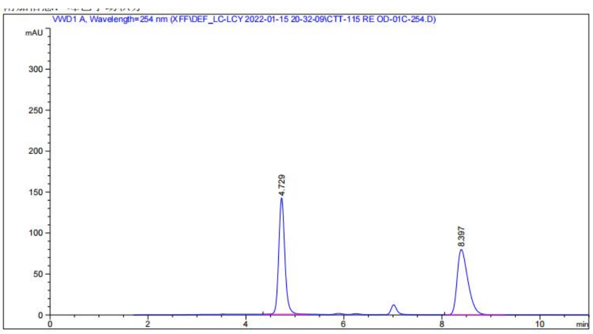

面积百分比报告

排序 : 信号
乘积因子: : 1.0000
稀释因子: : 1.0000
内标使用乘积因子和稀释因子

信号 1: VWD1 A, Wavelength=254 nm

峰 #	保留时间 [min]	类型	峰宽 [min]	峰面积 [mAU*s]	峰高 [mAU]	峰面积 %
1	4.729	BB	0.1331	1230.83557	142.02164	50.2485
2	8.397	BB	0.2332	1218.66296	79.78933	49.7515
总量 :				2449.49854	221.81097	

*** 报告结束 ***

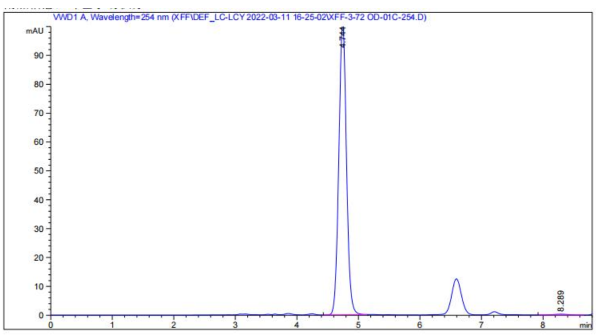

面积百分比报告

排序 : 信号
乘积因子: : 1.0000
稀释因子: : 1.0000
内标使用乘积因子和稀释因子

信号 1: VWD1 A, Wavelength=254 nm

峰 #	保留时间 [min]	类型	峰宽 [min]	峰面积 [mAU*s]	峰高 [mAU]	峰面积 %
1	4.744	BB	0.1349	873.89362	101.05562	99.4907
2	8.289	BB	0.2494	4.47342	2.78500e-1	0.5093
总量 :				878.36704	101.33412	

*** 报告结束 ***

(*S*)-(–)-6-(Benzyl(methyl)amino) pentadec-7-yn-2-one (3aa)

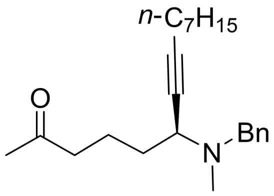

Following general procedure C, the reaction of keto amide **1s** (233.1mg, 1.0 mmol) with alkyne **2j** (188 µL, 1.2 mmol) gave, after FC (eluent: EtOAc/*n*-hexane = 1: 20), propargylic amine **3aa** (273.0 mg, 80% yield, 94% *ee*) as a colorless oil; $[\alpha]_D^{25} = -79.3$ (*c* 1, $CHCl_3$); **IR** (film) $\tilde{\nu}$**:** 2927, 2855, 1716, 1635, 1453, 1362, 1124, 1082, 1027 cm^{-1}; ^{1}**H NMR** (400 MHz, Chloroform-*d*) δ 7.36 – 7.21 (m, 5H), 3.62 (d, *J* = 13.1 Hz, 1H), 3.44 (d, *J* = 13.1 Hz, 1H), 3.32 (t, *J* = 6.0 Hz, 1H), 2.39 (t, *J* = 7.2 Hz, 2H), 2.24 (t, *J* = 6.9 Hz, 2H), 2.17 (s, 3H), 2.11 (s, 3H), 1.74 – 1.65 (m, 3H), 1.60 – 1.50 (m, 3H), 1.47 – 1.40 (m, 2H), 1.37 – 1.26 (m, 6H), 0.90 (t, *J* = 6.0 Hz, 3H) ppm; ^{13}**C NMR** (100 MHz, Chloroform-*d*) δ 208.9, 139.5, 128.9, 128.2, 126.9, 85.9, 77.2, 59.1, 55.0, 43.2, 37.5, 33.4, 31.8, 29.8, 29.2, 28.8, 28.8, 22.6, 20.9, 18.7, 14.1 ppm; **HRMS** (ESI) *m/z* for $C_{23}H_{36}NO$ ($[M+H]^+$): 342.2791; Found: 364.2611; **Chiral HPLC** (Chiralpak OJ-H, hexane/*i*-PrOH = 99/1, 1 mL/min., λ = 254 nm), t_R (major) = 4.9 min., t_R (minor) = 5.3 min., 94% *ee*.

Supplementary Fig. 29. HPLC traces of 3aa

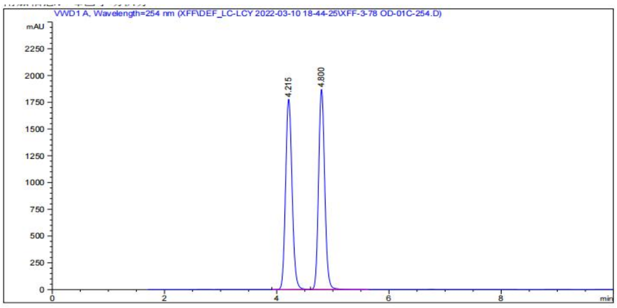

面积百分比报告

排序 : 信号
乘积因子: : 1.0000
稀释因子: : 1.0000
内标使用乘积因子和稀释因子

信号 1: VWD1 A, Wavelength=254 nm

峰 #	保留时间 [min]	类型	峰宽 [min]	峰面积 [mAU*s]	峰高 [mAU]	峰面积 %
1	4.215	BB	0.1247	1.42670e4	1775.77942	50.1488
2	4.800	BB	0.1186	1.41823e4	1866.84290	49.8512

总量 : 2.84493e4 3642.62231

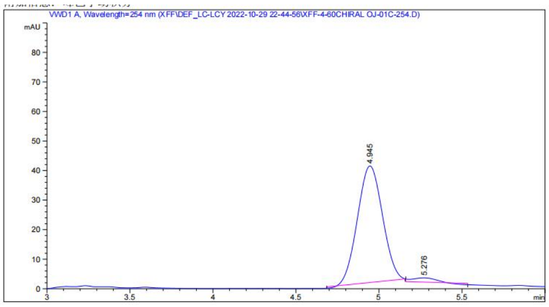

面积百分比报告

排序 : 信号
乘积因子: : 1.0000
稀释因子: : 1.0000
内标使用乘积因子和稀释因子

信号 1: VWD1 A, Wavelength=254 nm

峰 #	保留时间 [min]	类型	峰宽 [min]	峰面积 [mAU*s]	峰高 [mAU]	峰面积 %
1	4.945	MM R	0.1744	413.29678	39.50119	96.7432
2	5.276	MM R	0.1630	13.91342	1.42223	3.2568

总量 : 427.21020 40.92342

(*R*)-(–)-*N*-Benzyl-1,1-diethoxy-*N*-methyloctadec-2-yn-4-amine (3ab)

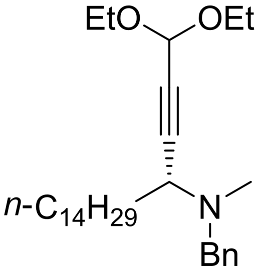

Following general procedure C, the reaction of amide **1t** (345.3 mg, 1.0 mmol) with alkyne **2k** (170 µL, 1.2 mmol), (*R*,*M*)-**L4** (28 mg, 0.02 mmol) gave, after FC (eluent: EtOAc/*n*-hexane = 1: 20), propargylic amine **3ab** (402.5 mg, 88% yield, 94% *ee*) as a colorless oil; $[\alpha]_D^{25}$ = 49.1 (*c* 1, $CHCl_3$); **IR** (film) $\tilde{v}$**:** 2974, 2924, 2853, 1455, 1326, 1133, 1053, 1011 cm^{-1}; **^{1}H NMR** (400 MHz, Chloroform-*d*) δ 7.35 – 7.27 (m, 4H), 7.25 – 7.19 (m, 1H), 5.36 (s, 1H), 3.83–3.75 (m, 2H), 3.69 – 3.56 (m, 3H), 3.48 – 3.39 (m, 2H), 2.20 (s, 3H), 1.72–1.63 (m, 2H), 1.48 – 1.38 (m, 2H), 1.31 – 1.20 (m, 28H), 0.88 (t, *J* = 6.7 Hz, 3H) ppm; **^{13}C NMR** (100 MHz, Chloroform-*d*) δ 139.2, 128.8, 128.1, 126.9, 91.4, 83.2, 80.9, 60.6, 59.1 (2C), 55.3, 37.9, 33.6, 31.9, 29.6 (5C), 29.5 (2C), 29.3, 29.2, 26.3, 22.6, 15.1 (2C), 14.1 ppm; **HRMS** (ESI) *m/z* for $C_{30}H_{52}NO$ ($[M+H]^+$): 458.3993; Found: 458.3998; **HPLC** (Chiralpak AD-H, hexane/*i*-PrOH = 99/1, 1 mL/min., λ = 254 nm), t_R (major) = 5.3 min., t_R (minor) = 6.1 min., 94% *ee*.

Supplementary Fig. 30. HPLC traces of 3ab

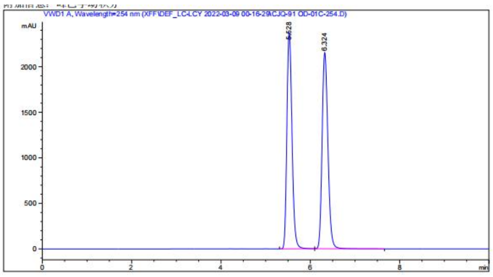

面积百分比报告

排序 : 信号
乘积因子: : 1.0000
稀释因子: : 1.0000
内标使用乘积因子和稀释因子

信号 1: VWD1 A, Wavelength=254 nm

峰 #	保留时间 [min]	类型	峰宽 [min]	峰面积 [mAU*s]	峰高 [mAU]	峰面积 %
1	5.528	BB	0.1251	1.89374e4	2373.78271	49.3039
2	6.324	BB	0.1415	1.94721e4	2153.27026	50.6961

总量 : 3.84094e4 4527.05298

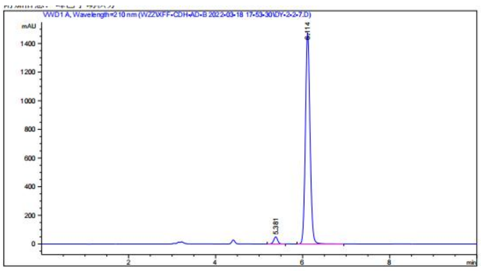

面积百分比报告

排序 : 信号
乘积因子: : 1.0000
稀释因子: : 1.0000
内标使用乘积因子和稀释因子

信号 1: VWD1 A, Wavelength=210 nm

峰 #	保留时间 [min]	类型	峰宽 [min]	峰面积 [mAU*s]	峰高 [mAU]	峰面积 %
1	5.381	BV	0.0970	309.33008	49.41142	2.7540
2	6.114	BV	0.1147	1.09226e4	1470.55872	97.2460

总量 : 1.12320e4 1519.97014

*** 报告结束 ***

(*S*)-(–)-*N*-Benzyl-1,1-diethoxy-*N*-methyloctadec-2-yn-4-amine (*ent*-3ab)

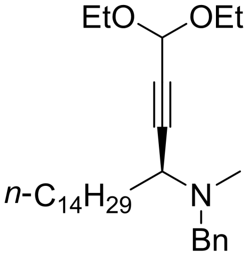

The product was prepared as described for its enantiomer (see above), Following general procedure C, the reaction of amide **1t** (416.2 mg, 1.0 mmol) with alkyne **2k** (170 μL, 1.2 mmol), (*R*,*P*)-L4(28 mg, 0.05 mmol) gave, after FC (eluent: EtOAc/*n*-hexane = 1: 20), propargylic amine ***ent*-3ab** (375.1 mg, 82% yield, 92% *ee*) as a colorless oil; $[\alpha]_D^{25}$ = –48.2 (*c* 1, $CHCl_3$); **HPLC** (Chiralpak AD-H, hexane/*i*-PrOH = 99.5/0.5, 1 mL/min., λ = 254 nm), t_R (major) = 6.3 min., t_R (minor) = 7.5 min., 92% *ee*.

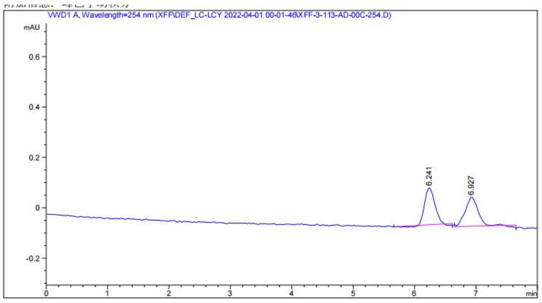

面积百分比报告

排序 : 信号
乘积因子: : 1.0000
稀释因子: : 1.0000
内标使用乘积因子和稀释因子

信号 1: VWD1 A, Wavelength=254 nm

峰 #	保留时间 [min]	类型	峰宽 [min]	峰面积 [mAU*s]	峰高 [mAU]	峰面积 %
1	6.241	MM R	0.1950	1.70608	1.45844e-1	50.4579
2	6.927	MM R	0.2415	1.67512	1.15601e-1	49.5421

总量 : 3.38120 2.61445e-1

*** 报告结束 ***

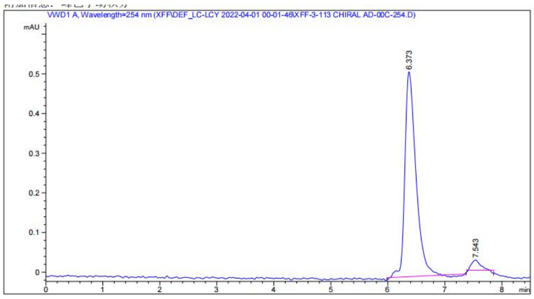

面积百分比报告

排序 : 信号
乘积因子: : 1.0000
稀释因子: : 1.0000
内标使用乘积因子和稀释因子

信号 1: VWD1 A, Wavelength=254 nm

峰 #	保留时间 [min]	类型	峰宽 [min]	峰面积 [mAU*s]	峰高 [mAU]	峰面积 %
1	6.373	MM R	0.2256	6.99114	5.16386e-1	96.1666
2	7.543	MM R	0.1811	2.78680e-1	2.56433e-2	3.8334

总量 : 7.26982 5.42030e-1

*** 报告结束 ***

(*R*)-(–)-11-(Dibenzylamino)-14,14-diethoxytetradec-12-yn-7-one (3ac)

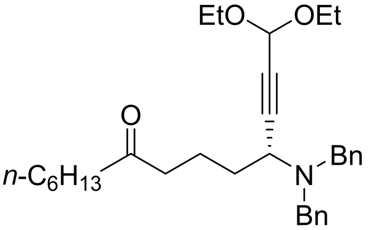

Following general procedure C, the reaction of keto amide **1u** (379.2 mg, 1.0 mmol) with alkyne **2k** (170 µL, 1.2 mmol), (*R*,*M*)-**L4** (28 mg, 0.02 mmol) gave, after FC (eluent: EtOAc/*n*-hexane = 1: 20), propargylic amine **3ac** (373.4 mg, 76% yield, 90% *ee*) as a colorless oil; $[\alpha]_D^{25}$ = 65.2 (*c* 1, $CHCl_3$); **IR** (film) $\tilde{\nu}$**:** 2928, 2028, 1635, 1453, 1122, 1051 cm^{-1}; **^{1}H NMR** (400 MHz, Chloroform-*d*) δ 7.36 (d, *J* = 7.5 Hz, 4H), 7.29 (t, *J* = 7.4 Hz, 4H), 7.22 (t, *J* = 7.2 Hz, 2H), 5.38 (s, 1H), 3.91 – 3.74 (m + d, *J* = 13.6 Hz, 4H), 3.69 – 4.61 (m, 2H), 3.45 (t, *J* = 6.9 Hz, 1H), 3.38 (d, *J* = 13.6 Hz, 2H), 2.25 (t, *J* = 7.5 Hz, 2H), 2.17 (t, *J* = 7.0 Hz, 2H), 1.81 – 1.64 (m, 2H), 1.64 – 1.54 (m, 2H), 1.53 – 1.43 (m, 2H), 1.32 – 1.22 (m, 12H), 0.88 (t, *J* = 6.8 Hz, 3H) ppm; **^{13}C NMR** (100 MHz, Chloroform-*d*) δ 210.8, 139.4, 128.8, 128.2, 126.9, 91.4, 83.3, 80.6, 60.7 (2C), 54.8 (2C), 50.9, 42.7, 41.6, 32.6, 31.5, 28.8, 23.7, 22.4, 20.2, 15.1 (2C), 13.9 ppm; **HRMS** (ESI) *m/z* for $C_{32}H_{46}NO_3$ ($[M+H]^+$): 492.3472; Found: 492.3473; **Chiral HPLC** (Chiralpak AD-H, hexane/*i*-PrOH = 99/1, 1 mL/min., λ = 254 nm), t_R (major) = 7.5 min., t_R (minor) = 6.0 min., 90% *ee*.

Supplementary Fig. 31. HPLC traces of 3ac

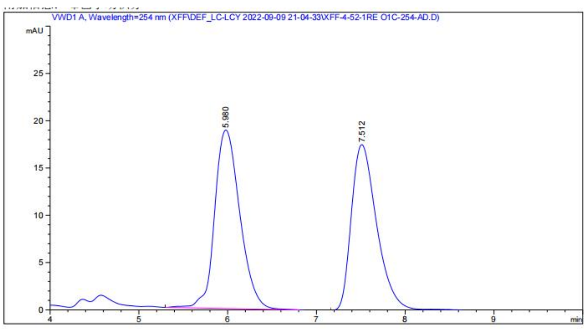

面积百分比报告

排序 : 信号
乘积因子: : 1.0000
稀释因子: : 1.0000
内标使用乘积因子和稀释因子

信号 1: VWD1 A, Wavelength=254 nm

峰 #	保留时间 [min]	类型	峰宽 [min]	峰面积 [mAU*s]	峰高 [mAU]	峰面积 %
1	5.980	BB	0.3059	371.94107	18.87450	51.3450
2	7.512	BB	0.3086	352.45438	17.52410	48.6550

总量 : 724.39545 36.39859

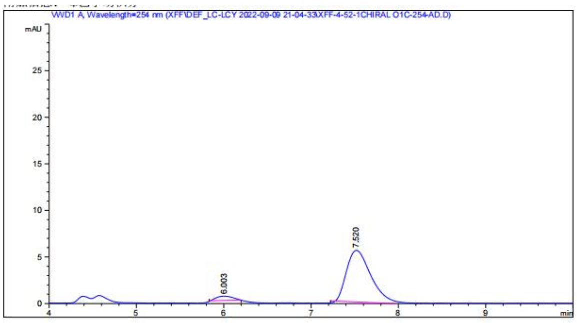

面积百分比报告

排序 : 信号
乘积因子: : 1.0000
稀释因子: : 1.0000
内标使用乘积因子和稀释因子

信号 1: VWD1 A, Wavelength=254 nm

峰 #	保留时间 [min]	类型	峰宽 [min]	峰面积 [mAU*s]	峰高 [mAU]	峰面积 %
1	6.003	MM R	0.2151	5.88439	4.55990e-1	5.1156
2	7.520	MM R	0.3286	109.14443	5.53510	94.8844

总量 : 115.02882 5.99109

*** 报告结束 ***

(*S*)-(–)-8-(2-Butyl-1,3-dioxolan-2-yl)-6-(dibenzylamino)oct-7-yn-2-one (3ad)

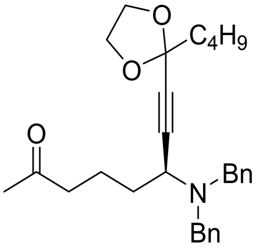

Following general procedure C, the reaction of keto amide **1p** (309.2 mg, 1.0 mmol) with alkyne **2l** (0.19 mL, 184.9 mg, 1.2 mmol) gave, after FC (eluent: EtOAc/*n*-hexane = 1: 10), propargylic amine **3ad** (357.8 mg, 80% yield, 93% *ee*) as a colorless oil; $[\alpha]_D^{25}$ = –57.3 (*c* 1, $CHCl_3$); **IR** (film) $\tilde{\nu}$**:** 2955, 2890, 1716, 1494, 1453, 1363, 1235, 1162, 1119, 1073, 1028, 966, 748, 699 cm^{-1}; **^{1}H NMR** (400 MHz, Chloroform-*d*) δ 7.41 (d, *J* = 7.5 Hz, 4H), 7.35 (t, *J* = 7.4 Hz, 4H), 7.30 –7.23 (m, 2H), 4.21 – 4.18 (m, 2H), 4.09 – 4.06 (m, 2H), 3.86 (d, *J* = 13.7 Hz, 2H), 3.46 (t, *J* = 7.0 Hz, 1H), 3.39 (d, *J* = 13.7 Hz, 2H), 2.27 (t, *J* = 6.8 Hz, 2H), 2.09 (s, 3H), 2.02 – 1.98 (m, 2H), 1.82 – 1.75 (m, 2H), 1.70 – 1.59 (m, 4H), 1.54 – 1.44 (m, 2H), 1.02 (t, *J* = 7.3 Hz, 3H) ppm; **^{13}C NMR** (126 MHz, Chloroform-*d*) δ 140.2, 139.3, 128.7, 128.2, 128.2, 128.0, 126.9, 126.8, 103.5, 83.1, 81.6, 64.4 (2C), 54.8, 53.0, 50.8, 42.5, 39.1, 32.5, 29.6, 26.4, 22.5, 20.1, 13.9 ppm; **HRMS** (ESI) *m/z* for $C_{29}H_{37}NO_3$($[M+H]^+$): 448.2846; Found: 448.2848; **Chiral HPLC** (Chiralpak OD-H, hexane/*i*-PrOH = 99/1, 1 mL/min., λ = 254 nm), t_R (major) = 9.3 min., t_R (minor) = 14.3 min., 93% *ee*.

Supplementary Fig. 32. HPLC traces of 3ad

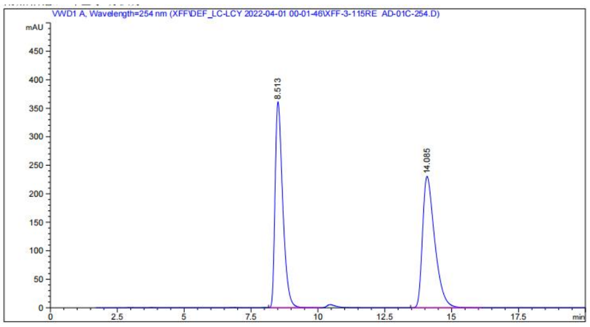

面积百分比报告

排序 : 信号
乘积因子: : 1.0000
稀释因子: : 1.0000
内标使用乘积因子和稀释因子

信号 1: VWD1 A, Wavelength=254 nm

峰 #	保留时间 [min]	类型	峰宽 [min]	峰面积 [mAU*s]	峰高 [mAU]	峰面积 %
1	8.513	VV	0.3019	7181.85254	361.35175	49.6211
2	14.085	BV	0.4771	7291.52832	230.47110	50.3789

总量 : 1.44734e4 591.82285

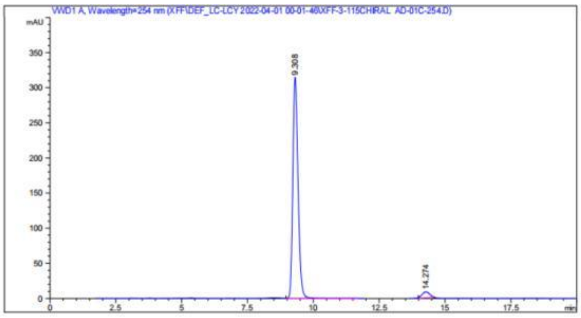

面积百分比报告

排序 : 信号
乘积因子: : 1.0000
稀释因子: : 1.0000
内标使用乘积因子和稀释因子

信号 1: VWD1 A, Wavelength=254 nm

峰 #	保留时间 [min]	类型	峰宽 [min]	峰面积 [mAU*s]	峰高 [mAU]	峰面积 %
1	9.308	VB	0.2129	4343.42188	314.84064	96.3824
2	14.274	MM R	0.3297	163.02560	8.24129	3.6176

总量 : 4506.44748 323.08193

*** 报告结束 ***

General Procedure for the One-pot Catalytic Asymmetric Synthesis of Alkaloids.

General procedure D:

To a flame-dried Schlenk tube were added CuI (9.5 mg, 0.05 mmol), (*R*, *P*)-**L4** (28.0 mg, 0.05 mmol), and toluene: THF = 3:1 (2 mL) under a N_2 atmosphere. The resulting mixture was stirred at room temperature for 5 min. Triethylamine (13 μL, 0.05 mmol) and alkyne **2** (1.2 mmol) were added, and the mixtures was stirred at room temperature for 30 min.

To another flame-dried Schlenk tube were sequentially added $IrCl(CO)(PPh_3)_2$ (7.8 mg, 1 mol%), an keto amide **1** (1.0 mmol, 1 equiv), TMDS (0.36 mL, 2 mmol) and toluene: THF = 3:1 (3 mL) under a N_2 atmosphere at room temperature. After being stirred for 10 minutes, the resulting mixture was added to the abovementioned Schlenk tube containing CuI, (*R*,*P*)-**L4**, triethylamine and alkyne at 0 °C. The mixture was stirred at room temperature for 5 d, then filtered through a short pad of Celite. The filtrate was concentrated under reduced pressure, then the residue was dissolved in MeOH (5 mL). $Pd(OH)_2/C$ was added, and the flask was purged three times with hydrogen. The suspension was stirred for 12 h at room temperature under a hydrogen atmosphere (1 atm). The resulting mixture was filtered through a short pad of Celite, and washed with methanol (50 mL). The filtrate was concentrated and the residue was purified by flash chromatography on silica gel to afford the corresponding amine.

3.2 One-pot Catalytic Asymmetric Synthesis of (–)-*cis*-2-Methyl-6-nonyl-piperidine Alkaloid [(–)-(2*S*,6*R*)-A-3])

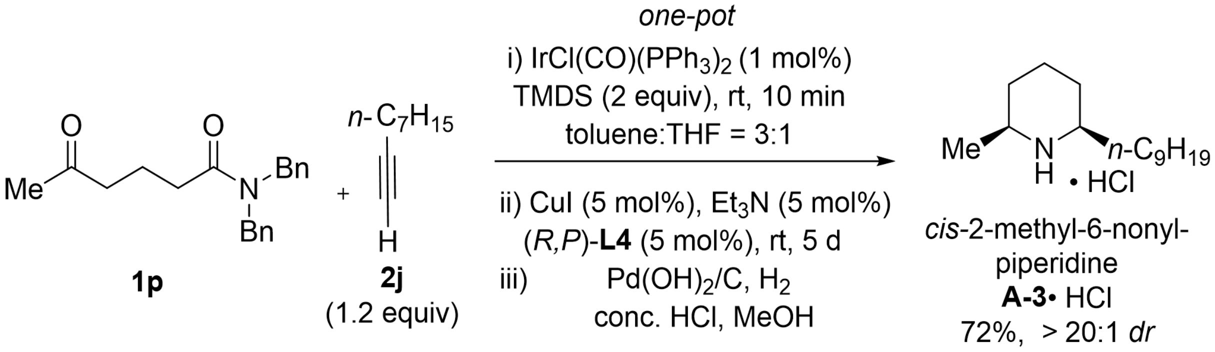

Following general procedure D, the reaction of keto amide **1p** (309.2 mg, 1.0 mmol) with alkyne **2j** (0.197 mL, 1.2 mmol), (*R*,*P*)-**L4** (28.0 mg, 0.05 mmol) and 20% $Pd(OH)_2/C$ (95.4 mg, 30% in mass, 12 h) and HCl (0.3 mL) gave, after FC (eluent: DCM/ MeOH = 20: 1), alkaloid (–)-*cis*-**225I**·HCl [(–)-(2*S*,6*R*)-**A-3**·HCl] (185.4 mg, yield: 71%) as a white solid; M. p. 173 – 174 °C (lit.: M. p. 172 – 173 °C) [12]; $[\alpha]_D^{25}$ = –10.1 (*c* 0.2, $CHCl_3$) {lit.: $[\alpha]_D^{25}$ = –9.0 (*c* 1, $CHCl_3$), *ee* > 99%, *de* > 98%}[12]; **IR** (film) $\tilde{\nu}$: 3430, 2919, 2850, 1586, 1466, 1383, 1259, 1164, 1070, 802, 720 cm^{-1}; **^{1}H NMR** (400 MHz, Chloroform-*d*) δ 9.28 (br. s, 1H), 9.13 (br. s, 1H), 3.35 (s, 1H), 3.09 (m, 1H), 2.36 (m, 1H), 2.10 – 1.81 (m, 6H), 1.66 (d, *J* = 6.4 Hz, 3H), 1.44 – 1.20 (m, 15H), 0.91 (t, *J* = 6.7 Hz, 3H) ppm; **^{13}C NMR** (100 MHz, Chloroform-*d*) δ 58.6, 54.5, 33.2, 31.8, 30.7, 29.5, 29.4, 29.3, 29.2, 27.4, 25.6, 22.9, 22.6, 19.4, 14.0 ppm; **HRMS** (ESI) *m/z* for $C_{15}H_{32}N$ ($[M+H]^+$): 226.2529; Found: 226.2534.

Supplementary Table 1: ^{1}H and ^{13}C NMR data of our synthetic compound (2*S*,6*R*)-**A-3** and those reported in Ref 12.

^{1}H NMR ($CDCl_3$)		^{13}C NMR ($CDCl_3$)	
Our synthetic compound (400 MHz)	Lit. [12]	Our synthetic compound (100 MHz)	Lit. [12]
9.45 (br. s, 1H)	9.39 (br. s, 1H)	58.6	58.8
9.06 (br. s, 1H)	9.01 (br. s, 1H)	54.5	54.7
3.35 (s, 1H)	3.06 (m, 1H)	33.2	33.4
3.09 (m, 1H)	2.88 (m, 1H)	31.8	32.0
2.36 (m, 1H)	2.06 – 2.20 (m, 1H)	30.7	30.9
2.10 – 1.81 (m, 6H)	1.55 – 2.05 (m, 6H)	29.5	29.7
1.66 (d, *J* = 6.4 Hz, 3H)	1.55 (d, *J* = 6.3 Hz, 3H)	29.4	29.6
1.44 – 1.20 (m, 15H)		29.3	29.5

0.91 (t, J = 6.7 Hz, 3H)	1.15 – 1.50 (m, 15H)	29.2	29.4
	0.85 (t, J = 6.9 Hz, 3H)	27.4	27.6
		25.6	25.8
		22.9	23.0
		22.6	22.8
		19.4	19.6
		14.0	14.2

3.3 One-pot Catalytic Asymmetric Synthesis of (−)-*cis*-2-Methyl-6-undecyl-piperidine Alkaloid [(−)-(2*S*,6*R*)-A-4]

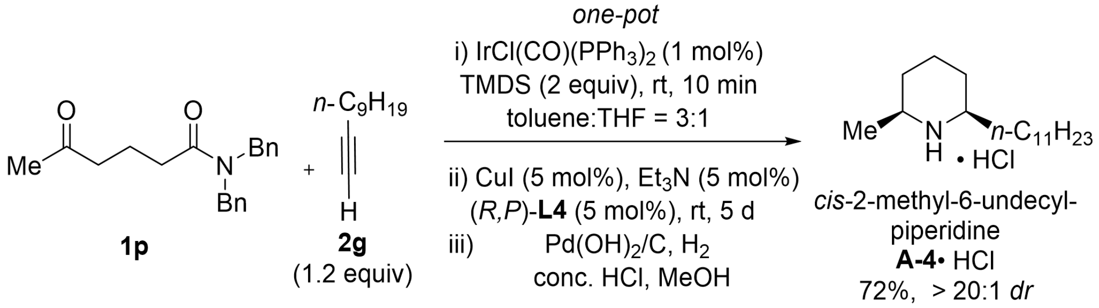

Following general procedure D, the reaction of keto amide **1p** (309.2 mg, 1.0 mmol) with alkyne **2g** (0.23 mL, 1.2 mmol), (*R*,*P*)-**L4** (28.0 mg, 0.05 mmol) and 20% $Pd(OH)_2/C$ (133.6 mg, 30% in mass, 12 h) and HCl (0.3 mL) gave, after FC (eluent: DCM/ MeOH = 20: 1), alkaloid (−)-*cis*-**253J**·HCl [(2*S*,6*R*)-**A-4**·HCl**]** (182.5 mg, yield: 72%) as a white solid; M. p. 243 – 245 °C (lit.: M. p. 240 – 242 °C) [13a]; $[\alpha]_D^{25}$ = –11.2 (*c* 0.2, $CHCl_3$) {lit.: $[\alpha]_D^{25}$ = –10.3 (*c* 1.3, $CHCl_3$)} [13b]; **IR** (film) $\tilde{\nu}$: 3407, 2922, 2851, 1560, 1465, 1404 cm^{-1}; **^{1}H NMR** (400 MHz, Chloroform-*d*) δ 9.42 (br s, 1H), 9.06 (br s, 1H), 3.12 – 3.04 (m, 1H), 2.94 – 2.86 (m, 1H), 2.18 –1.39 (m, 10H), 1.33 – 1.24 (m, 19H), 1.83 – 1.75 (m, 3H), 1.57 (d, J = 6.4 Hz, 3H), 1.49 – 1.39 (m, 1H), 1.32 – 1.21 (m, 18H), 0.88 (t, J = 6.8 Hz, 3H) ppm; **^{13}C NMR** (100 MHz, Chloroform-*d*) δ 58.6, 54.5, 33.2, 31.8, 30.7, 29.6, 29.5, 29.3, 29.2, 27.5, 25.6, 22.8, 22.6, 19.4, 14.1 ppm; **HRMS** (ESI) *m/z* for $C_{17}H_{36}N$ ($[M+H]^+$): 254.2842; Found: 254.2840.

Supplementary Table 2:^{1}H and ^{13}C NMR data of our synthetic compound (2*S*,6*R*)-**A-4** and those reported in Ref 13a.

^{1}H NMR ($CDCl_3$)		^{13}C NMR ($CDCl_3$)	
Our synthetic	Lit. [13a]	Our synthetic	Lit. [13a]

compound (400 MHz)		compound (100 MHz)	
9.42 (br s, 1H)	9.43 (br s, 1H)	58.6	58.6
9.06 (br s, 1H)	9.06 (br s, 1H)	54.5	54.5
3.12-3.04 (m, 1H)	3.13 – 2.96 (m, 1H)	33.2	33.2
2.94-2.86 (m, 1H)	2.96 – 2.80 (m, 1H)	31.8	31.8
2.18-1.39 (m, 10H)	2.20 – 1.48 (m, 10H)	30.7	30.7
1.33-1.24 (m, 19H)	1.48 – 1.10 (m, 19H)	29.6	29.6
0.88 (t, *J* = 6.8 Hz, 3H)	0.86 (t, *J* = 6.5 Hz, 3H)	29.5	29.5
		29.3	29.3
		29.2	29.2
		27.5	27.4
		25.6	25.6
		22.8	22.8
		22.6	22.6
		19.4	19.4
		14.1	14.1

3.4 One-pot Catalytic Asymmetric Synthesis of Alkaloid (−)-*cis*-197F [(−)-(2*S*,6*R*)-A-5]

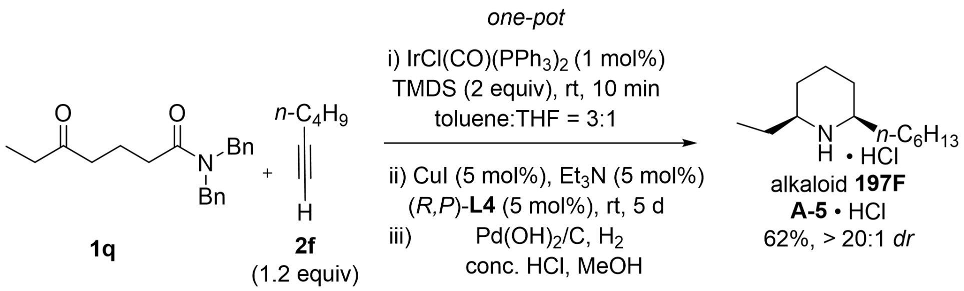

Following general procedure D, the reaction of keto amide **1q** (323.2mg, 1.0 mmol) with alkyne **2f** (0.15 mL, 1.2 mmol), 20% $Pd(OH)_2/C$ (116.8 mg, 30% in mass, 12 h) and HCl (0.3 mL) gave, after FC (eluent: DCM/ MeOH = 10: 1), alkaloid (−)-*cis*-**197F**·HCl [(2*S*,6*R*)-**A-5**·HCl] (122.3 mg, yield: 62%) as a white solid; M. p. 110 – 111 °C; $[\alpha]_D^{25} = -7.3$ (*c* 1, MeOH); **IR** (film) $\tilde{\nu}$: 3357, 2923, 2852, 1654, 1457, 1377, 1080 cm^{-1}; **^{1}H NMR** (500 MHz, Chloroform-*d*) δ 9.19 (s, 1H), 8.96 (s, 1H), 3.14 – 2.68 (m, 2H), 2.52 – 2.15 (m, 2H), 1.98 (t, *J* = 16.0 Hz, 3H), 1.45 – 1.37 (m, 2H), 1.28 (m, 1.13-1.23, 7H), 0.97 (t, *J* = 5.7 Hz, 3H), 0.87 (t, *J* = 5.6 Hz, 3H) ppm; **^{13}C NMR** (100 MHz, Chloroform-*d*) δ 60.2, 58.9, 33.2,

31.7, 29.6, 29.1, 27.8, 27.3, 26.3, 25.6, 22.5, 13.9, 9.9 ppm; **HRMS** (ESI) *m/z* for $C_{13}H_{28}N$ ($[M+H]^+$): 198.2216; Found: 198.2215.

3.5 One-pot Catalytic Asymmetric Synthesis of Alkaloid (−)-*cis*-225C [(−)-(2*S*,5*R*)-A-2]

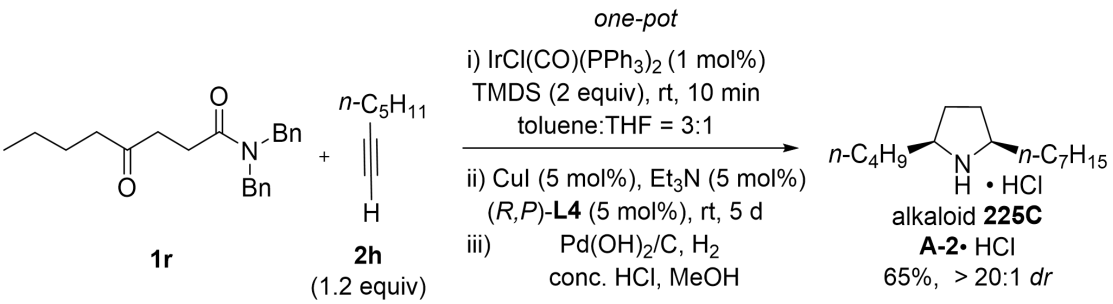

Following general procedure D, the reaction of keto amide **1r** (337.2 mg, 1.0 mmol) with alkyne **2h** (0.15 mL, 1.2 mmol), (*R*,*P*)-**L4** (28.0 mg, 0.05 mmol) and 20% $Pd(OH)_2/C$ (125.2 mg, 30% in mass, 12 h) and HCl (0.4 mL) gave, after FC (eluent: DCM/ MeOH = 20: 1), alkaloid (−)-*cis*-**225C**·HCl [(2*S*,5*R*)-**A-2**·HCl] (146.4 mg, yield: 65%) as a colorless oil; $[\alpha]_D^{25} = -0.73$ (*c* 1, MeOH) {lit.: $[\alpha]_D^{20} = 0$ (*c* 1.3, MeOH)} [14]; **IR** (film) $\tilde{\nu}$: 3440, 2927, 2856, 1555, 1465, 1405 cm^{-1}; **^{1}H NMR** (500 MHz, Chloroform-*d*) δ 10.20 (s, 1H), 8.81 (s, 1H), 3.47 (s, 2H), 1.33 –2.05 (m, 22H), 0.94 – 0.86 (m, 6H) ppm; **^{13}C NMR (100 MHz, Chloroform-*d*)** δ 59.9, 32.6, 31.8, 29.4, 29.2, 29.2, 28.9, 26.9, 22.6, 22.4, 13.8 ppm; **HRMS** (ESI) *m/z* for $C_{15}H_{32}N$ ($[M+H]^+$): 226.2529; Found: 226.2521.

Supplementary Table 3: ^{1}H and ^{13}C NMR data of our synthetic compound alkaloid (−)-*cis*-225C [(2*S*,5*R*)-**A-2**] and those reported in Ref 14a.

^{1}H NMR ($CDCl_3$)		^{13}C NMR ($CDCl_3$)	
Our synthetic compound (500 MHz)	Lit. [14a]	Our synthetic compound (100 MHz)	Lit. [14a]
10.20 (s, 1H)	4.40 (m, 1H)	59.9	59.5
8.81 (s, 1H)	3.09 (m, 2H)	32.6	35.5
3.47 (s, 2H)	1.23-1.91(m, 22H)	31.8	31.7
1.33–2.05 (m, 22H)	0.87-0.89 (m, 6H)	29.4	30.8
0.94-0.86 (m, 6H)		29.2	29.6
		29.0	29.4
		28.9	29.1
		26.9	27.3

		22.6	22.7
		22.4	22.5
		13.8.	13.9

3.6 One-pot Catalytic Asymmetric Synthesis of Alkaloid (−)-*cis*-1,2-Dimethyl-6-nonyl-piperidine [(−)-(2*S*,6*R*)-A-6]

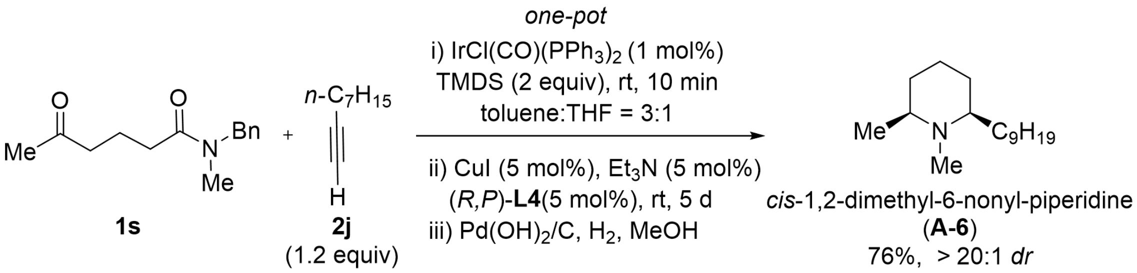

Following general procedure D, the reaction of keto amide **1s** (233.1 mg, 1.0 mmol) with alkyne **2j** (0.19 mL, 1.2 mmol), (*R*,*P*)-**L4** (28.0 mg, 0.05 mmol) and 20% $Pd(OH)_2/C$ (102.4 mg, 30% in mass, 12 h) gave, after FC (eluent: DCM/ MeOH = 10: 1), alkaloid (−)-*cis*-1,2-dimethyl-6-nonyl-piperidine [(2*S*,6*R*)-**A-6**] (181.8 mg, yield: 76%, *dr* > 20:1) as a pale yellow oil; $[\alpha]_D^{25} = –27.9$ (*c* 1, $CHCl_3$); **IR** (film) $\tilde{\nu}$: 2910, 2798, 2723, 1460 cm^{-1}; **^{1}H NMR** (500 MHz, Chloroform-*d*) δ 2.26 – 2.24 (m, 4H), 2.07 (d, J = 9.0 Hz, 1H), 1.75 – 1.63 (m, 3H), 1.58 – 1.49 (m, 1H), 1.43 – 1.22 (m, 18H), 1.12 (d, J = 6.2 Hz, 3H), 0.88 (t, J = 6.8 Hz, 3H) ppm; **^{13}C NMR** (126 MHz, Chloroform-*d*) δ 64.4, 59.7, 33.9, 31.8 (2C), 29.9, 29.5 (2C), 29.2, 26.1, 24.3, 22.5, 20.7, 14.0 ppm; **HRMS** (ESI) *m/z* for $C_{16}H_{34}N$ ($[M+H]^+$): 240.2686; Found: 240.2686.

3.7 One-pot Catalytic Asymmetric Synthesis of Alkaloid (–)-Bgugaine [(–)-(*R*)-A-1)]

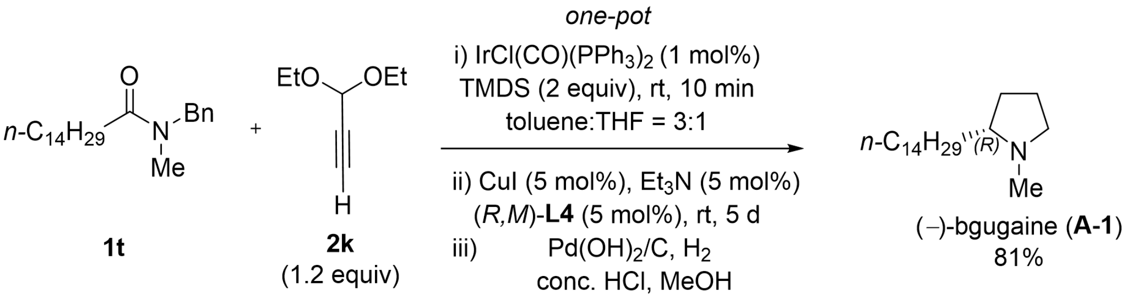

Following general procedure D, the reaction of keto amide **1t** (345.5 mg, 1.0 mmol) with alkyne **2k** (0.17 mL, 1.2 mmol), (*R*,*M*)-**L4** (28.0 mg, 0.05 mmol) and 20% $Pd(OH)_2/C$ (95.4 mg, 30% in mass, 12 h) and HCl (0.3 mL), then the residue were added CH_2Cl_2 (5 mL) and Et_3N (1 mL), and the mixture was stirred for 2 h at RT before treating with a saturated aqueous Na_2CO_3 solution. The resulting mixture

was extracted with CH_2Cl_2 (3 × 5 mL). The combined organic layers were washed with brine, dried over anhydrous Na_2SO_4, filtered and concentrated under reduced pressure. The residue was purified by flash column chromatography (FC) on silica gel (eluent: DCM/MeOH = 20: 1) to afford (-)-bgugaine [(*R*)-**A-1**)] (227.8 mg, yield: 81%) as a pale yellow oil; $[\alpha]_D^{25} = -46.3$ (*c* 1, MeOH) {lit.: $[\alpha]_D^{20} = -48$ (*c* 0.5, MeOH)}[15]; **IR** (film) $\tilde{\nu}$: 2925, 2854, 1462, 1105 cm^{-1}; ^{1}**H NMR** (400 MHz, Chloroform-*d*) δ 3.13 – 3.08 (m, 1H), 2.34 (s, 3H), 2.16 (dd, *J* = 18.0 Hz, *J* = 8.9 Hz, 1H), 2.03 – 1.88 (m, 2H), 1.84 – 1.75 (m, 1H), 1.72 – 1.62 (m, 2H), 1.50 – 1.42 (m, 1H), 1.29 (s, 25H), 0.91 (t, *J* = 6.7 Hz, 3H) ppm; ^{13}**C NMR** (100 MHz, Chloroform-*d*) δ 66.5, 57.3, 40.4, 33.9, 31.9, 30.8, 30.0, 29.7, 29.6, 29.6, 29.4, 26.7, 22.7, 21.8, 14.1 ppm; **HRMS** (ESI) *m/z* for $C_{19}H_{40}N$ ($[M+H]^+$): 282.3155; Found: 282.3154.

Supplementary Table 4:^{1}H and ^{13}C NMR data of our synthetic compound (–)-bgugaine [(*R*)-**A-1**)] and those reported in Ref 15.

^{1}H NMR ($CDCl_3$)		^{13}C NMR ($CDCl_3$)	
Our synthetic compound	Lit. [15]	Our synthetic compound	Lit. [15]
3.13-3.08 (m, 1H)	3.30-3.03 (m, 1H)	66.5	66.5
2.34 (s, 3H)	2.29 (s, 3H)	57.3	57.4
2.16 (dd, *J* = 18.0 Hz, *J* = 8.9 Hz, 1H)	2.11 (dd, *J* = 18.0 Hz, *J* = 8.5 Hz, 1H)	40.4	40.5
		33.9	33.9
2.03 – 1.88 (m, 2H)	1.97 – 1.88 (m, 2H)	31.9	31.9
1.84 – 1.75 (m, 1H)	1.80 – 1.71 (m, 1H)	30.8	30.8
1.72 – 1.62 (m, 2H)	1.69 – 1.62 (m, 2H)	30.0	30.0
1.50-1.42 (m, 1H)	1.46 – 1.38 (m, 1H)	29.7	29.7
1.29 (s, 25H)	1.28 (s, 25H)	29.6	29.6
0.91 (t, *J* = 6.7 Hz, 3H)	0.88 (t, *J* = 7.0 Hz, 3 H)	29.3	29.3
		26.7	26.7
		22.7	22.7
		21.8	21.8
		14.1	14.1

3.8 One-pot Catalytic Asymmetric Synthesis of Alkaloid (+)-Bgugaine (*ent*-A-1)

(+)-Bgugaine was prepared as described for its (–)-enantiomer as a pale-yellow oil in 82% yield. $[\alpha]_D^{25} = 40.1$ (*c* 1, MeOH); ^{1}**H NMR** (400 MHz, Chloroform-*d*) δ 3. 20 – 3.16 (m, 1H), 2.38 (s, 3H), 2.16 (dd, *J* = 18.0 Hz, *J* = 8.9 Hz, 1H), 2.13 – 1.94 (m, 2H), 1.87 – 1.80 (m, 1H), 1.74 – 1.67 (m, 2H), 1.59 – 1.46

(m, 1H), 1.27 (s, 25H), 0.88 (t, J = 6.7 Hz, 3H) ppm; ^{13}**C NMR** (100 MHz, Chloroform-d) δ 66.9, 57.1, 40.2, 33.2, 31.9, 30.6, 29.9, 29.7, 29.7, 29.6, 29.4, 26.7, 22.7, 21.7, 14.1 ppm.

3.9 One-pot Catalytic Asymmetric Synthesis of Alkaloid (–)-209D [(–)-A-7]

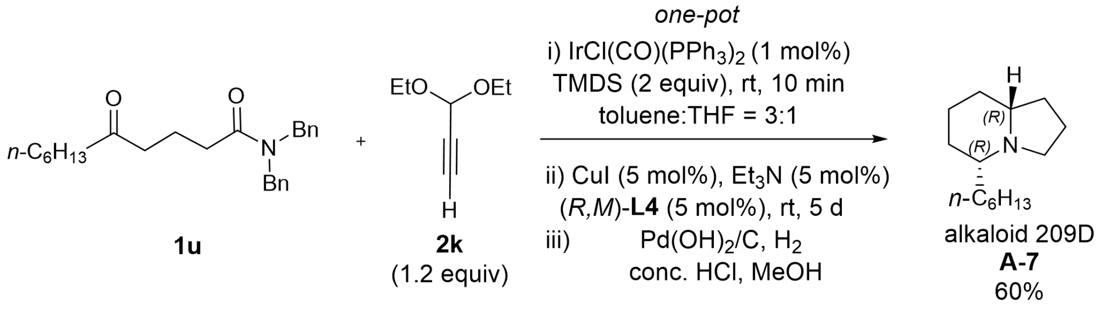

Following general procedure D, the reaction of keto amide **1u** (379.3 mg, 1.0 mmol) with alkyne **2k** (0.17 mL, 1.2 mmol), (R,M)-**L4** (28.0 mg, 0.05 mmol) and 20% $Pd(OH)_2$/C (147.4 mg, 30% in mass, 24 h) and HCl (0.3 mL), then the residue were added CH_2Cl_2 (5 mL) and Et_3N (1 mL), and the mixture was stirred for 2 h at RT before treating with a saturated aqueous Na_2CO_3 solution. The resulting mixture was extracted with CH_2Cl_2 (3 × 5 mL). The combined organic layers were washed with brine, dried over anhydrous Na_2SO_4, filtered and concentrated under reduced pressure. The residue was purified by flash column chromatography (FC) on silica gel (eluent: DCM/MeOH = 10: 1) to afford alkaloid (–)-209D [(–)-**A-7**] (125.5 mg, yield: 60%) as a pale yellow oil; $[\alpha]_D^{25}$ = –62.4 (c 1, $CHCl_3$); {lit.: $[\alpha]_D^{20}$ = –66.5 (c 1, $CHCl_3$)} [16a]; **IR** (film) $\tilde{\nu}$: 2923, 2854, 2782, 1462, 1389 cm^{-1}; ^{1}**H NMR** (400 MHz, Chloroform-d) δ 3.29 (t, J = 8.9 Hz, 1H), 2.01 (q, J = 9.0 Hz, 1H), 1.96 – 1.59 (m, 8H), 1.55 – 1.38 (m, 2H), 1.37 – 1.15 (m, 12H), 0.88 (t, J = 6.5 Hz, 3H) ppm; ^{13}**C NMR** (100 MHz, Chloroform-d) δ 65.1, 64.0, 51.4, 34.4, 31.8, 30.8, 30.6, 30.4, 29.7, 25.8, 24.6, 22.6, 20.3, 14.1 ppm; **HRMS** (ESI) m/z for $C_{17}H_{25}N$ ($[M+H]^+$): 210.2216; Found: 210.2218.

Supplementary Table 5: ^{1}H and ^{13}C NMR data of our synthetic compound alkaloid 209D (**A-7**) and those reported in Ref 16b

^{1}H NMR ($CDCl_3$)		^{13}C NMR ($CDCl_3$)	
Our synthetic compound (400 MHz)	Lit. [16b]	Our synthetic compound (100 MHz)	Lit. [16b]
3.29 (t, J = 8.9 Hz, 1H)<br>2.01 (q, J = 9.0 Hz, 1H)	3.25 (td, J = 8.8, 2.2 Hz, 1H)<br>1.96 (q, J = 8.9 Hz,1H)	65.1<br>64.0<br>51.4	65.2<br>64.1<br>51.7

1.96 – 1.59 (m, 8H)	1.91-1.69 (m, 8H)	34.4	34.8
1.55 – 1.38 (m, 2H)	1.69-1.59 (m, 2H)	31.8	32.0
1.37 – 1.15 (m, 12H)	1.43-1.26 (m, 12H)	30.8	31.2
0.88 (t, *J* = 6.5 Hz, 3H)	0.88 (t, *J* = 6.8, 3 H)	30.6	31.0
		30.4	30.7
		29.7	29.9
		25.8	26.0
		24.6	24.9
		22.6	22.8
		20.3	20.6
		14.1	14.3

3.10 One-pot Catalytic Asymmetric Synthesis of Alkaloid (+)-Monomorine I [(+)-A-8)]

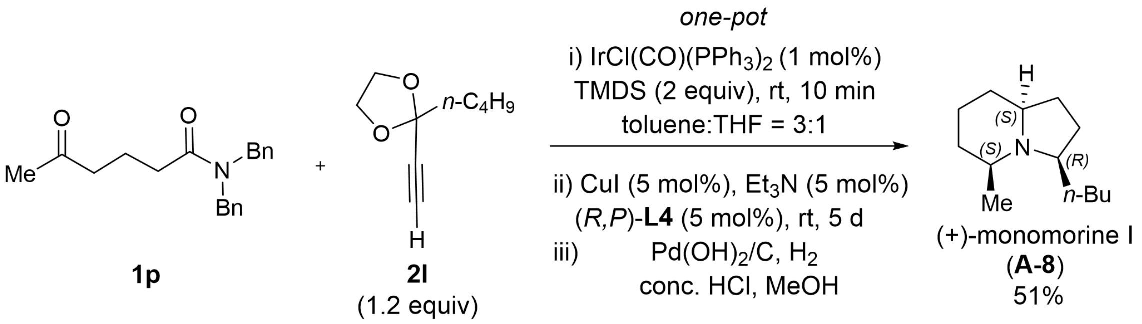

Following general procedure D, the reaction of keto amide **1p** (309.2 mg, 1.0 mmol) with alkyne **2l** (0.19 mL, 184.9 mg, 1.2 mmol), (*R*,*P*)-**L4** (28.0 mg, 0.05 mmol) and $Pd(OH)_2/C$ (147.4 mg, 30% in mass, 24 h) and HCl (0.3 mL) gave. To the residue were added CH_2Cl_2 (5 mL) and Et_3N (1 mL), and the mixture was stirred for 2 h at RT before treating with a saturated aqueous Na_2CO_3 solution. The resulting mixture was extracted with CH_2Cl_2 (3 × 5 mL). The resulting mixture was filtered through a short pad of Celite eluting with methanol (50 mL). The fractions were collected, and concentrated. Then, the residue was dissolved in MeOH (5 mL), 20% $Pd(OH)_2/C$ (147.4 mg, 30% in mass) was added, the flask was purged three times with hydrogen. The suspension was stirred for 12 h at room temperature under a hydrogen pressure of 1 atm as monitored by TLC. The resulting mixture was filtered through a short pad of Celite eluted with methanol (50 mL). The filtrate was concentrated. The combined organic layers filtered and concentrated under reduced pressure. The residue was purified by flash column chromatography (FC) on silica gel (eluent: DCM/MeOH = 10: 1) to afford (+)-monomorine I [(+)-**A-8**)] (99.6 mg, yield: 51%) as a pale yellow oil; $[\alpha]_D^{25}$ = +29.7 (*c* 0.5, *n*-hexane) {lit. $[\alpha]_D^{26}$ = +29.1 (*c*

0.0043, *n*-hexane)[17a]; lit. $[\alpha]_D^{26}$ +33.32 (*c* 1.4, *n*-hexane)[17b]}; **IR** (film) $\tilde{\nu}$: 2970, 2923, 2863, 1650 cm^{-1}; **^{1}H NMR** (500 MHz, Chloroform-*d*) δ 2.53 – 2.49 (m, 1H), 2.28 – 2.26 (m, 1H), 2.15-2.08 (m, 1H), 1.86 – 1.55 (m, 6H), 1.53 – 1.25 (m, 10H), 1.14 (d, *J* = 6.7 Hz, 3H), 0.92 – 0.84 (m, 3H) ppm; **^{13}C NMR** (100 MHz, Chloroform-*d*) δ 67.5, 63.2, 60.6, 38.1, 35.6, 30.2, 29.7, 29.4, 29.3, 24.8, 22.9, 22.6, 14.1 ppm; **HRMS** (ESI) *m/z* for $C_{13}H_{26}N$ ($[M+H]^+$): 196.2060; Found: 196.2063.

Supplementary Table 6: ^{1}H and ^{13}C NMR data of our synthetic compound (+)-monomorine I (**A-8**) and those reported in Ref. 17c.

^{1}H NMR ($CDCl_3$)		^{13}C NMR ($CDCl_3$)	
Our synthetic compound (500 MHz)	Lit.[17c]	Our synthetic compound (100 MHz)	Lit.[17c]
2.53 – 2.49 (m, 1H)	2.49 – 2.43 (m, 1H)	67.5	67.2
2.28 – 2.26 (m, 1H)	2.27 – 2.17 (m, 1H)	63.3	62.9
2.15 – 2.08 (m, 1H)	2.07 – 2.05 (m, 1H)	60.6	60.3
1.86 – 1.25 (m, 16H)	1.18 – 1.84 (m, 16H)	39.1	39.7
1.14 (d, *J* = 6.7 Hz, 3H)	1.23 (d, *J* = 6.4 Hz, 3H)	35.3	35.6
		30.4	30.9
0.86 (t, *J* = 6.8 Hz, 3H)	0.89 (t, *J* = 7.2 Hz, 3H)	30.0	30.3
		29.6	29.7
		29.4	29.4
		24.7	24.9
		22.8	23.0
		22.5	22.9
		14.1	14.1

3.11 One-pot Catalytic Asymmetric Synthesis of Intermediate 4a

1-((2*S*,6*S*)-6-Methylpiperidin-2-yl)heptan-3-one (4a)[17b]

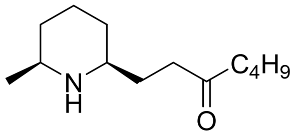

4a

Following general procedure D, the reaction of keto amide **1p** (309.2 mg, 1.0 mmol) with alkyne **2l** (0.19 mL, 184.9 mg, 1.2 mmol), (*R*,*P*)-**L4** (28.0 mg, 0.05 mmol) and $Pd(OH)_2/C$ (147.4 mg, 30% in mass, 24 h) and HCl (0.3 mL) gave. To the residue were added CH_2Cl_2 (5 mL) and Et_3N (1 mL), and the mixture was stirred for 6 h at RT before treating with a saturated aqueous Na_2CO_3 solution. The resulting mixture was extracted with CH_2Cl_2 (3 × 5 mL). The resulting mixture was filtered through a short pad of Celite eluted with methanol (50 mL). The filtrate was concentrated. The combined organic layers filtered and concentrated under reduced pressure. The residue was purified by flash column chromatography (FC) on silica gel (eluent: DCM/MeOH = 20: 1) to afford (2*S*,6*S*)-**4a**] (152.2 mg, yield: 72%, >20:1 *dr*) as a pale yellow oil; $[a]_D^{25}$ - 17.3 (*c* 1, $CHCl_3$); **IR** (film) $\tilde{v}$: 3350, 2956, 1461 cm^{-1}; ^{1}**H NMR** (400 MHz, Chloroform-*d*) δ 2.82-2.75 (m, 1H), 2.70-2.63 (m, 1H), 2.62 – 2.48 (m, 2H), 2.44-2.37(m, 2H), 1.98 – 1.75 (m, 3H), 1.74 – 1.63 (m, 2H), 1.58 – 1.50 (m, 2H), 1.35 – 1.20 (m, 9H), 0.90 (t, *J* = 7.3, 3H); ^{13}**C NMR** (101 MHz, Chloroform-*d*) δ 210.8, 57.0, 53.3, 42.6, 38.9, 32.9, 30.5, 29.7, 25.9, 24.1, 22.4, 21.9, 13.9; **HRMS** (ESI) *m/z* for $C_{13}H_{26}NO$ ($[M+H]^+$):212.2009, Found: 212.2003;

4. Supplementary Notes

4.1 NMR Spectra of Compounds

Supplementary Fig. 33. ^{1}H NMR (400 MHz, $CDCl_3$) and ^{13}C NMR (100 MHz, $CDCl_3$) spectra of 1d

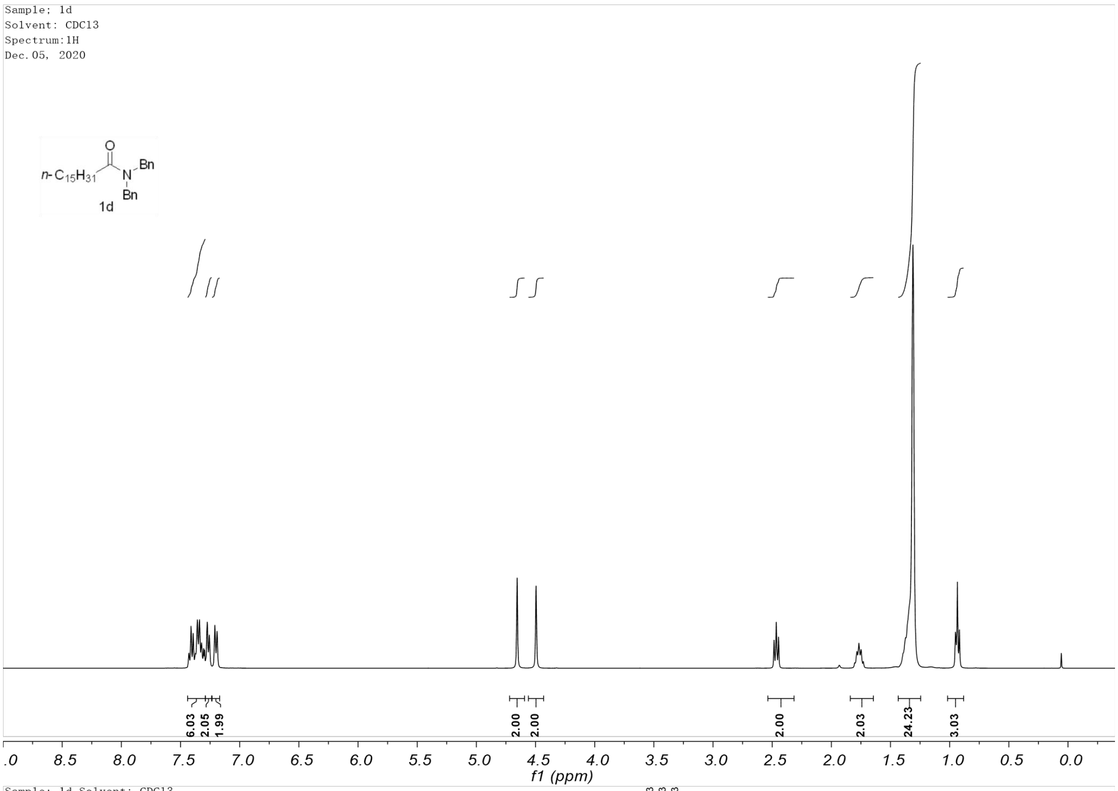

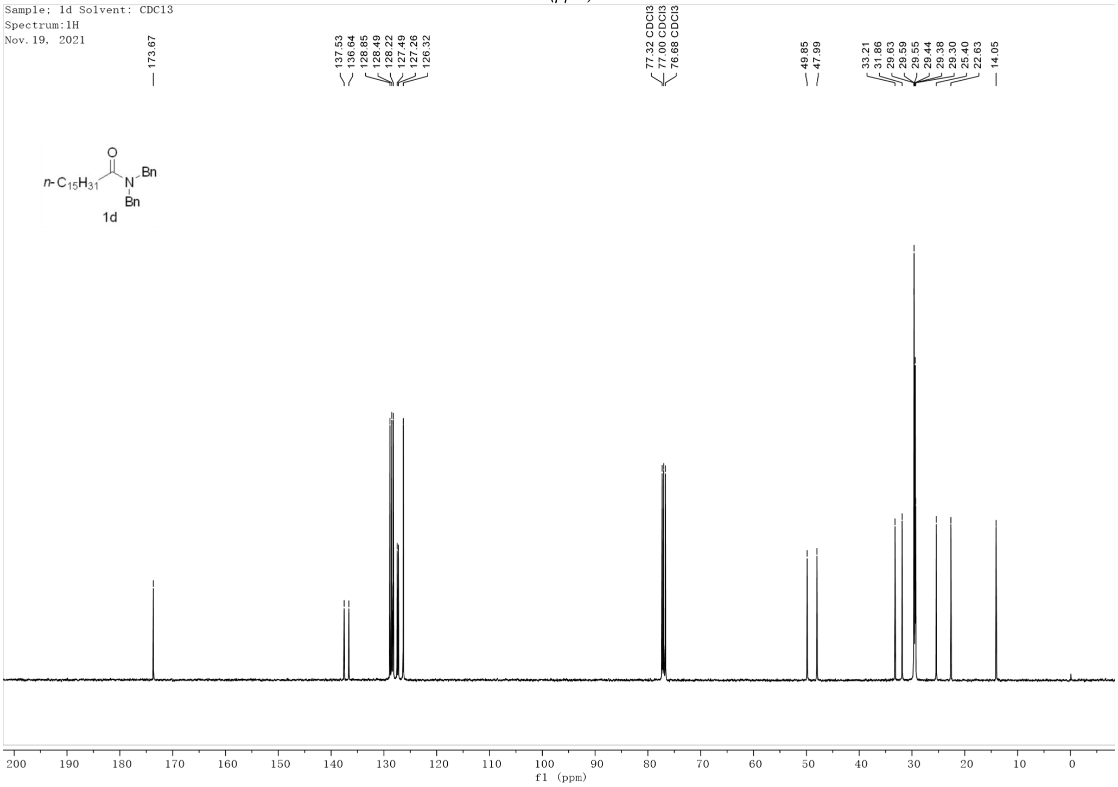

Supplementary Fig. 34. ^{1}H NMR (400 MHz, $CDCl_3$) and ^{13}C NMR (100 MHz, $CDCl_3$) spectra of 1h

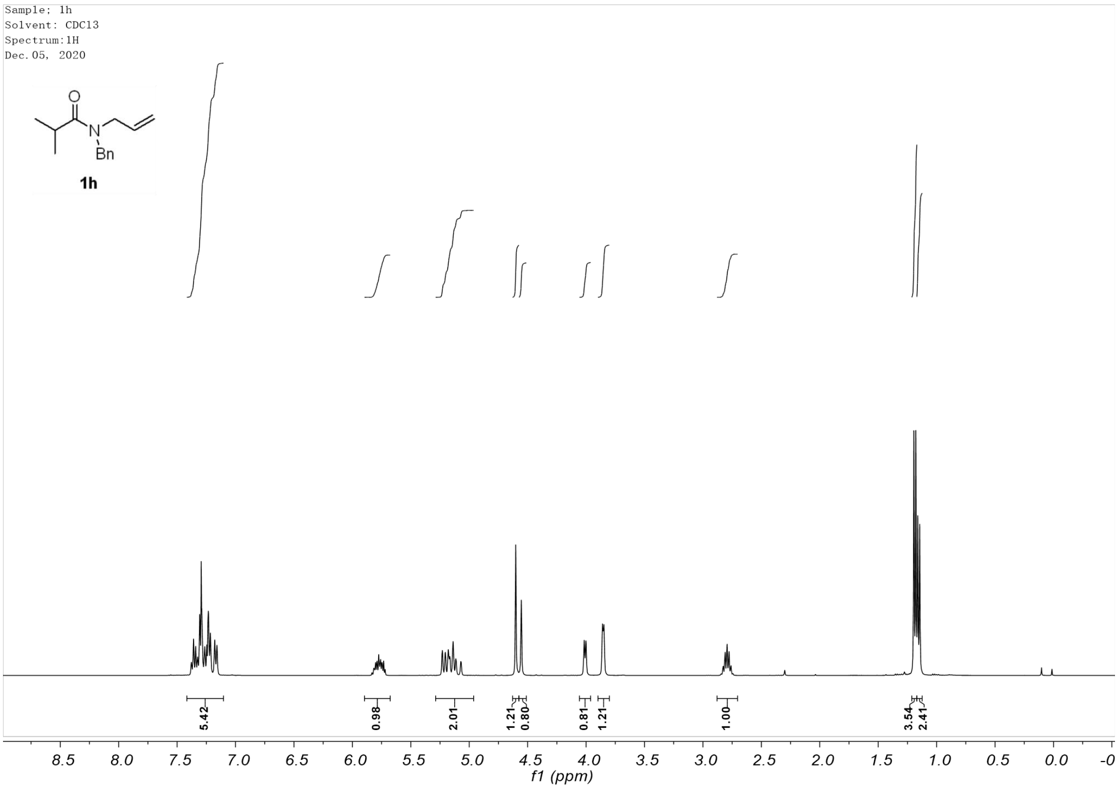

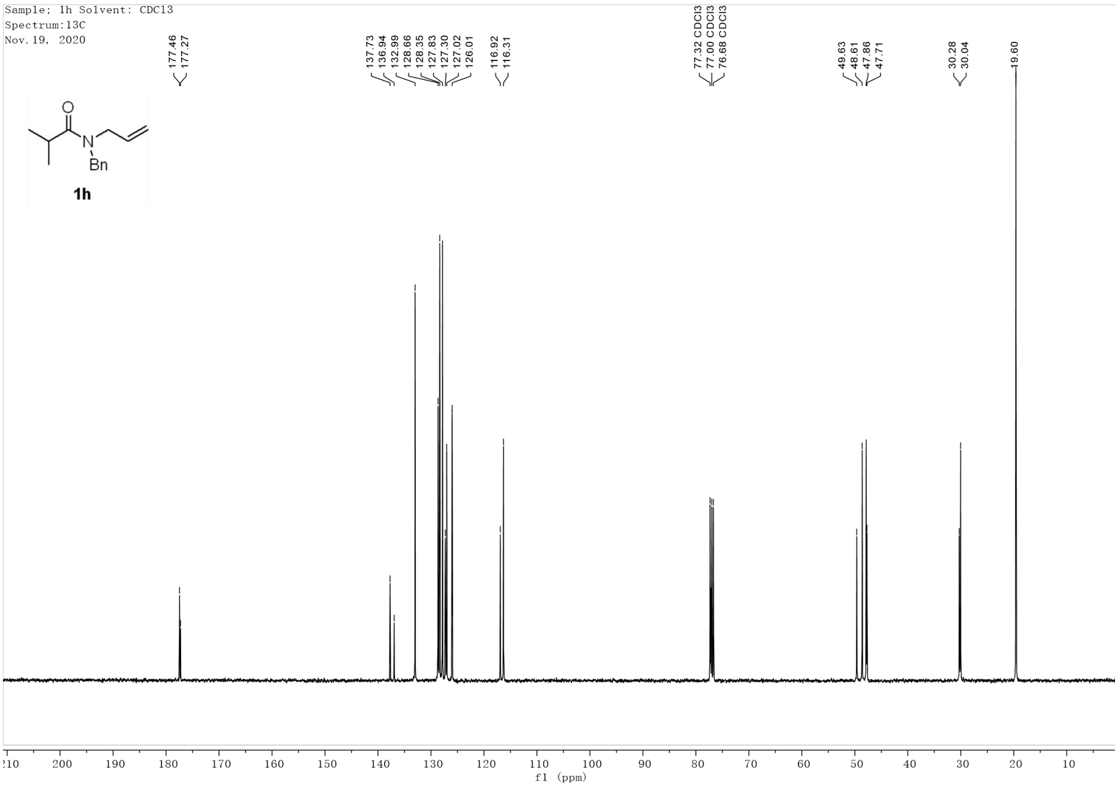

Supplementary Fig. 35. ^{1}H NMR (400 MHz, $CDCl_3$) and ^{13}C NMR (100 MHz, $CDCl_3$) spectra of 1i

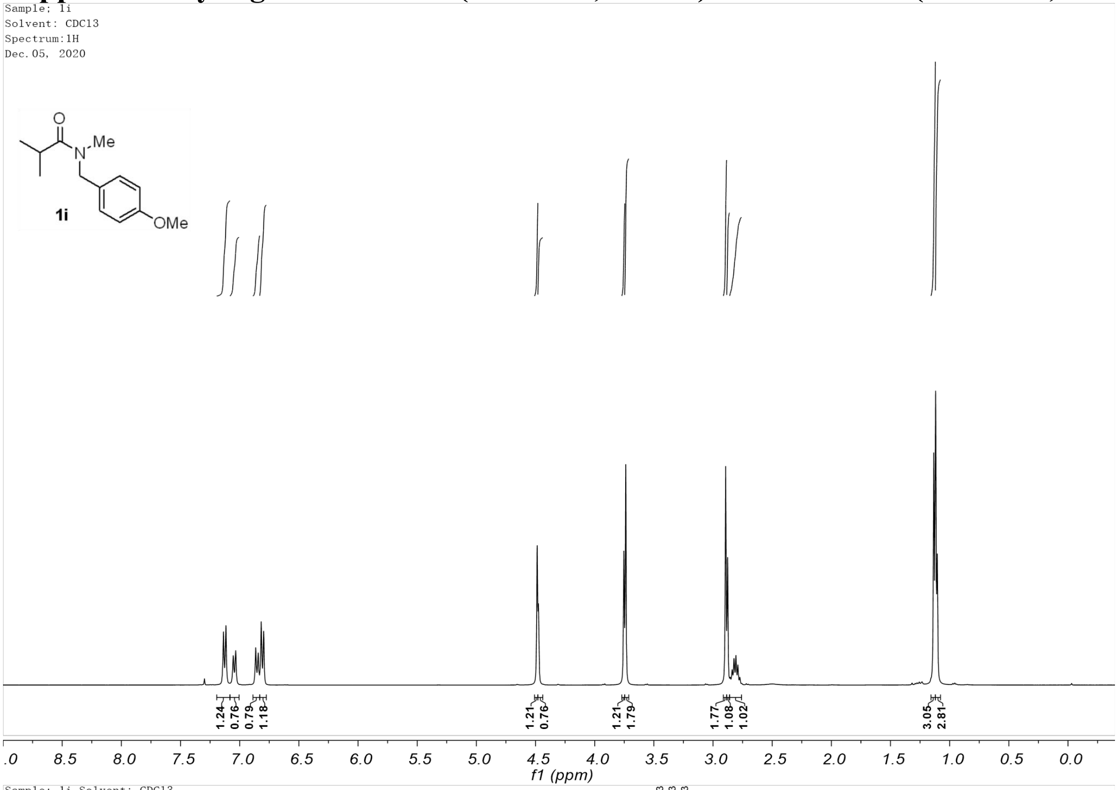

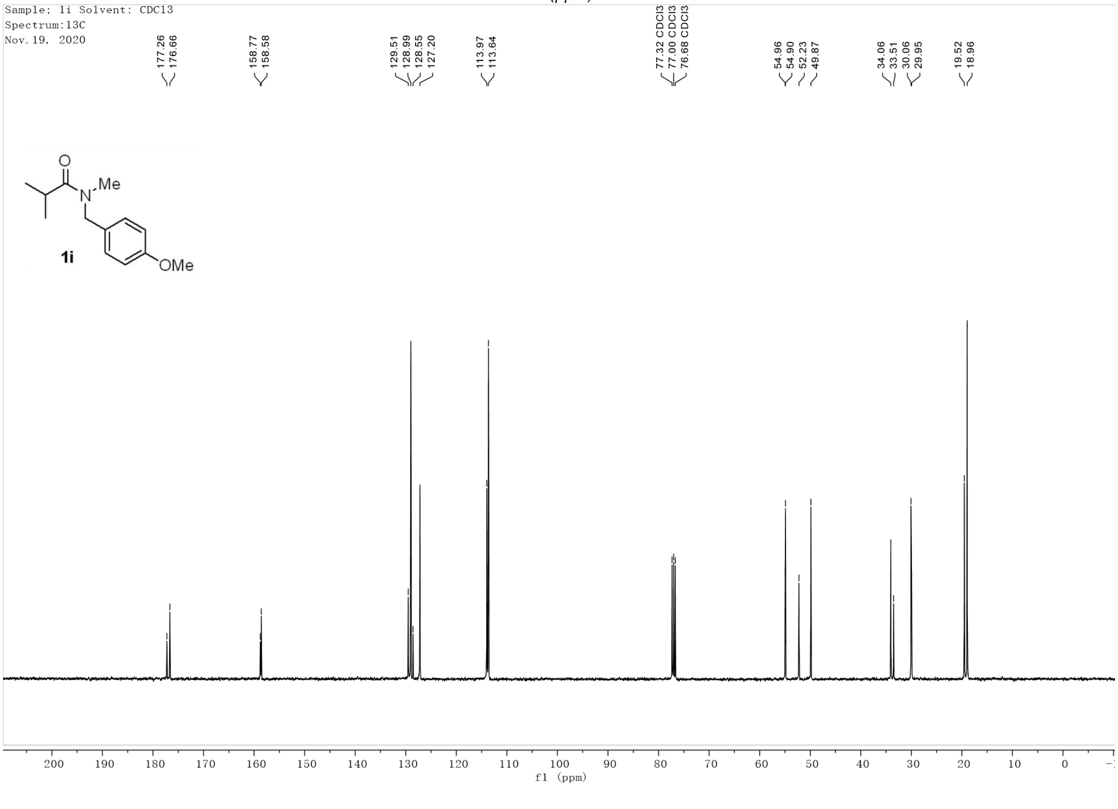

Supplementary Fig. 36. ^{1}H NMR (400 MHz, $CDCl_3$) and ^{13}C NMR (100 MHz, $CDCl_3$) spectra of 1l

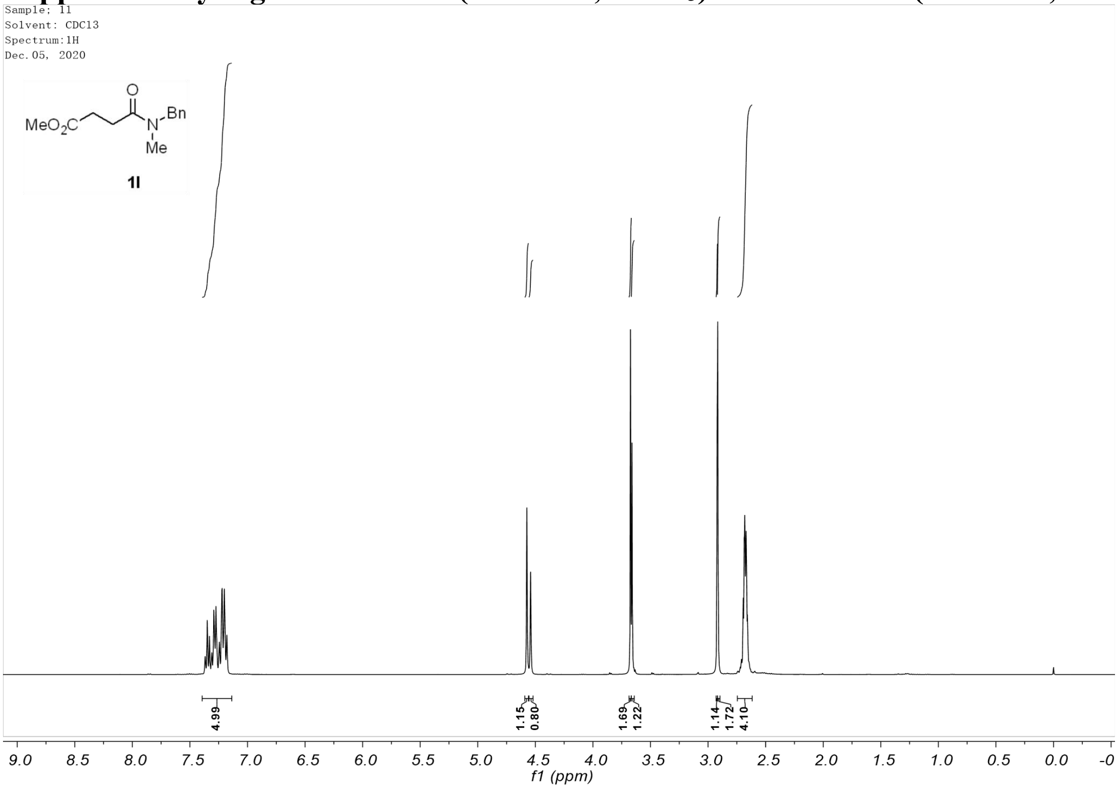

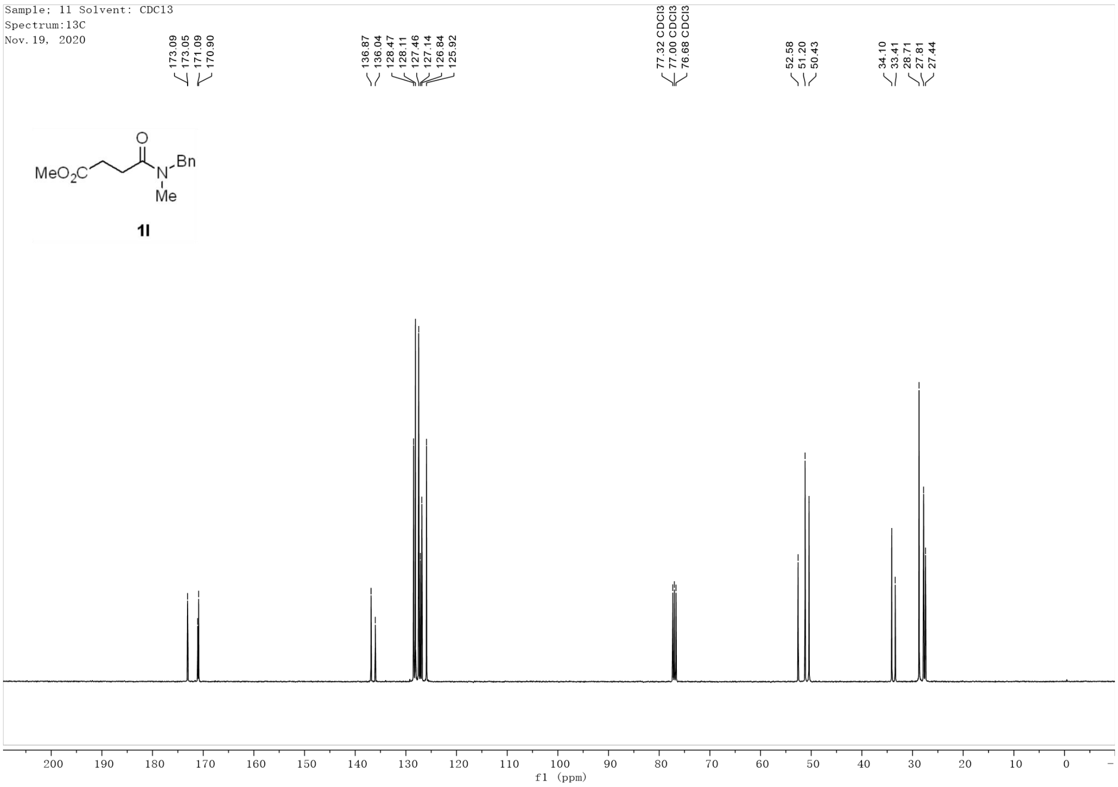

Supplementary Fig. 37. ^{1}H NMR (400 MHz, $CDCl_3$) and ^{13}C NMR (100 MHz, $CDCl_3$) spectra of 1m

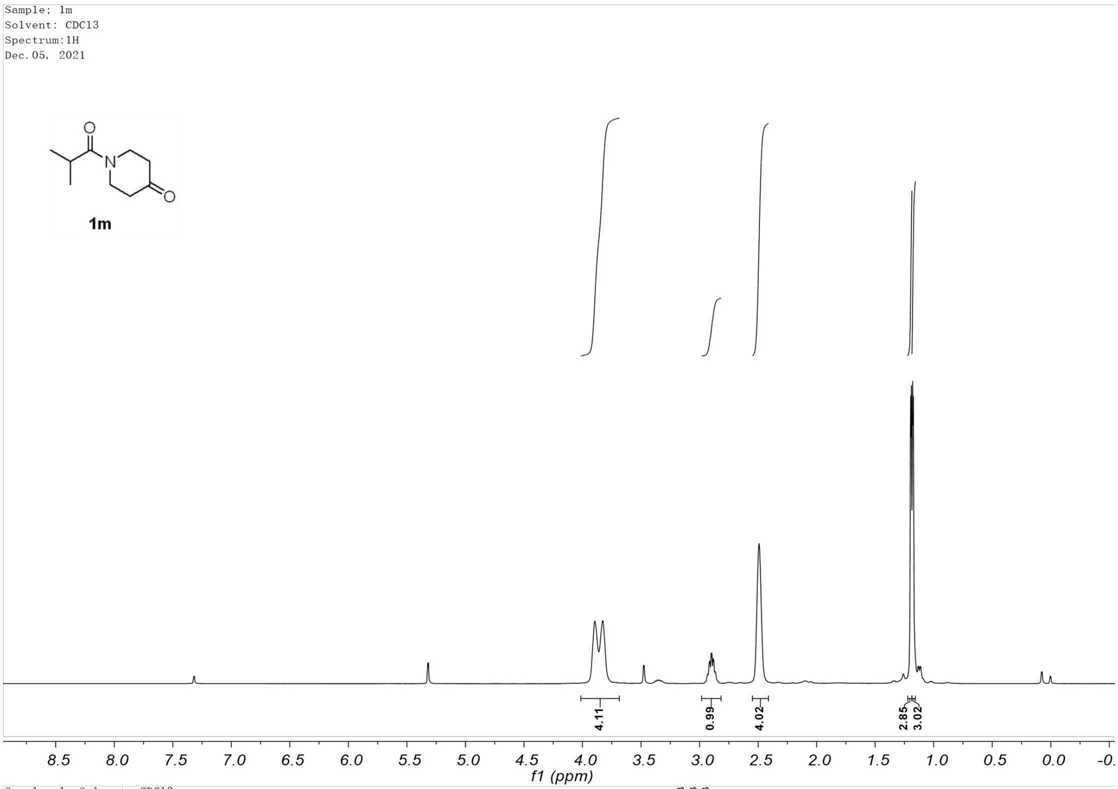

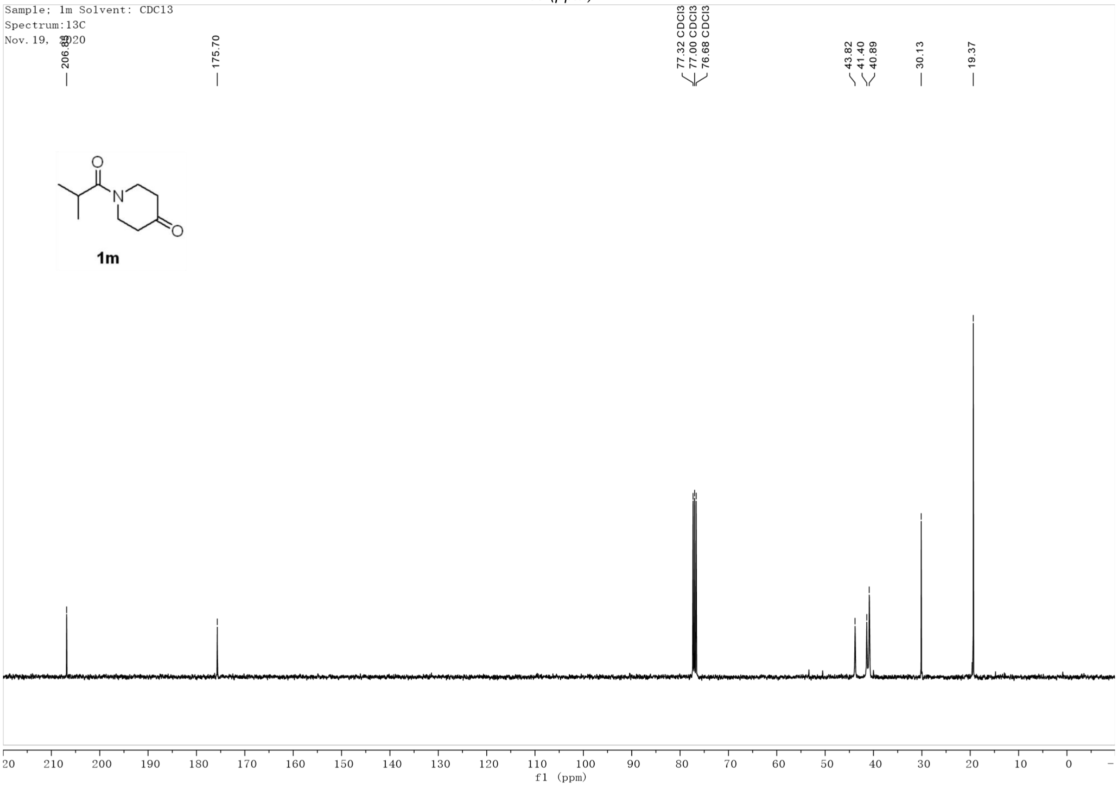

Supplementary Fig. 38. ^{1}H **NMR (400 MHz, CDCl$_3$),** ^{13}C **NMR (100 MHz, CDCl$_3$) and** ^{19}F **NMR (376 MHz, CDCl$_3$) spectra of 1o**

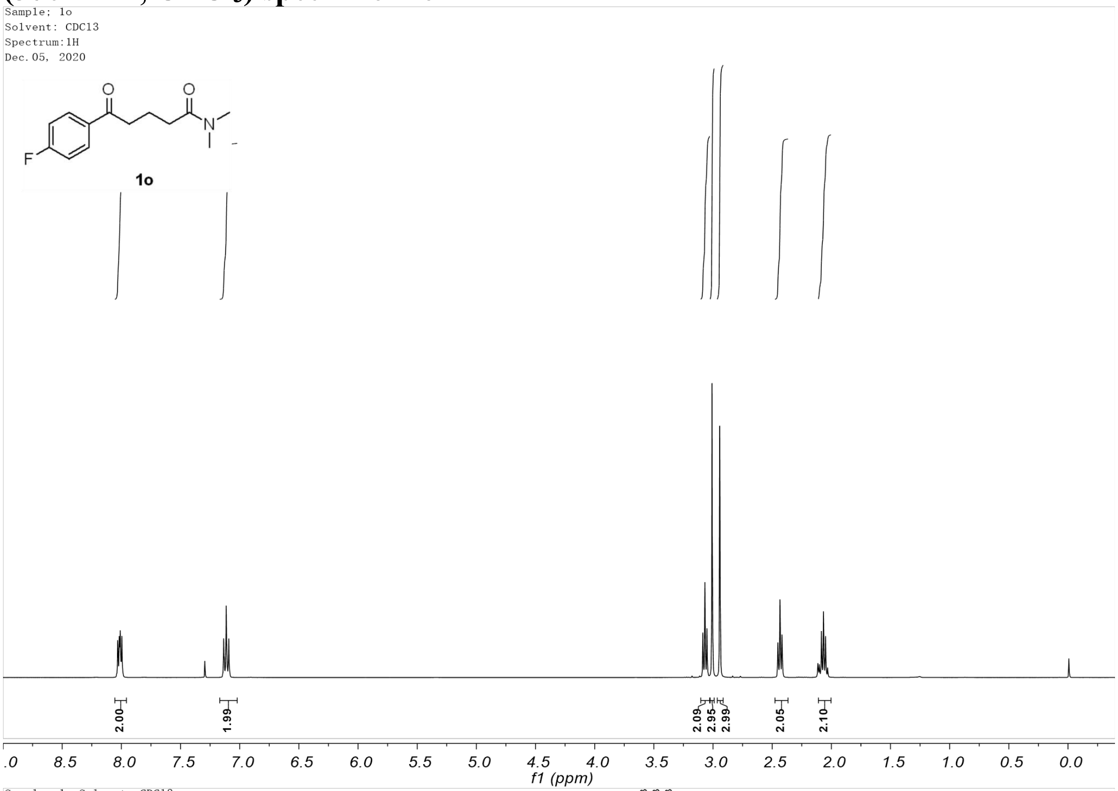

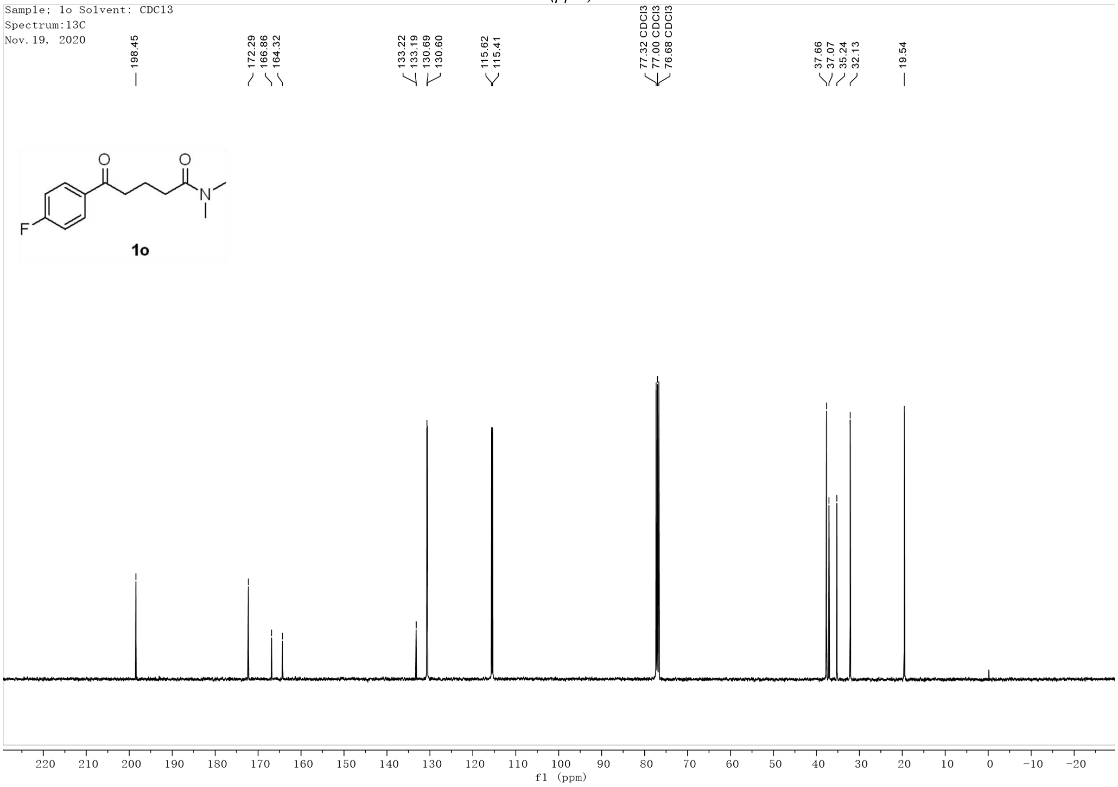

Sample: 1o Solvent: CDCl3
Spectrum:F
Aug. 18, 2021

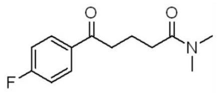

1o

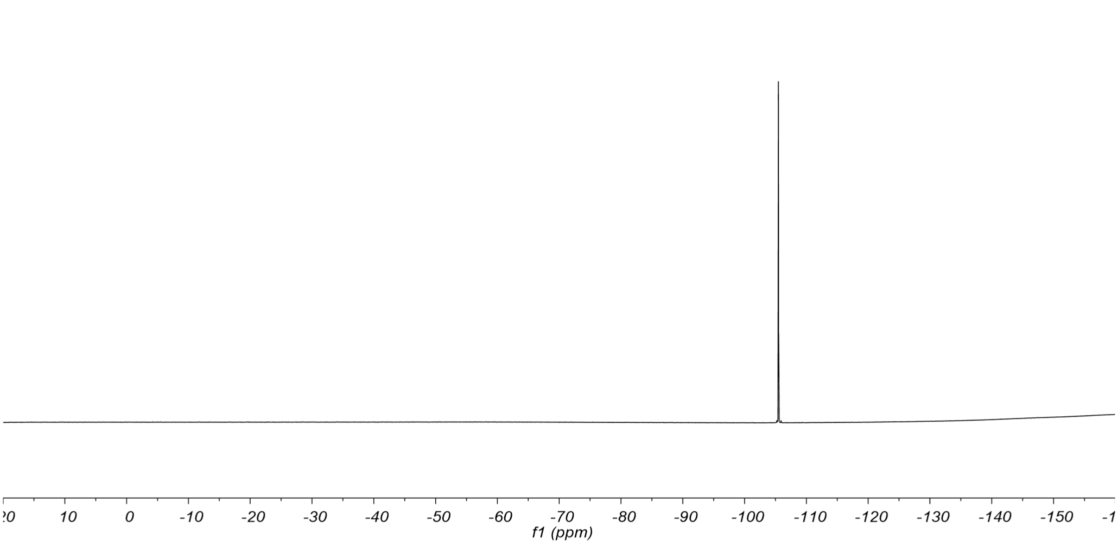

Supplementary Fig. 39. ^{1}H **NMR (400 MHz,** $CDCl_3$**) and** ^{13}C **NMR (100 MHz,** $CDCl_3$**) spectra of 1s**

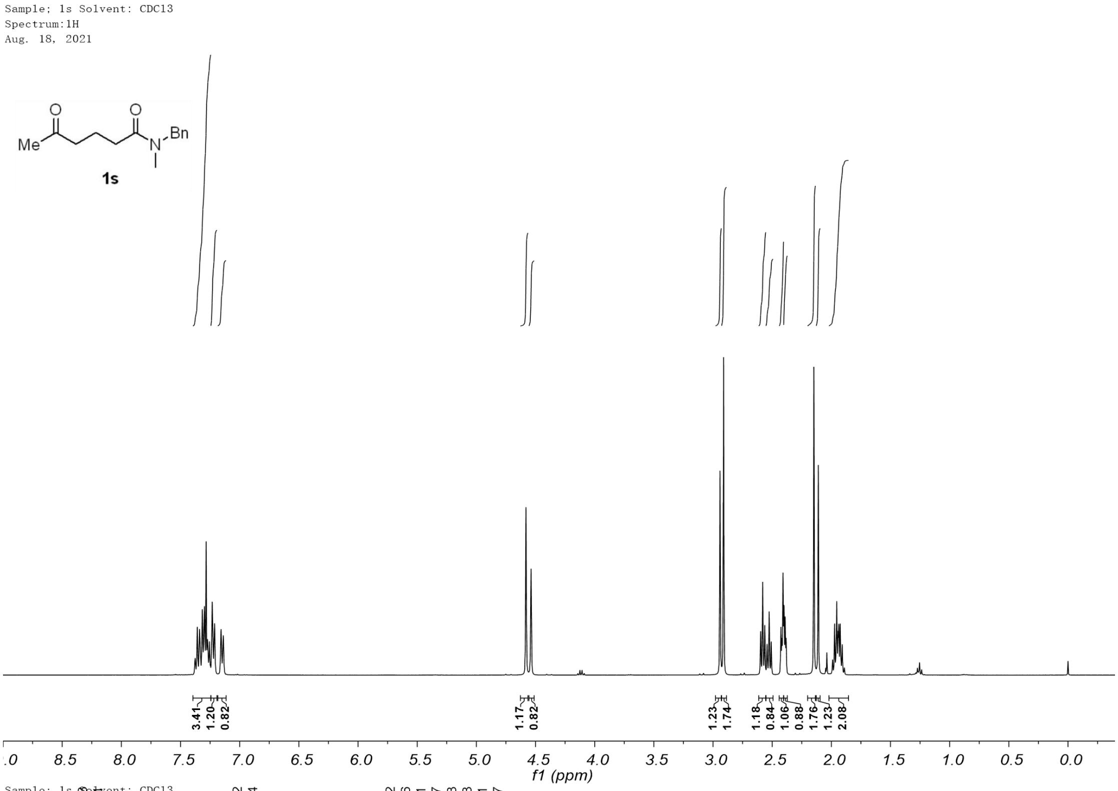

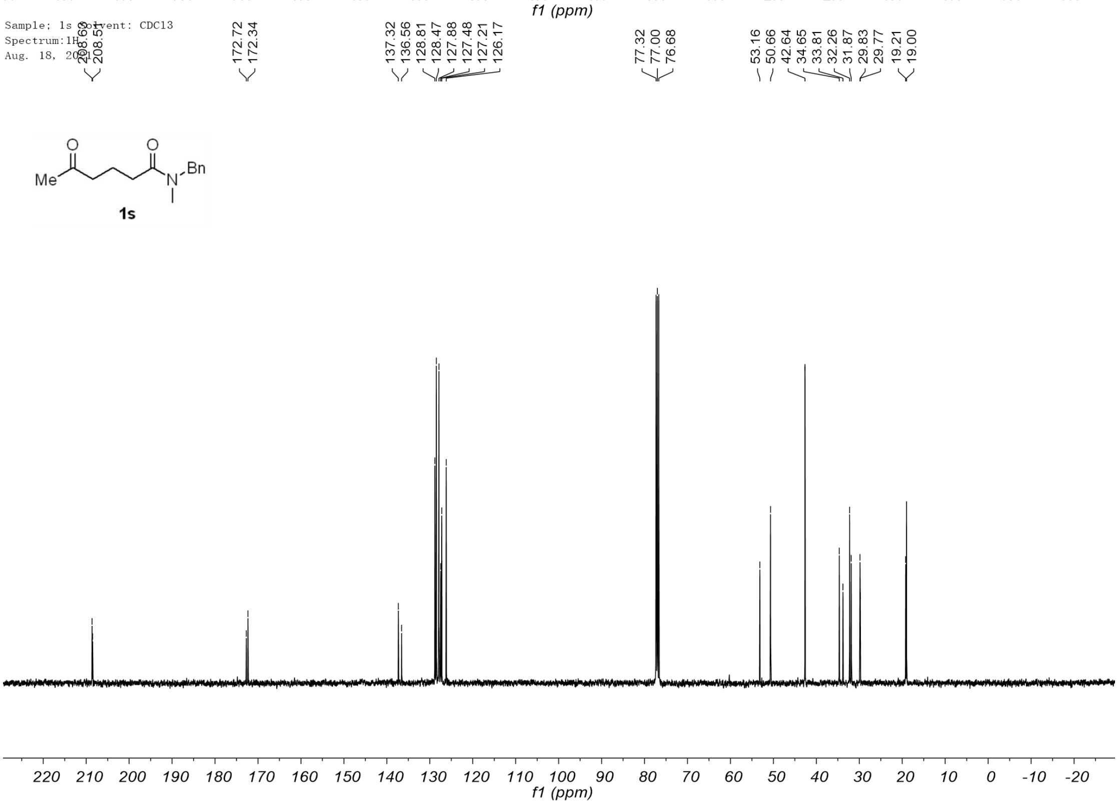

Supplementary Fig. 40. **^{1}H NMR (400 MHz, $CDCl_3$) and ^{13}C NMR (100 MHz, $CDCl_3$) spectra of 1t**

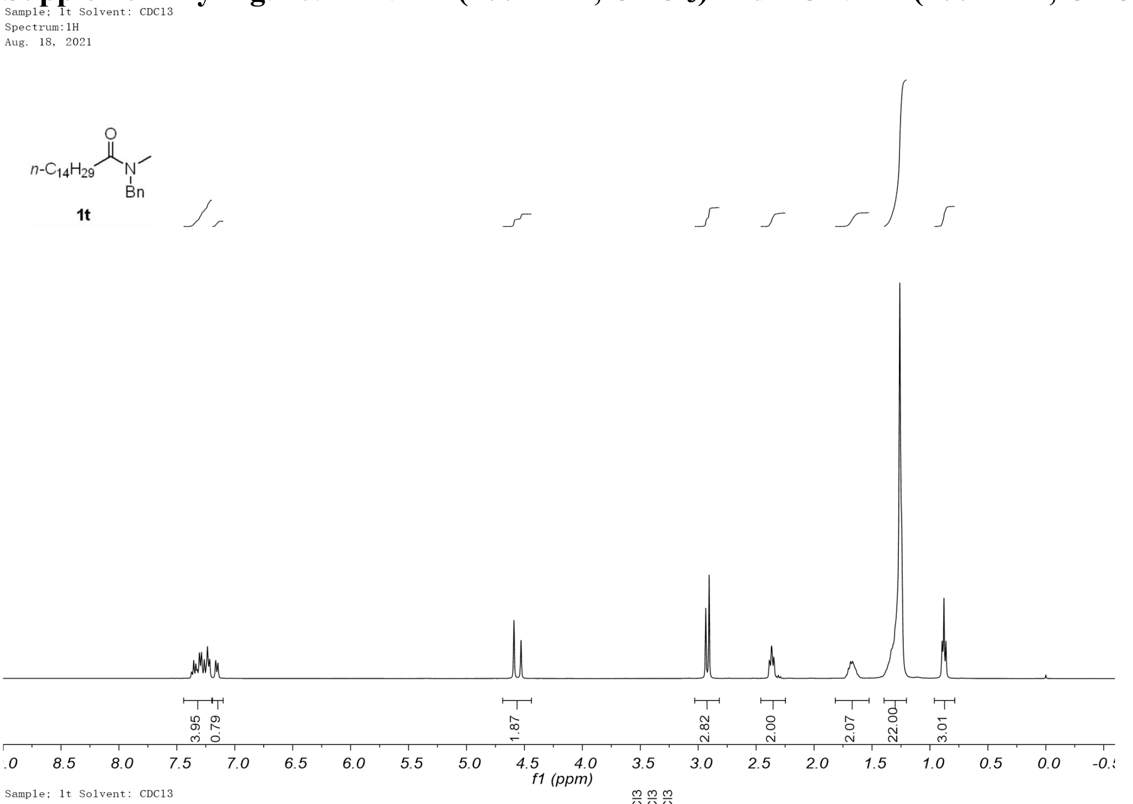

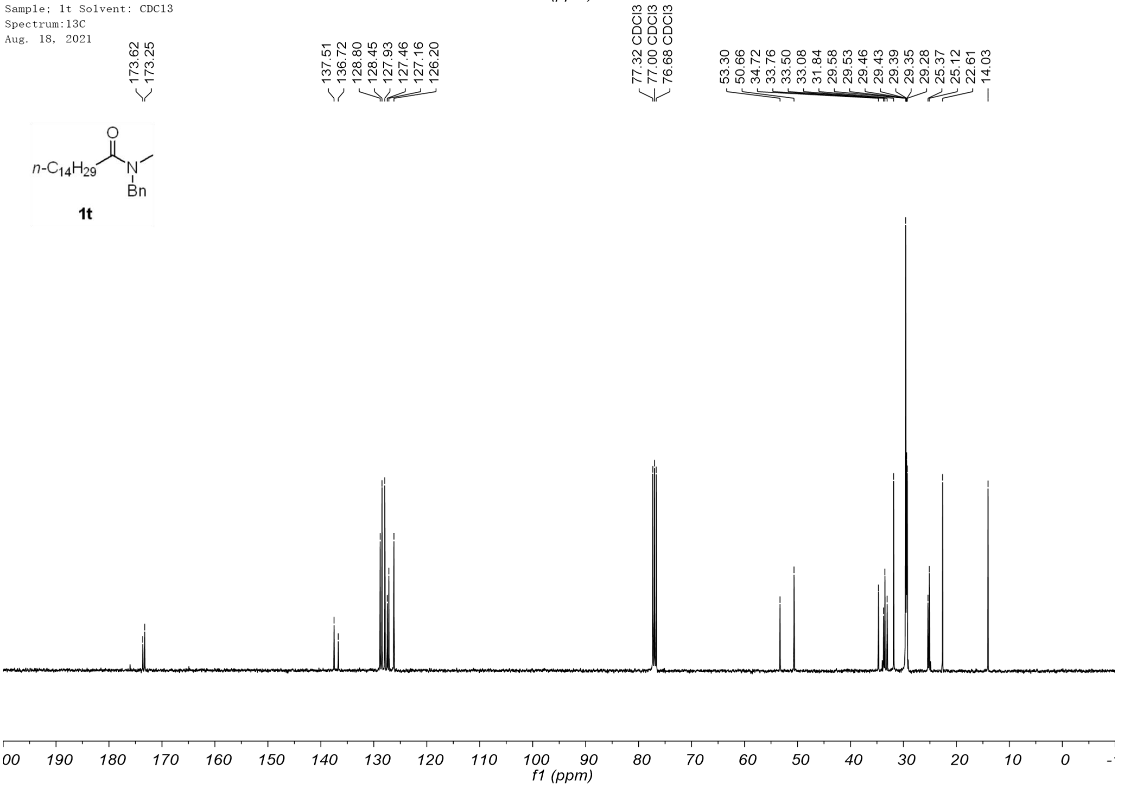

Supplementary Fig. 41. ¹H NMR (400 MHz, $CDCl_3$) and ¹³C NMR (100 MHz, $CDCl_3$) spectra of 1q

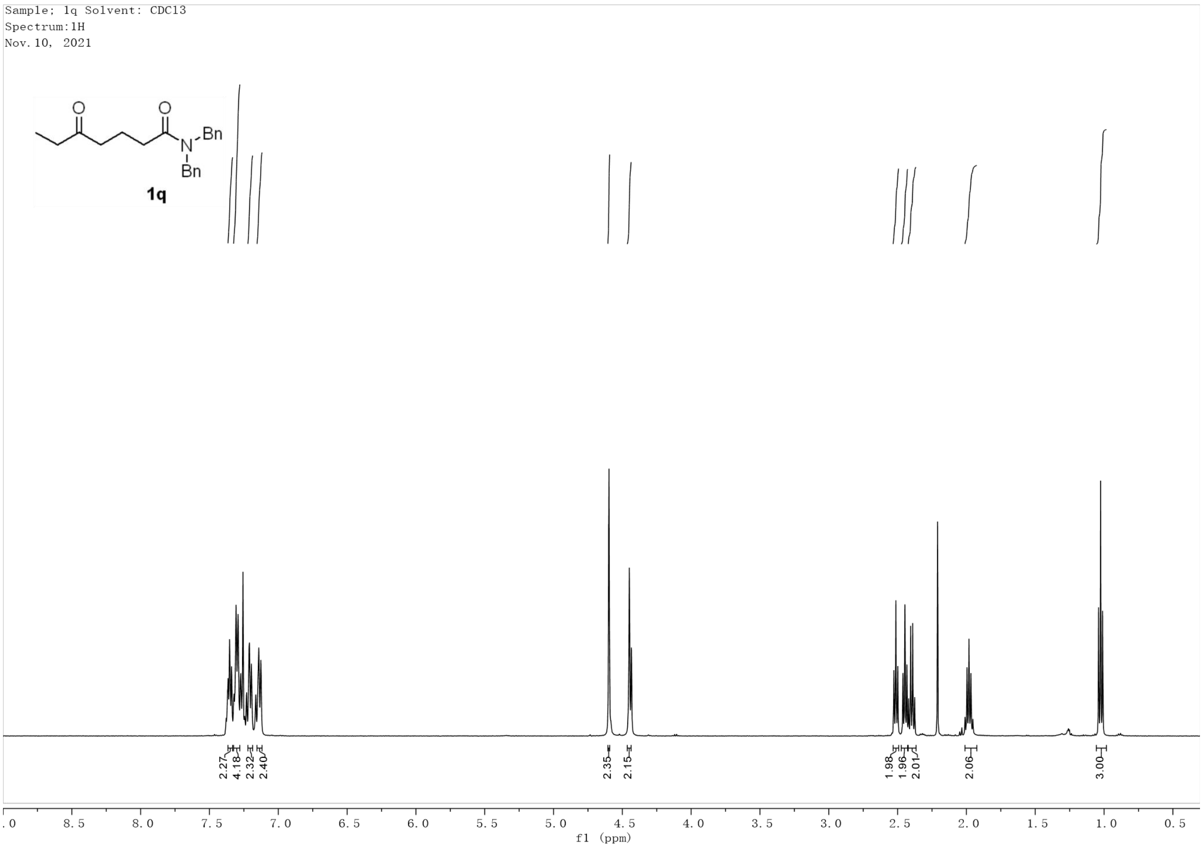

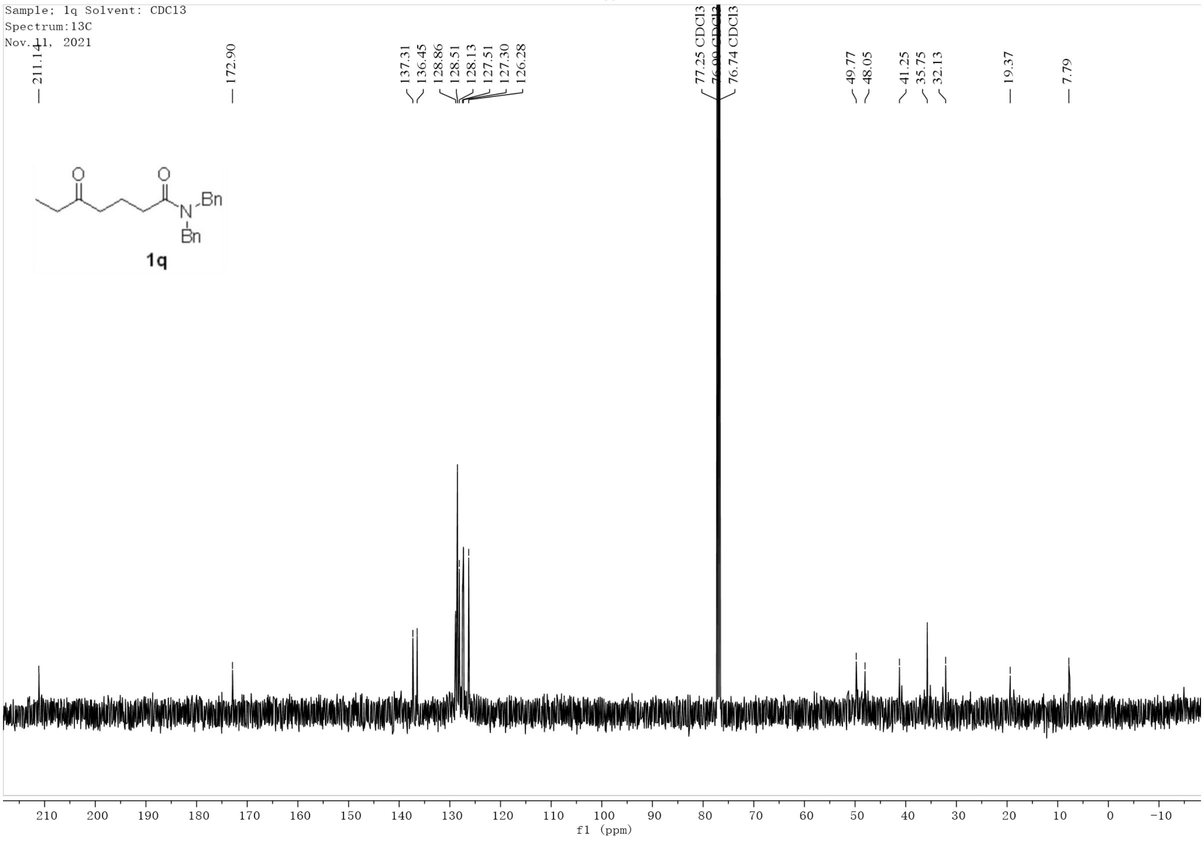

Supplementary Fig. 42. ^{1}H NMR (400 MHz, $CDCl_3$) and ^{13}C NMR (100 MHz, $CDCl_3$) spectra of 1r

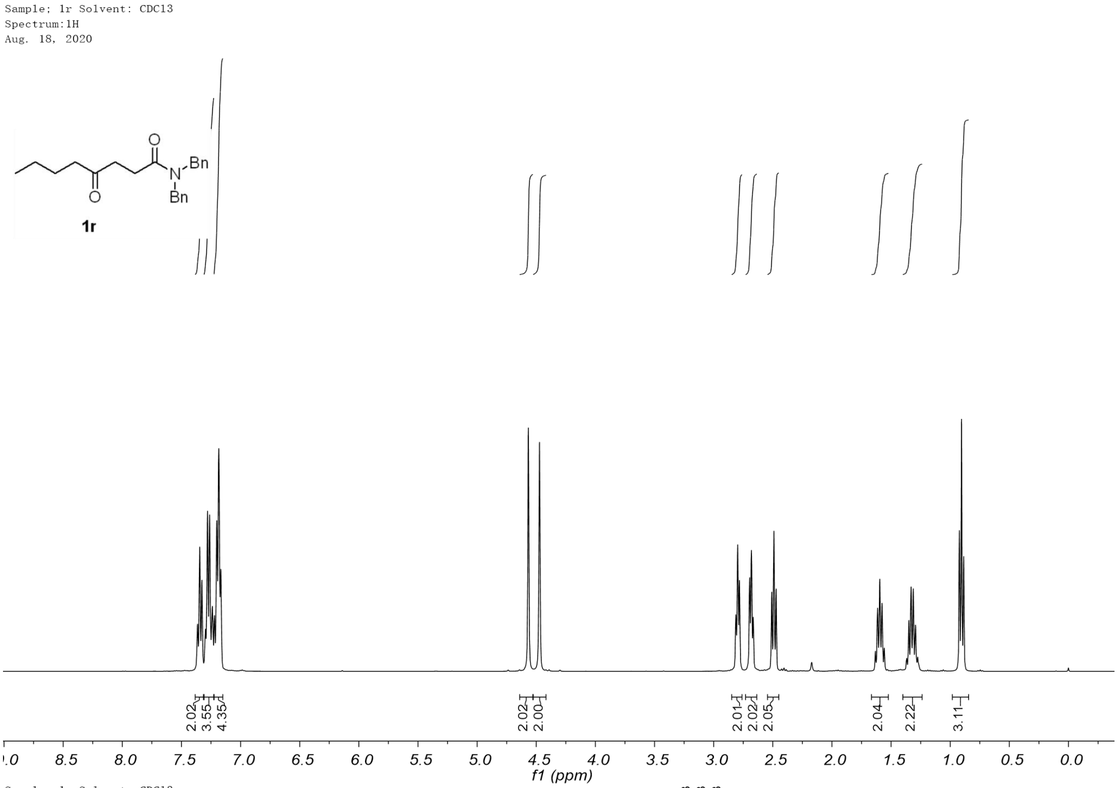

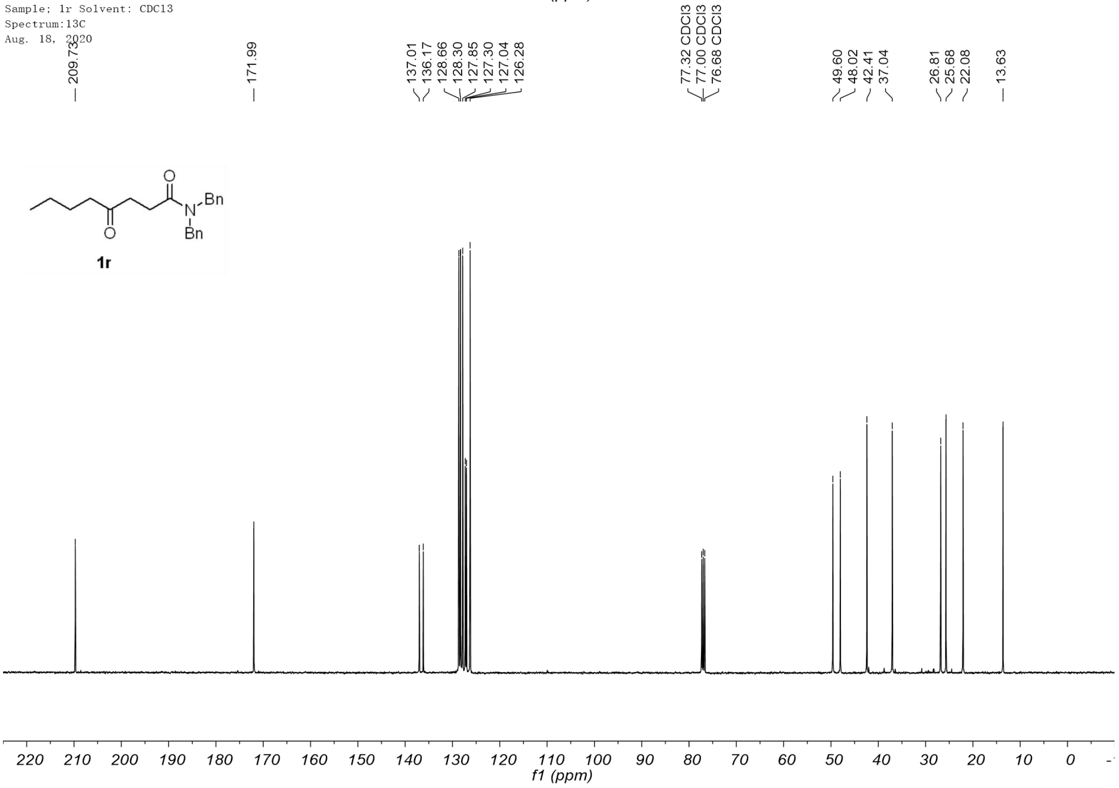

Supplementary Fig. 43. ^{1}H NMR (400 MHz, $CDCl_3$) and ^{13}C NMR (100 MHz, $CDCl_3$) spectra of 1u

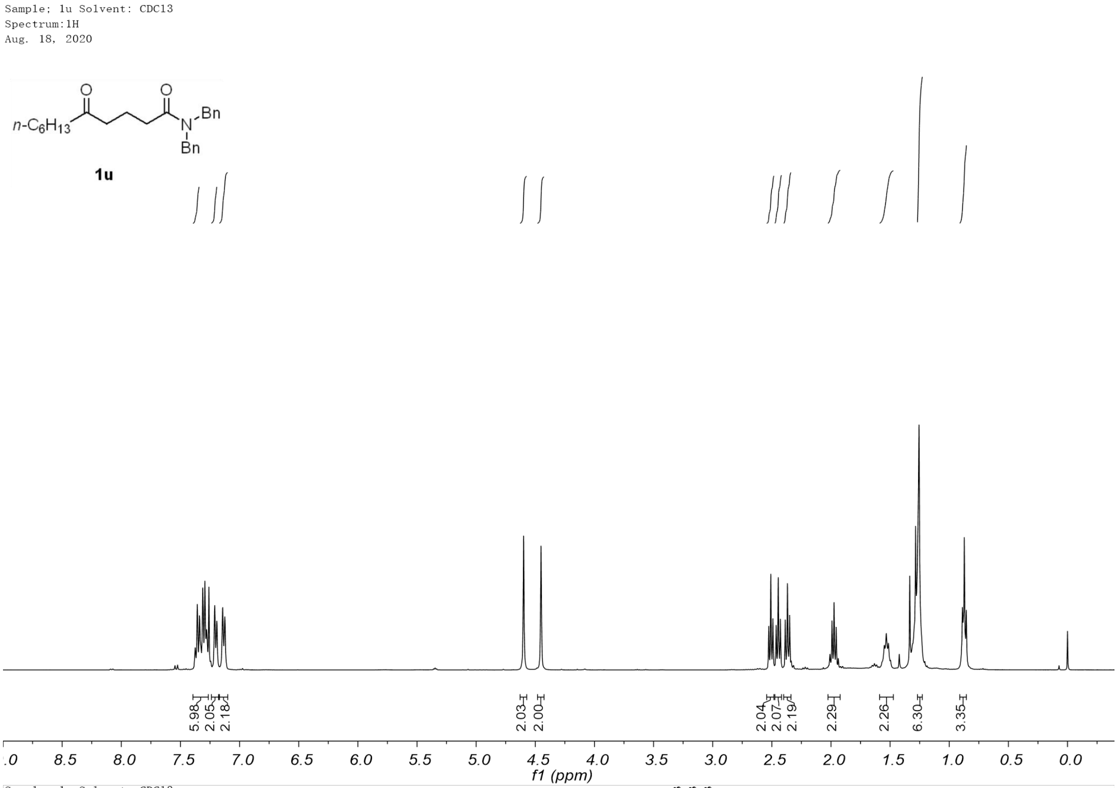

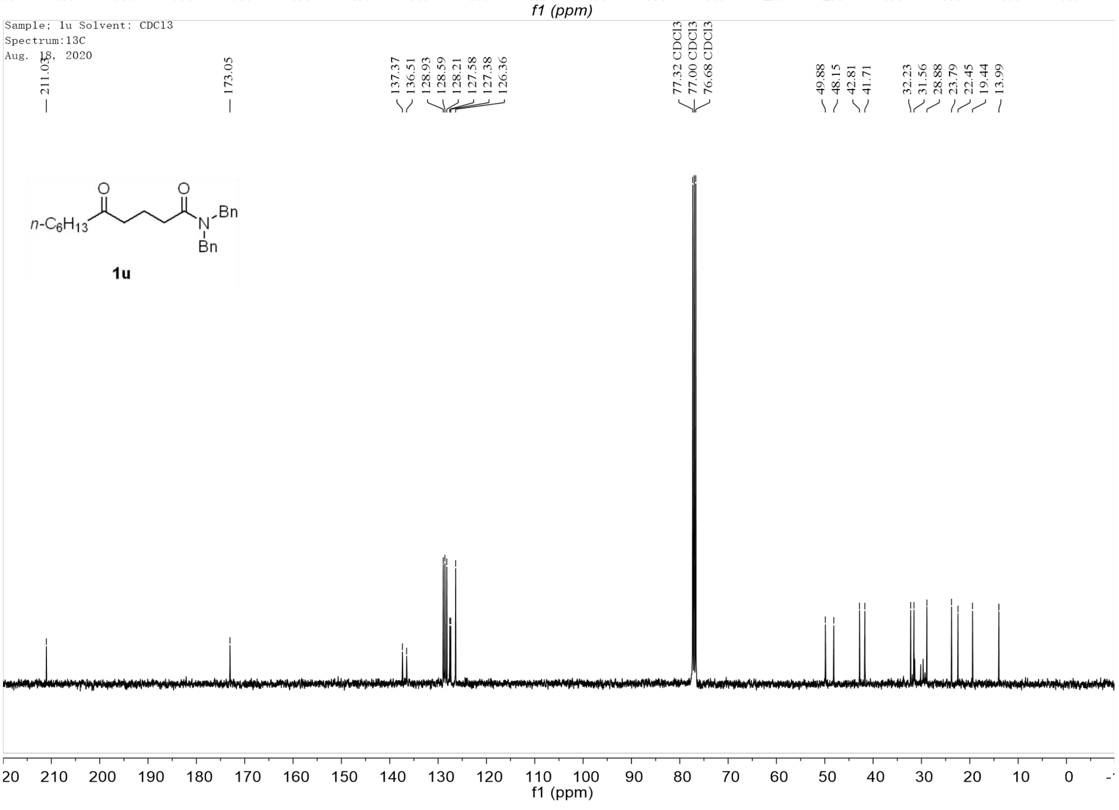

Supplementary Fig. 44. ¹H NMR (400 MHz, $CDCl_3$) and ¹³C NMR (100 MHz, $CDCl_3$) spectra of 2l

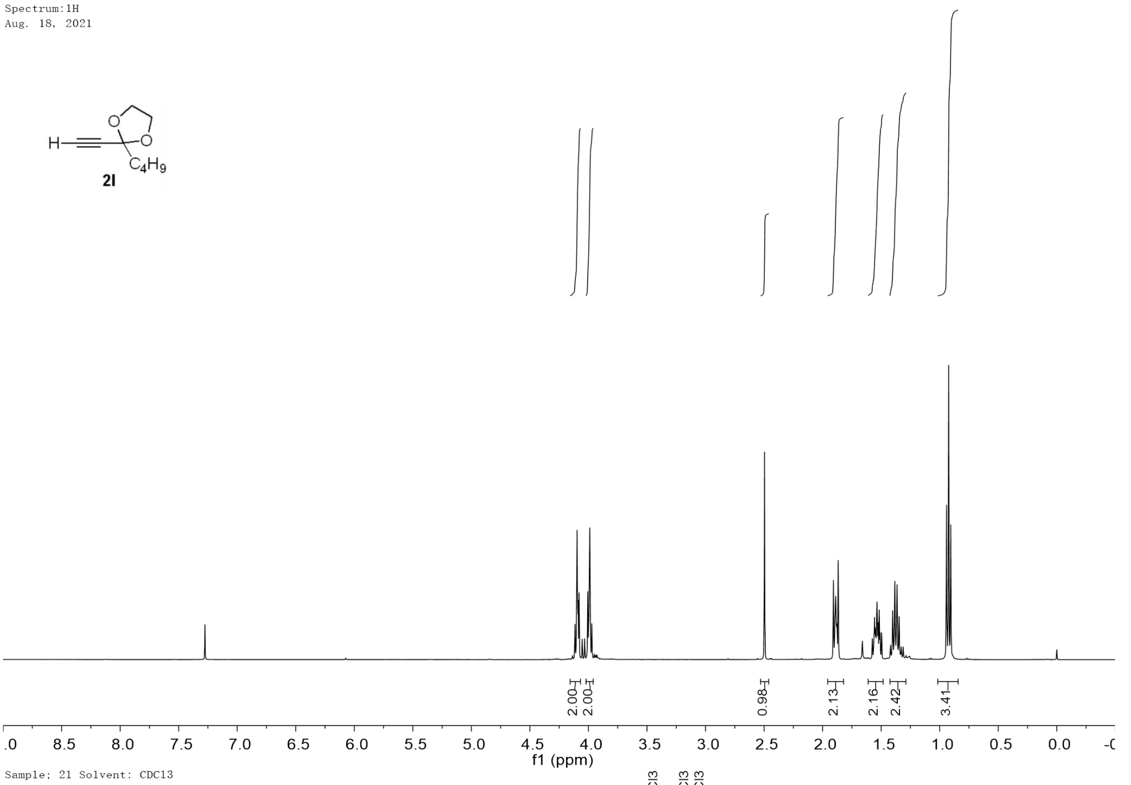

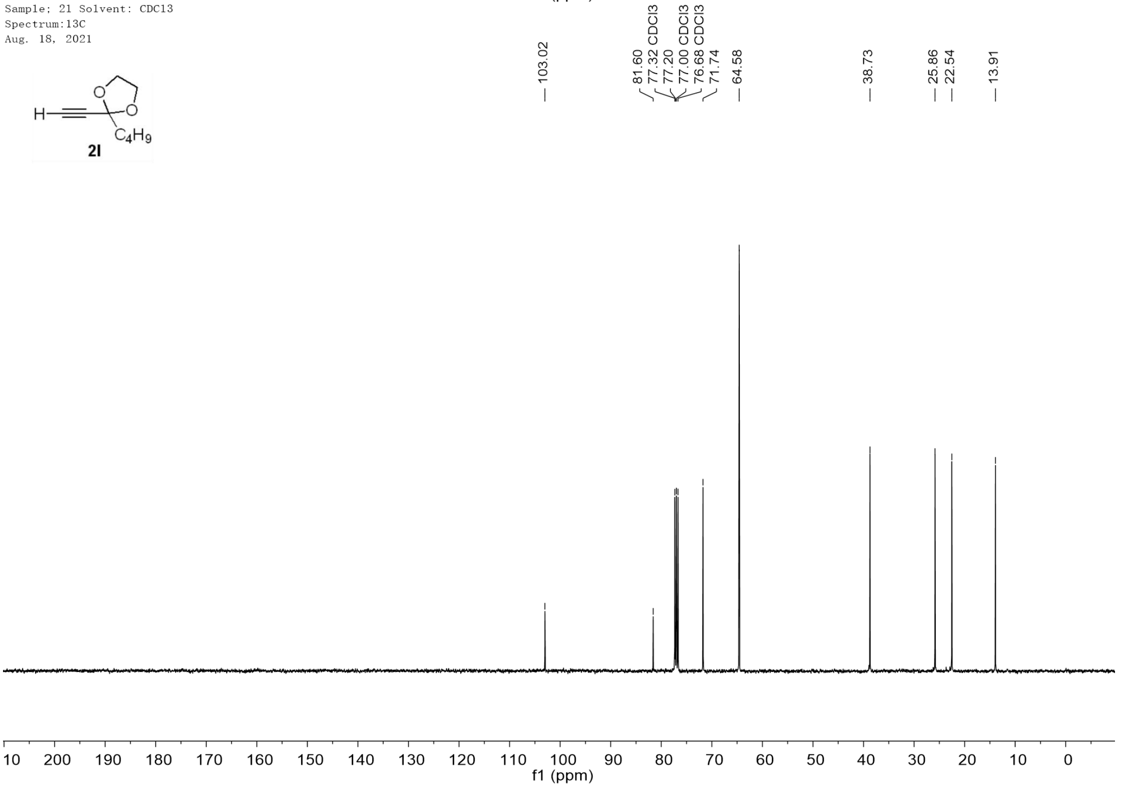

Supplementary Fig. 45. ^{1}H NMR (500 MHz, $CDCl_3$) and ^{13}C NMR (125 MHz, $CDCl_3$) spectra of 3a

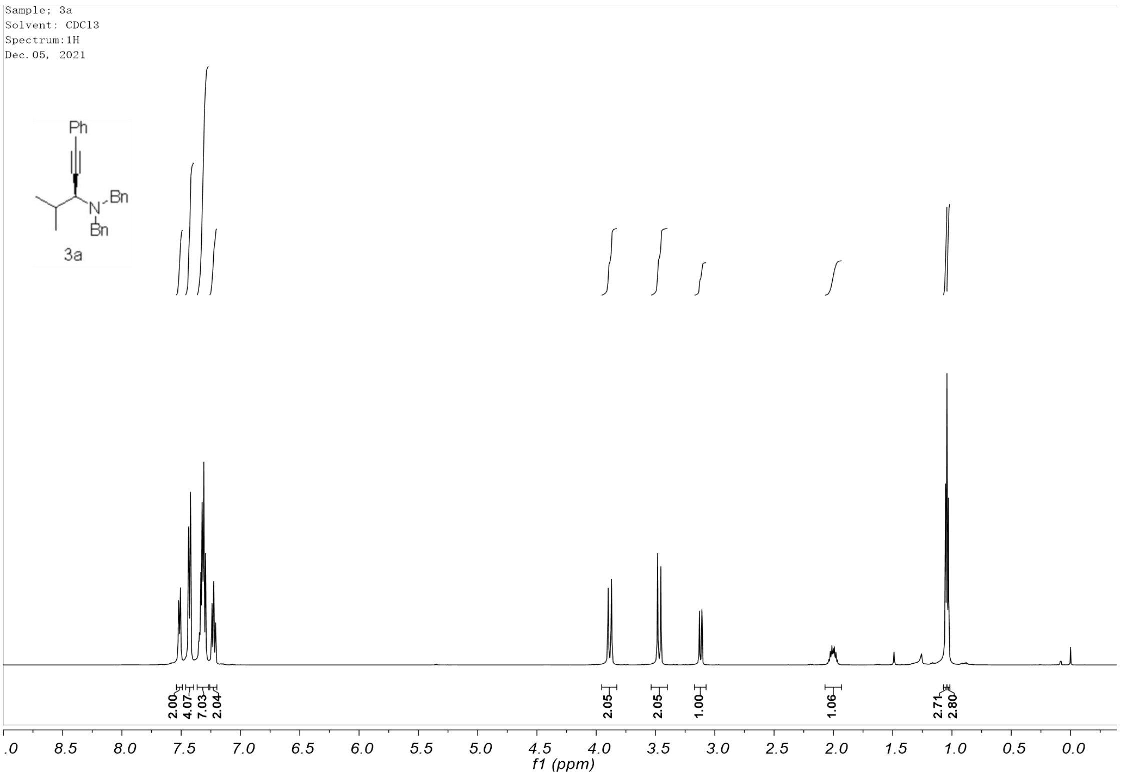

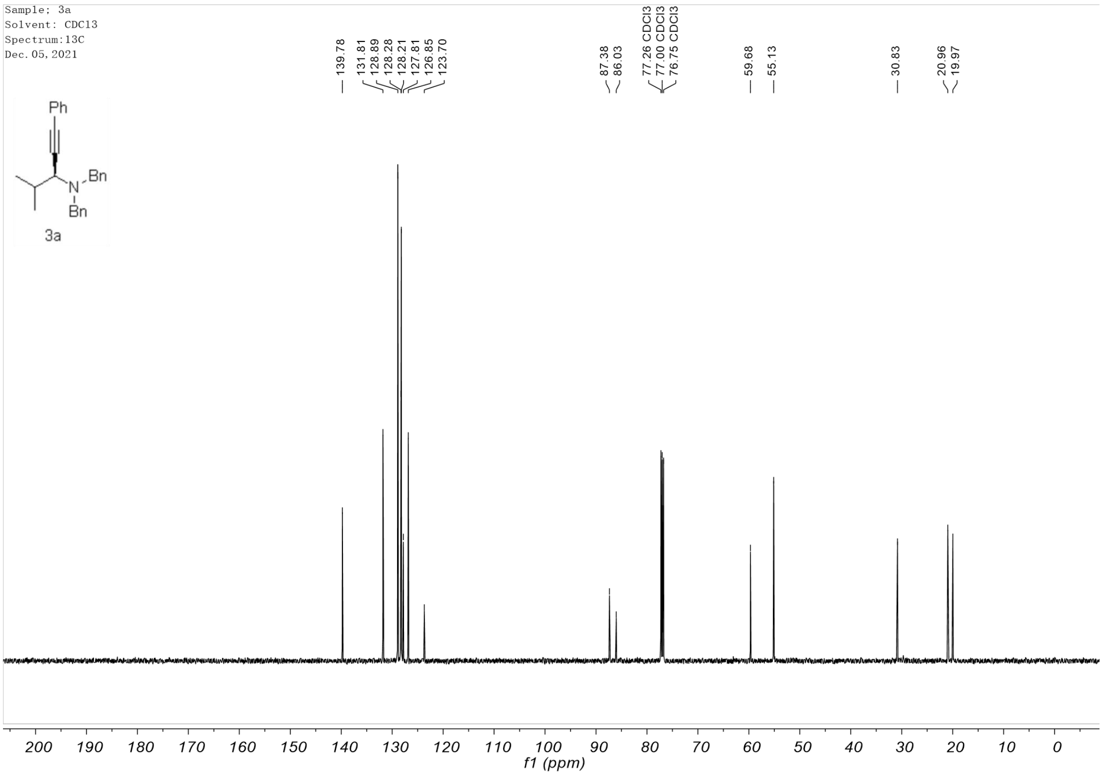

Supplementary Fig. 46. ^{1}H NMR (500 MHz, $CDCl_3$) and ^{13}C NMR (126 MHz, $CDCl_3$) spectra of 3b

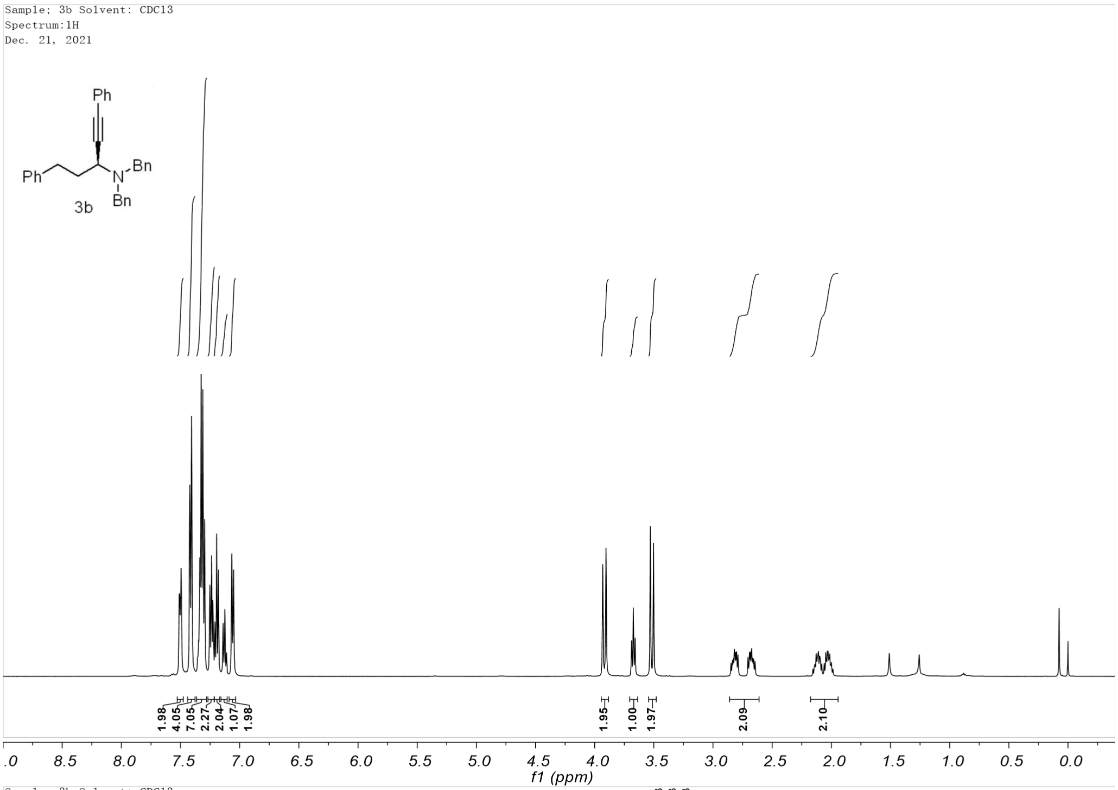

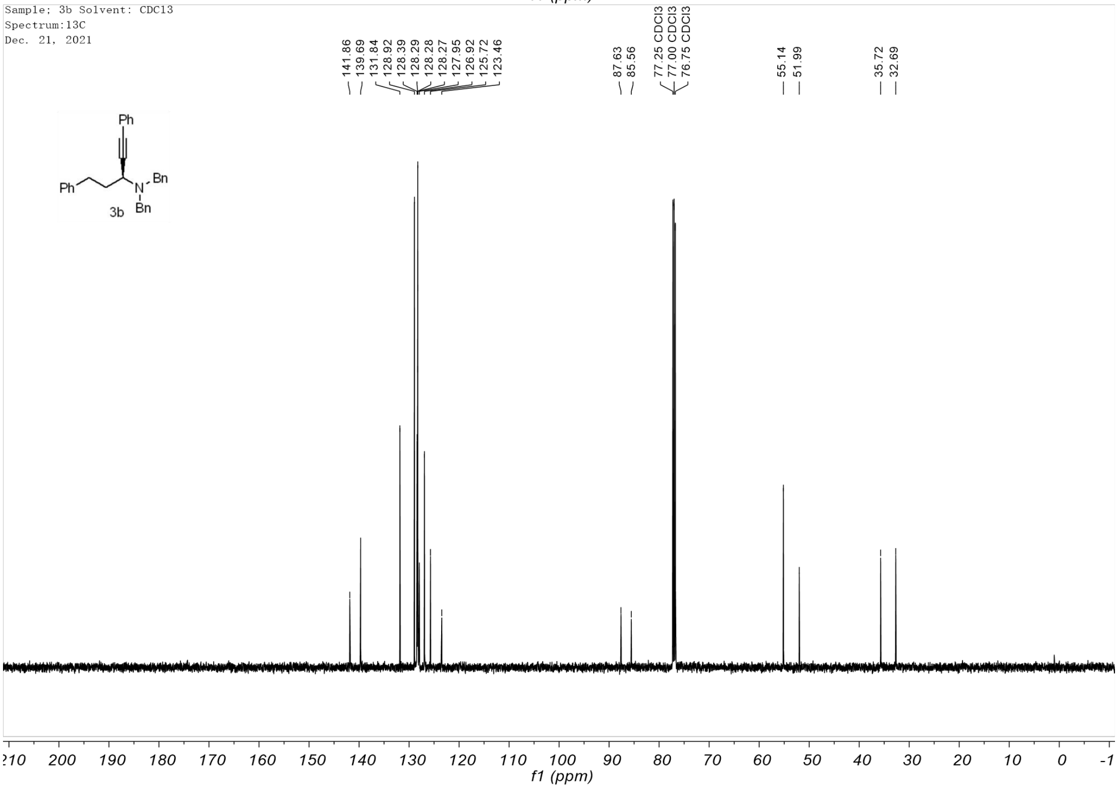

Supplementary Fig. 47. ^{1}H NMR (500 MHz, $CDCl_3$) and ^{13}C NMR (126 MHz, $CDCl_3$) spectra of 3c

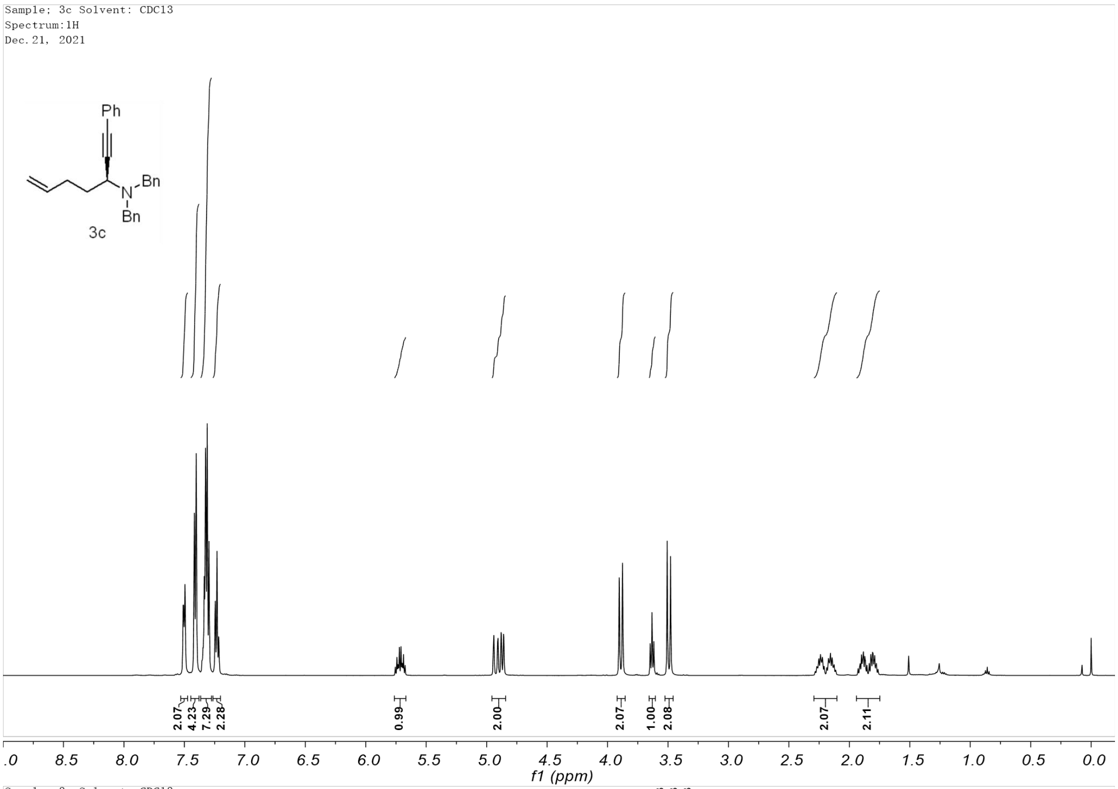

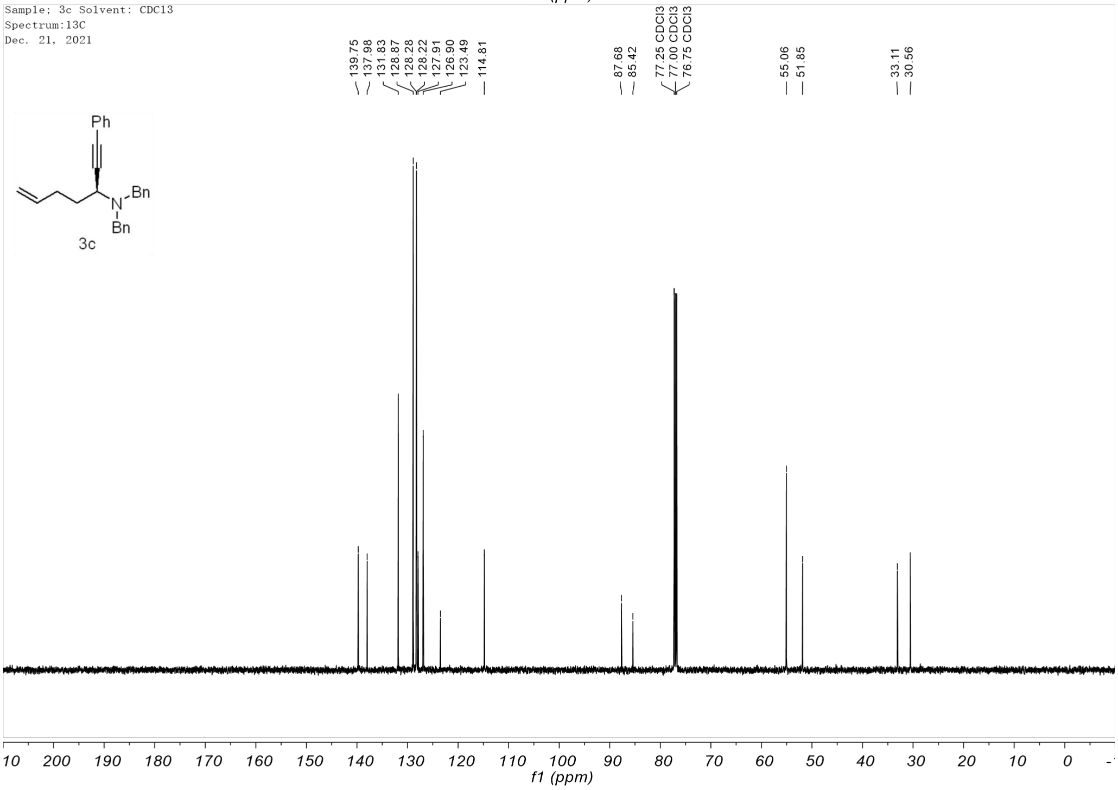

Supplementary Fig. 48. ^{1}H NMR (500 MHz, $CDCl_3$) and ^{13}C NMR (126 MHz, $CDCl_3$) spectra of 3d

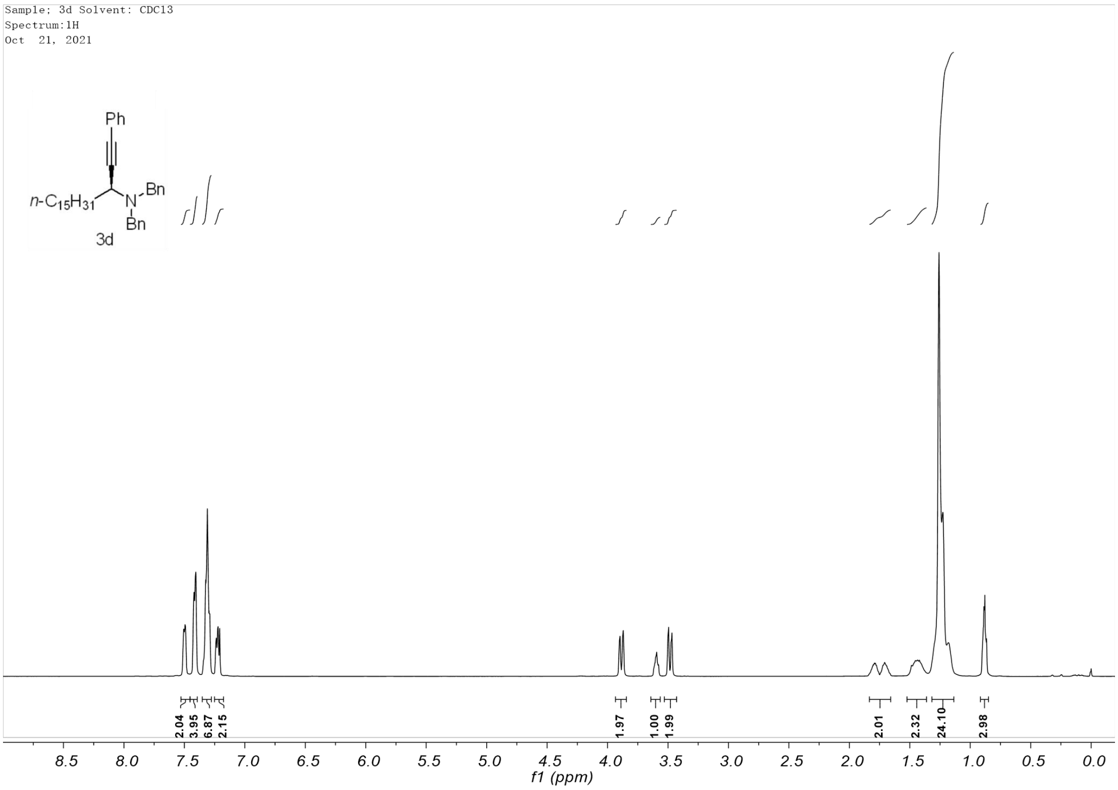

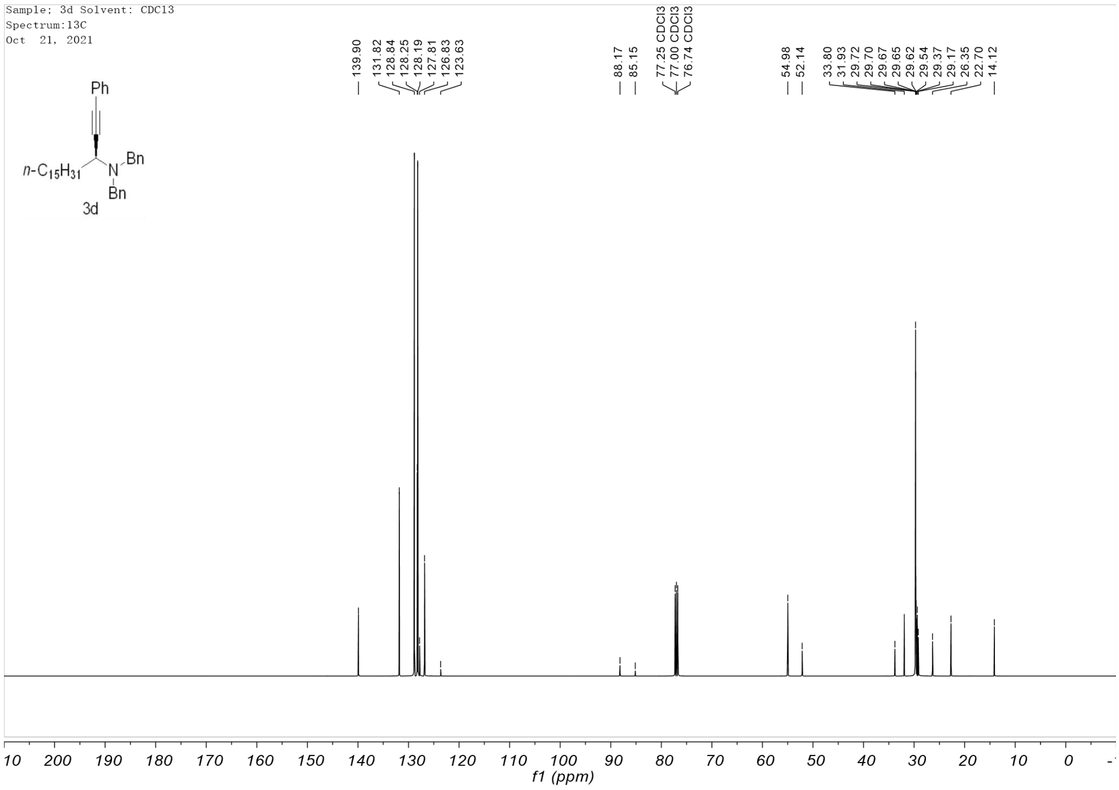

Supplementary Fig. 49. ^{1}H NMR (400 MHz, $CDCl_3$) and ^{13}C NMR (100 MHz, $CDCl_3$) spectra of 3e

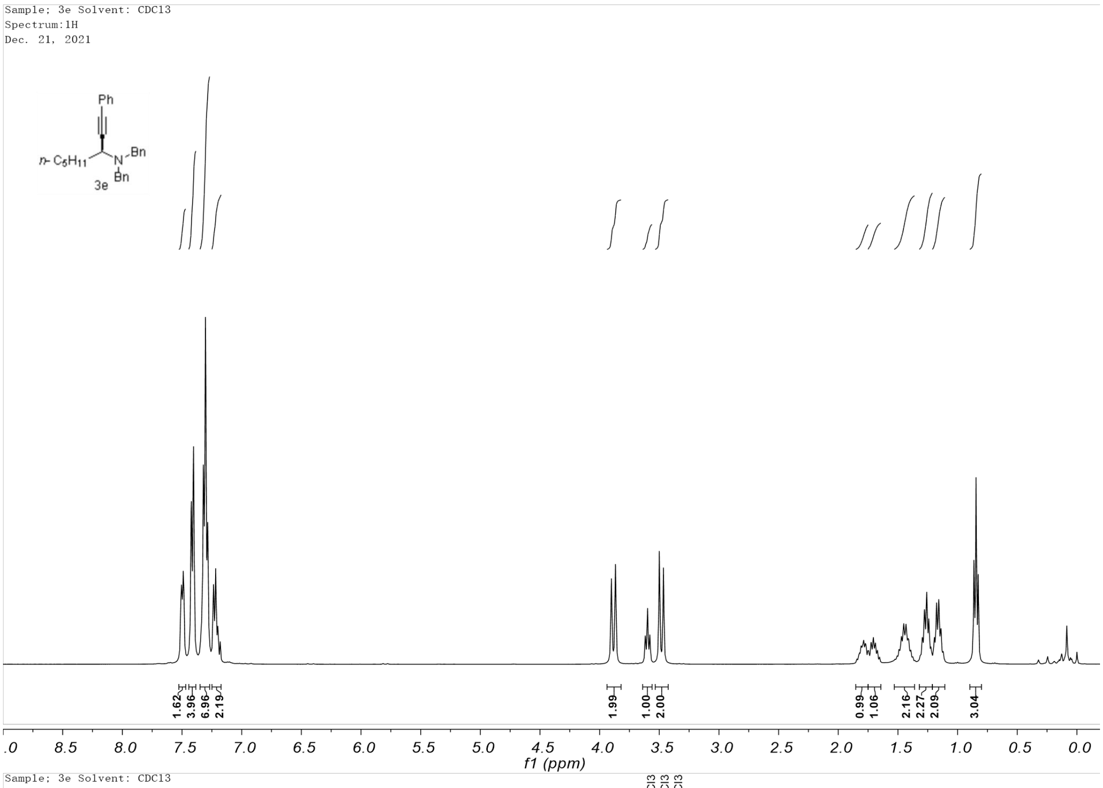

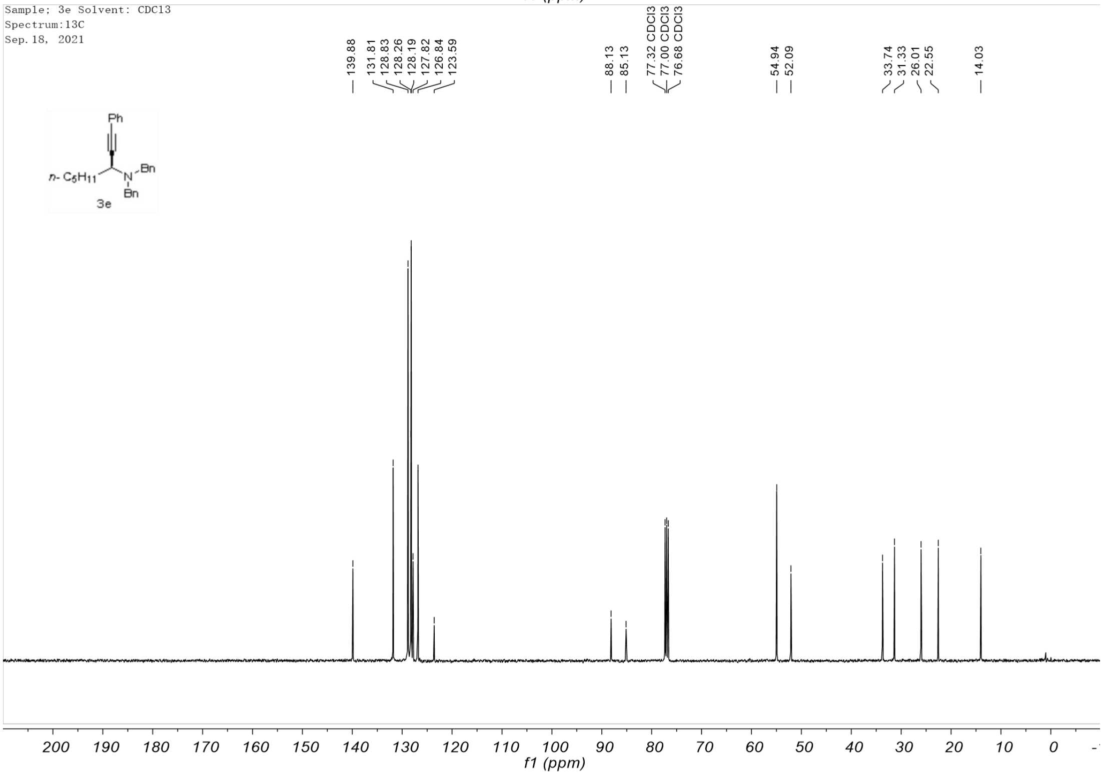

Supplementary Fig. 50. ¹H NMR (400 MHz, $CDCl_3$) and ¹³C NMR (100 MHz, $CDCl_3$) spectra of 3f

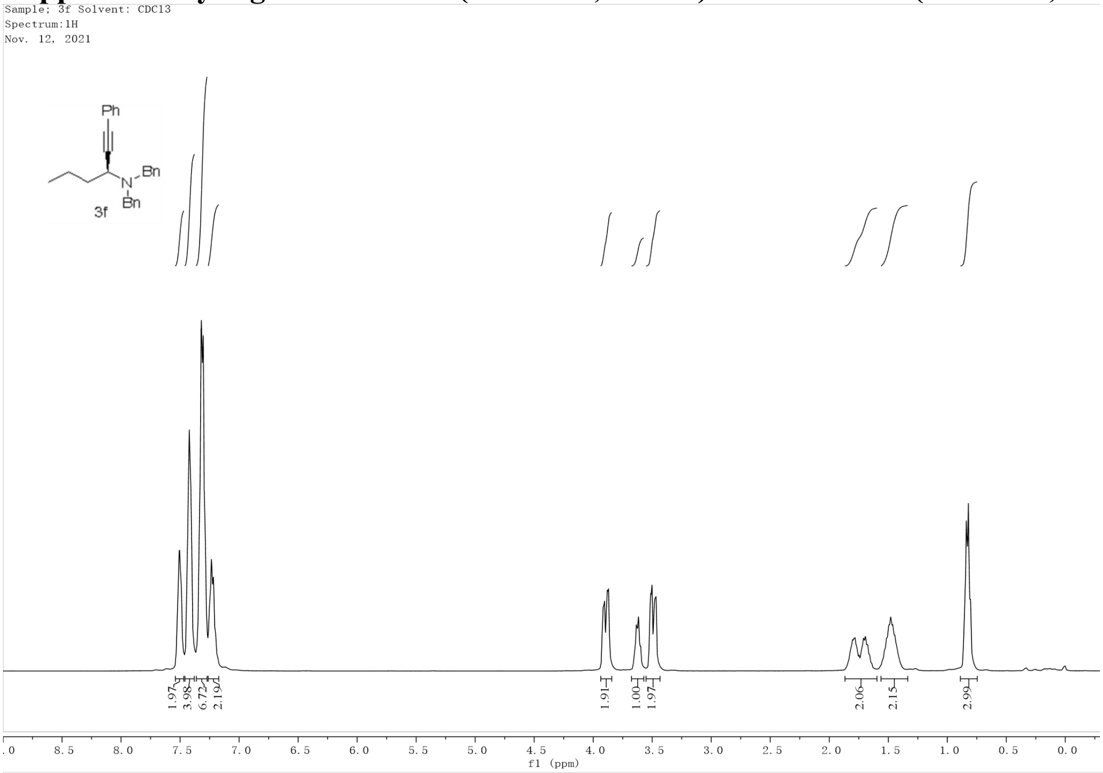

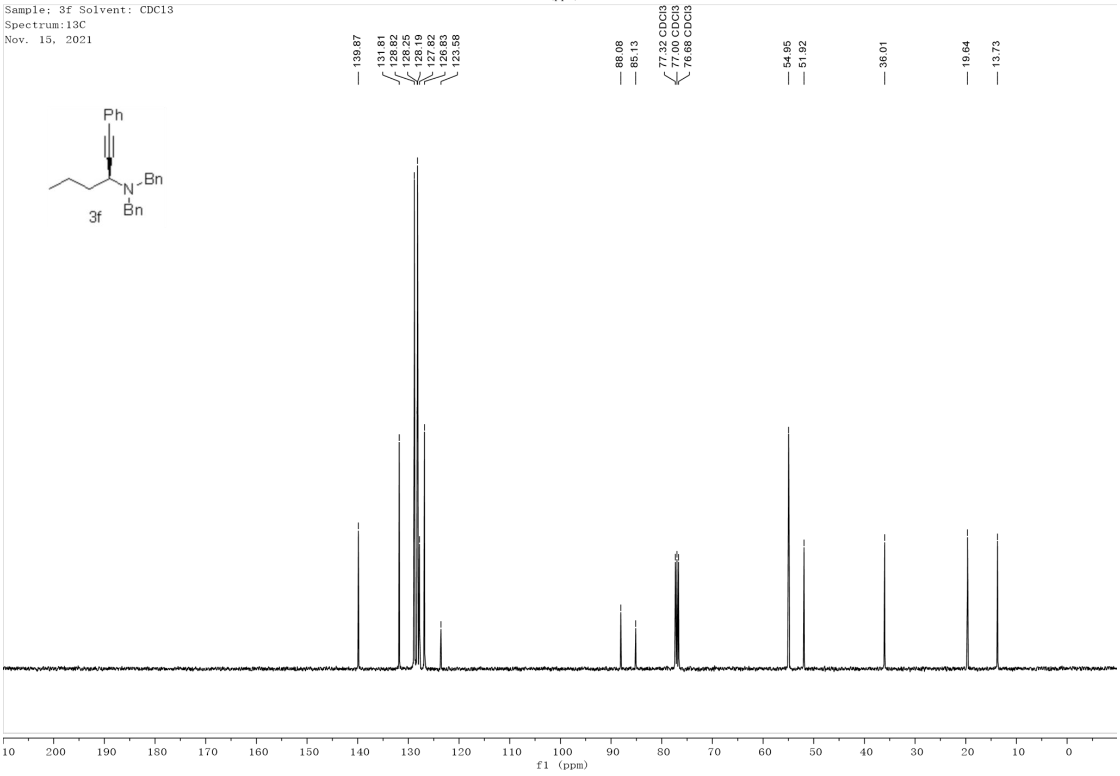

Supplementary Fig. 51. ^{1}H NMR (400 MHz, $CDCl_3$) and ^{13}C NMR (125 MHz, $CDCl_3$) spectra of 3g

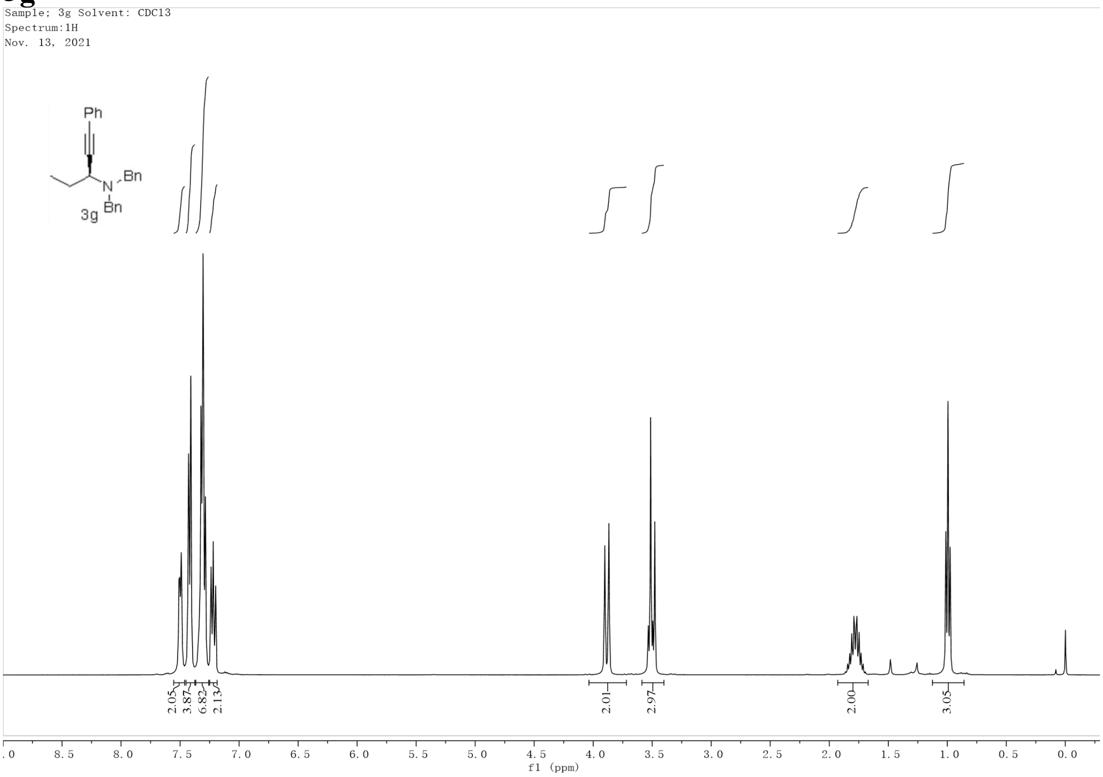

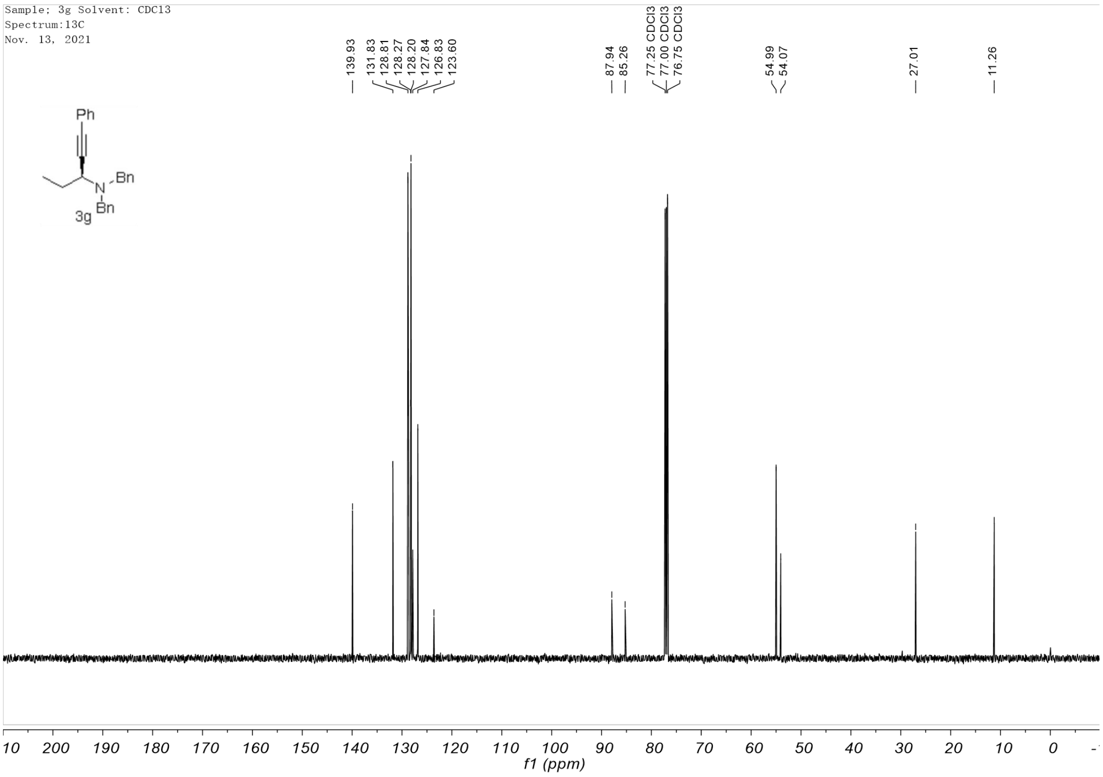

Supplementary Fig. 52. ^{1}H NMR (500 MHz, $CDCl_3$) and ^{13}C NMR (126 MHz, $CDCl_3$) spectra of 3h

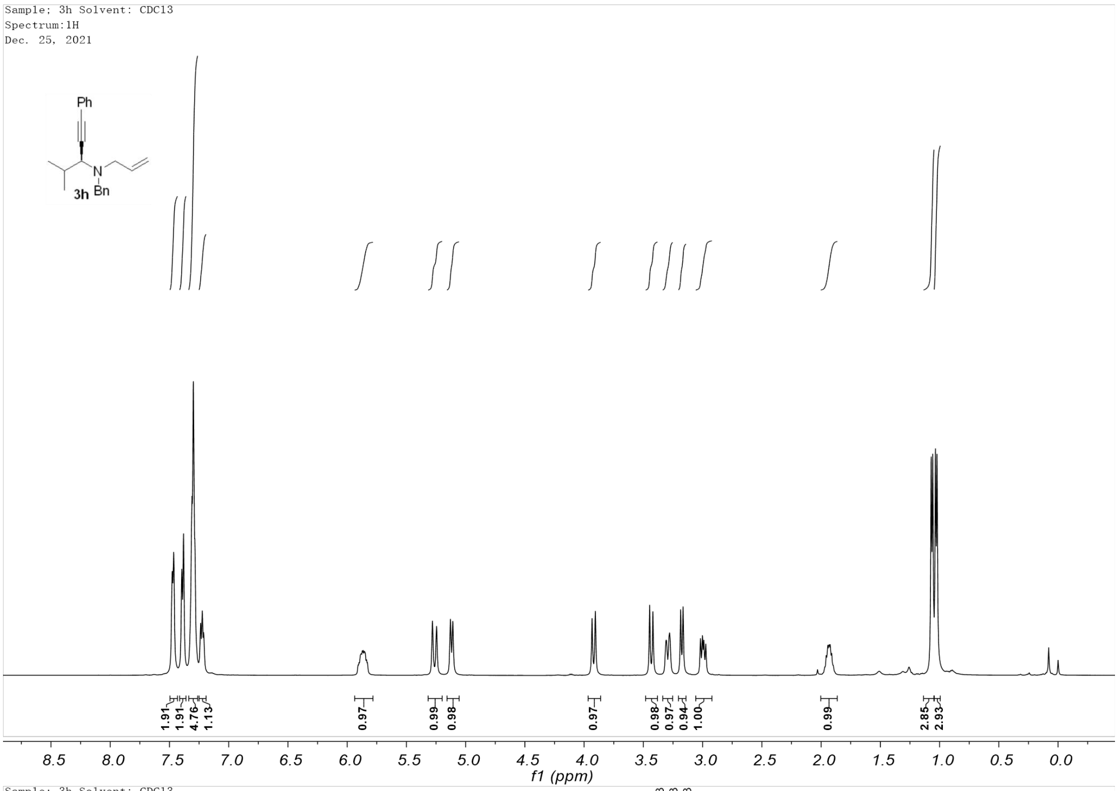

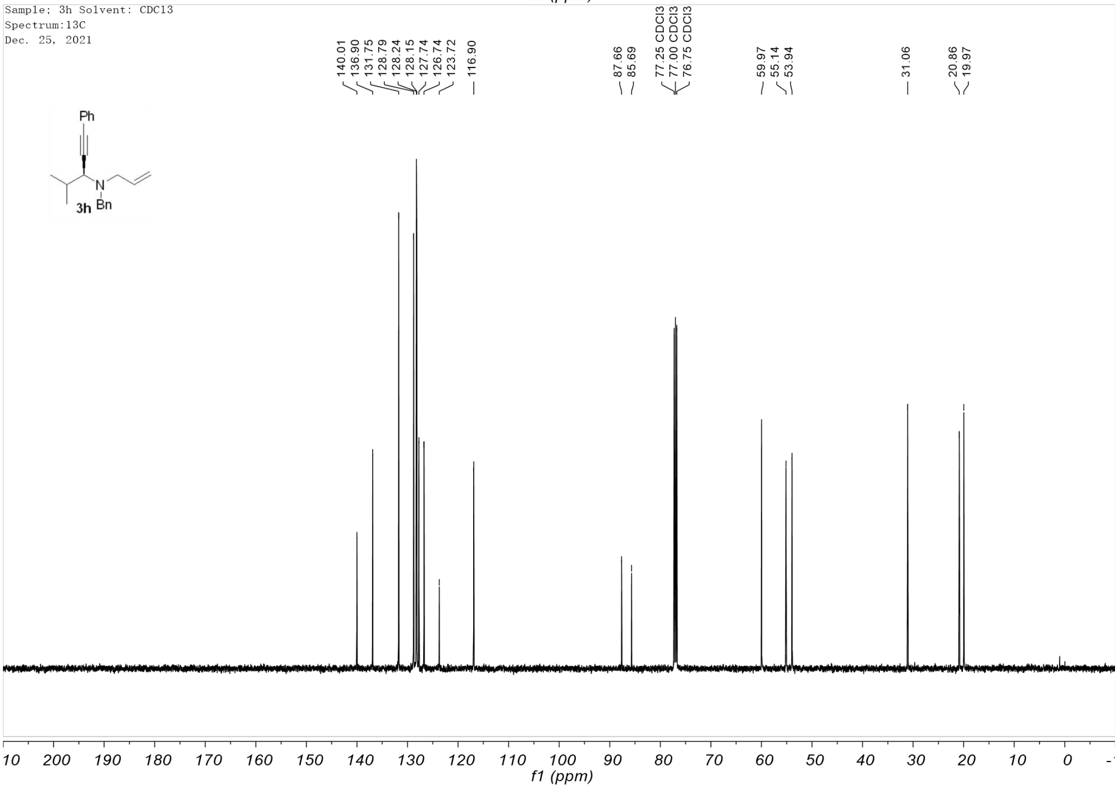

Supplementary Fig. 53. ^{1}H NMR (400 MHz, $CDCl_3$) and ^{13}C NMR (100 MHz, $CDCl_3$) spectra of 3i

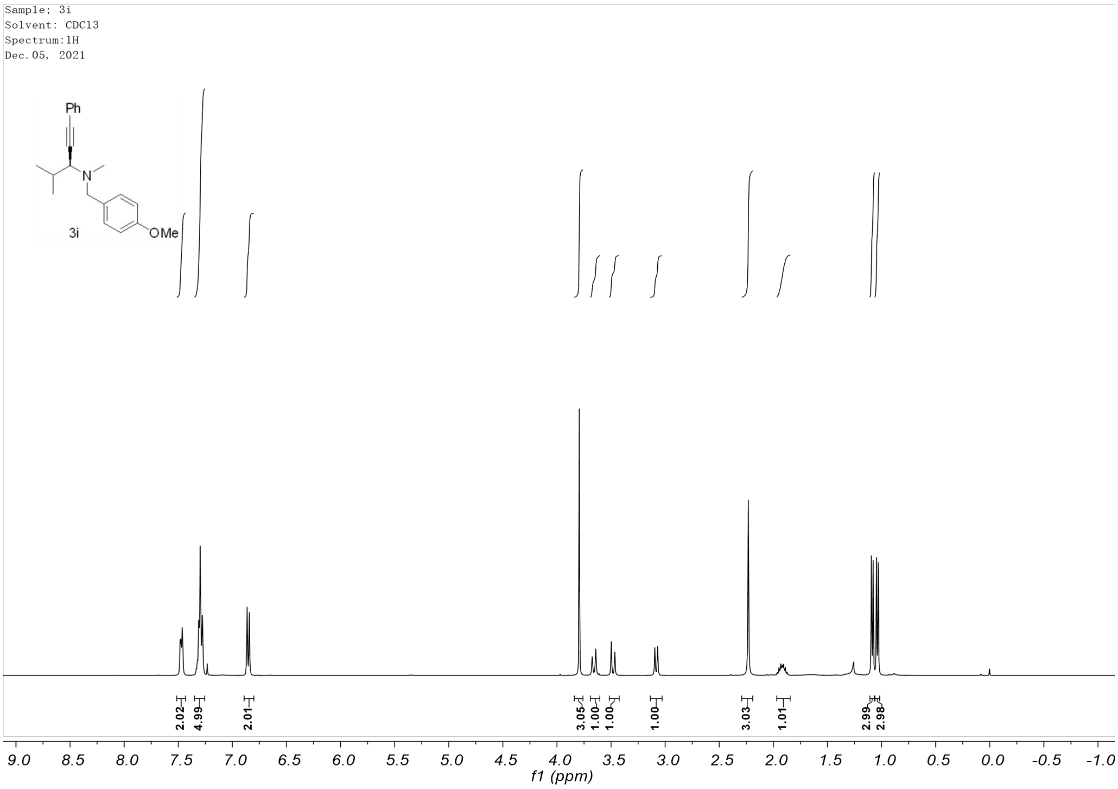

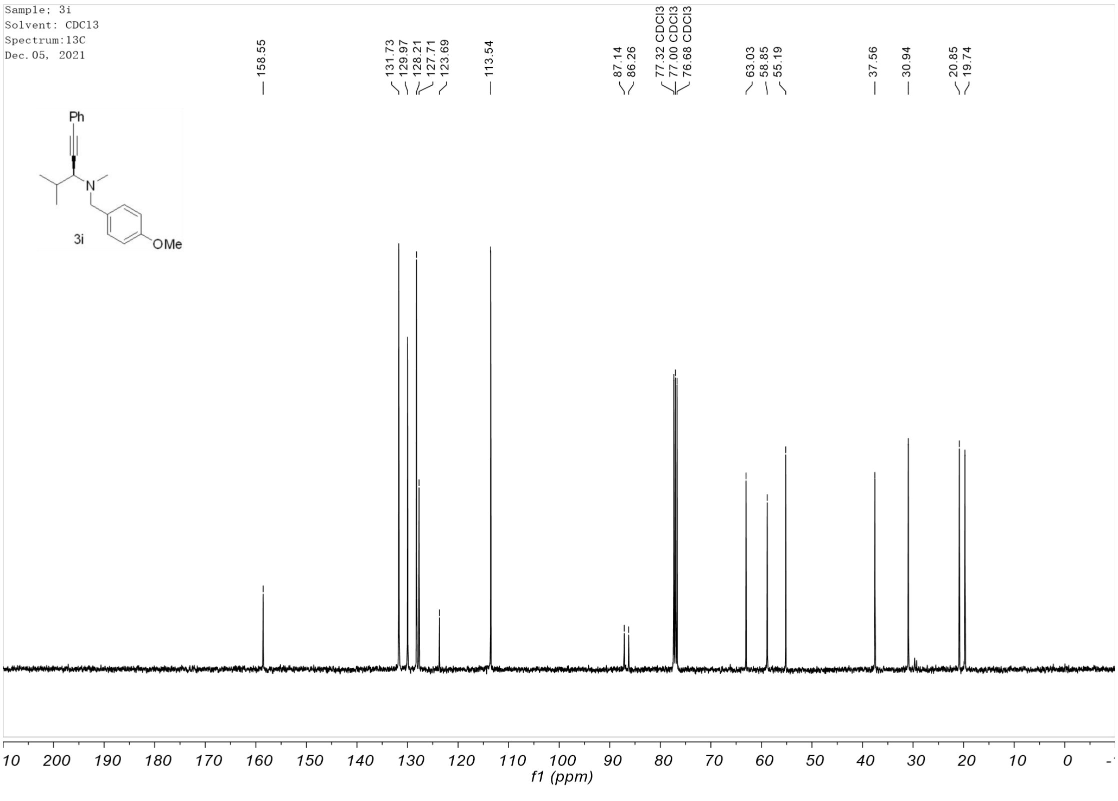

Supplementary Fig. 54. ^{1}H NMR (400 MHz, $CDCl_3$) and ^{13}C NMR (100 MHz, $CDCl_3$) spectra of 3j

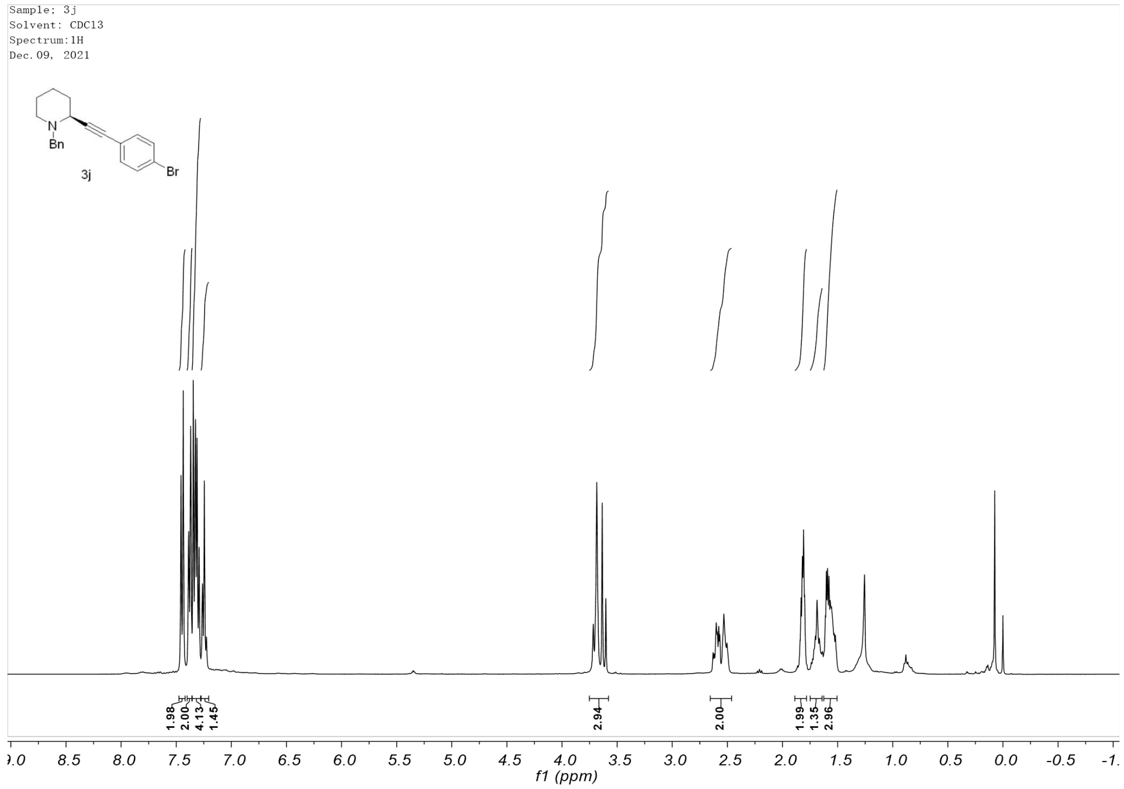

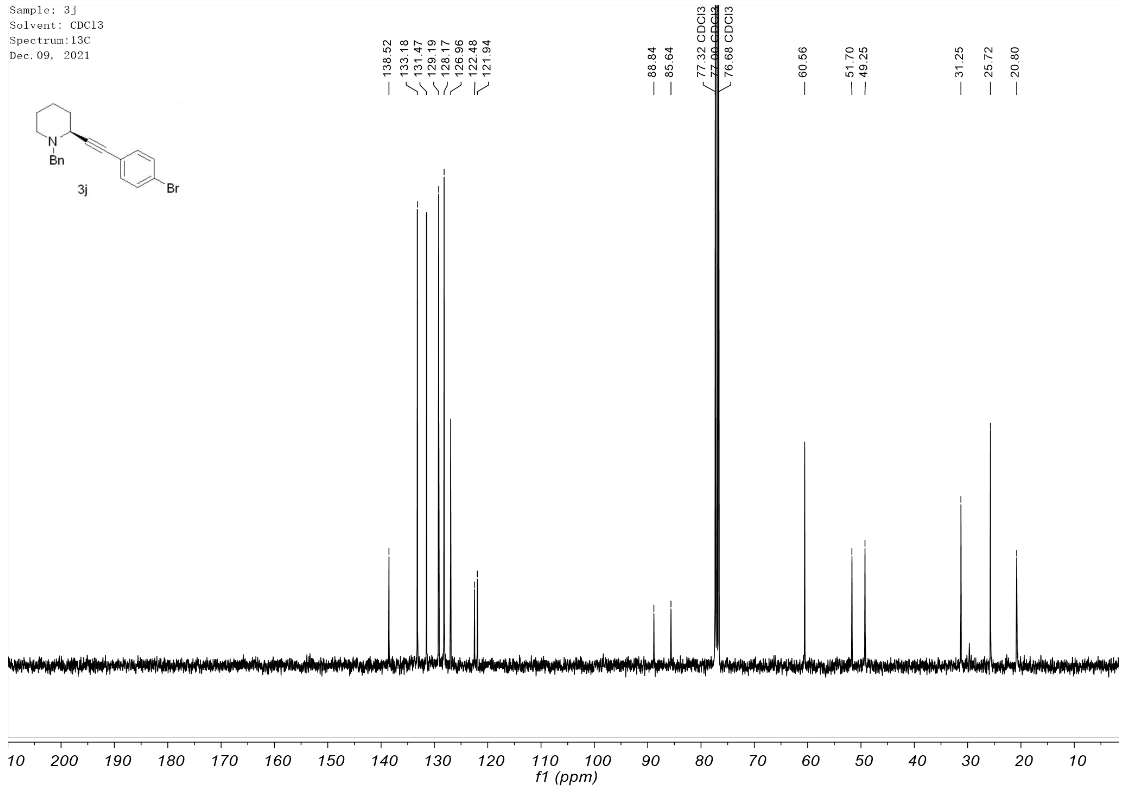

Supplementary Fig. 55. ^{1}H NMR (500 MHz, $CDCl_3$) and ^{13}C NMR (100 MHz, $CDCl_3$) spectra of 3k

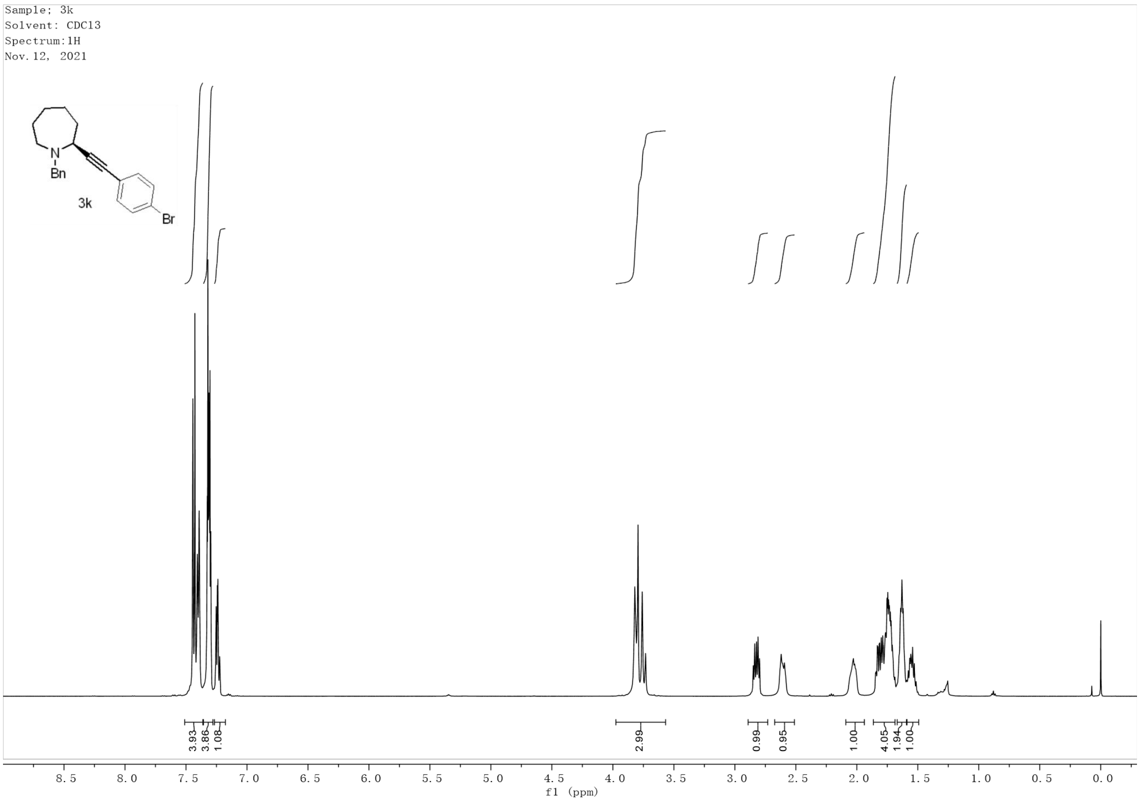

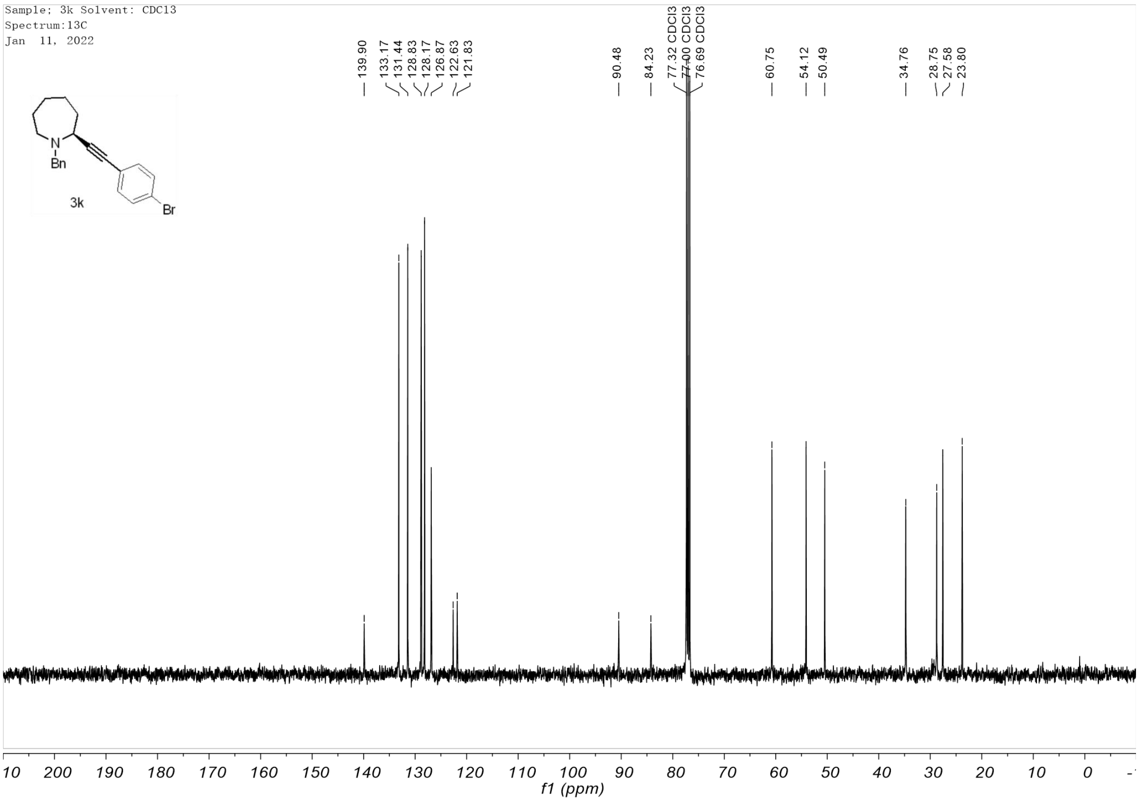

Supplementary Fig. 56. ^{1}H NMR (400 MHz, $CDCl_3$) and ^{13}C NMR (100 MHz, $CDCl_3$) spectra of 3l

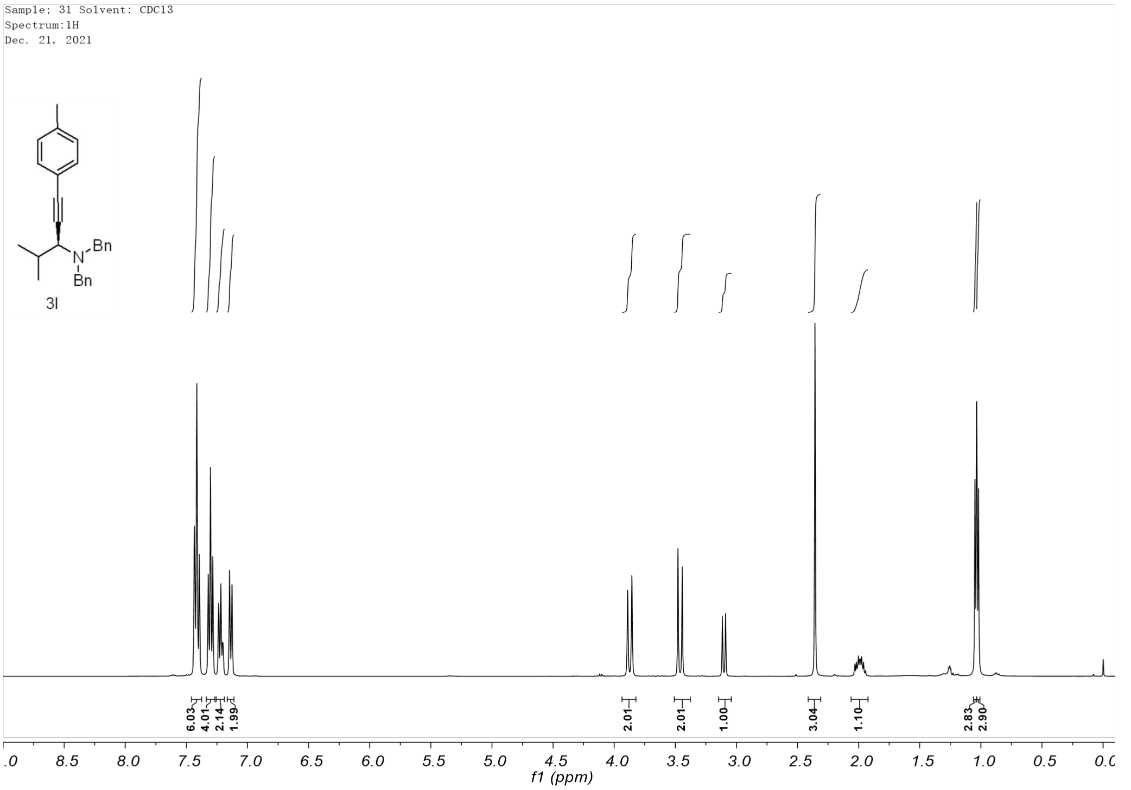

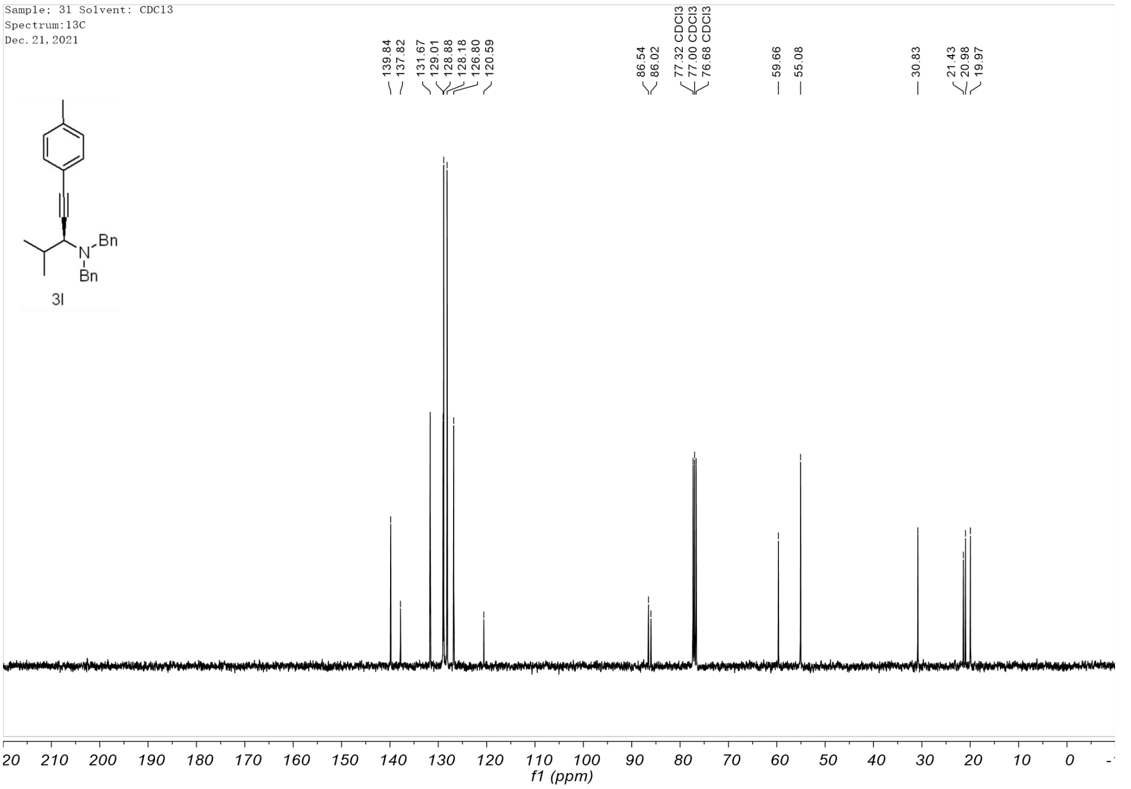

Supplementary Fig. 57. ^{1}H NMR (400 MHz, $CDCl_3$) and ^{13}C NMR (100 MHz, $CDCl_3$) spectra of 3m

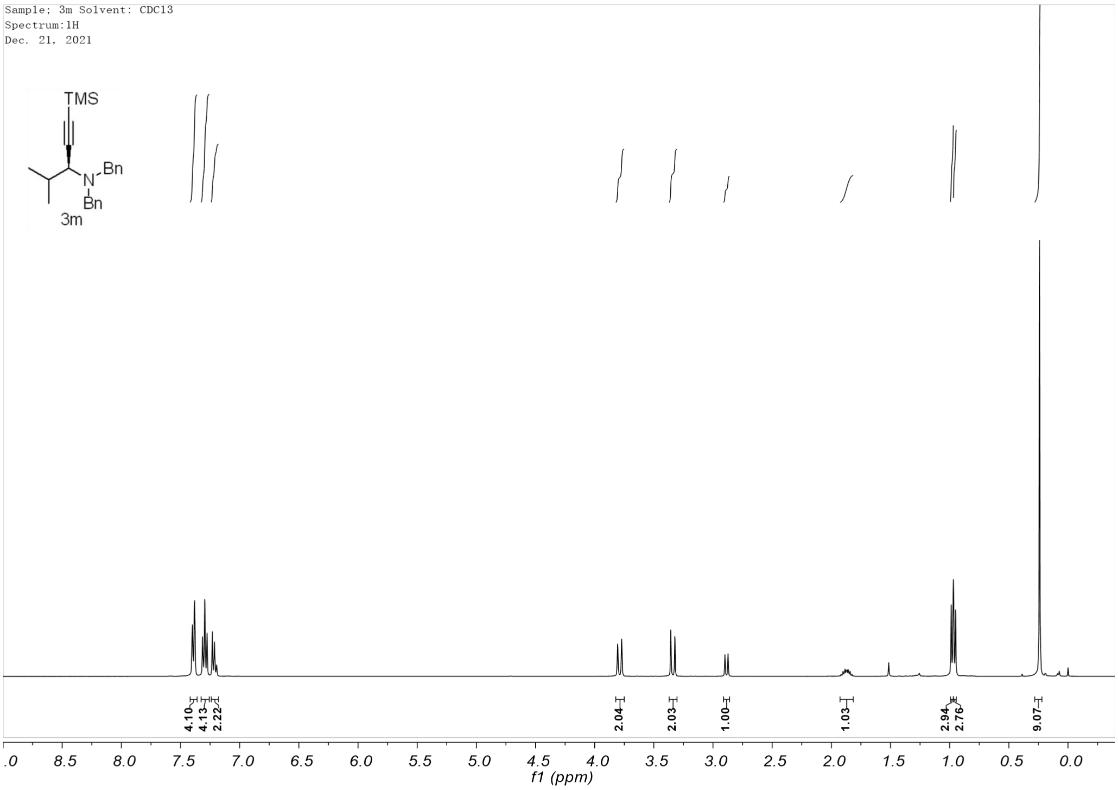

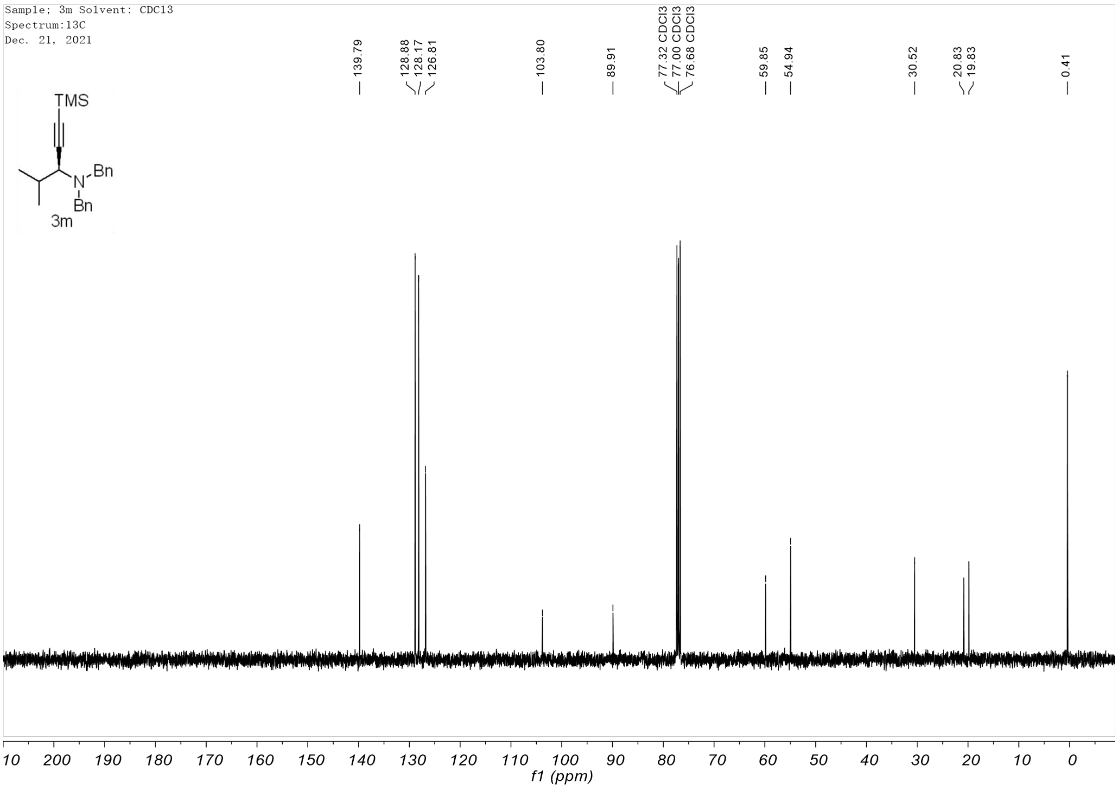

Supplementary Fig. 58. ^{1}H NMR (400 MHz, $CDCl_3$) and ^{13}C NMR (100 MHz, $CDCl_3$) spectra of 3n

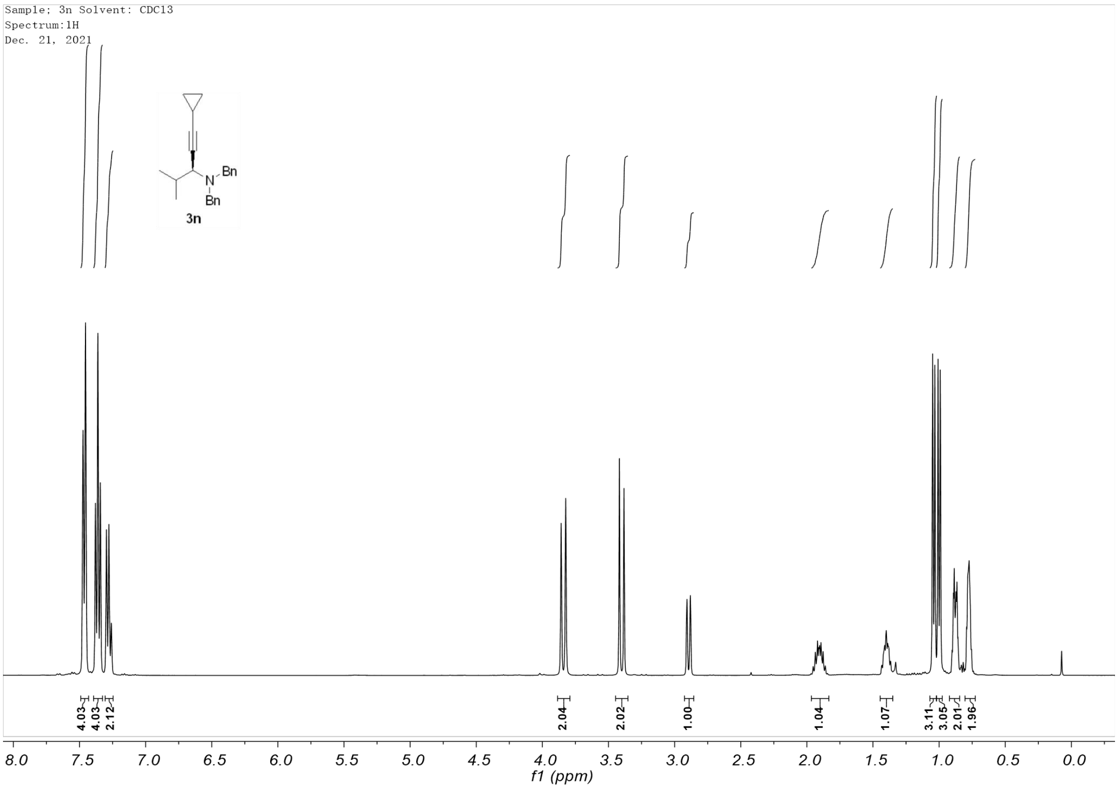

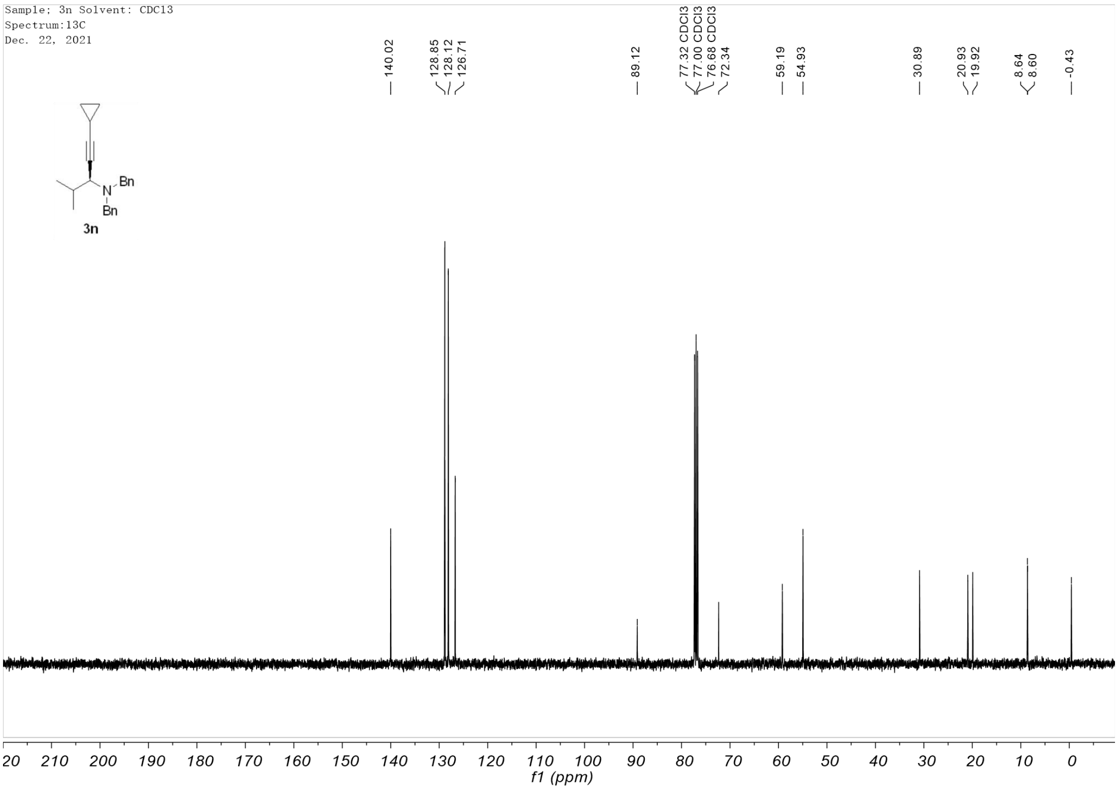

Supplementary Fig. 59. ^{1}H NMR (500 MHz, $CDCl_3$) and ^{13}C NMR (126 MHz, $CDCl_3$) spectra of 3o

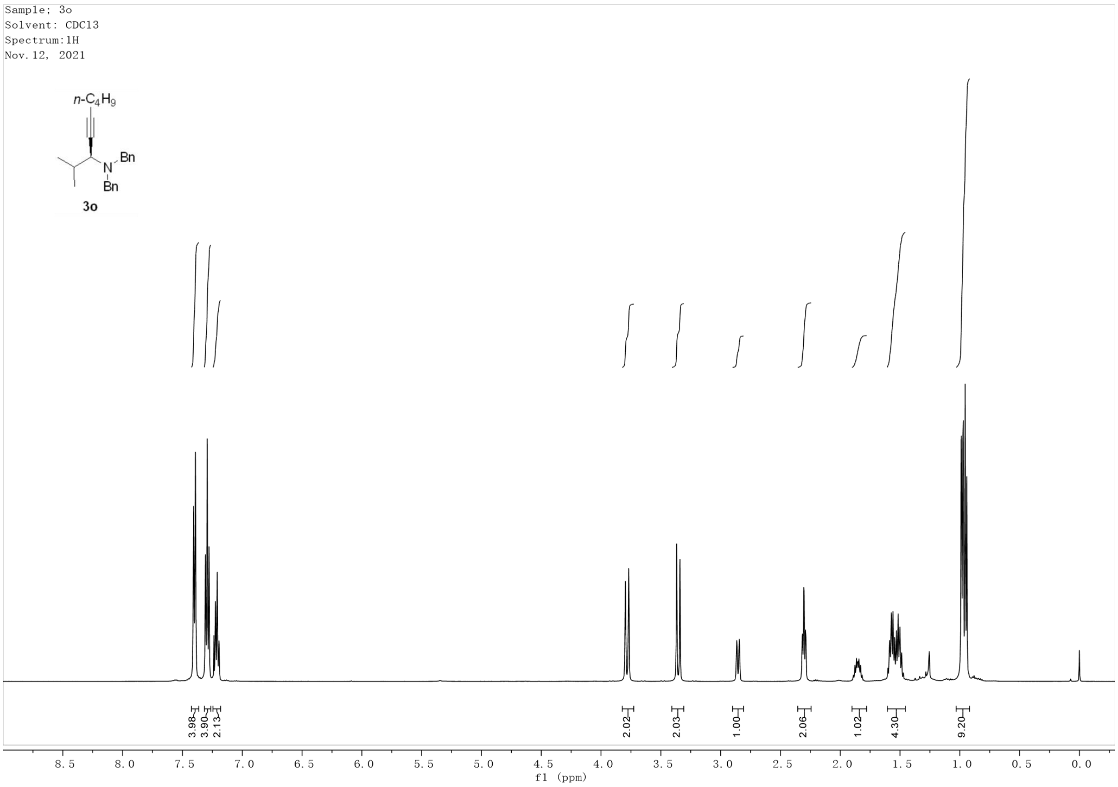

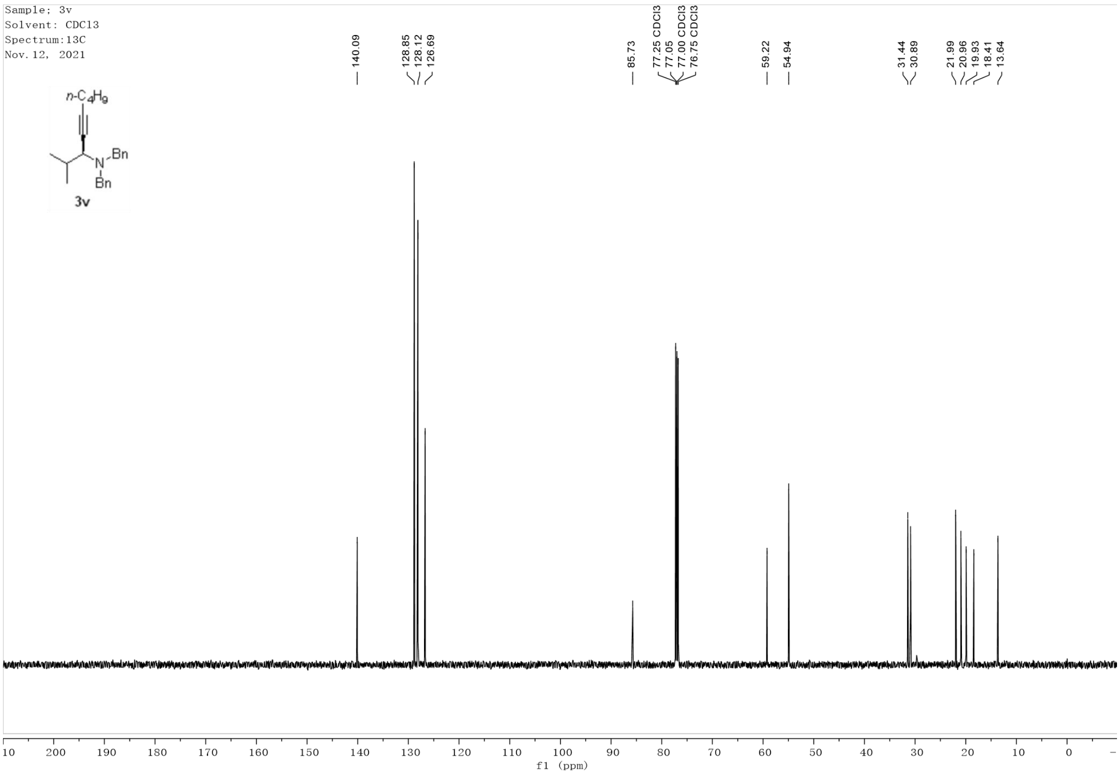

Supplementary Fig. 60. ^{1}H NMR (500 MHz, $CDCl_3$) and ^{13}C NMR (126 MHz, $CDCl_3$) spectra of 3p

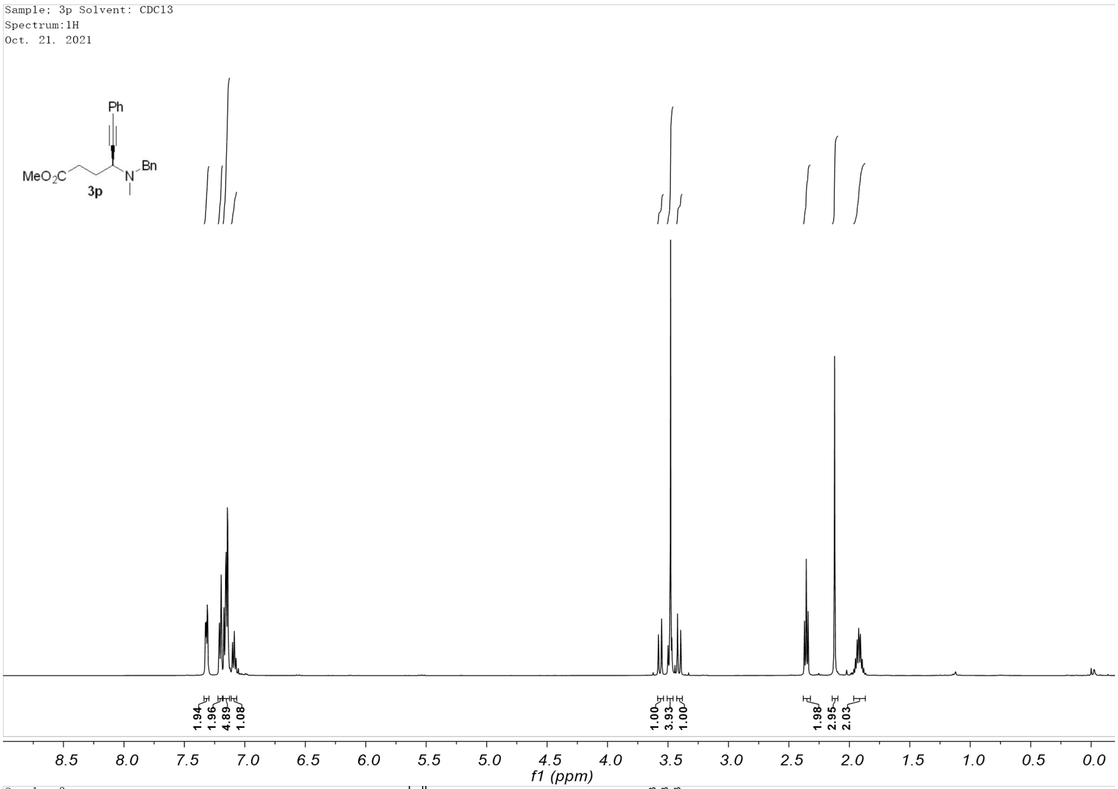

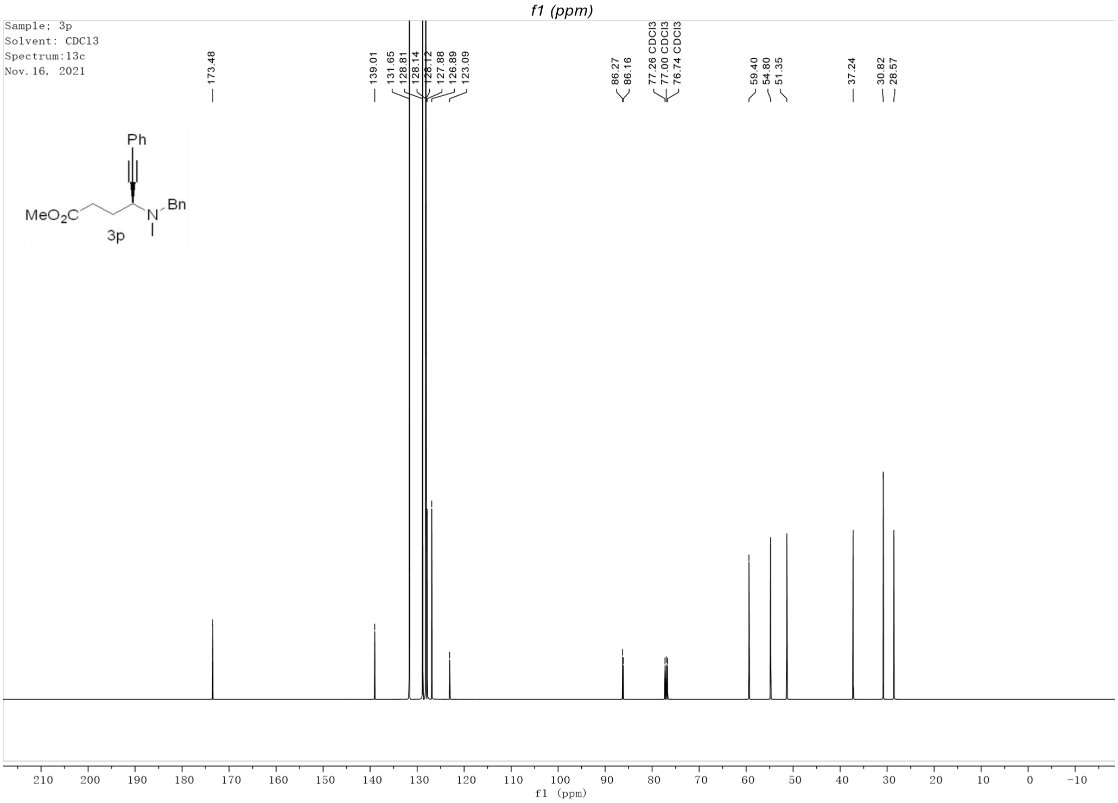

Supplementary Fig. 61. ^{1}H NMR (400 MHz, $CDCl_3$) and ^{13}C NMR (100 MHz, $CDCl_3$) spectra of 3q

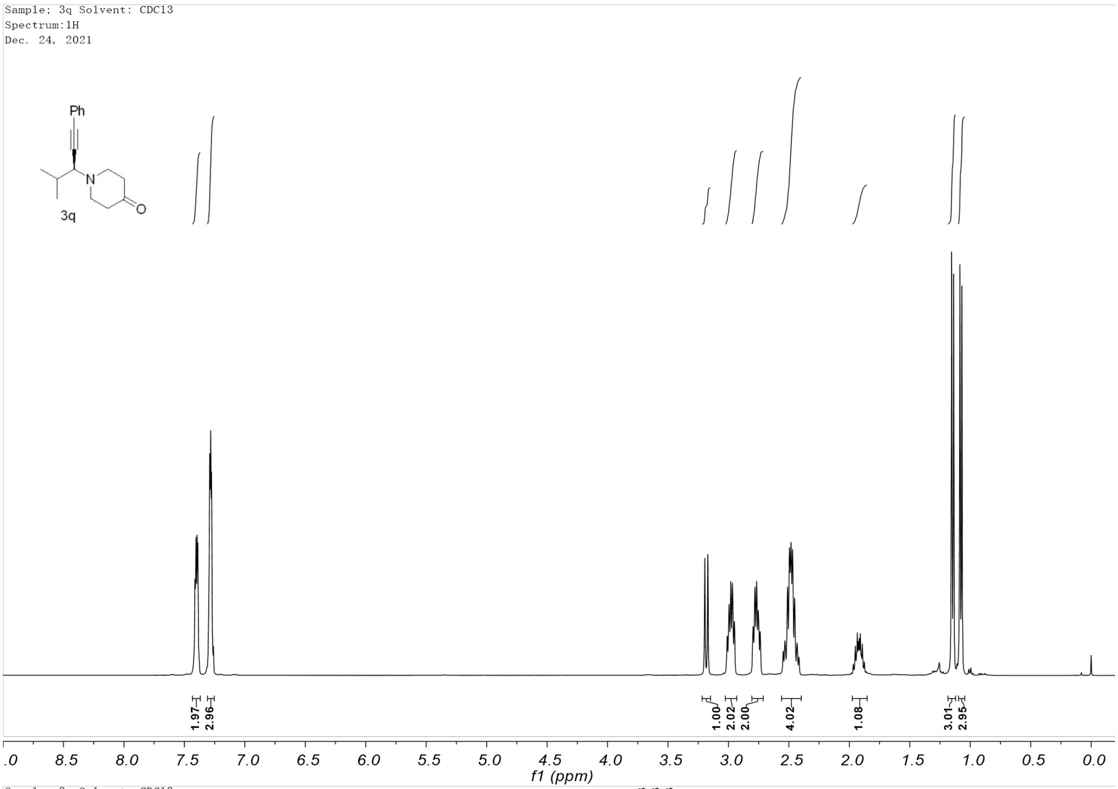

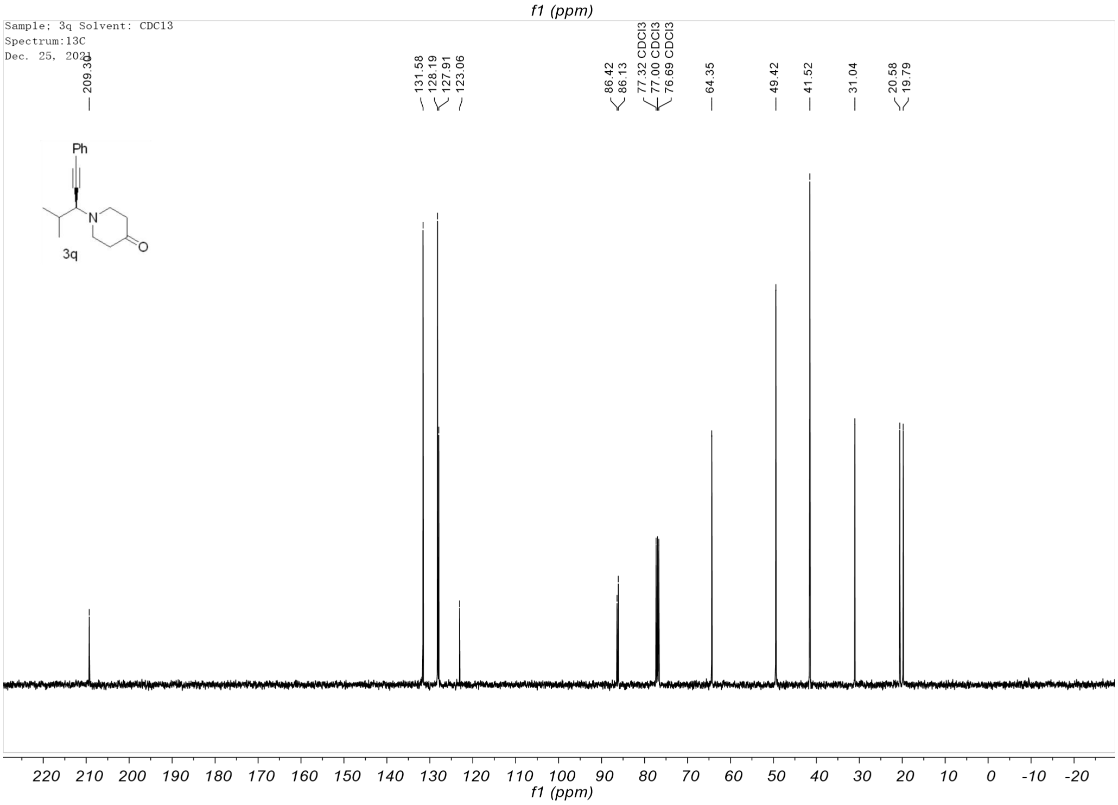

Supplementary Fig. 62. ^{1}H NMR (500 MHz, $CDCl_3$) and ^{13}C NMR (126 MHz, $CDCl_3$) spectra of 3r

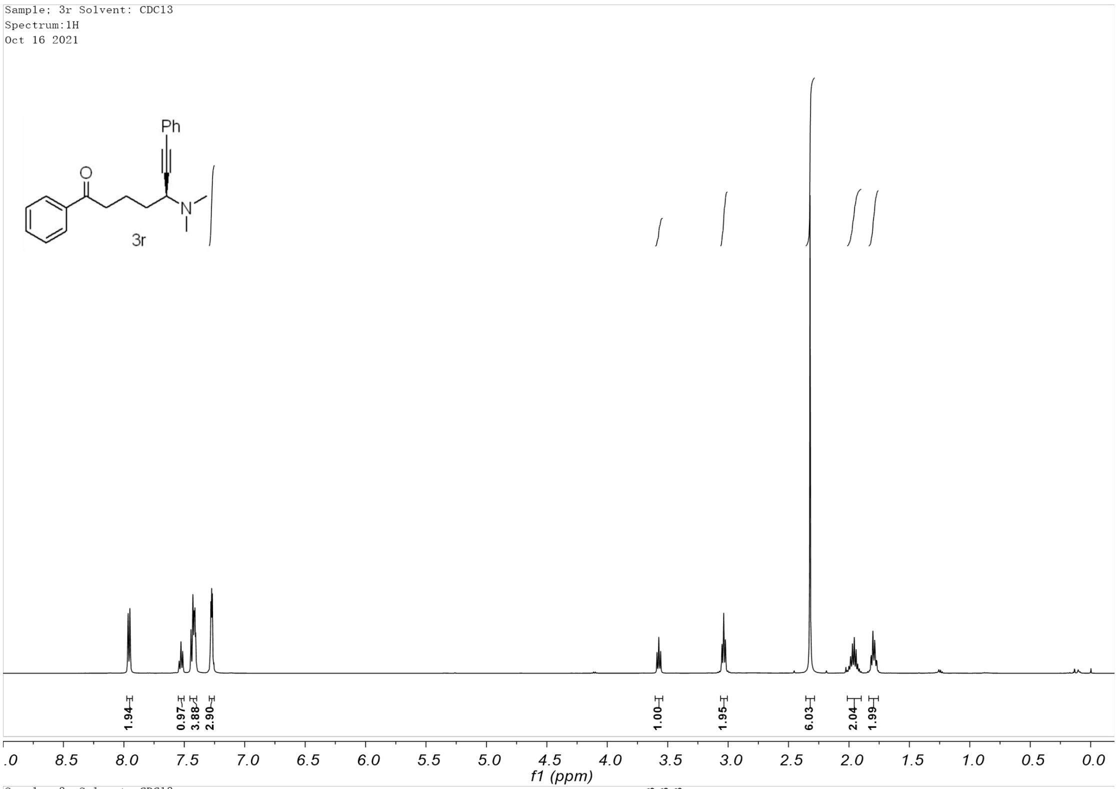

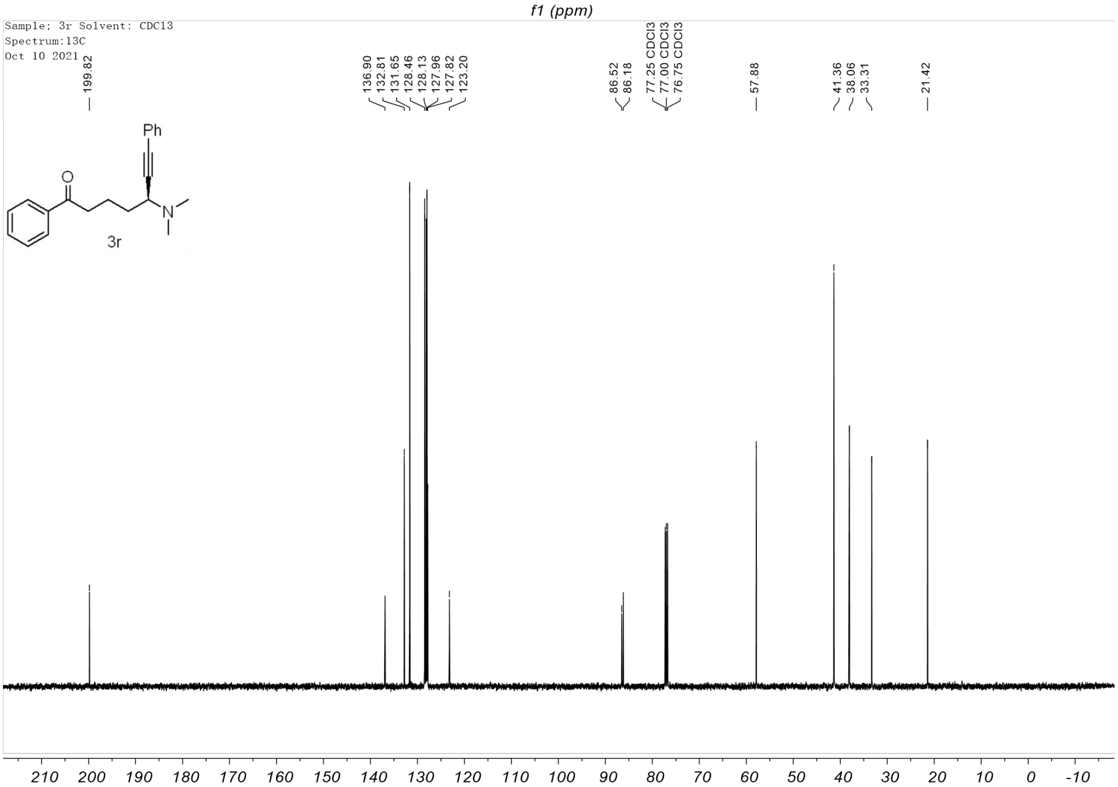

Supplementary Fig. 63. ^{1}H NMR (400 MHz, $CDCl_3$) and ^{13}C NMR (100 MHz, $CDCl_3$) spectra of 3s

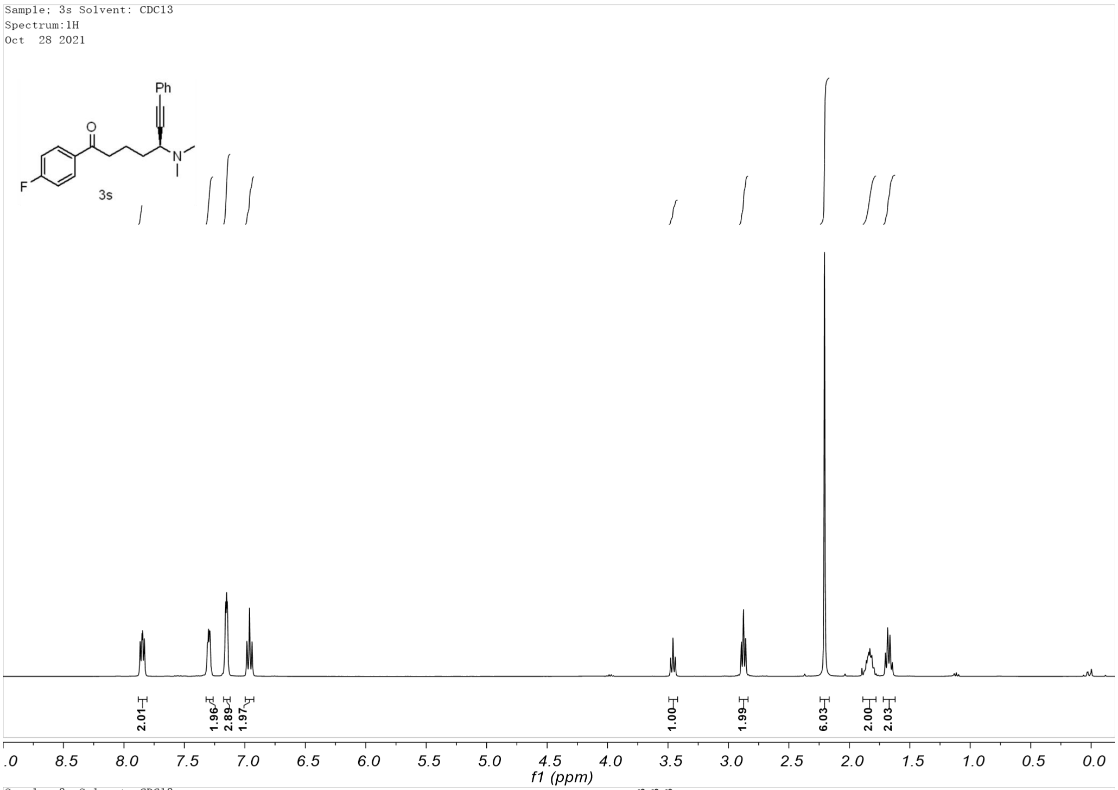

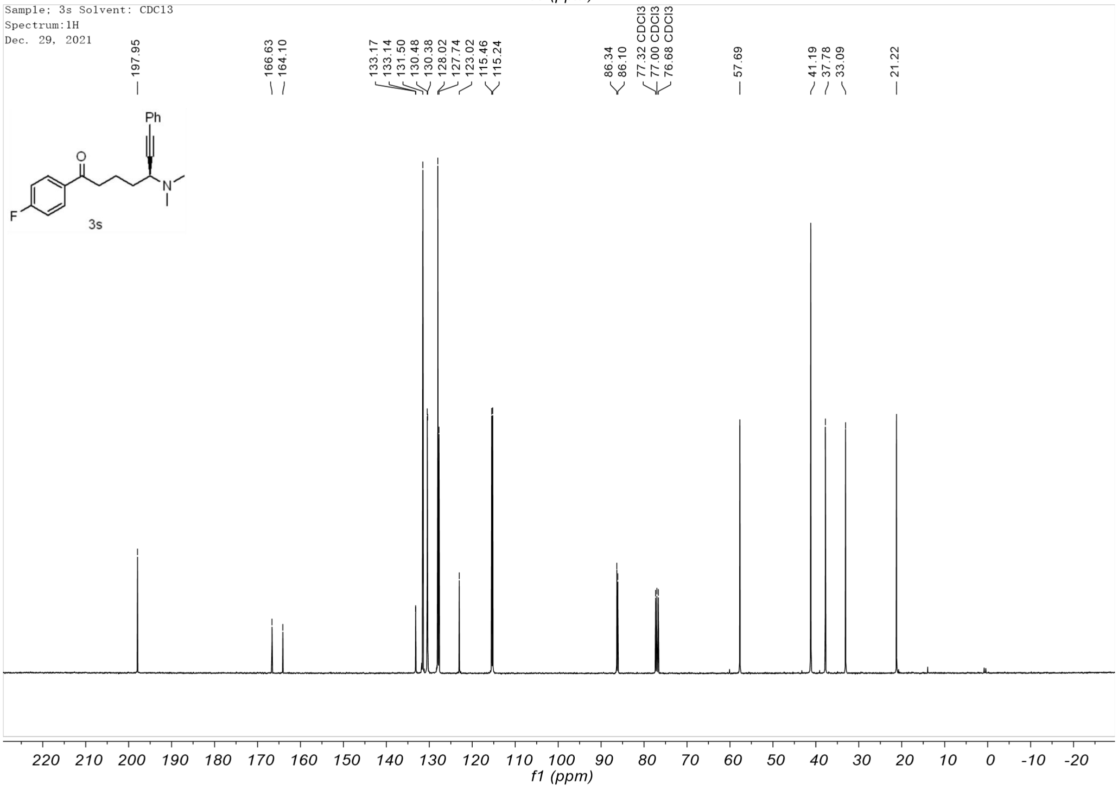

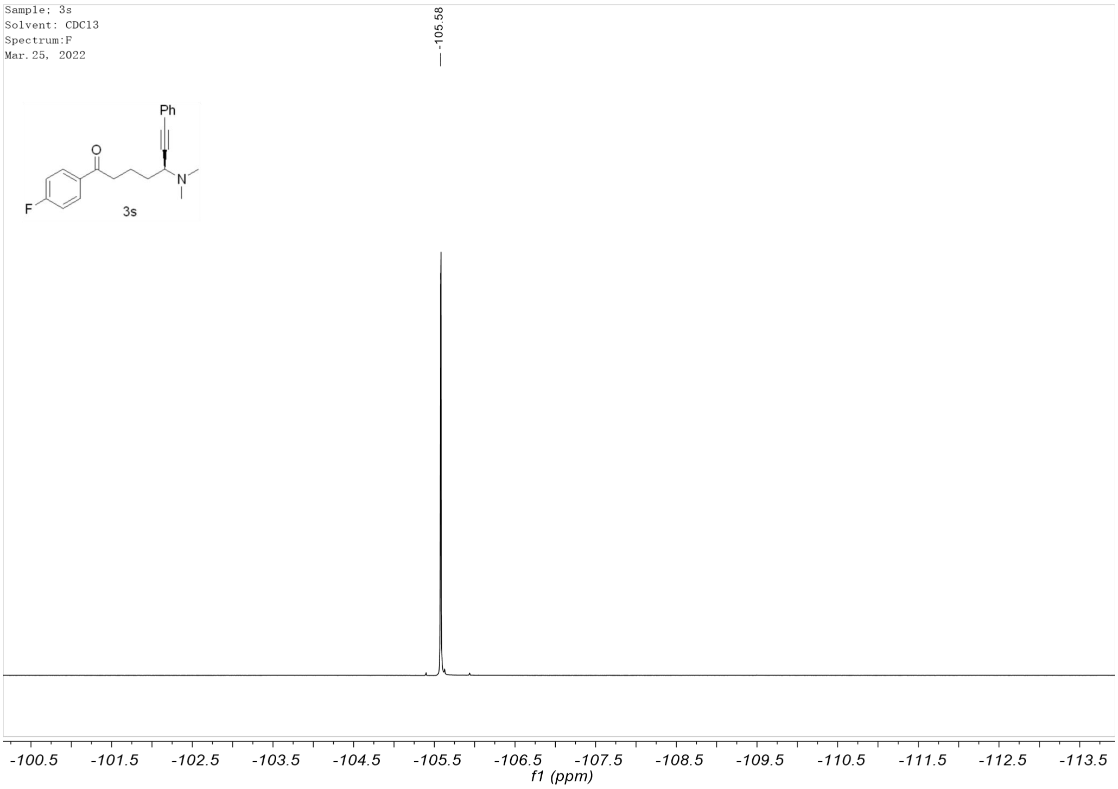

Sample: 3s
Solvent: CDCl3
Spectrum:F
Mar.25, 2022
-105.58
Ph
O
N
F
3s
-100.5
-101.5
-102.5
-103.5
-104.5
-105.5
-106.5
-107.5
-108.5
-109.5
-110.5
-111.5
-112.5
-113.5
f1 (ppm)

Supplementary Fig. 64. ^{1}H NMR (500 MHz, $CDCl_3$) and ^{13}C NMR (126 MHz, $CDCl_3$) spectra of 3t

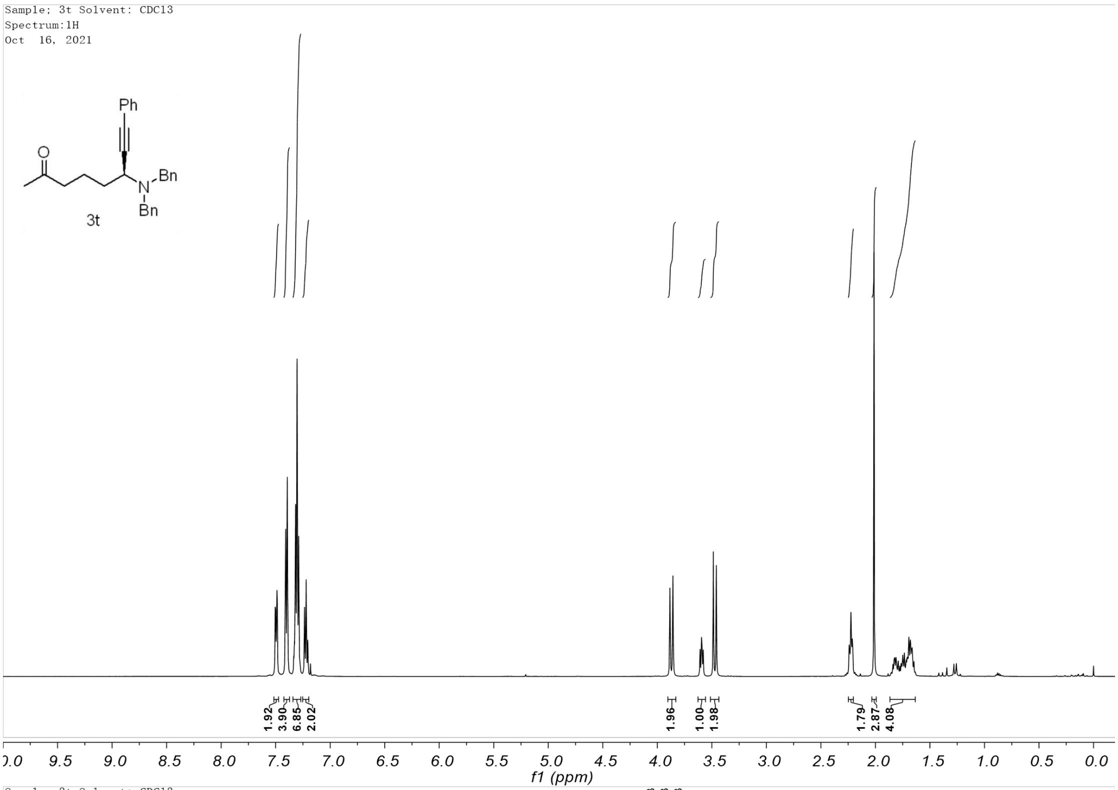

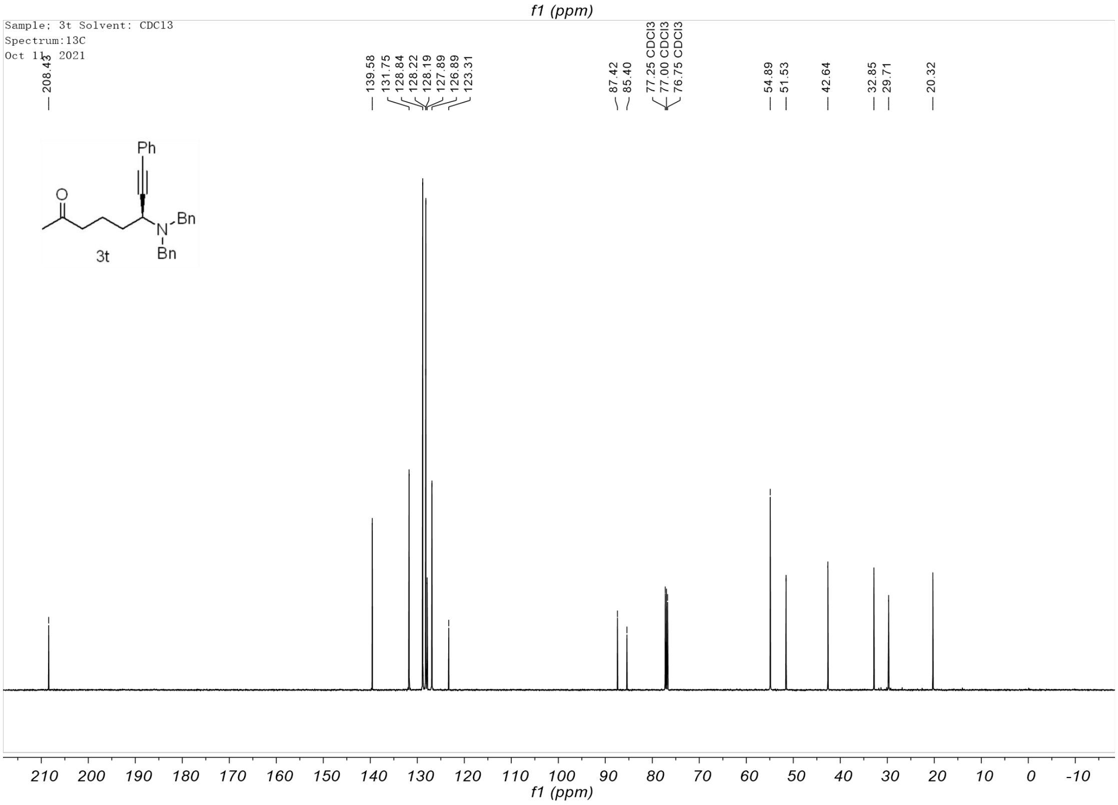

Supplementary Fig. 65. ^{1}H NMR (400 MHz, $CDCl_3$) and ^{13}C NMR (100 MHz, $CDCl_3$) spectra of 3u

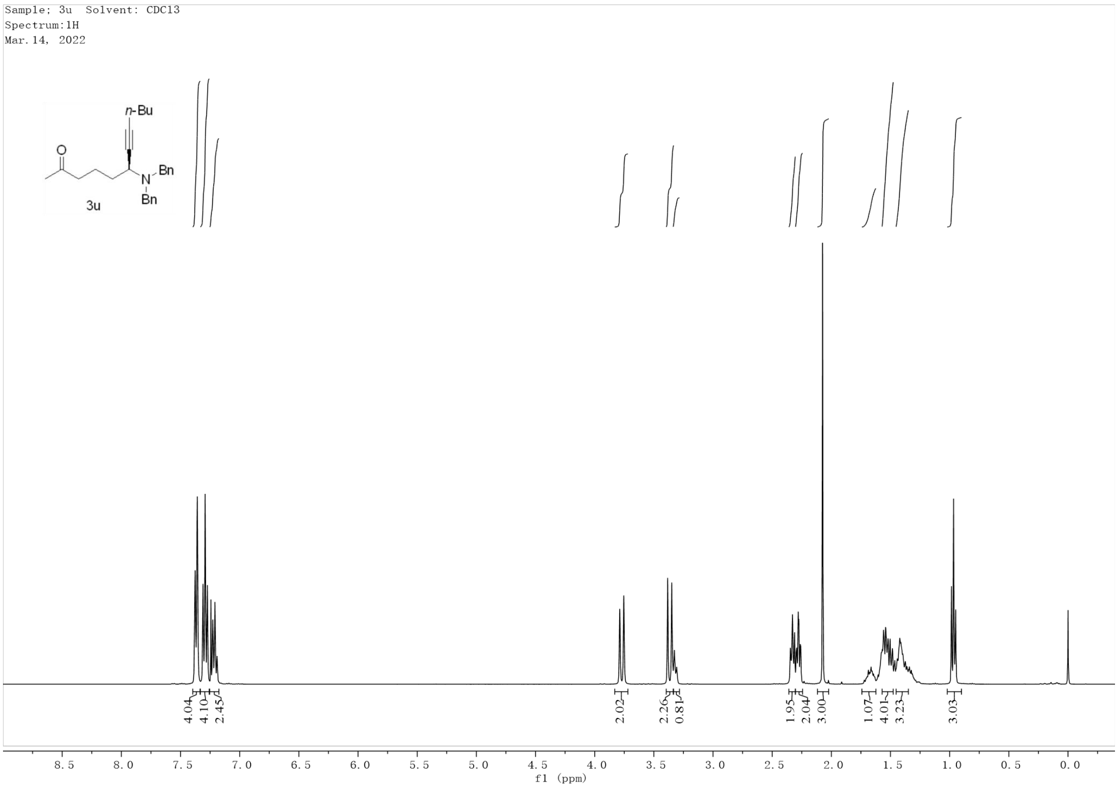

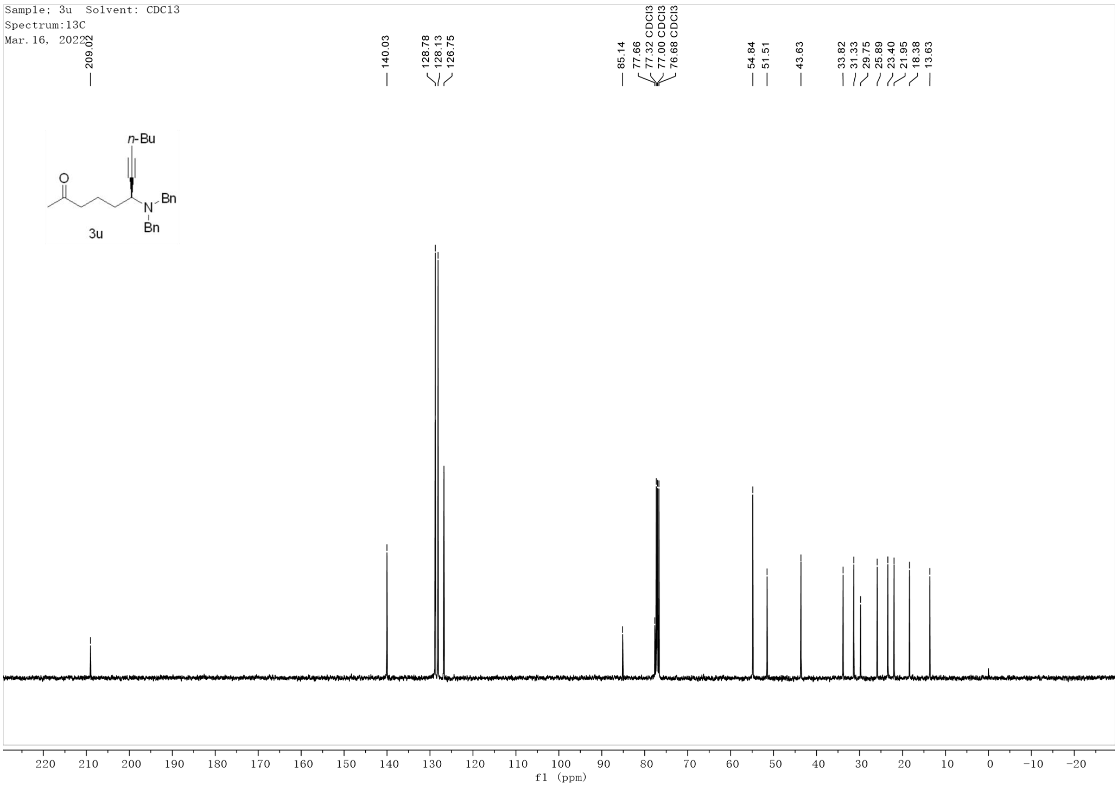

Supplementary Fig. 66. ^{1}H NMR (500 MHz, CDCl$_3$) and ^{13}C NMR (126 MHz, CDCl$_3$) spectra of 3v

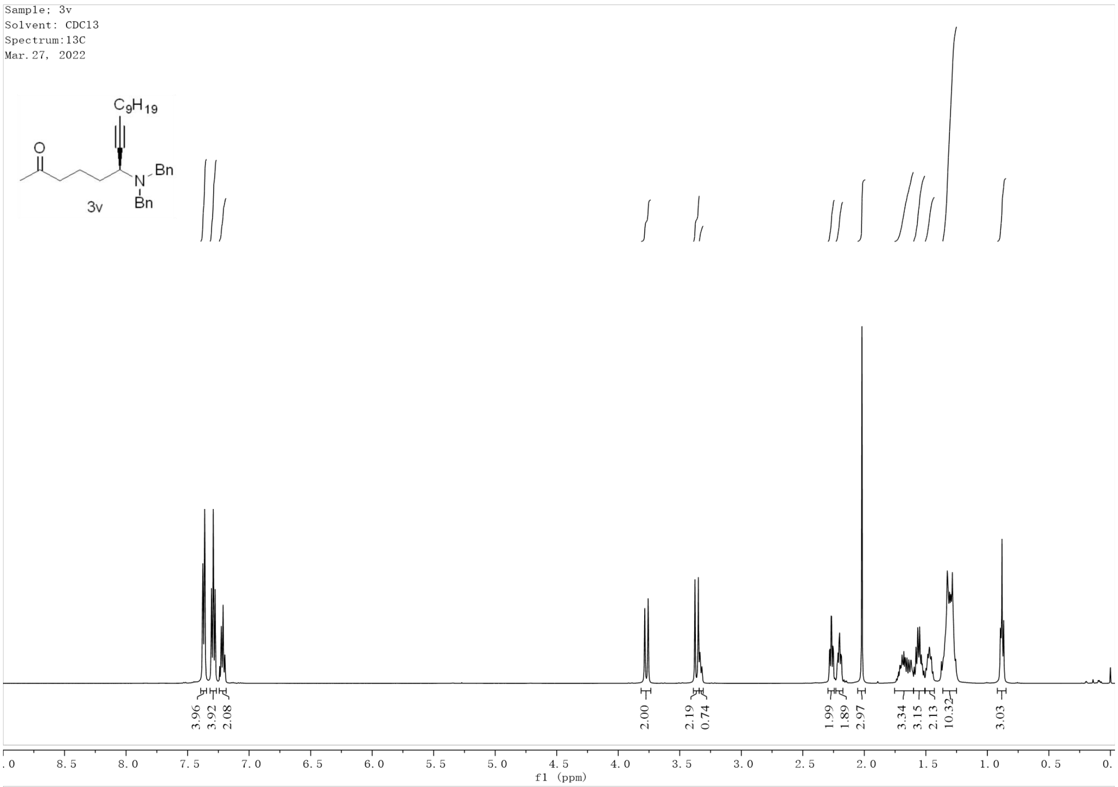

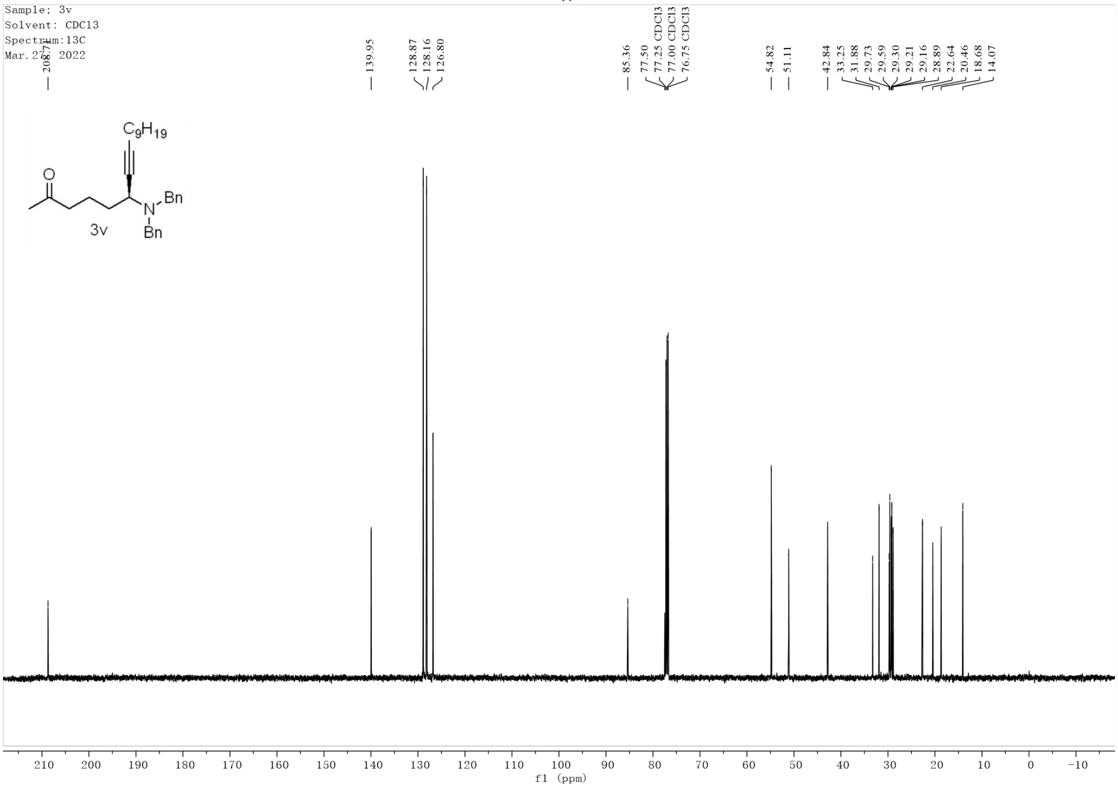

Supplementary Fig. 67. ^{1}H NMR (500 MHz, $CDCl_3$) and ^{13}C NMR (126 MHz, $CDCl_3$) spectra of 3w

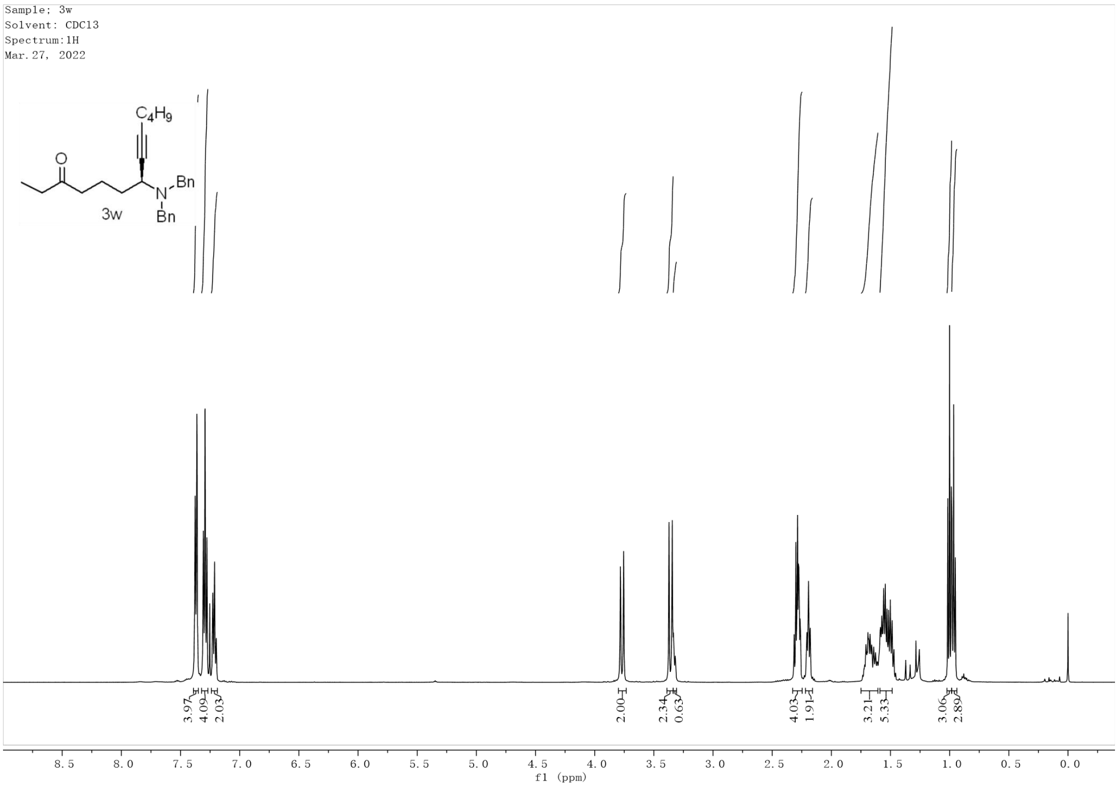

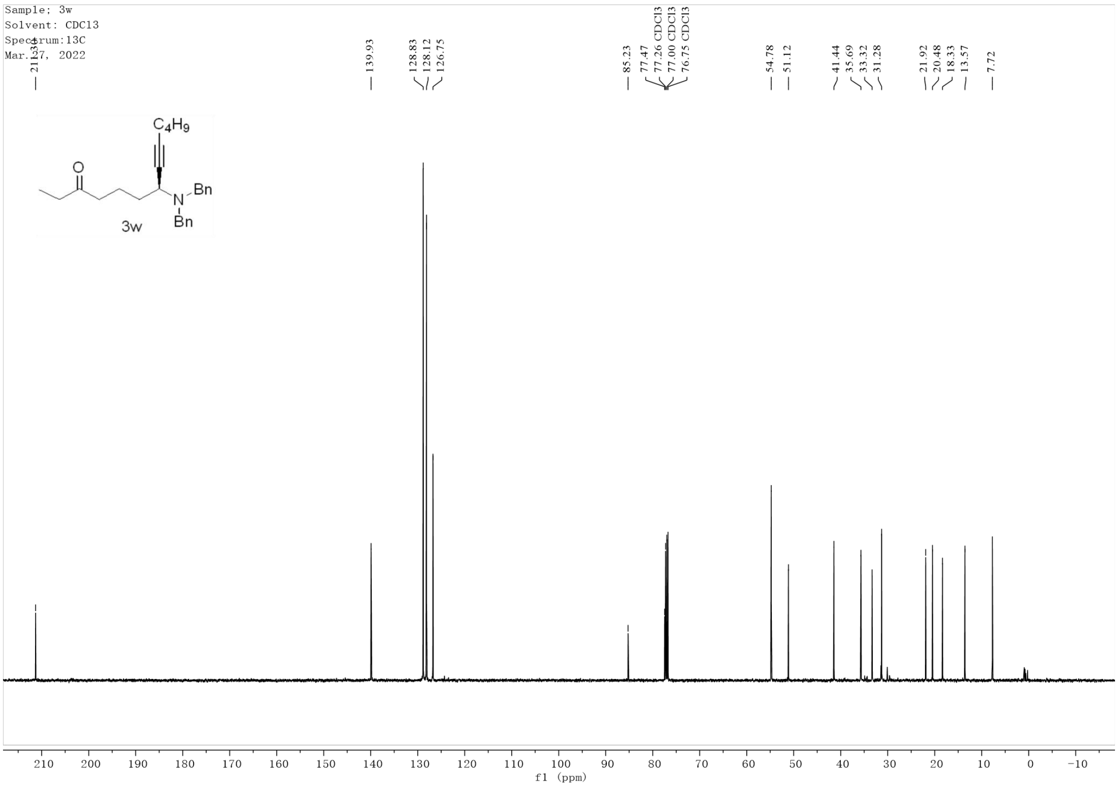

Supplementary Fig. 68. ^{1}H NMR (400 MHz, $CDCl_3$) and ^{13}C NMR (100 MHz, $CDCl_3$) spectra of 3x

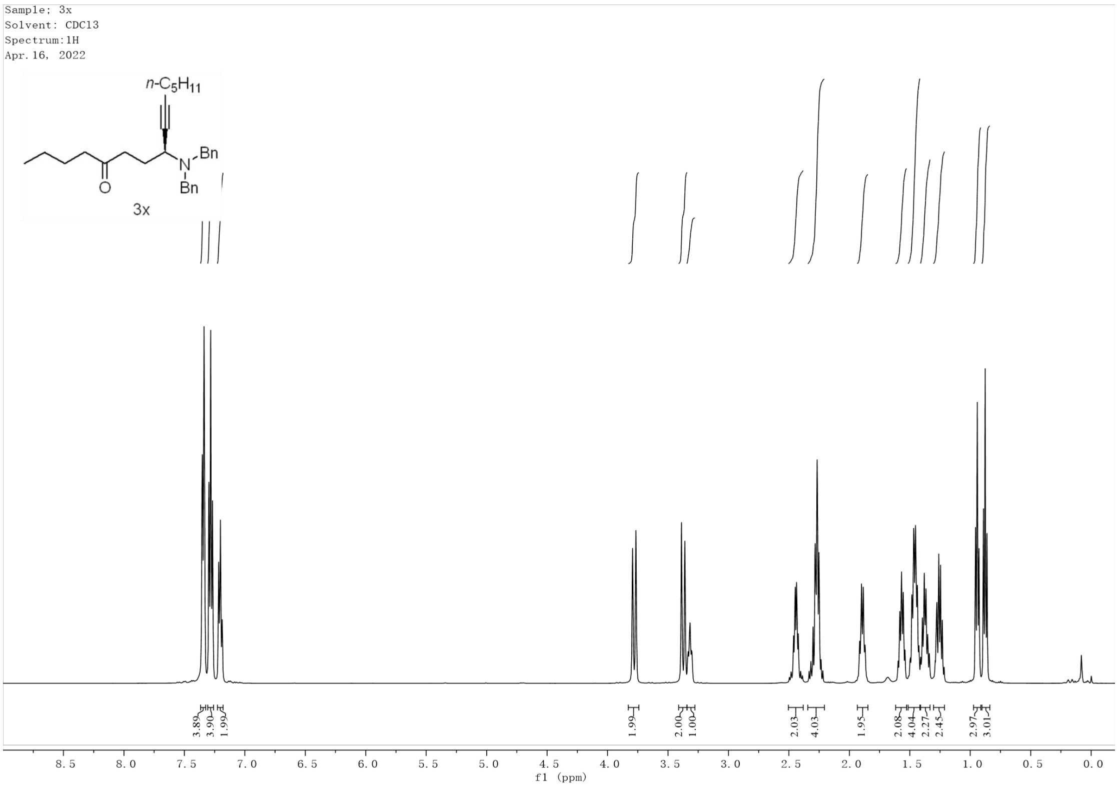

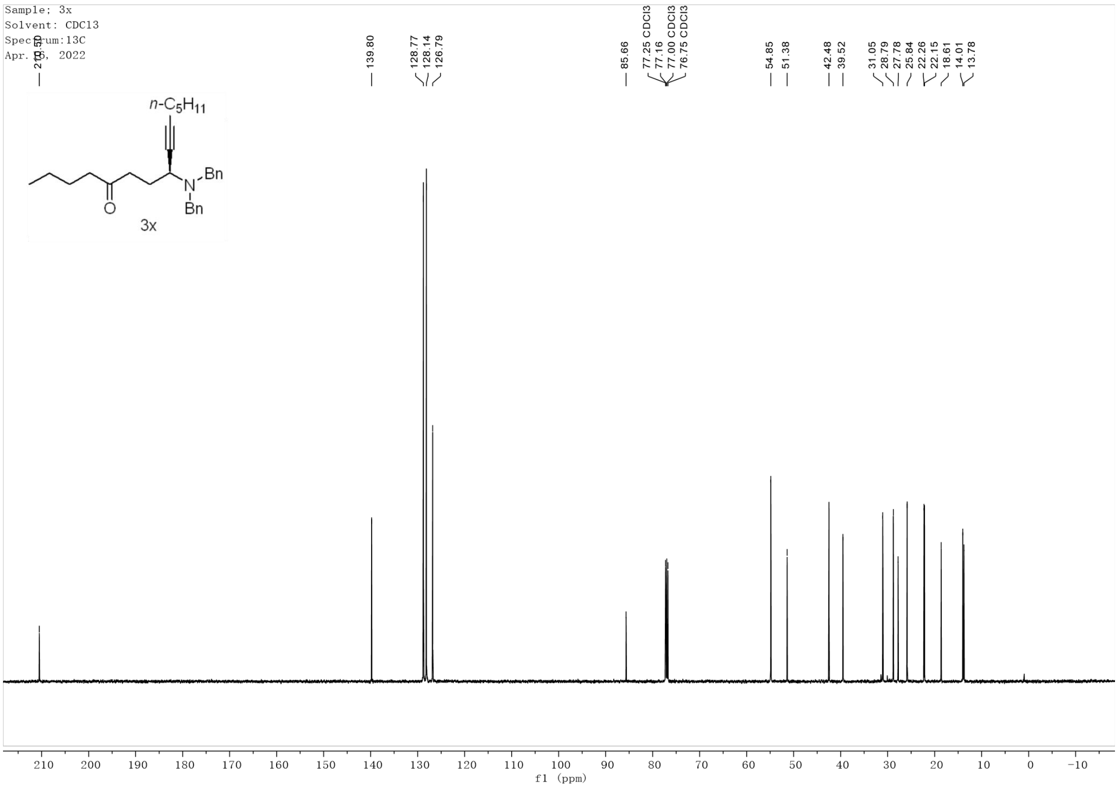

Supplementary Fig. 69. ^{1}H **NMR (400 MHz,** $CDCl_3$**) and** ^{13}C **NMR (100 MHz,** $CDCl_3$**) spectra of 3y**

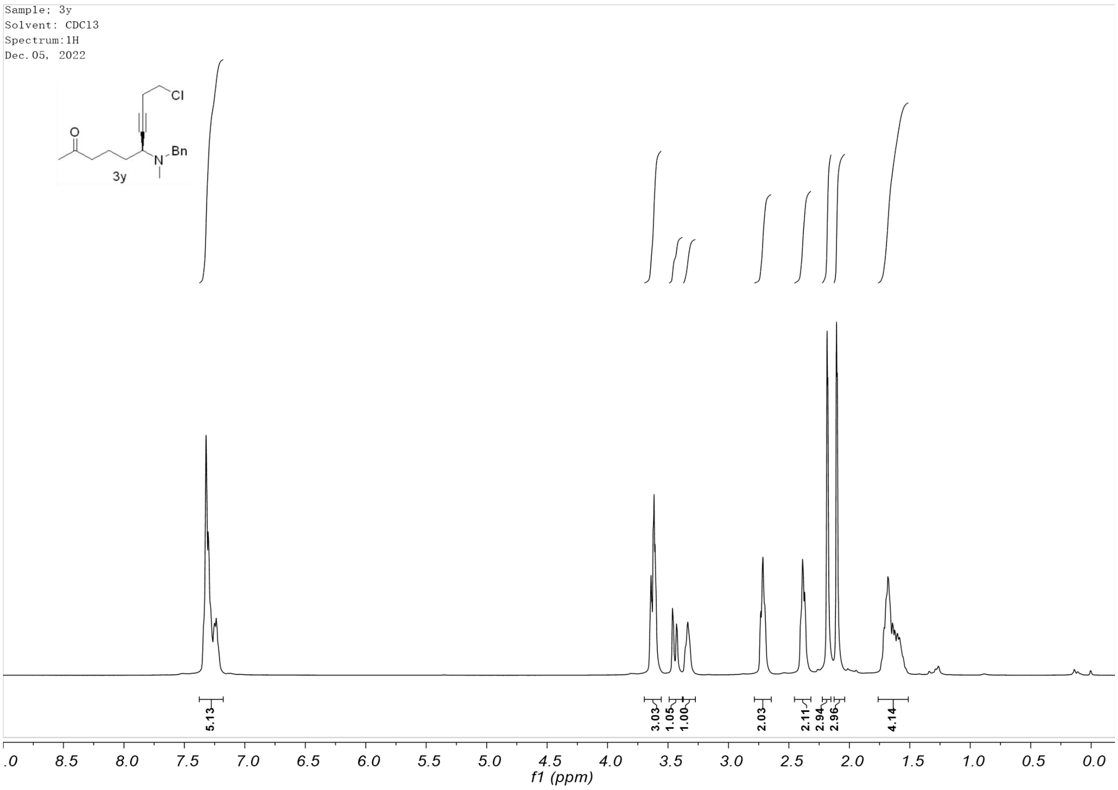

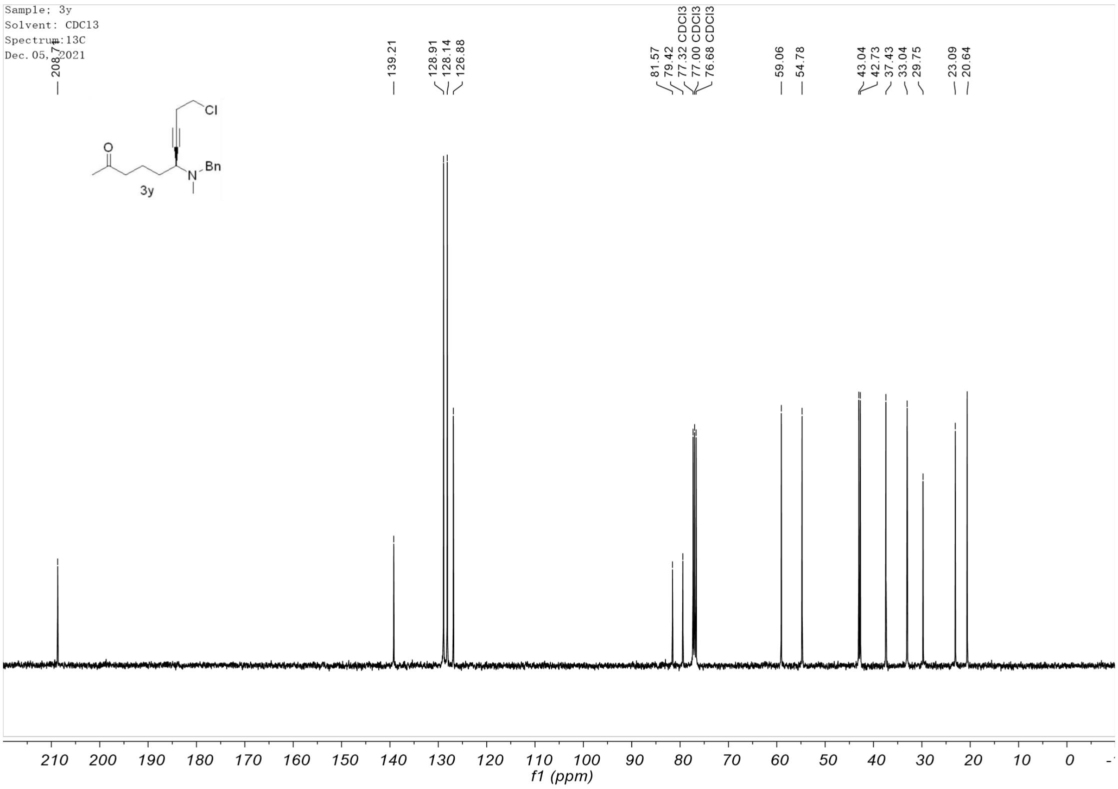

Supplementary Fig. 70. ^{1}H NMR (400 MHz, $CDCl_3$) and ^{13}C NMR (100 MHz, $CDCl_3$) spectra of 3z

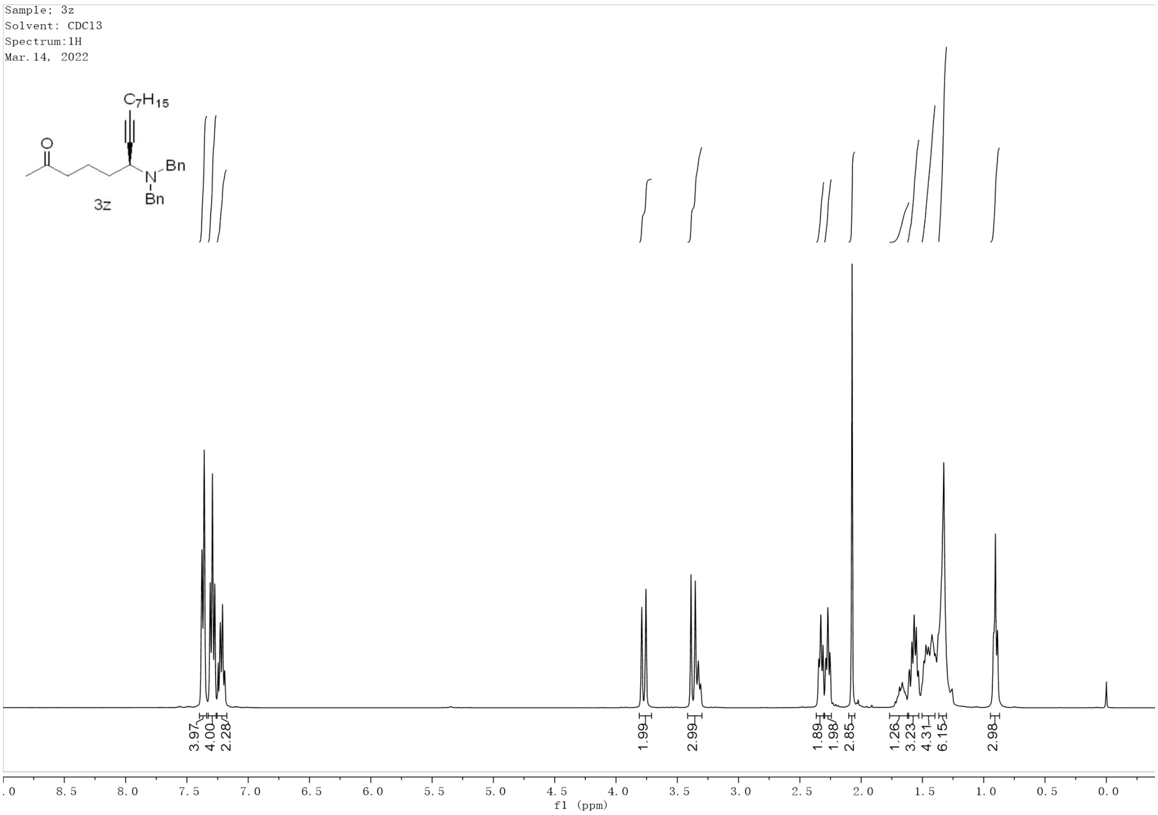

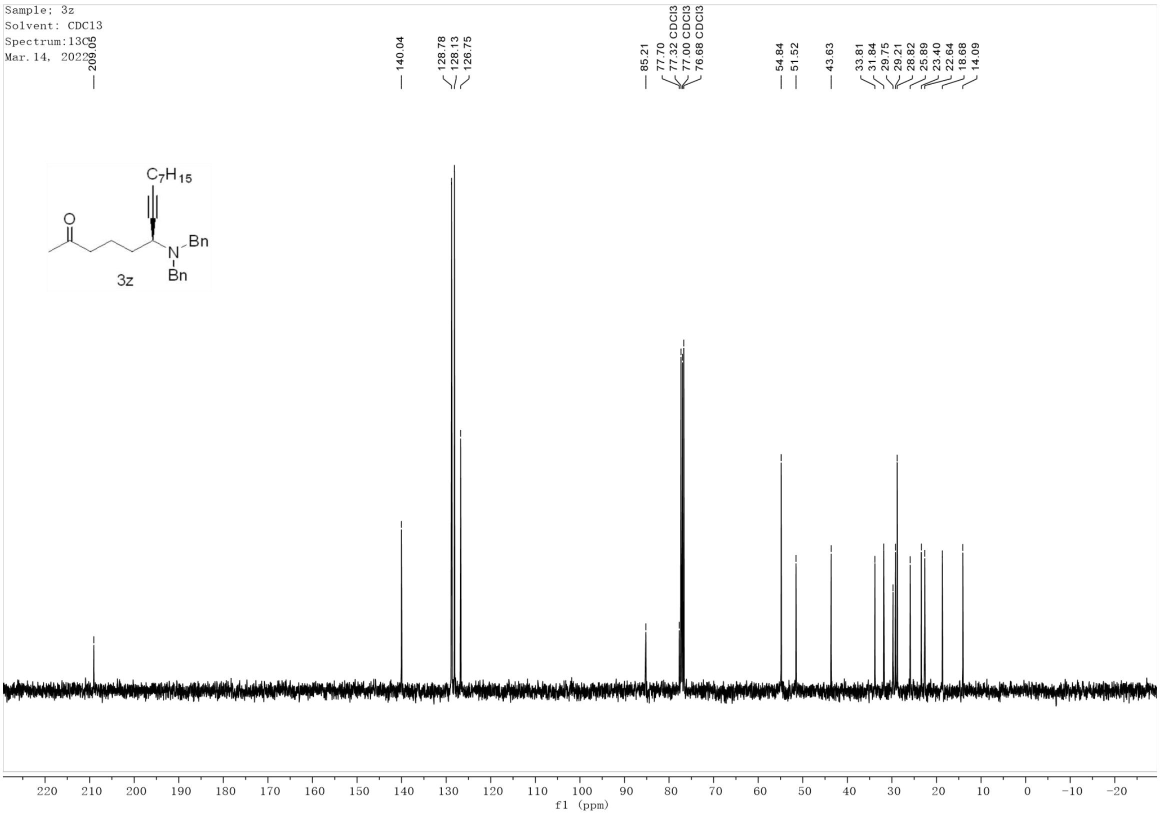

Supplementary Fig. 71. ¹H NMR (400 MHz, $CDCl_3$) and ¹³C NMR (100 MHz, $CDCl_3$) spectra of 3aa

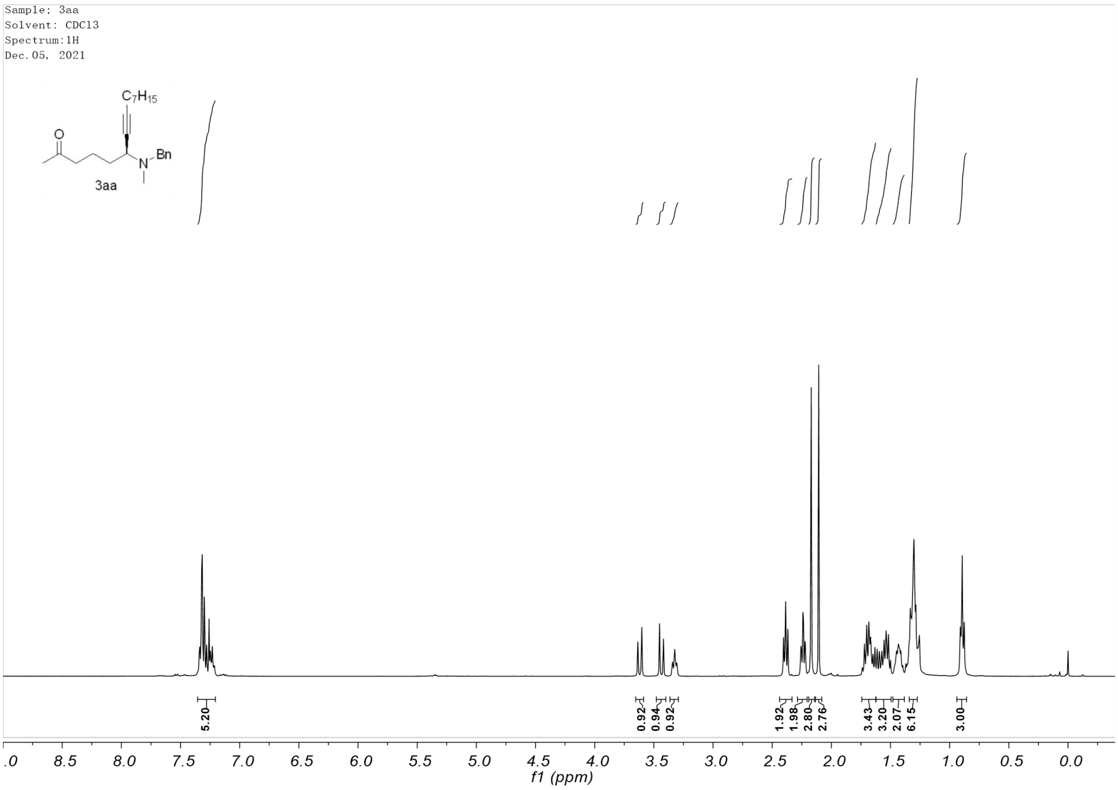

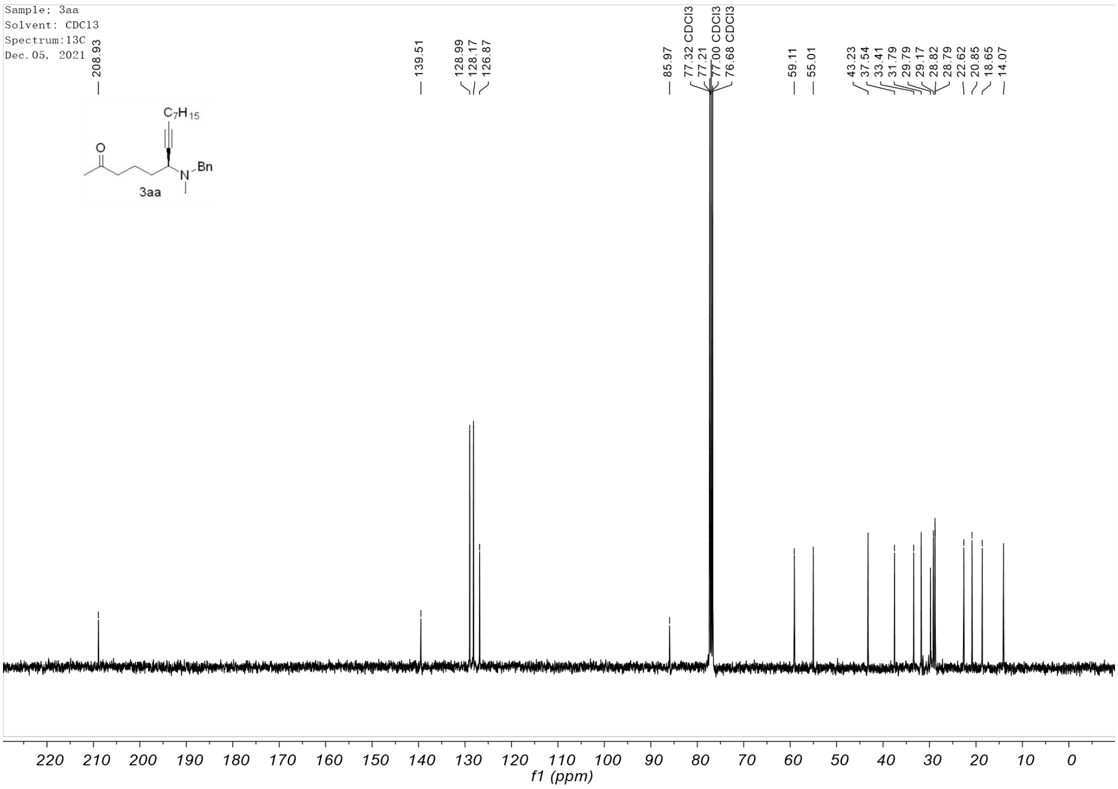

Supplementary Fig. 72. ^{1}H NMR (400 MHz, $CDCl_3$) and ^{13}C NMR (100 MHz, $CDCl_3$) spectra of 3ab

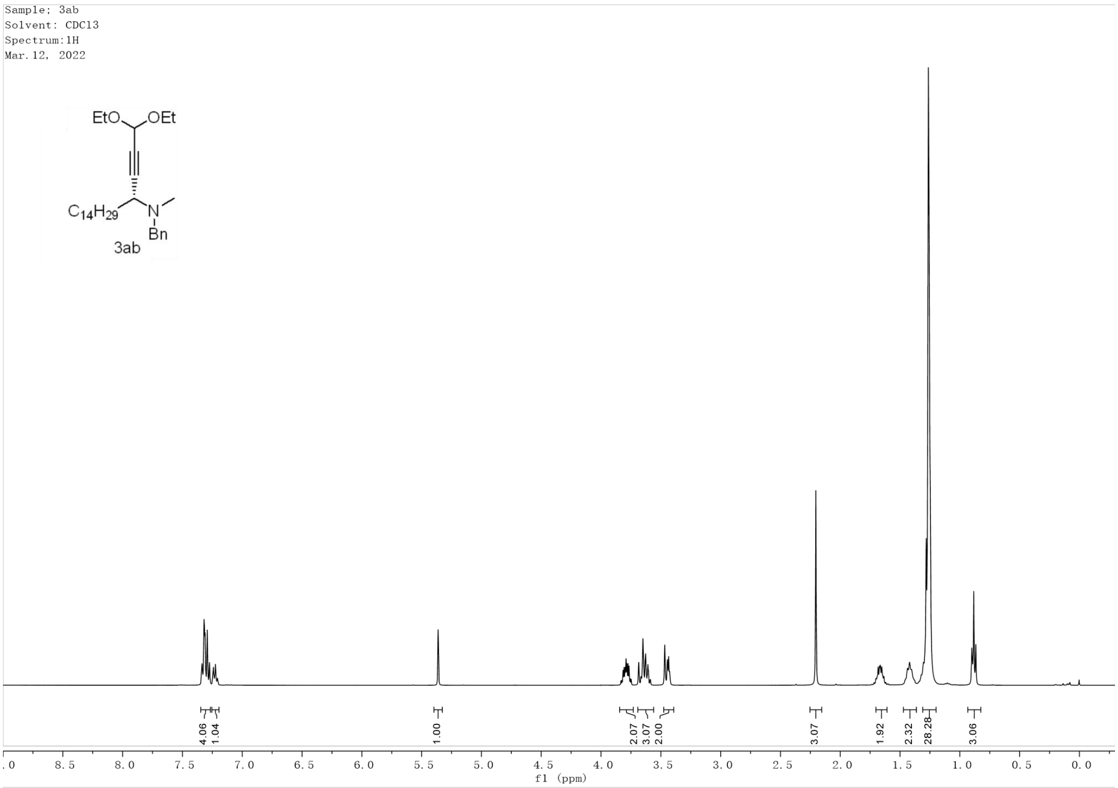

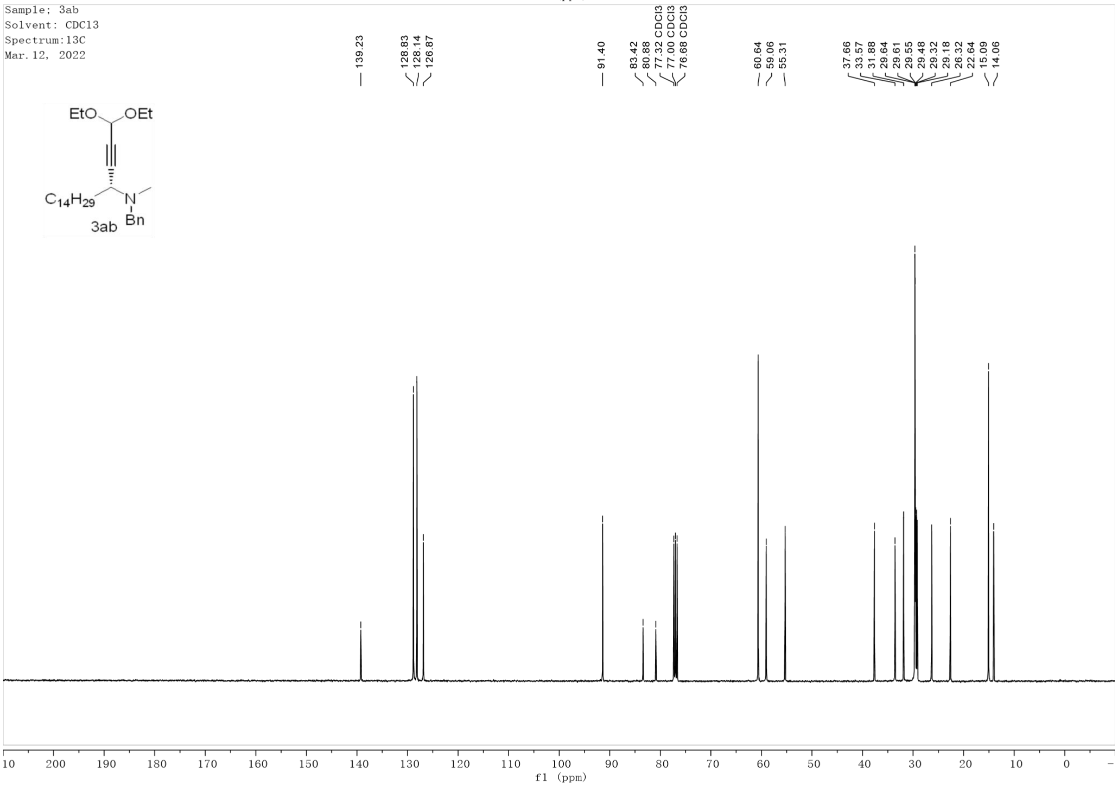

Supplementary Fig. 73. ^{1}H NMR (400 MHz, $CDCl_3$) and ^{13}C NMR (100 MHz, $CDCl_3$) spectra of 3ac

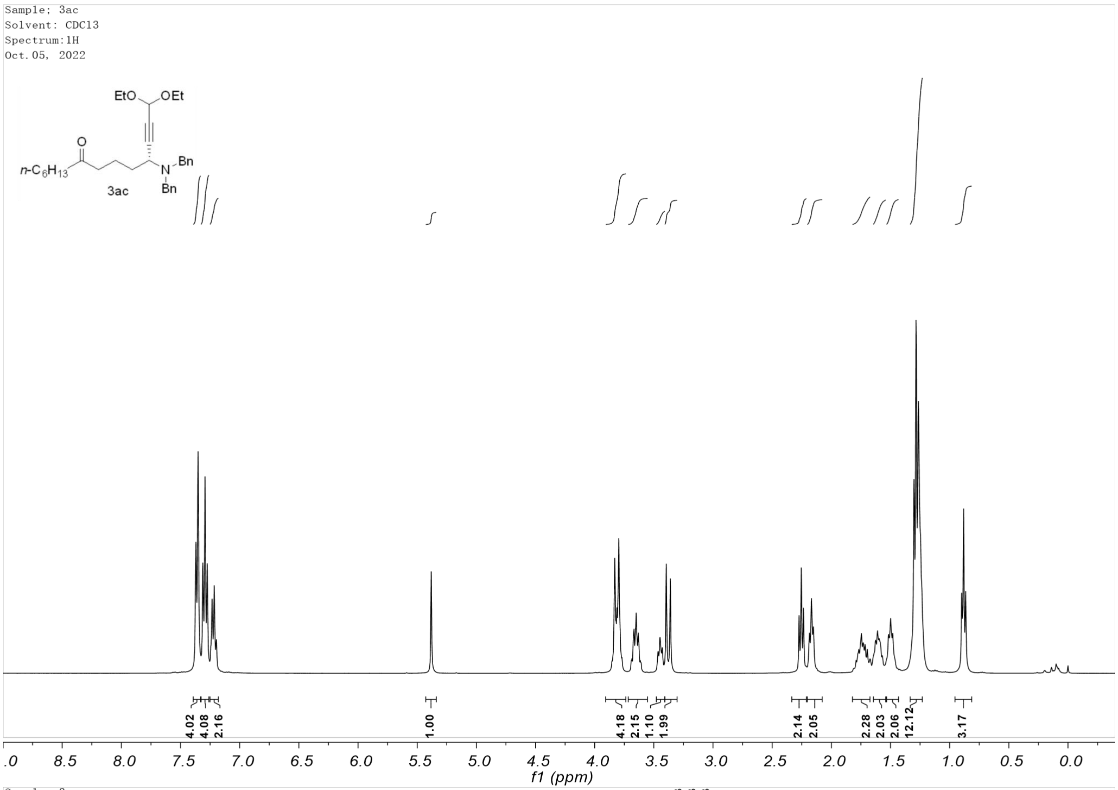

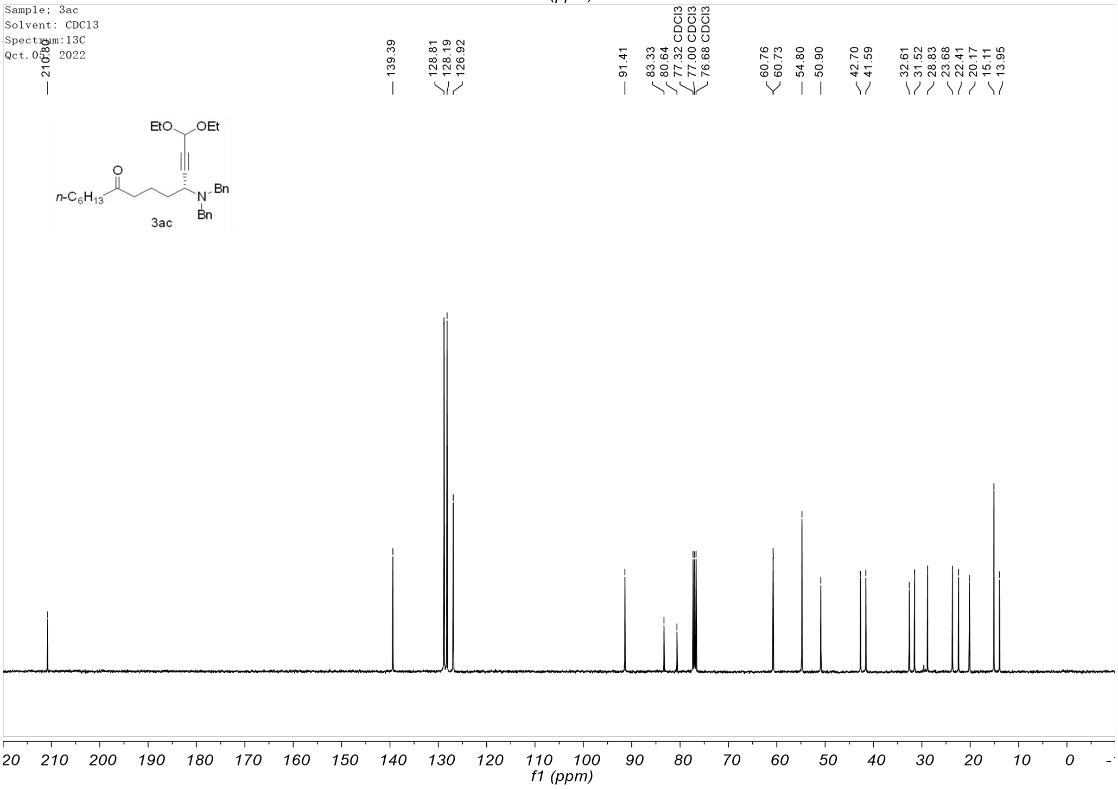

Supplementary Fig. 74. ^{1}H NMR (400 MHz, $CDCl_3$) and ^{13}C NMR (126 MHz, $CDCl_3$) spectra of 3ad

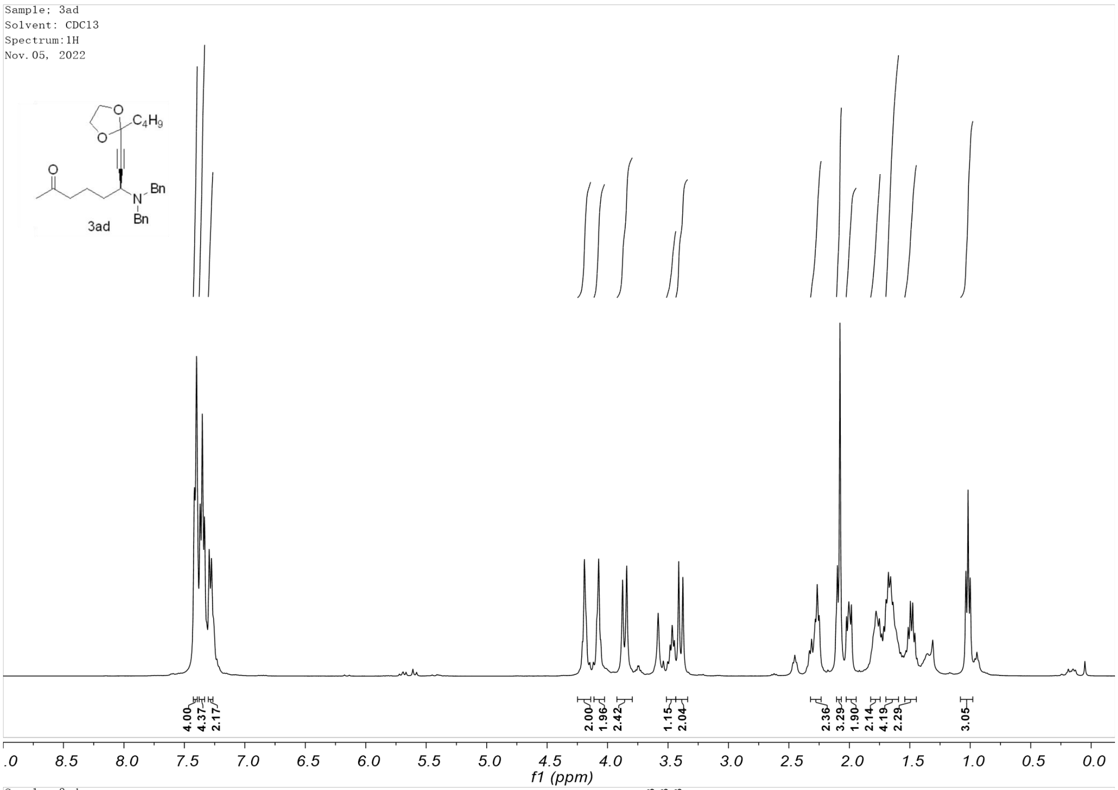

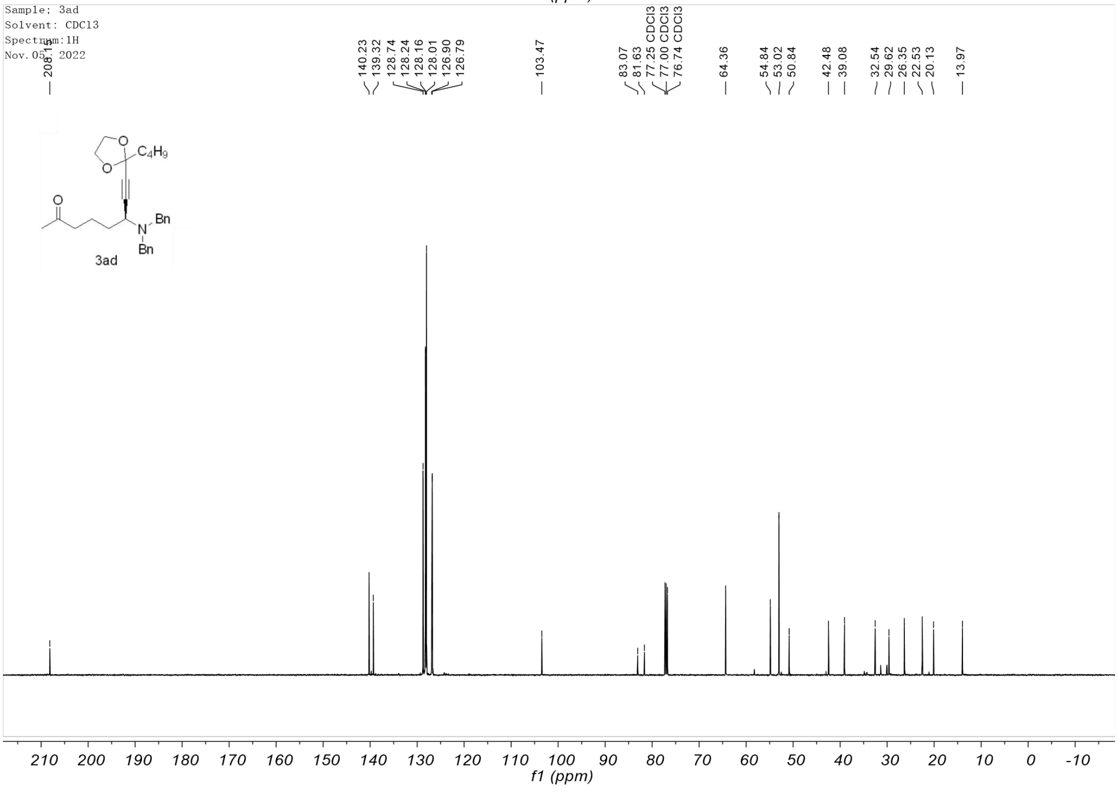

Supplementary Fig. 75. ^{1}H NMR (400 MHz, $CDCl_3$) and ^{13}C NMR (100 MHz, $CDCl_3$) spectra of A-3

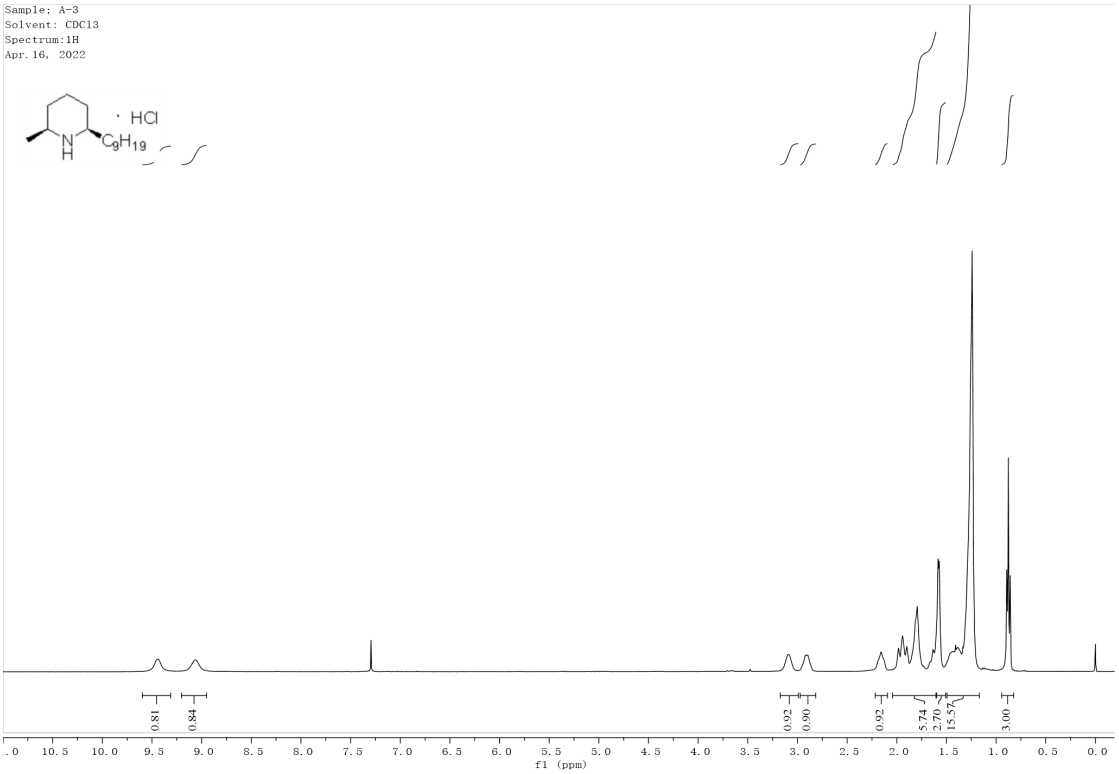

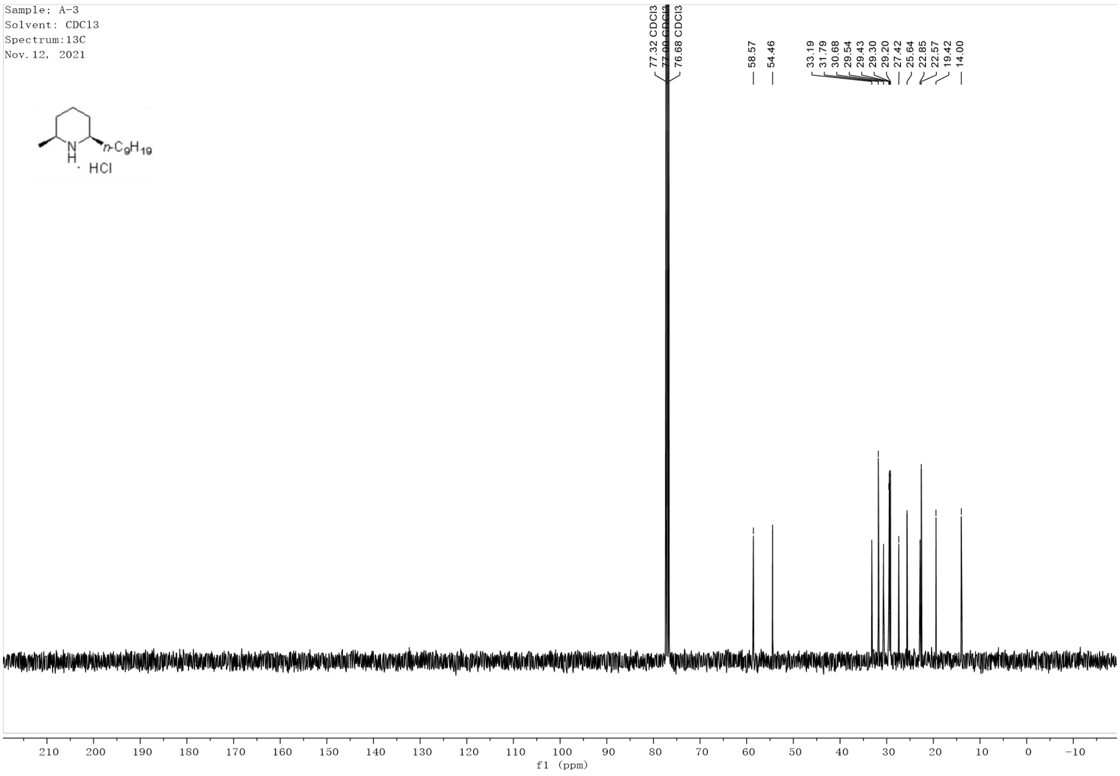

Supplementary Fig. 76. ^{1}H NMR (400 MHz, $CDCl_3$) and ^{13}C NMR (100 MHz, $CDCl_3$) spectra of A-4

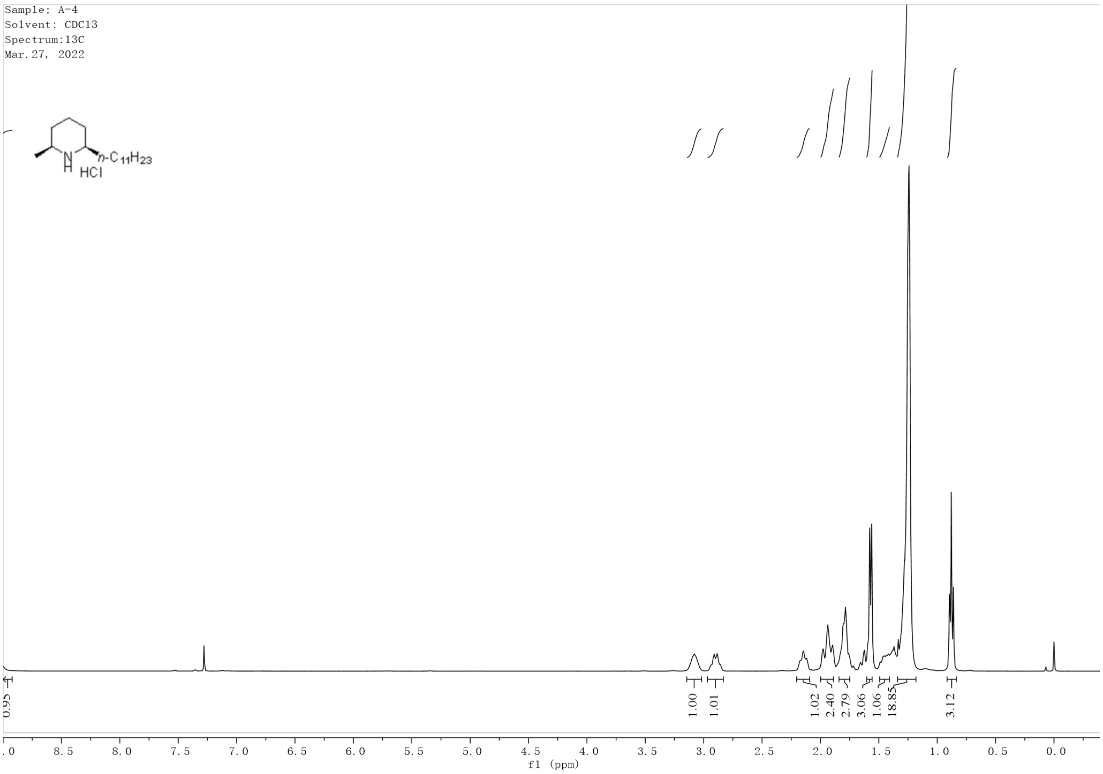

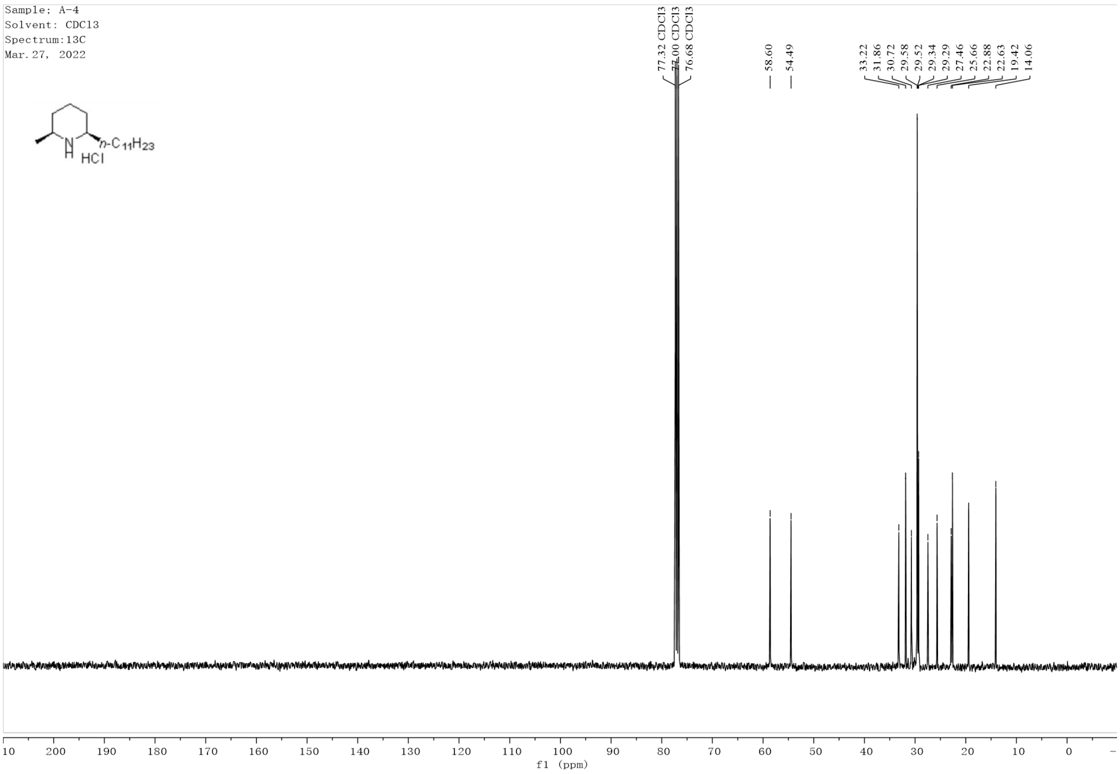

Supplementary Fig. 77. ^{1}H NMR (500 MHz, $CDCl_3$) and ^{13}C NMR (100 MHz, $CDCl_3$) spectra of A-5

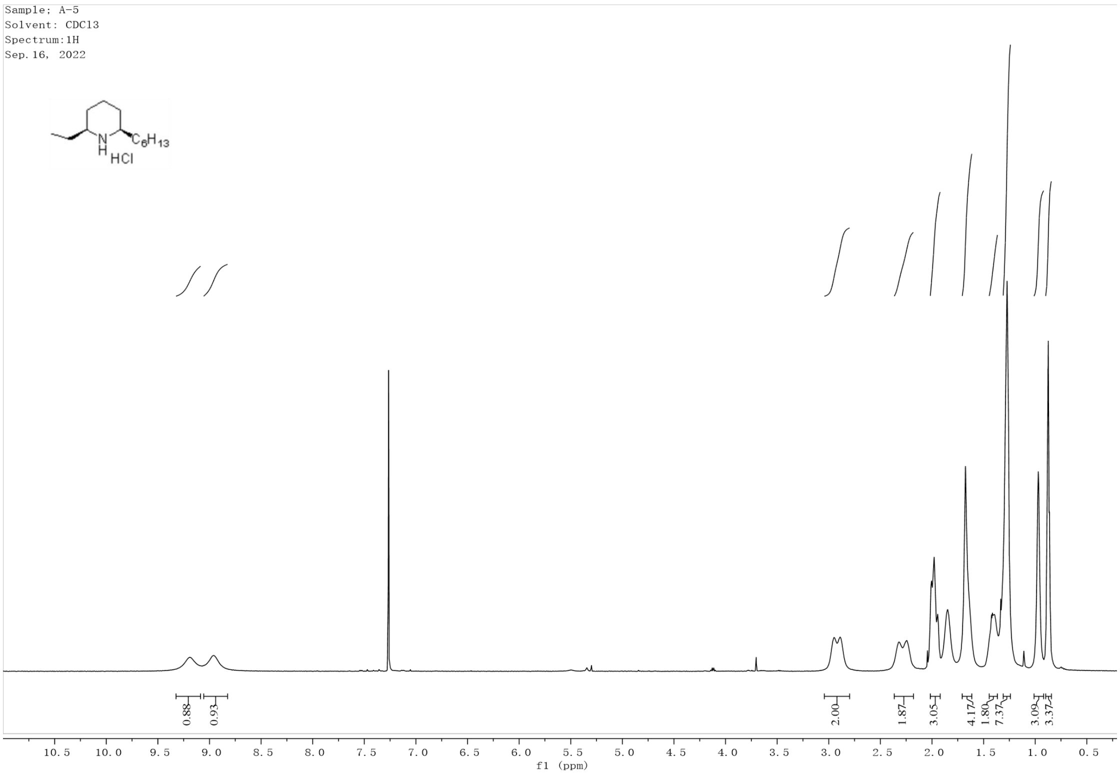

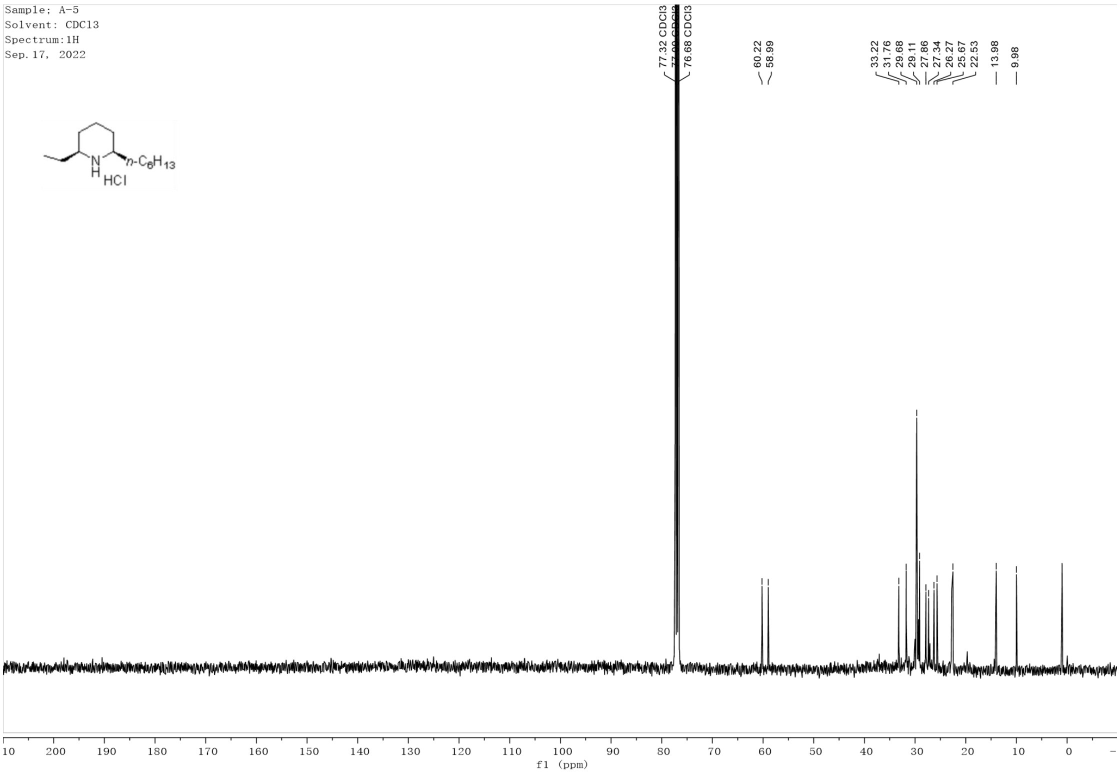

Supplementary Fig. 78. ^{1}H NMR (500 MHz, $CDCl_3$) and ^{13}C NMR (100 MHz, $CDCl_3$) spectra of A-2

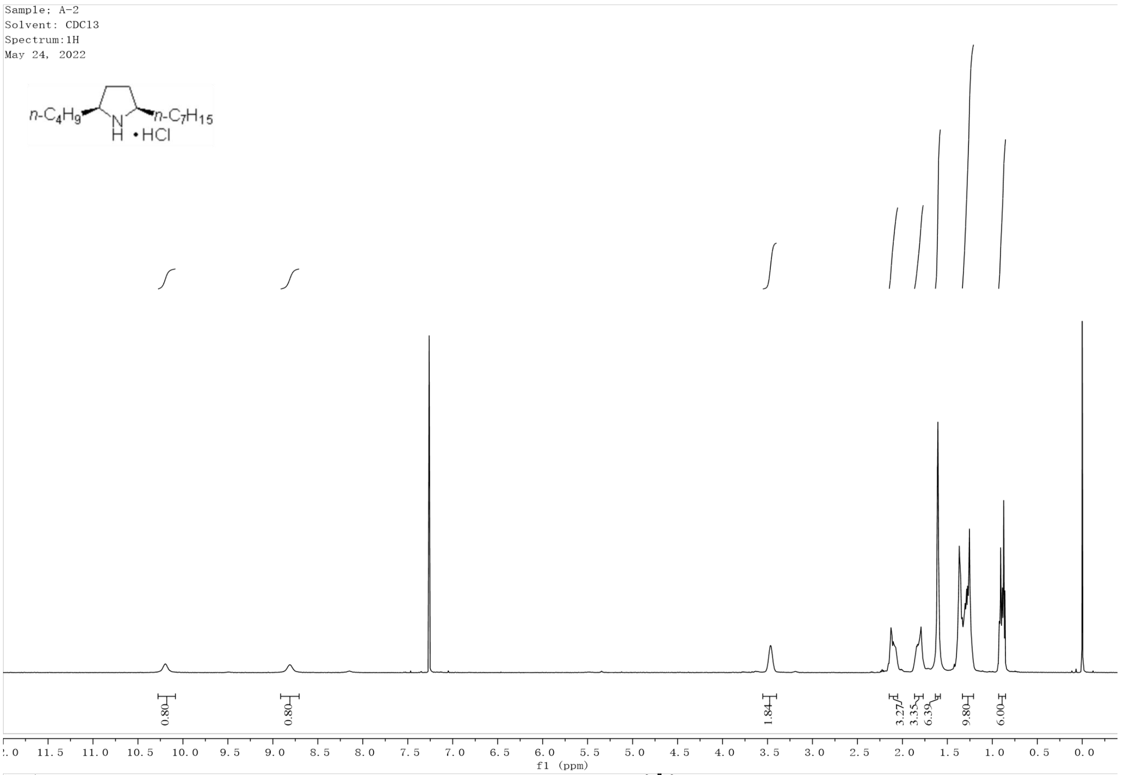

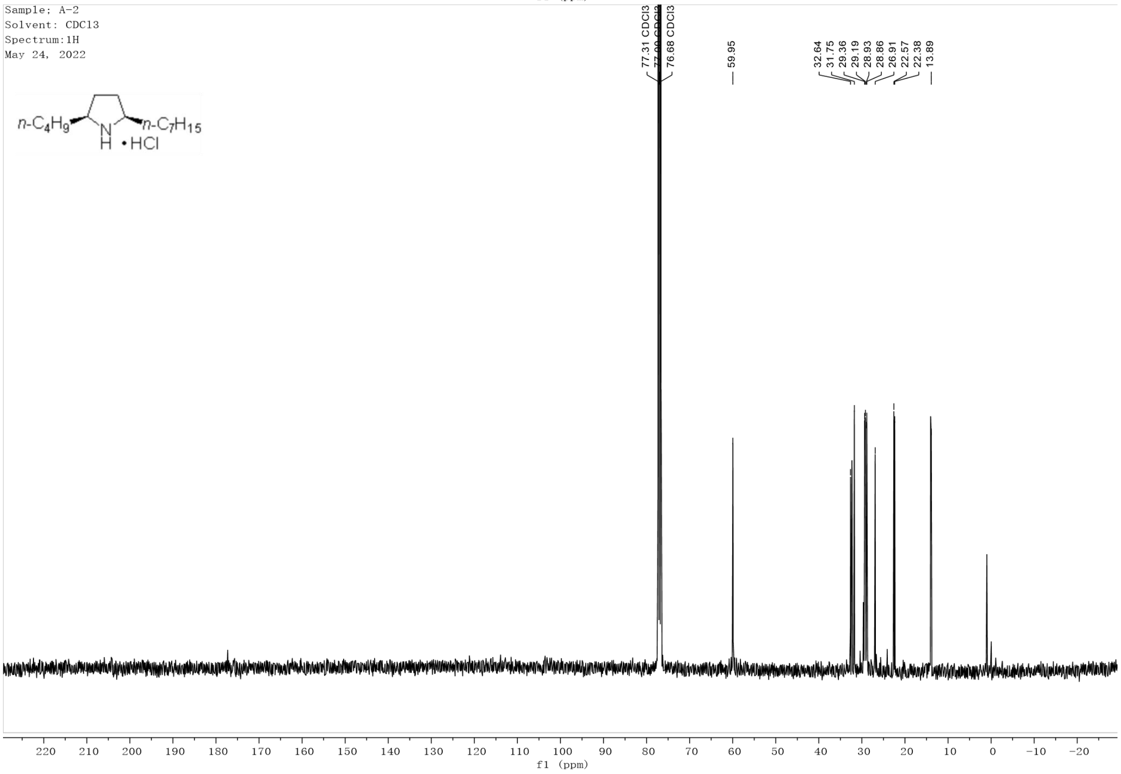

Supplementary Fig. 79. ^{1}H NMR (500 MHz, $CDCl_3$) and ^{13}C NMR (126 MHz, $CDCl_3$) spectra of A-6

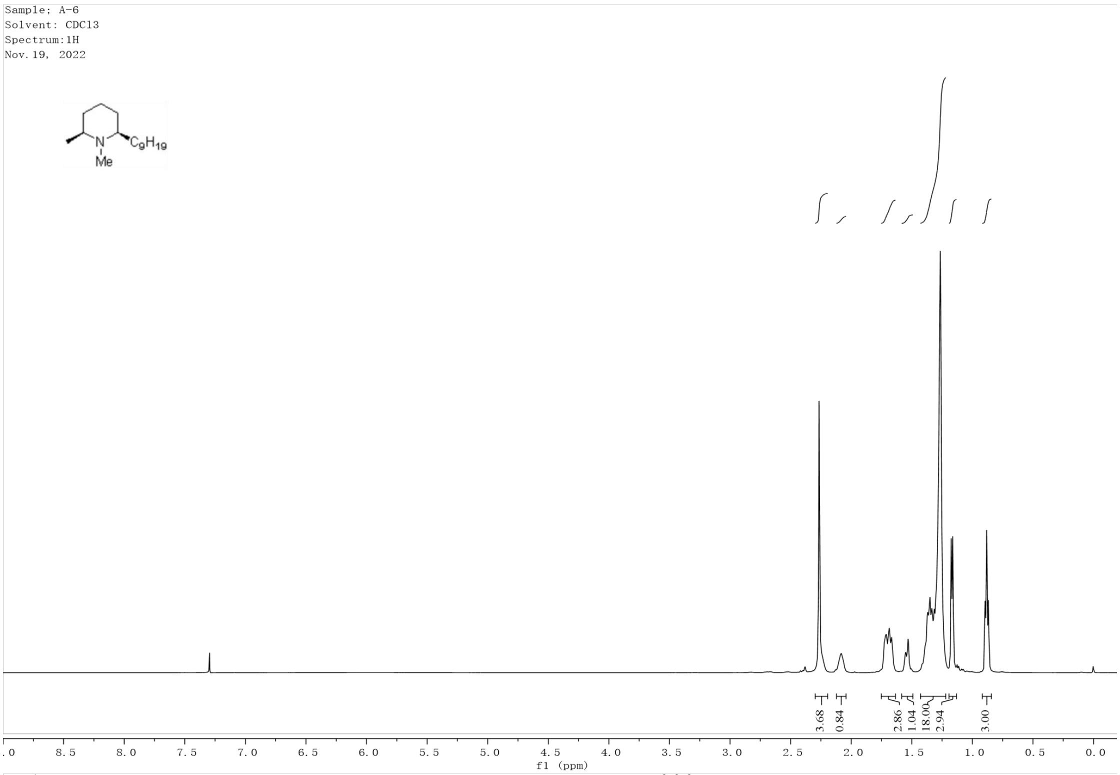

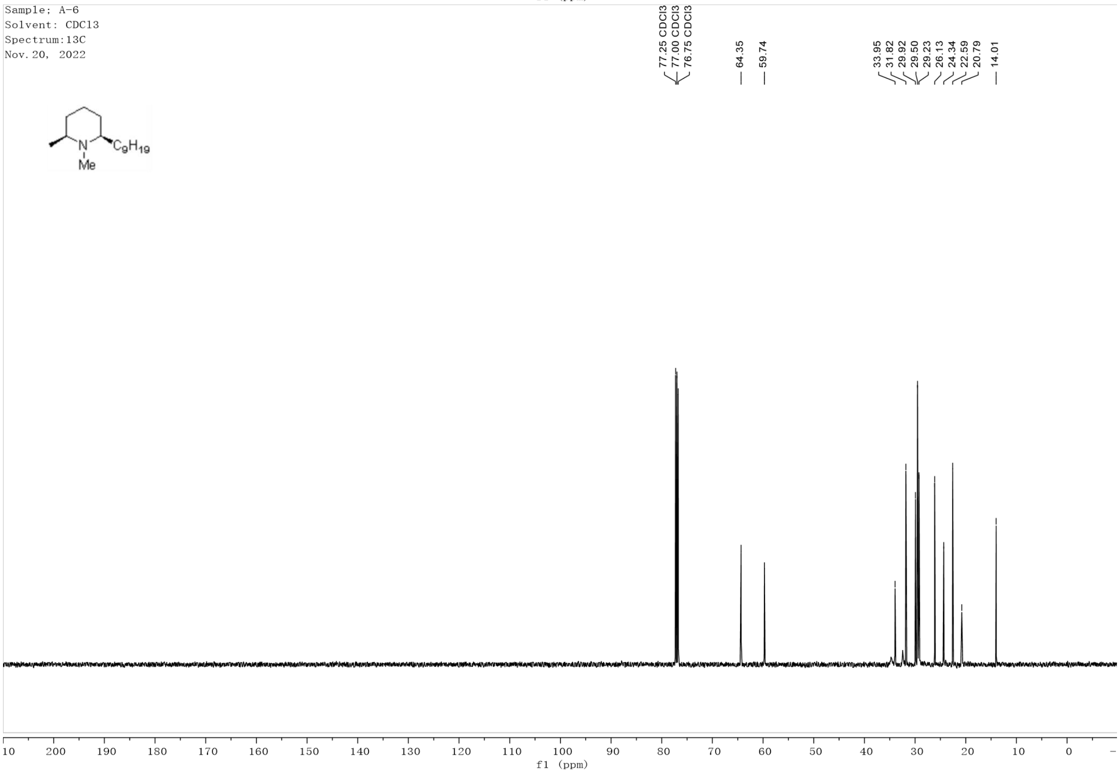

Supplementary Fig. 80. ^{1}H NMR (400 MHz, $CDCl_3$) and ^{13}C NMR (100 MHz, $CDCl_3$) spectra of A-1

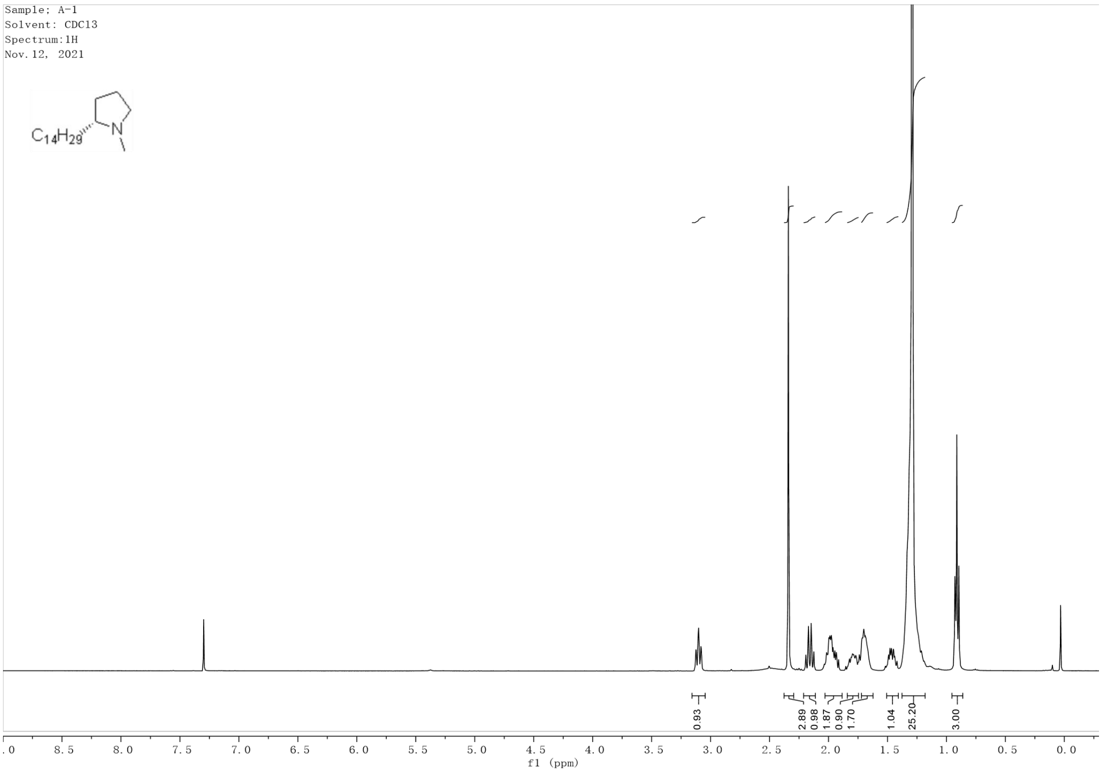

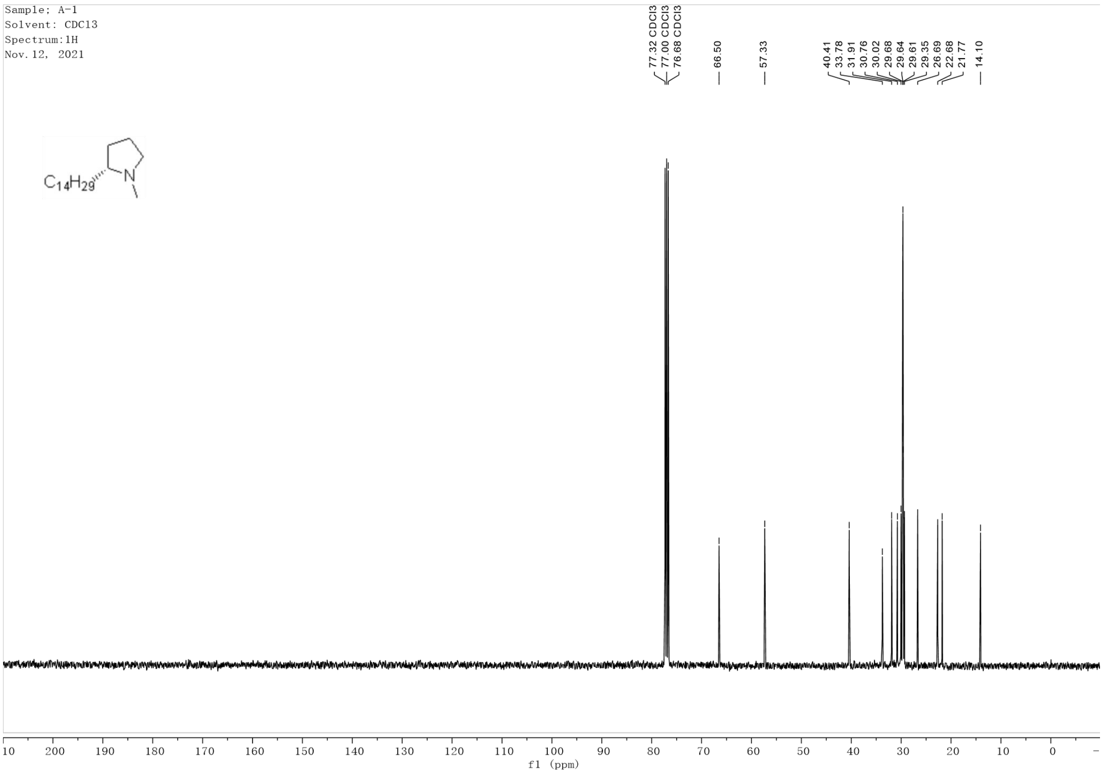

Supplementary Fig. 81. ^{1}H **NMR (400 MHz,** $CDCl_3$**) and** ^{13}C **NMR (100 MHz,** $CDCl_3$**) spectra of** ***Ent*-A-1**

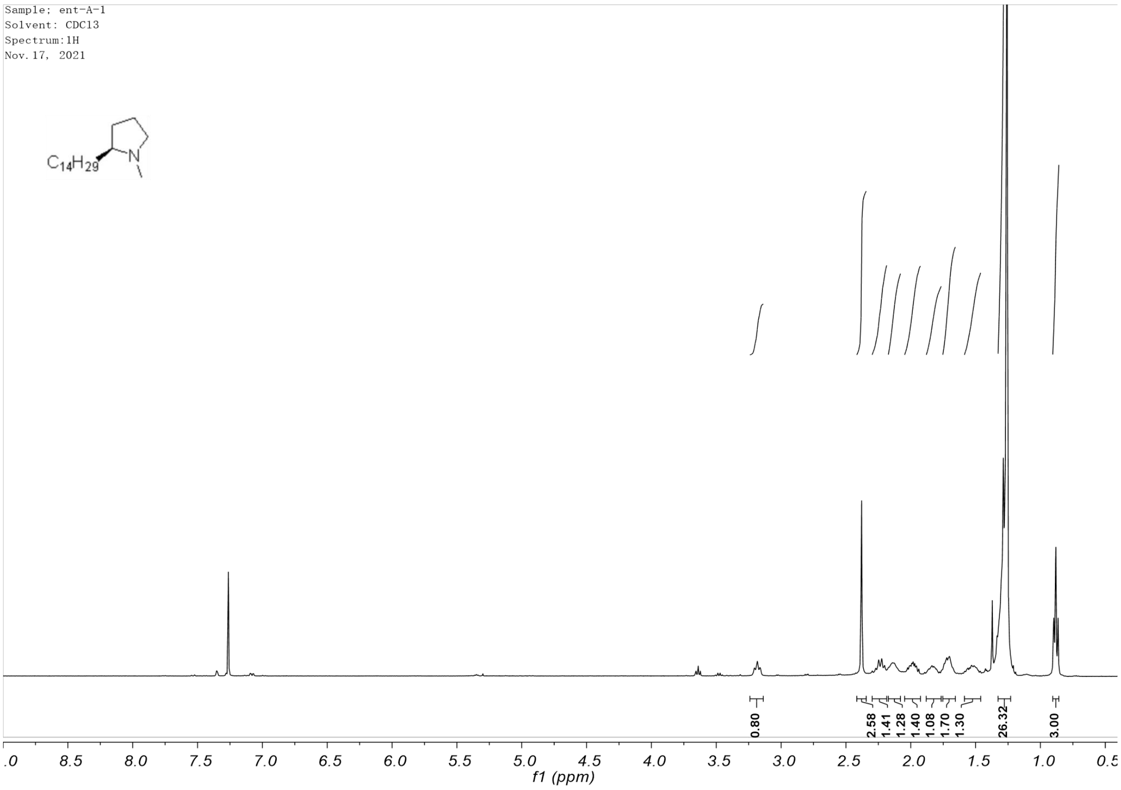

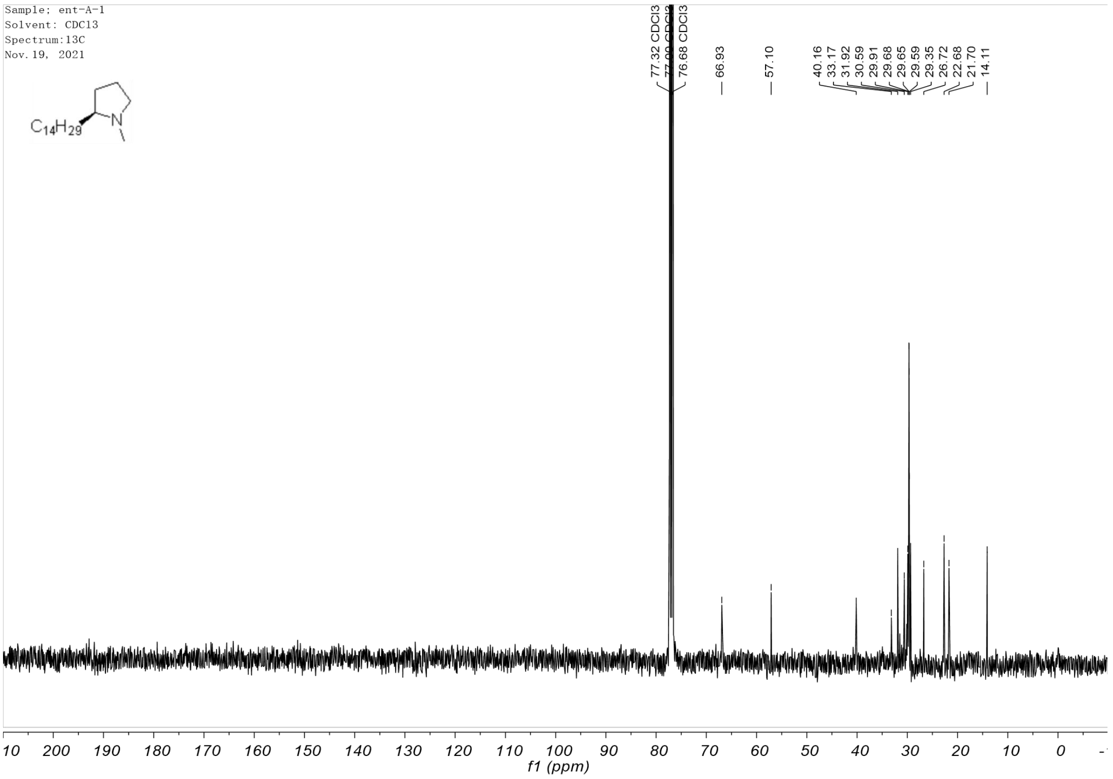

Supplementary Fig. 82. ^{1}H NMR (400 MHz, $CDCl_3$) and ^{13}C NMR (100 MHz, $CDCl_3$) spectra of A-7

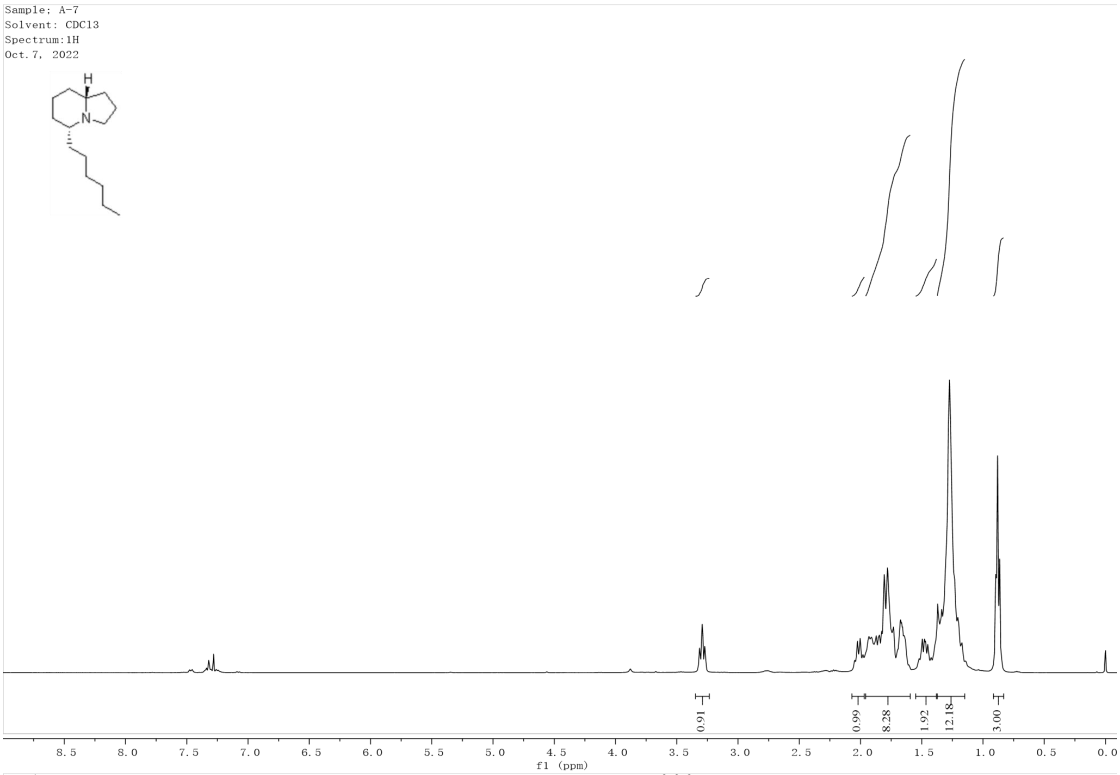

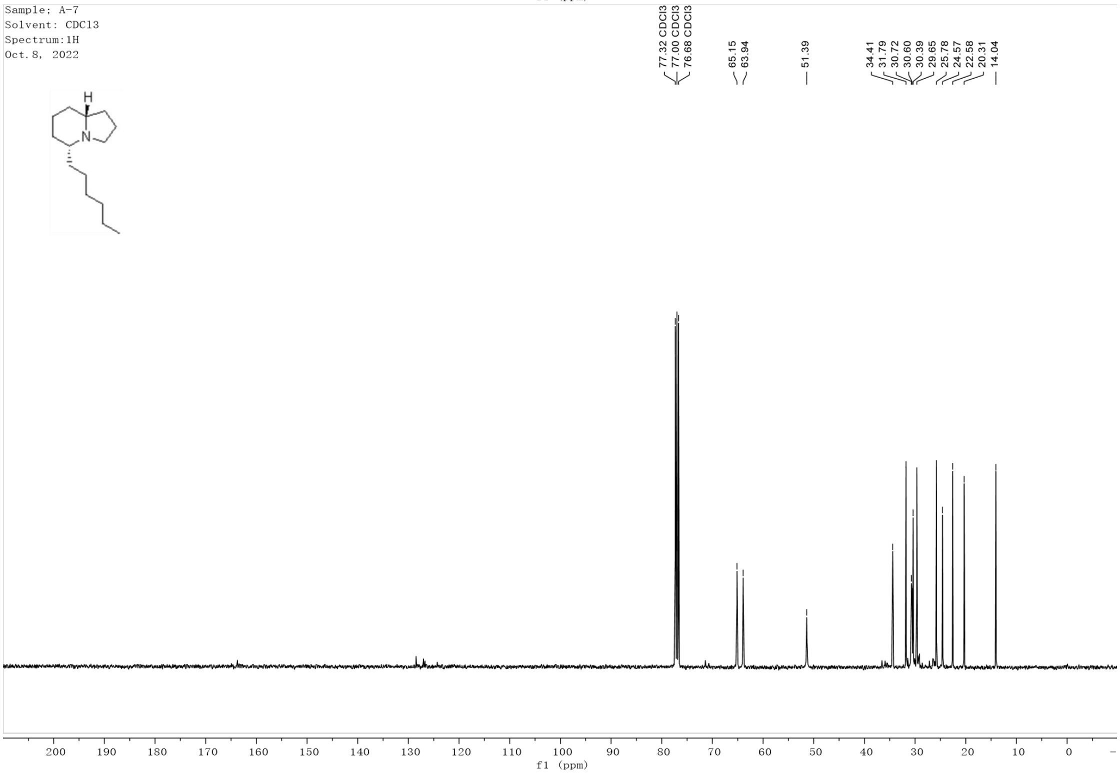

Supplementary Fig. 83. ^{1}H NMR (500 MHz, $CDCl_3$) and ^{13}C NMR (101 MHz, $CDCl_3$) spectra of A-8

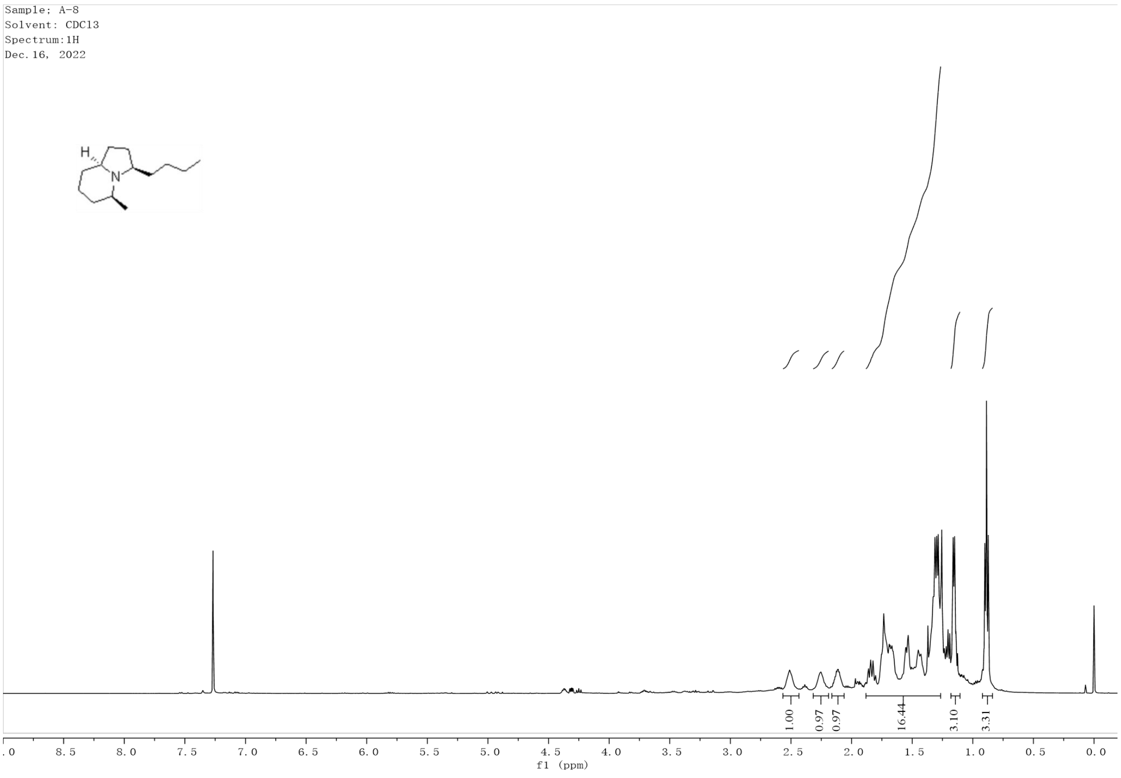

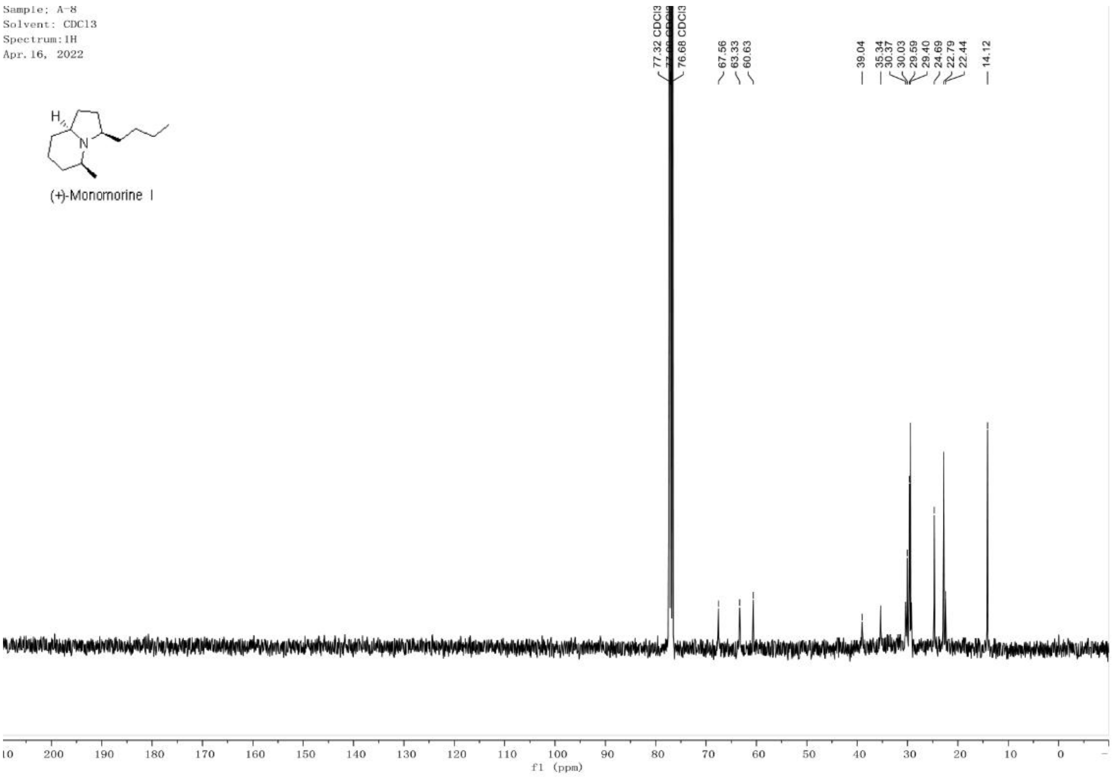

Supplementary Fig. 84. ^{1}**H NMR (500 MHz,** $CDCl_3$**) and** ^{13}**C NMR (101 MHz,** $CDCl_3$**) spectra of 4a**

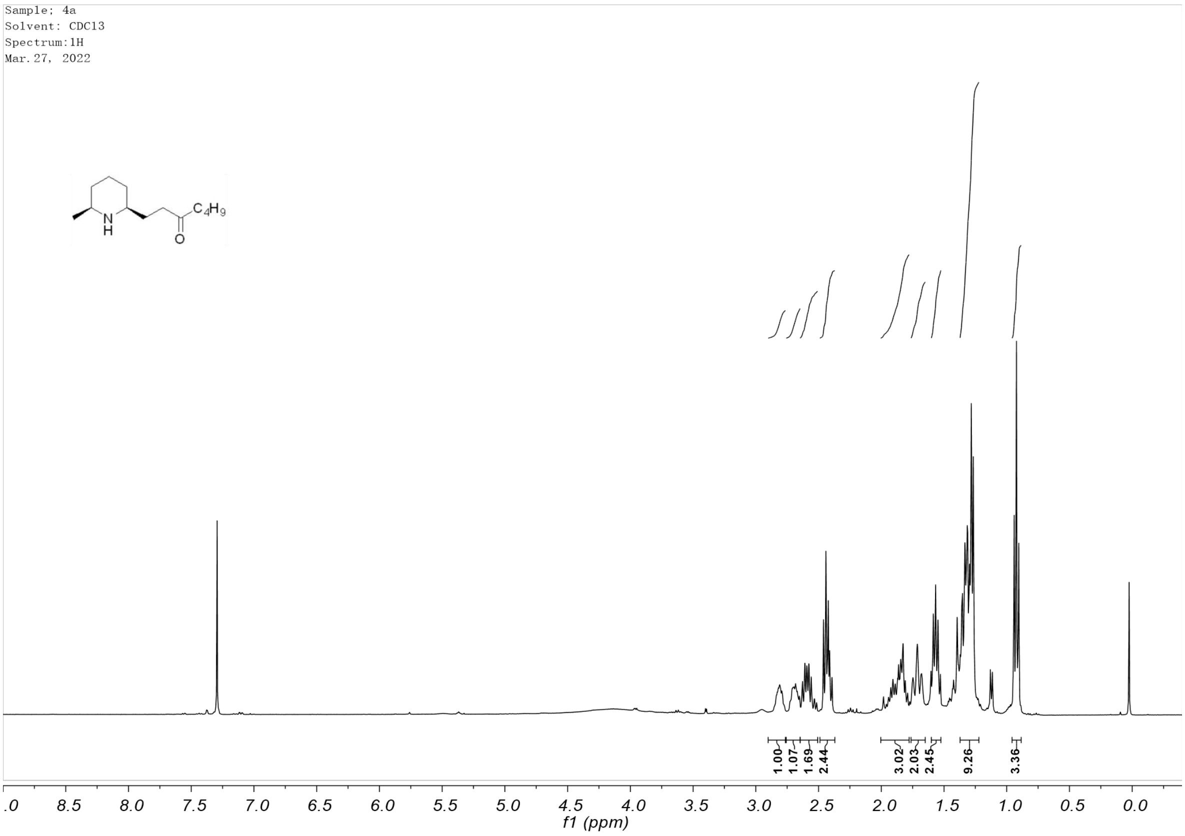

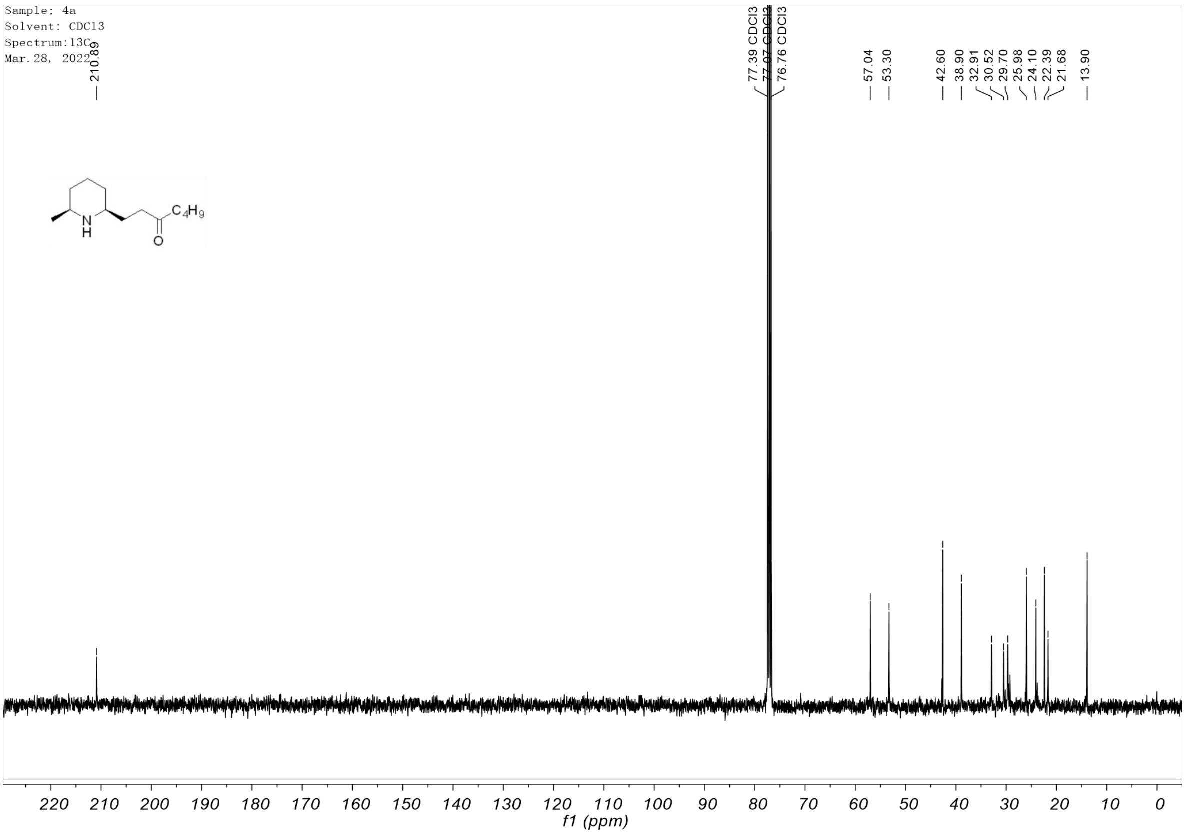

5. Supplementary References

1 Corre, Y., Rysak, V., Nagyházi, M., Kalocsai, D., Trivelli, X., Djukic, J. P., Agbossou-Niedercorn, F. & Michon, C. One-pot controlled reduction of conjugated amides by sequential double hydrosilylation catalyzed by an iridium (III) metallacycle. *Eur. J. Org. Chem.* **39**, 6212–6220 (2020).

2 Ramachandran. P. V. J. & Hamann, H. J. Ammonia-borane as a catalyst for the direct amidation of carboxylic acids. *Org. Lett.* **23**, 2938−2942 (2021).

3 Dai, F.-Y., Yang, Y.-H., Gu, J.-J., Fang, Z., Yang, Z., Liu, C.-K., He, W., Zhu, N., Lu, B. & Guo, K. Synthesis of tertiary aromatic amides from tertiary benzylamines via TBAI/TBHP-mediated C-N-bond cleavage. *ChemistrySelect*, **4**, 3500–3504 (2019).

4 Corre, Y., Trivelli, X., Capet, F., Djukic, J.-P., Agbossou-Niedercorn, F. & Michon, C. Efficient and selective hydrosilylation of secondary and tertiary amides catalyzed by an iridium (III) metallacycle: development and mechanistic investigation. *ChemCatChem*. *9*, 2009–2017 (2017).

5 Wang, S., Miao, R., Xia, Y. P., Wei, Y. F. & Luo, R. S., Ouyang. L. Ir-catalyzed *α*-alkylation of ketones with alcohols: one-step access to donepezil. *Synthesis*, **54**, 5253–5260 (2022).

6 Ong, D. Y., Fan, D. Y., Dixon, D. J. & Chiba, S. Transition-metal-free reductive functionalization of tertiary carboxamides and lactams for α-branched amine synthesis. *Angew. Chem. Int. Ed.* **59**, 11903–11907 (2020).

7 a) Starodubtseva, E. V. et al. Stereocontrolled approach to δ-amino acids by asymmetric hydrogenation of 5-acetylaminopent-4-enoic acid derivatives. *Russ. Chem. Bull., Int. Ed.* **59,** 1463–1466 (2010); b) Wang, X.-G., Ou. W., Liu, M.-H., Liu. Z.-J. & Huang, P.-Q. Tandem-catalysis-enabled highly chemoselective deoxygenative alkynylation and alkylation of tertiary amides: a versatile entry to functionalized α-substituted amines. *Org. Chem. Front.* **9**, 3237–3246 (2022).

8 Quntar, A. A. A., Dweik, H. & Dembitsky, V. Facile synthesis of 2-aminocyclobutenylphosphonates. *J. Org. Chem.* **56**, 137–142 (2020).

9 Snider, B. B. & Zhou, J. Y. Synthesis of (+)-Sch 642305 by a biomimetic transannular michael reaction. *Org. Lett.* **8**, 1283-1286 (2006).

10 a) Knopfel, T. F., Aschwanden, P., Ichikawa, T., Watanabe, Y. & Carreira, E. M. Readily Available biaryl *P,N*-ligands for asymmetric catalysis. *Angew. Chem. Int. Ed.*, **43**, 5971–5973(2004); b) Koradin, C., Gommermann, N., Polborn, K. & Knochel, P. Synthesis of enantiomerically enriched

propargylamines by copper-catalyzed addition of alkynes to enamines. *Chem. Eur. J.* **9**, 2797–2811 (2003).

11 a) Gommermann, N., Koradin, X., Polborn, K. & Knochel, P. Enantioselective, copper (I)-catalyzed three-component reaction for the preparation of propargylamines. *Angew. Chem. Int. Ed.*, **42**, 5763–5766 (2003); b) Rokade, B. V. & Guiry, P. J. Diastereofacial π stacking as an approach to access an axially chiral *P,N*-ligand for asymmetric catalysis. *ACS Catal.* **7**, 2334−2338 (2017); c) Liu, Q., Xu, H.-B., Li, Y.-L., Yao, Y., Zhang, X., Guo, Y.-L. & Ma, S.-M. Pyrinap ligands for enantioselective syntheses of amines. *Nat. Commun.*, **12**, 19 (2021).

12 Simon, R. C., Fuchs, C. S., Lechner, H., Zepeck, F. & Kroutil, W. Concise chemoenzymatic three-step total synthesis of isosolenopsin through medium engineering. *Eur. J. Org. Chem.* 3397–3402. (2013).

13 a) Wang, X., Dong, Y., Sun, J., Xu, X., Li, R. & Hu, Y. Nonracemic betti base as a new chiral auxiliary: application to total syntheses of enantiopure (*2S,6R*)-dihydropinidine and (*2S,6R*)-isosolenopsins. *J. Org. Chem.*, **70**, 1897–1900 (2005); b) Poerwono, H., Higashiyama, K., Yamauchi, T., Kubo, H., Ohmiya, S. & Takahashi, H. Stereocontrolled preparation of *cis*- and *rans*-2,6-dialkylpiperidines via diastereoselective reaction of 1-aza-4-oxabicyclo [4.3.0] nonane derivatives with grignard reagents. *Tetrahedron*, **54**, 13955–13970 (1988).

14 a) Arseniyadis, S., Huang, P.-Q., Piveteau, D. & Husson, H. P. Asymmetric synthesis: XII: stereocontrolled electrophilic-nucleophilic α,α'-substitution of the pyrrolidine ring. *Tetrahedron* **44**, 2457–2470 (1988); b) Davis, F. A., Xu, H., Wu, Y. Z. & Zhang, J. Y. Asymmetric synthesis of polyfunctionalized pyrrolidines from sulfinimine-derived pyrrolidine 2-phosphonates. synthesis of Pyrrolidine 225C. *Org. Lett*. **8**, 2273–2276 (2006).

15 Shu, C., Liu, M. Q., Wang, S. S. & Ye, L. W. Gold-catalyzed oxidative cyclization of chiral homopropargyl amides: synthesis of enantioenriched γ-lactams. *J. Org. Chem.* **78**, 3292−3299 (2013).

16 a) Yu, R. T., Lee, E. E., Malik, G. & Rovis, T. Total synthesis of indolizidine alkaloid (−)-209D: overriding substrate bias in the asymmetric rhodium-catalyzed [2+2+2] Cycloaddition. *Angew. Chem., Int. Ed.*, **48**, 2379–2382 (2009); b) Koo, S. M., Vendola, A. J., Momm, S. N. & Morken. J. P. Alkyl group migration in Ni-catalyzed conjunctive coupling with C(sp^3) electrophiles: reaction development and application to targets of interest. *Org. Lett.*, **22**, 666–669 (2020).

17 a) McManus, J. B., Onuska, N. P. R. & Nicewicz, D. A. Generation and alkylation of alpha-carbamyl radicals via organic photoredox catalysis. *J. Am. Chem. Soc.* **140**, 9056-9060 (2018); b) Toyooka, N.; Zhou, D. J. & Nemoto, H. Enantioselective syntheses of (–)- and (+)-monomorine I. *J. Org. Chem.* **73**, 4575–4577 (2008); c) Mori, M., Hori, M. & Sato, R. Y.

Atmospheric nitrogen fixation. short-step synthesis of monomorine I. *J. Org. Chem.* **63**, 4832-4833 (1998).
